# TANGO: a placebo-controlled randomized phase 2 study of efficacy and safety of the anti-tau monoclonal antibody gosuranemab in early Alzheimer’s disease

**DOI:** 10.1038/s43587-023-00523-w

**Published:** 2023-11-27

**Authors:** Melanie Shulman, Jessica Kong, John O’Gorman, Elena Ratti, Rajasimhan Rajagovindan, Louis Viollet, Ellen Huang, Sanjiv Sharma, Annie M. Racine, Julie Czerkowicz, Danielle Graham, Yumeng Li, Heike Hering, Samantha Budd Haeberlein

**Affiliations:** 1 Biogen, Cambridge, MA USA; 2 Moderna, Cambridge, MA USA; 3 CenExel AMRI, Toms River, NJ USA; 4 Present Address: Takeda Pharmaceuticals, Cambridge, MA USA; 5 Present Address: Vigil Neuroscience, Cambridge, MA USA; 6 Present Address: Alexion, AstraZeneca Rare Disease, Boston, MA USA; 7 Present Address: Enigma Biomedical USA, Knoxville, TN USA

**Keywords:** Alzheimer's disease, Alzheimer's disease

## Abstract

In Alzheimer’s disease, the spread of aberrantly phosphorylated tau is an important criterion in the Braak staging of disease severity and correlates with disease symptomatology. Here, we report the results of TANGO (NCT03352557), a randomized, double-blind, placebo-controlled, parallel-group and multiple-dose long-term trial of gosuranemab—a monoclonal antibody to N-terminal tau—in patients with early Alzheimer’s disease. The primary objective was to assess the safety and tolerability of gosuranemab compared to placebo. The secondary objectives were to assess the efficacy of multiple doses of gosuranemab in slowing cognitive and functional impairment (using the Clinical Dementia Rating Scale Sum of Boxes (CDR-SB) scores at week 78) and evaluate the immunogenicity of gosuranemab (using the incidence of anti-gosuranemab antibody responses). Participants were randomized (*n* = 654); received (*n* = 650) low-dose (125 mg once every 4 weeks (q4w), *n* = 58; 375 mg q12w, *n* = 58), intermediate-dose (600 mg q4w, *n* = 106) or high-dose (2,000 mg q4w, *n* = 214) gosuranemab or placebo (q4w, *n* = 214) intravenously for 78 weeks; and assigned to cerebrospinal fluid (*n* = 327) and/or tau positron emission tomography (*n* = 357) biomarker substudies. Gosuranemab had an acceptable safety profile and was generally well tolerated (incidence of serious adverse events: placebo, 12.1%; low dose, 10.3%; intermediate dose, 12.3%; high dose, 11.7%). The incidence of treatment-emergent gosuranemab antibody responses was low at all time points. No significant effects were identified in cognitive and functional tests as no dose resulted in a favorable change from the baseline CDR-SB score at week 78 compared to placebo control (adjusted mean change: placebo, 1.85; low dose, 2.20; intermediate dose, 2.24; high dose, 1.85). At week 76, all doses caused significant (*P* < 0.0001) reductions in the cerebrospinal fluid levels of unbound N-terminal tau compared to placebo.

## Abstract (web summary)

The authors present the results of a phase 2 study of gosuranemab, a monoclonal antibody targeting N-terminal tau, in patients with early Alzheimer’s disease. Gosuranemab was safe and well tolerated, but the clinical efficacy endpoint was not met.

## Main

Alzheimer’s disease (AD) is a progressive neurodegenerative disease with a high unmet need^1^. It is characterized by extracellular deposition of amyloid-β and neurofibrillary tangles composed of hyperphosphorylated tau protein^2–4^. Tau is a microtubule-associated protein integral to normal neuronal structure and function, and tau phosphorylation regulates the function of this protein within the cell^5–7^. In AD, hyperphosphorylation of tau (pathological tau) leads to its dissociation from microtubules and may contribute to the formation of neurotoxic tau aggregates^8–11^. The release of pathological tau from neurons is hypothesized to drive the seeding and spreading of tau pathology from cell to cell^10,12–14^. The spread of tau pathology is an important criterion for Braak staging and closely correlates with disease symptomatology^15^. Thus, therapies that reduce the accumulation of pathological tau species are hypothesized to delay AD progression.

Gosuranemab is a humanized immunoglobulin G4 monoclonal antibody to N-terminal tau^16–18^, and a study has shown that it binds to tau monomers and fibrils with high affinity^18^. In preclinical studies, gosuranemab robustly removed N-terminal tau from brain interstitial fluid, reducing tau aggregation in cells^18^. Therefore, gosuranemab has been hypothesized to potentially slow disease progression in tauopathies by preventing the uptake and neuronal transmission of the pathological tau responsible for neurodegeneration^18^.

The safety, efficacy, pharmacokinetics and pharmacodynamics of gosuranemab have been evaluated in previous clinical trials^16,17,19^. Two phase 1 studies (NCT02294851 and NCT02460094) found gosuranemab to be well tolerated and to demonstrate robust target engagement, reducing the levels of unbound N-terminal tau in the cerebrospinal fluid (CSF) of healthy volunteers and patients with progressive supranuclear palsy^16,17^. A phase 2 placebo-controlled study of gosuranemab in patients with progressive supranuclear palsy (PASSPORT, NCT03068468) reported similar safety profiles between the treatment and placebo groups; however, it did not demonstrate a benefit of gosuranemab in delaying disease progression^19^. A phase 1b basket trial of gosuranemab in patients with four primary tauopathies was initiated (TauBasket, NCT03658135) but terminated early owing to the lack of efficacy observed in the PASSPORT study^20^.

However, because AD is pathologically and clinically distinct from primary tauopathies^21^, we evaluated gosuranemab as an investigational agent in patients with AD in the TANGO study (NCT03352557). The primary objective of this study was to evaluate the safety and tolerability of gosuranemab in patients with mild cognitive impairment (MCI) due to AD and those with mild AD dementia. The study further tested the hypothesis that antibody engagement of extracellular N-terminal tau in the brain would slow AD progression.

## Results

### Participants

**Fig. 1:**
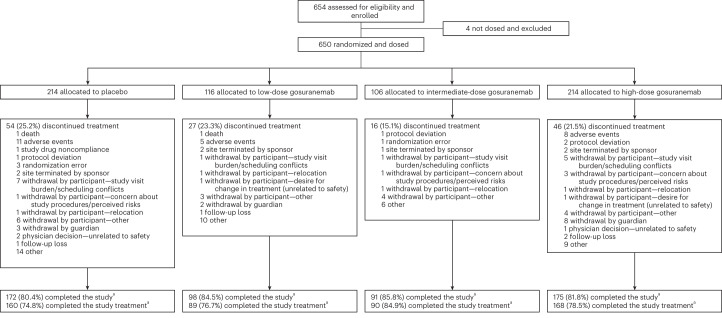
Participant disposition. ^a^Placebo-controlled period only. None of the participants completed the LTE period due to early study termination. Source data

A total of 654 participants were randomized (updated from the preplanned 528 participants due to overenrollment caused by fast recruitment) to one of four groups (650 participants were dosed): placebo (*n* = 214), low-dose gosuranemab (*n* = 58 in the 125 mg once every 4 weeks (q4w) subgroup and *n* = 58 in the 375 mg q12w subgroup, *n* = 116 total), intermediate-dose gosuranemab (600 mg q4w, *n* = 106) and high-dose gosuranemab (2,000 mg q4w, *n* = 214) (Fig. 1). Patient demographics at baseline were similar across treatment groups (Table 1). No apparent differences in baseline disease or biomarker characteristics were found across treatment groups (Table 2).

**Table 1:** Baseline demographics

Variable	Placebo (*n* = 214)	Low dose			600 mg q4w (*n* = 106)	2,000 mg q4w (*n* = 214)
		125 mg q4w (*n* = 58)	375 mg q12w (*n* = 58)	Total (*n* = 116)		
Age (years), mean ± s.d.	69.8 ± 6.6	70.4 ± 6.8	70.3 ± 6.8	70.4 ± 6.8	69.7 ± 6.7	69.4 ± 7.1
Female^a^, *n* (%)	106 (49.5)	28 (48.3)	26 (44.8)	54 (46.6)	55 (51.9)	112 (52.3)
Country, *n* (%)						
USA	117 (54.7)	31 (53.4)	30 (51.7)	61 (52.6)	56 (52.8)	119 (55.6)
Australia	6 (2.8)	2 (3.4)	2 (3.4)	4 (3.4)	2 (1.9)	7 (3.3)
Germany	18 (8.4)	4 (6.9)	9 (15.5)	13 (11.2)	10 (9.4)	21 (9.8)
Spain	20 (9.3)	8 (13.8)	4 (6.9)	12 (10.3)	12 (11.3)	13 (6.1)
France	19 (8.9)	5 (8.6)	5 (8.6)	10 (8.6)	7 (6.6)	17 (7.9)
Italy	9 (4.2)	1 (1.7)	3 (5.2)	4 (3.4)	5 (4.7)	9 (4.2)
Japan	3 (1.4)	3 (5.2)	2 (3.4)	5 (4.3)	6 (5.7)	5 (2.3)
Poland	14 (6.5)	3 (5.2)	3 (5.2)	6 (5.2)	7 (6.6)	14 (6.5)
Sweden	8 (3.7)	1 (1.7)	0	1 (0.9)	1 (0.9)	9 (4.2)
Race^b^, *n* (%)						
Asian	5 (2.3)	3 (5.2)	2 (3.4)	5 (4.3)	6 (5.7)	6 (2.8)
White	201 (93.9)	53 (91.4)	53 (91.4)	106 (91.4)	98 (92.5)	203 (94.9)
Education (years), mean ± s.d.	14.8 ± 3.7	14.4 ± 4.1	13.9 ± 3.2	14.2 ± 3.7	14.2 ± 3.7	14.3 ± 3.7
AD medication use, *n* (%)	139 (65.0)	38 (65.5)	37 (63.8)	75 (64.7)	69 (65.1)	137 (64.0)
ApoE ε4 status^c^, *n* (%)						
Carrier	157 (73.4)	35 (60.3)	43 (74.1)	78 (67.2)	66 (62.3)	160 (74.8)
Noncarrier	54 (25.2)	21 (36.2)	15 (25.9)	36 (31.0)	40 (37.7)	54 (25.2)
Clinical stage, *n* (%)						
MCI	98 (45.8)	25 (43.1)	31 (53.4)	56 (48.3)	51 (48.1)	98 (45.8)
Mild AD dementia	116 (54.2)	33 (56.9)	27 (46.6)	60 (51.7)	55 (51.9)	116 (54.2)

^a^Sex and/or gender was determined based on self-report.

^b^Ten participants did not provide ‘Race’ information due to confidentiality regulations, and two participants reported ‘Other’.

^c^Five participants reported ApoE ε4 status as ‘Undetermined’.

**Table 2:** Baseline clinical disease and biomarker characteristics

Variable	Placebo (*n* = 214)	Low dose			600 mg q4w (*n* = 106)	2,000 mg q4w (*n* = 214)
		125 mg q4w (*n* = 58)	375 mg q12w (*n* = 58)	Total (*n* = 116)		
MMSE score, mean ± s.d.	25.4 ± 2.3	25.4 ± 2.5	25.4 ± 2.22	25.4 ± 2.4	25.1 ± 2.3	25.4 ± 2.2
CDR global score, *n* (%)						
0.5	176 (82.2)	40 (69.0)	51 (87.9)	91 (78.4)	87 (82.1)	177 (82.7)
1	38 (17.8)	18 (31.0)	7 (12.1)	25 (21.6)	19 (17.9)	37 (17.3)
CDR-SB score, mean ± s.d.	3.1 ± 1.5	3.3 ± 1.7	2.6 ± 1.5	2.9 ± 1.6	3.2 ± 1.6	3.0 ± 1.4
ADAS-Cog13 score, mean ± s.d.	26.4 ± 8.4	25.6 ± 8.0	26.2 ± 8.7	25.9 ± 8.3	27.1 ± 8.8	25.3 ± 7.7
FAQ score, mean ± s.d.	8.1 ± 6.0	9.4 ± 7.0	7.4 ± 6.6	8.4 ± 6.8	9.9 ± 6.9	8.1 ± 6.4
ADCS-ADL score, mean ± s.d.	69.3 ± 6.1	68.7 ± 6.4	69.4 ± 7.3	69.1 ± 6.9	67.6 ± 8.0	69.5 ± 6.8
ISLT (*z* score), mean ± s.d.	−1.9 ± 0.9	−1.9 ± 0.9	−2.0 ± 1.0	−1.9 ± 0.9	−2.1 ± 1.0	−1.9 ± 1.0
ISLR (*z* score), mean ± s.d.	−2.4 ± 0.7	−2.2 ± 0.9	−2.4 ± 0.7	−2.3 ± 0.8	−2.3 ± 0.8	−2.3 ± 0.8
Tau PET^a,b,c^ SUVR, mean ± s.d.						
Braak I–II composite	1.934 ± 0.5934	1.843 ± 0.6571			1.917 ± 0.6196	1.937 ± 0.5342
Braak III–IV composite	1.890 ± 0.7223	1.918 ± 0.7770			1.888 ± 0.7062	1.891 ± 0.6801
Braak V–VI composite	1.742 ± 0.7881	1.754 ± 0.7604			1.741 ± 0.7714	1.766 ± 0.8555
Medial temporal cortex	2.154 ± 0.7990	2.194 ± 0.9274			2.183 ± 0.8140	2.194 ± 0.7840
Lateral temporal cortex	2.280 ± 1.0249	2.334 ± 1.1139			2.272 ± 1.0108	2.283 ± 0.9699
Frontal cortex	1.646 ± 0.7568	1.633 ± 0.7550			1.564 ± 0.7171	1.588 ± 0.7587
Amyloid PET^d,e^ SUVR, mean ± s.d.						
Amyloid-β composite	1.414 ± 0.182	1.417 ± 0.245			1.409 ± 0.170	1.454 ± 0.182

^a^In the tau PET sub-study, the placebo group included 118 participants, the low-dose group included 62 participants who received 125 mg (q4w) or 375 mg (q12w) gosuranemab, the 600 mg q4w group included 56 participants and the 2,000 mg q4w group included 121 participants.

^b^Tau PET tracer: [^18^F]MK-6240.

^c^Tau PET SUVR was computed for composite brain regions included in Braak staging^51,52^.

^d^In amyloid PET imaging, the placebo group included 105 participants, the low-dose group included 57 participants who received 125 mg (q4w) or 375 mg (q12w) gosuranemab, the 600 mg q4w group included 50 participants and the 2,000 mg q4w group included 105 participants.

^e^Amyloid-β tracer: [^18^F]florbetapir.

### Primary endpoint results for gosuranemab safety and tolerability

Safety results are presented in Table 3. Overall, the incidence of adverse events (AEs) and serious AEs (SAEs) was similar across the treatment and placebo groups. Likewise, the reported incidence of infusion-reaction AEs was similar for gosuranemab-treated (38.3%) and placebo-treated (36.9%) participants. The incidence of SAEs considered treatment-related (as determined by the investigator) was low for both gosuranemab-treated (0.5%) and placebo-treated (0.9%) participants. Rates of treatment discontinuation due to AEs were low overall and comparable between the placebo (5.1%) and combined gosuranemab (3.2%) groups.

**Table 3:** Primary safety endpoint for the placebo-controlled period

Variable	Placebo (*n* = 214)	125 mg q4w (*n* = 58)	375 mg q12w (*n* = 58)	600 mg q4w (*n* = 106)	2,000 mg q4w (*n* = 214)	Total gosuranemab (*n* = 436)
Any AE	181 (84.6)	50 (86.2)	48 (82.8)	94 (88.7)	189 (88.3)	381 (87.4)
Treatment-related^a^ AEs	47 (22.0)	15 (25.9)	12 (20.7)	21 (19.8)	50 (23.4)	98 (22.5)
SAEs	26 (12.1)	6 (10.3)	6 (10.3)	13 (12.3)	25 (11.7)	50 (11.5)
Treatment-related SAEs	2 (0.9)	0	0	0	2 (0.9)	2 (0.5)
Drug withdrawal due to AEs	11 (5.1)	4 (6.9)	2 (3.4)	0	8 (3.7)	14 (3.2)
Study withdrawal due to AEs	11 (5.1)	2 (3.4)	1 (1.7)	0	6 (2.8)	9 (2.1)
Mortality events	1 (0.5)	1 (1.7)	0	0	1 (0.5)	2 (0.5)
Infusion reactions	79 (36.9)	31 (53.4)	23 (39.7)	38 (35.8)	75 (35.0)	167 (38.3)
AEs with incidence of ≥10% in any single treatment group						
Falls	23 (10.7)	7 (12.1)	11 (19.0)	20 (18.9)	30 (14.0)	68 (15.6)
Nasopharyngitis	22 (10.3)	4 (6.9)	6 (10.3)	9 (8.5)	24 (11.2)	43 (9.9)
Arthralgia	14 (6.5)	6 (10.3)	7 (12.1)	9 (8.5)	19 (8.9)	41 (9.4)
Headache	20 (9.3)	1 (1.7)	6 (10.3)	11 (10.4)	22 (10.3)	40 (9.2)
Diarrhea	12 (5.6)	11 (19.0)	3 (5.2)	6 (5.7)	11 (5.1)	31 (7.1)
Constipation	8 (3.7)	6 (10.3)	1 (1.7)	2 (1.9)	6 (2.8)	15 (3.4)

Data are presented as *n* (%).

^a^Related as determined by the investigator.

The most common AEs (reported in at least 10% of participants in any single group) were falls, nasopharyngitis, arthralgia, headache, diarrhea and constipation (Table 3). AEs with at least 2% higher incidence in the combined gosuranemab-treated groups than in the placebo group were falls (10.7% in the placebo group, 15.6% in the combined gosuranemab groups) and arthralgia (6.5% in the placebo group, 9.4% in the combined gosuranemab groups). The most common categories of AEs leading to discontinuation were nervous system disorders (for example, cerebral hemorrhage, with an incidence of 1.4% in the placebo group and 1.1% in the combined gosuranemab groups) and neoplasms (2.3% in the placebo group, 0.7% in the gosuranemab groups); the only AE leading to discontinuation that was reported in more than one participant was seizure (one case in the placebo group, one case in the high-dose gosuranemab group). During the placebo-controlled period, one death occurred in the placebo group (0.5%) and two deaths occurred across all gosuranemab groups (0.5%). One death (in the high-dose group) was considered treatment-related (subdural hematoma related to a possible fall); the other two deaths were not considered related to treatment (COVID-19 pneumonia in the placebo group, metastatic pancreatic carcinoma in the low-dose group).

The most notable change in magnetic resonance imaging (MRI) scans compared to baseline was the appearance of new microhemorrhages. The incidence of cerebral microhemorrhages was lower in the gosuranemab-treated groups (5.0%) than in the placebo group (11.4%).

### Secondary endpoint results for cognitive and functional measurements (CDR-SB)

**Fig. 2:**
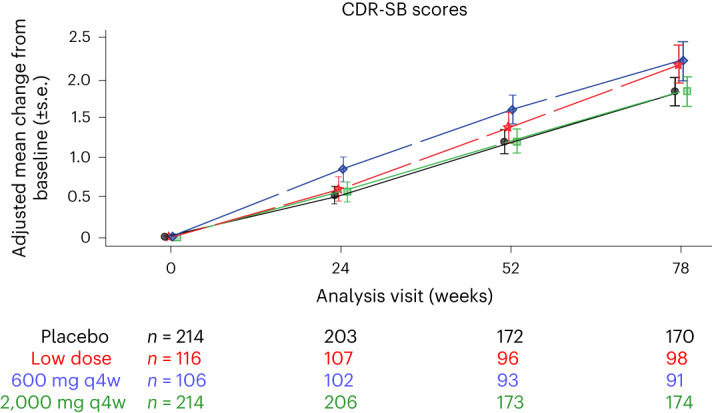
Longitudinal changes in a secondary efficacy outcome: CDR-SB scores from baseline to week 78. The graph shows the adjusted mean changes in CDR-SB scores from baseline (±standard error (s.e.)) up to week 78. A greater positive change indicates a worsening of symptoms. Sample sizes for each group at each time point are listed. Analyses were two-sided at a 5% significance level. No adjustments were made for multiple comparisons. Source data

No significant difference in the CDR-SB score (a secondary endpoint) was observed between the gosuranemab groups and the placebo group. The difference between the treated groups and the placebo group at week 78 was –0.01 (*P* = 0.9778) for the high-dose group, 0.38 (*P* = 0.1965) for the intermediate-dose group and 0.34 (*P* = 0.2362) for the low-dose group. The adjusted mean change from baseline at week 78 was 1.85 (95% confidence interval (CI) 1.48, 2.21) in the high-dose group, 2.24 (95% CI 1.75, 2.72) in the intermediate-dose group, 2.20 (95% CI 1.72, 2.67) in the low-dose group and 1.85 (95% CI 1.48, 2.22) in the placebo group; no dose–response was observed (Fig. 2).

### Secondary endpoint results for immunogenicity

The incidence of treatment-emergent anti-gosuranemab antibody responses was low at all time points and similar between all gosuranemab dose groups and the placebo group. One participant in the intermediate-dose group (1.0%) and four participants in the placebo group (1.9%) had a positive treatment-emergent anti-gosuranemab antibody response at any time point after baseline and within the week 76 visit. Persistent responses were observed in two participants in the placebo group (0.9%); no participants in the gosuranemab-treated groups showed a persistent response. Transient responses were observed in two participants in the placebo group (0.9%) and one participant in the intermediate-dose group (1.0%).

### Exploratory endpoints: other key efficacy outcomes (cognitive and functional analyses)

**Extended Data Fig. 1:**
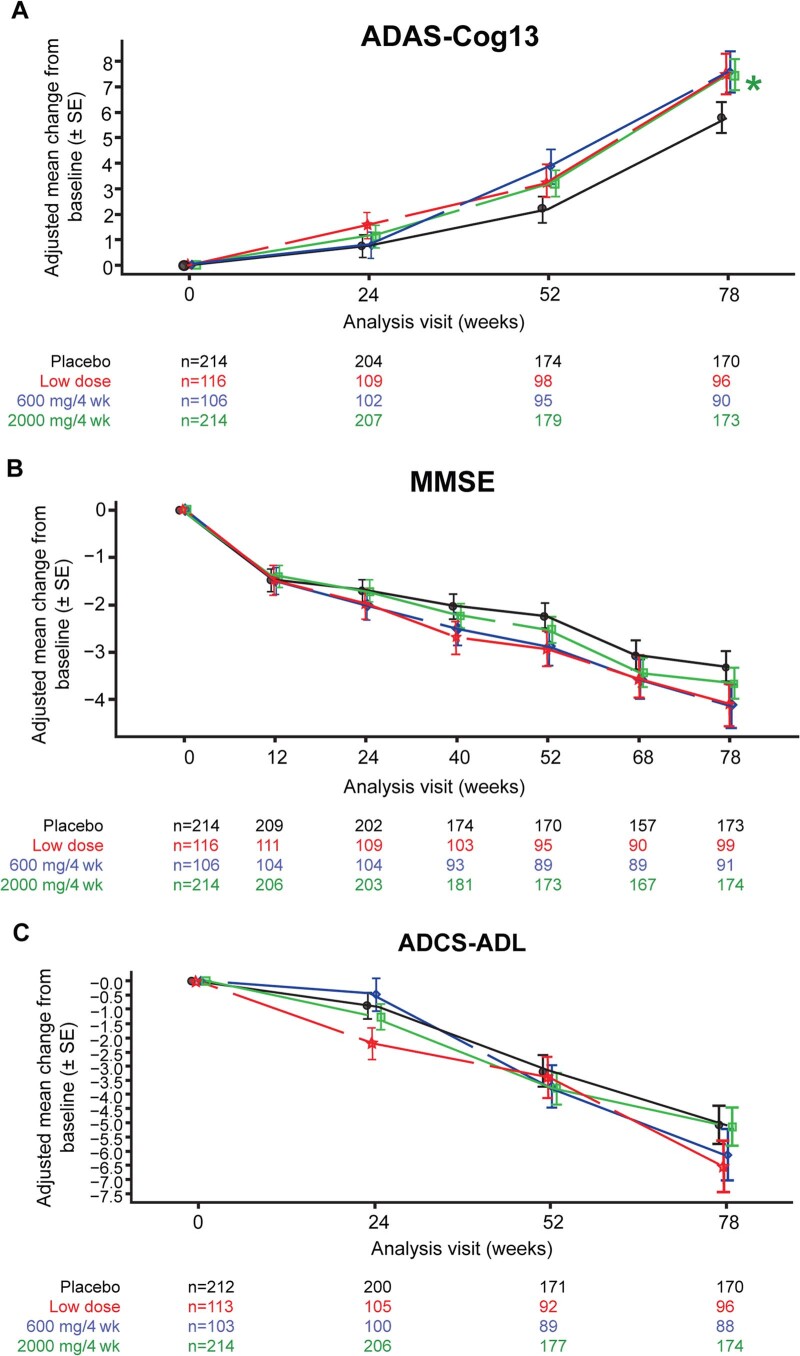
Longitudinal changes on secondary efficacy assessments from baseline to week 78. Adjusted mean change from baseline (±SE) up to week 78 on the (**a**) ADAS-Cog13, (**b**) MMSE, and (**c**) ADCS-ADL scores. In panel A, a greater positive change indicates worsening of symptoms; in panels B and C, a greater negative change indicates worsening. Sample sizes for each group at each time point are listed for each panel. Asterisk denotes a significant difference between the high-dose placebo groups (*P* = 0.0378). Analyses were two-sided at 5% significance level. No adjustments were made for multiple comparisons. Source data

**Extended Data Fig. 2:**
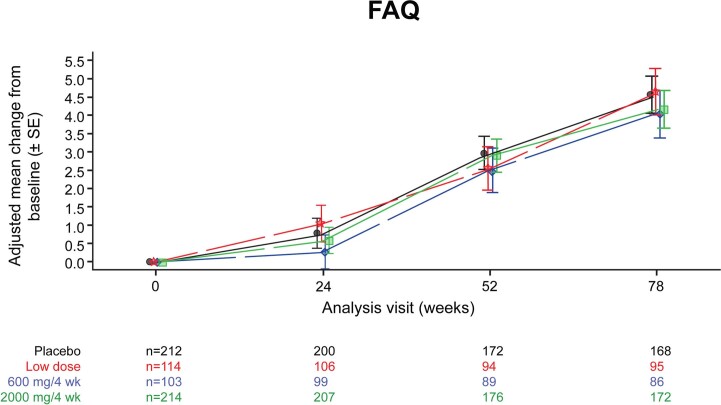
Longitudinal changes in the FAQ score from baseline to week 78. Adjusted mean change from baseline (±SE) up to week 78 on FAQ score. A greater positive change indicates worsening of symptoms. Sample sizes for each group at each time point are listed for each panel. Analyses were two-sided at 5% significance level. No adjustments were made for multiple comparisons. Source data

In one exploratory endpoint, the change from baseline in the 13-item Alzheimer’s Disease Assessment Scale–Cognitive Subscale (ADAS-Cog13) score at week 78, patients in the high-dose gosuranemab group performed statistically significantly worse than those in the placebo group (Extended Data Fig. 1a; difference = 1.69, *P* = 0.0378); however, no dose–response was observed for this measure (difference = 1.79, *P* = 0.0681 for the intermediate-dose group; difference = 1.73, *P* = 0.0719 for the low-dose group). Moreover, at week 104 during the long-term extension (LTE) period, this difference (1.80) was not significant (*P* = 0.0907) (see the ‘Post hoc analysis of the aborted LTE period’ subsection). No other significant differences were observed between the gosuranemab groups and the placebo group in any other exploratory endpoints, such as Mini-Mental State Examination (MMSE) scores (difference = –0.35, *P* = 0.0446 for the high-dose group; difference = –0.81, *P* = 0.1415 for the intermediate-dose group; difference = −0.79, *P* = 0.1415 for the low-dose group), AD Cooperative Study–Activities of Daily Living (ADCS-ADL) scores (difference = –0.06, *P* = 0.9429 for the high-dose group; difference = –1.06, *P* = 0.3336 for the intermediate-dose group; difference = –1.47, *P* = 0.1685 for the low-dose group) and Functional Activities Questionnaire (FAQ) scores (difference = –0.4, *P* = 0.5320 for the high-dose group; difference = –0.52, *P* = 0.5100 for the intermediate-dose group; difference = 0.08, *P* = 0.9139 for the low-dose group). For the MMSE score, the adjusted mean change from baseline at week 78 was –3.66 (95% CI –4.34, –2.99) in the high-dose group, –4.13 (95% CI –5.03, –3.22) in the intermediate-dose group, –4.11 (95% CI –4.98, –3.23) in the low-dose group and −3.32 (95% CI –4.00, −2.64) in the placebo group (Extended Data Fig. 1b). For the ADCS-ADL score, the adjusted mean change from baseline at week 78 was –5.14 (95% CI –6.47, –3.81) in the high-dose group, –6.13 (95% CI –7.93, –4.34) in the intermediate-dose group, –6.54 (95% CI –8.28, –4.81) in the low-dose group and –5.08 (95% CI –6.42, –3.73) in the placebo group (Extended Data Fig. 1c). For the FAQ score, the adjusted mean change from baseline at week 78 was 4.16 (95% CI 3.17, 5.14) in the high-dose group, 4.04 (95% CI 2.72, 5.35) in the intermediate-dose group, 4.64 (95% CI 3.37, 5.91) in the low-dose group and 4.56 (95% CI 3.56, 5.55) in the placebo group (Extended Data Fig. 2).

### Exploratory endpoints: CSF biomarkers

**Fig. 3:**
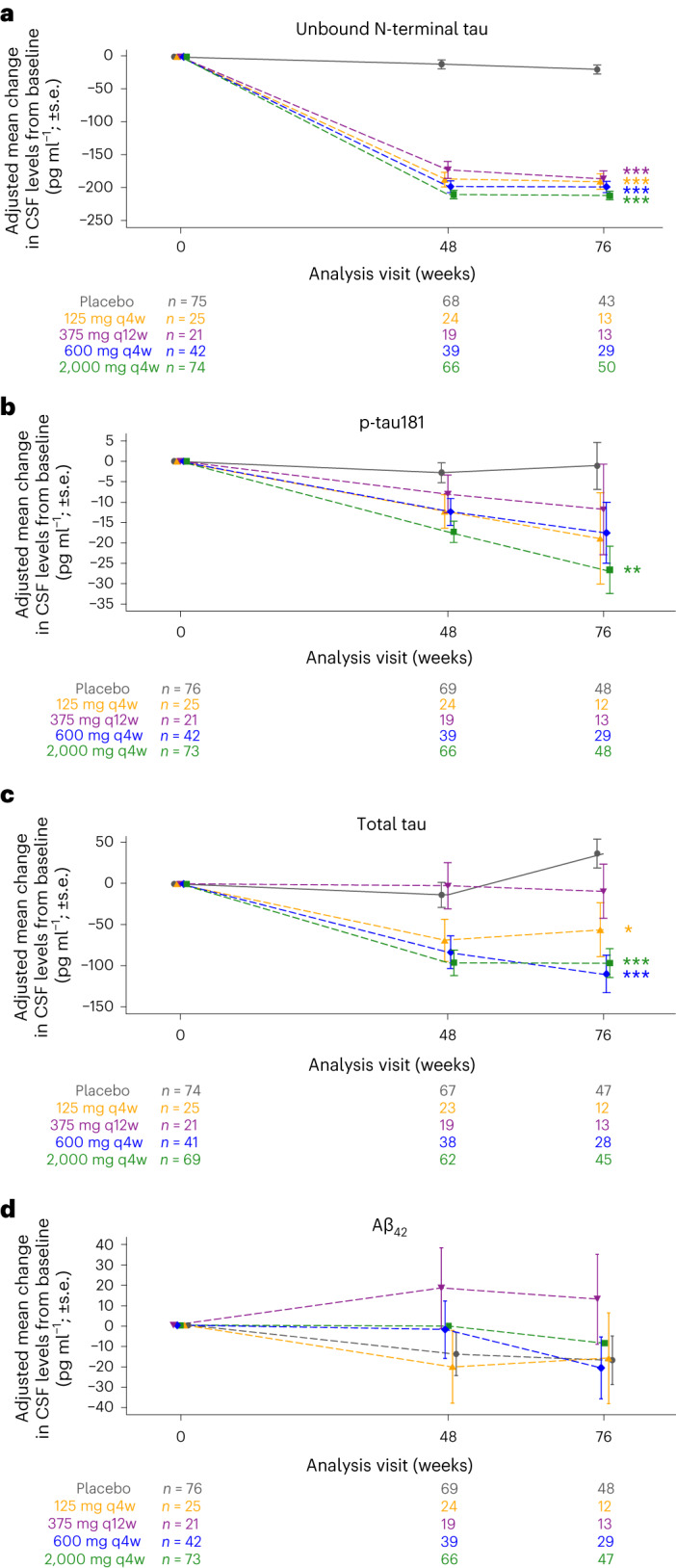
Target engagement and pharmacodynamics. **a**, Target engagement as measured by the adjusted mean change from baseline (±s.e.) in the CSF levels of unbound N-terminal tau. **b**, Pharmacodynamics as measured by the adjusted mean change from baseline (±s.e.) in the CSF levels of p-tau181. **c**, Pharmacodynamics as measured by the adjusted mean change from baseline (±s.e.) in the CSF levels of total tau. **d**, Pharmacodynamics as measured by the adjusted mean change from baseline (±s.e.) in the CSF levels of Aβ_42_. Sample sizes for each group at each time point are listed for each panel. In **a**, the asterisks denote a significant difference from placebo for the group of the same color (***P < 0.0001). In **b**, the asterisks denote a statistically significant difference between the high-dose and placebo groups at week 76 (**P = 0.0022). In **c**, the asterisks denote a statistically significant difference between the 125 mg q4w low-dose group and the placebo group (*P = 0.0138) and between the intermediate-dose and high-dose groups at week 76 (***P < 0.0001). Analyses were two-sided at a 5% significance level. No adjustments were made for multiple comparisons. Source data

A robust decrease in the CSF levels of unbound N-terminal tau compared to baseline was observed in all gosuranemab groups but not in the placebo group, confirming target engagement of gosuranemab (Fig. 3a). At week 76, decreases in unbound N-terminal tau levels in the CSF were statistically significant in all treatment groups relative to the placebo group (*P* < 0.0001). At week 76, the adjusted mean change from baseline (percentage from baseline, calculated as 100 × adjusted mean change from baseline/mean baseline value) in the CSF levels of unbound N-terminal tau was −212.83 pg ml^−1^ (−92.6%) in the high-dose group, −199.45 pg ml^−1^ (−86.0%) in the intermediate-dose group, −187.15 pg ml^−1^ (−80.1%) in the 375 mg q12w low-dose group, −191.38 pg ml^−1^ (−82.1%) in the 125 mg q4w low-dose group and −19.39 pg ml^−1^ (−8.8%) in the placebo group.

Treatment with gosuranemab was generally associated with reductions in other tau CSF biomarkers. For the CSF levels of phosphorylated tau181 (p-tau181), the adjusted mean change (percentage) from baseline at week 76 was −26.52 pg ml^−1^ (−23.8%) in the high-dose group, −17.44 pg ml^−1^ (−15.9%) in the intermediate-dose group, −11.72 pg ml^−1^ (−10.6%) in the 375 mg q12w low-dose group, −18.84 pg ml^−1^ (−17.6%) in the 125 mg q4w low-dose group and −1.05 pg ml^−1^ (−1.1%) in the placebo group; only the high-dose group showed a significant difference from placebo (*P* = 0.0022; Fig. 3b). For the CSF levels of total tau, the adjusted mean change (percentage) from baseline at week 76 was −97.46 pg ml^−1^ (−15.5%) in the high-dose group, −110.57 pg ml^−1^ (−15.5%) in the intermediate-dose group, −9.41 pg ml^−1^ (−1.3%) in the 375 mg q12w low-dose group, −56.14 pg ml^−1^ (−8.0%) in the 125 mg q4w low-dose group and 37.21 pg ml^−1^ (6.2%) in the placebo group; a significant difference from placebo was observed in the 125 mg q4w low-dose group (*P* = 0.0138) and the intermediate-dose and high-dose groups (*P* < 0.0001 for both; Fig. 3c). No dose–response for either CSF measure of tau was observed among treatment groups.

Levels of the amyloid-β isoform Aβ_42_ in the CSF were also measured, and the changes from baseline at week 76 were small and comparable between groups (Fig. 3d). The adjusted mean change (percentage) from baseline in the CSF levels of Aβ_42_ was −8.99 pg ml^−1^ (−1.90%) in the high-dose group, −21.07 pg ml^−1^ (−4.11%) in the intermediate-dose group, 12.66 pg ml^−1^ (2.50%) in the 375 mg q12w low-dose group, −16.24 pg ml^−1^ (−3.35%) in the 125 mg q4w low-dose group and −17.38 pg ml^−1^ (−3.81%) in the placebo group.

### Exploratory endpoint: tau PET neuroimaging

**Fig. 4:**
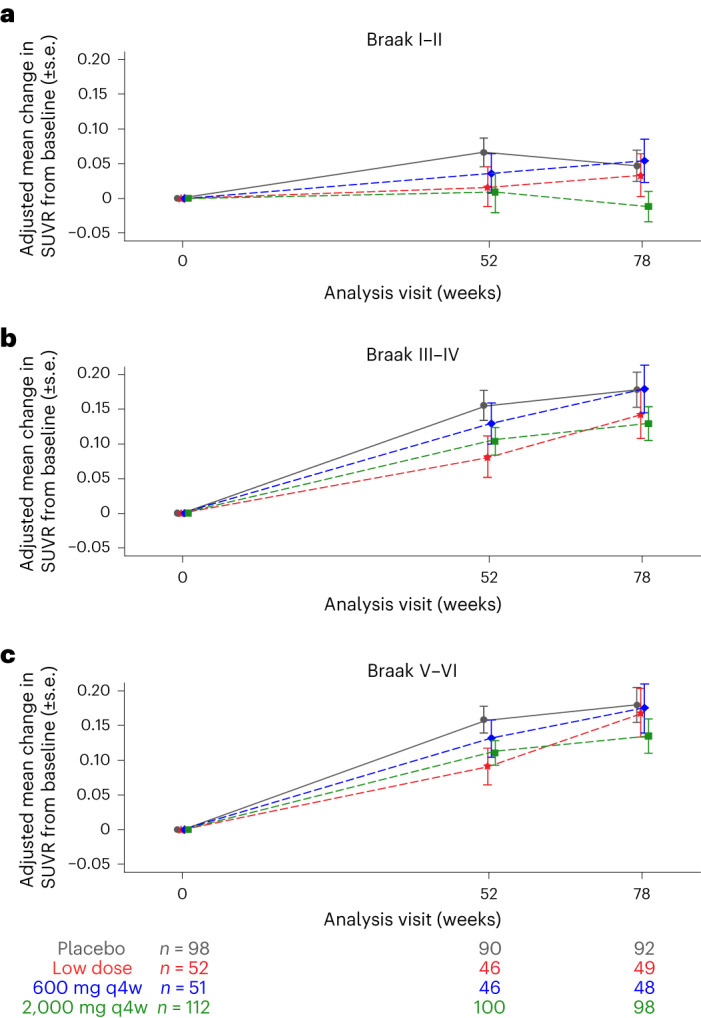
Adjusted mean change in tau PET SUVR from baseline to week 78. **a**–**c**, Adjusted mean change from baseline (±s.e.) in the tau PET SUVR in composite regions corresponding to Braak stages I–II (**a**), III–IV (**b**) and V–VI (**c**). Sample sizes are provided for each group at each time point and are the same for all panels. Analyses were two-sided at a 5% significance level. No adjustments were made for multiple comparisons. Source data

**Extended Data Fig. 3:**
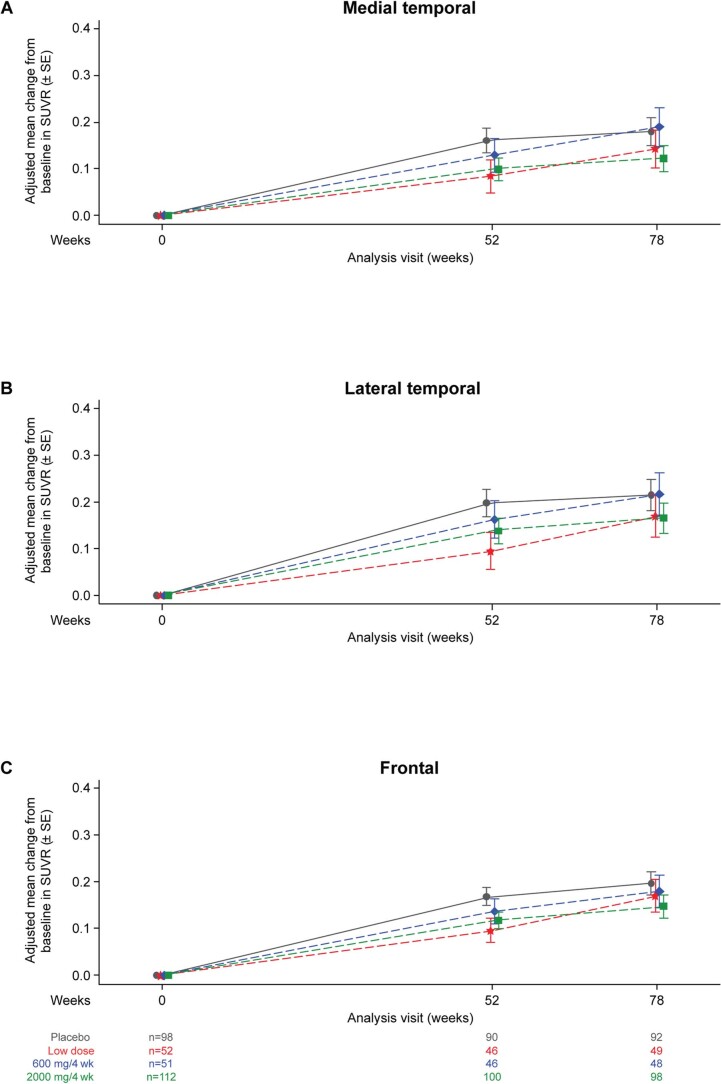
Adjusted mean change in tau PET SUVR from baseline to week 78. Adjusted mean change from baseline (±SE) in tau PET SUVR in brain regions corresponding to (**a**) medial temporal cortex, (**b**) lateral temporal cortex, and (**c**) frontal cortex. Sample sizes are provided for each group at each time point and are the same for all panels. Analyses were two-sided at 5% significance level. No adjustments were made for multiple comparisons. Source data

No significant differences were observed between the gosuranemab and placebo groups (*P* > 0.05) in the adjusted mean change in the tau positron emission tomography (PET) standardized uptake value ratio (SUVR) in each brain composite region corresponding to Braak stages I–II (–0.012 (95% CI −0.055, 0.031) in the high-dose group, 0.054 (95% CI −0.008, 0.115) in the intermediate-dose group, 0.033 (95% CI −0.028, 0.094) in the low-dose group and 0.047 (95% CI 0.002, 0.091) in the placebo group), Braak stages III–IV (0.129 (95% CI 0.082, 0.176) in the high-dose group, 0.178 (95% CI 0.110, 0.246) in the intermediate-dose group, 0.142 (95% CI 0.075, 0.209) in the low-dose group and 0.177 (95% CI 0.128, 0.227) in the placebo group) and Braak stages V–VI (0.135 (95% CI 0.087, 0.183) in the high-dose group, 0.175 (95% CI 0.106, 0.244) in the intermediate-dose group, 0.168 (95% CI 0.100, 0.237) in the low-dose group and 0.180 (95% CI 0.130, 0.230) in the placebo group; Fig. 4a–c). As expected, the placebo group demonstrated increased tau PET SUVR over 78 weeks. Similar results were obtained for the medial temporal, lateral temporal and frontal cortices (Extended Data Fig. 3). No dose–responses were observed for changes in the tau PET SUVR.

**Extended Data Fig. 4:**
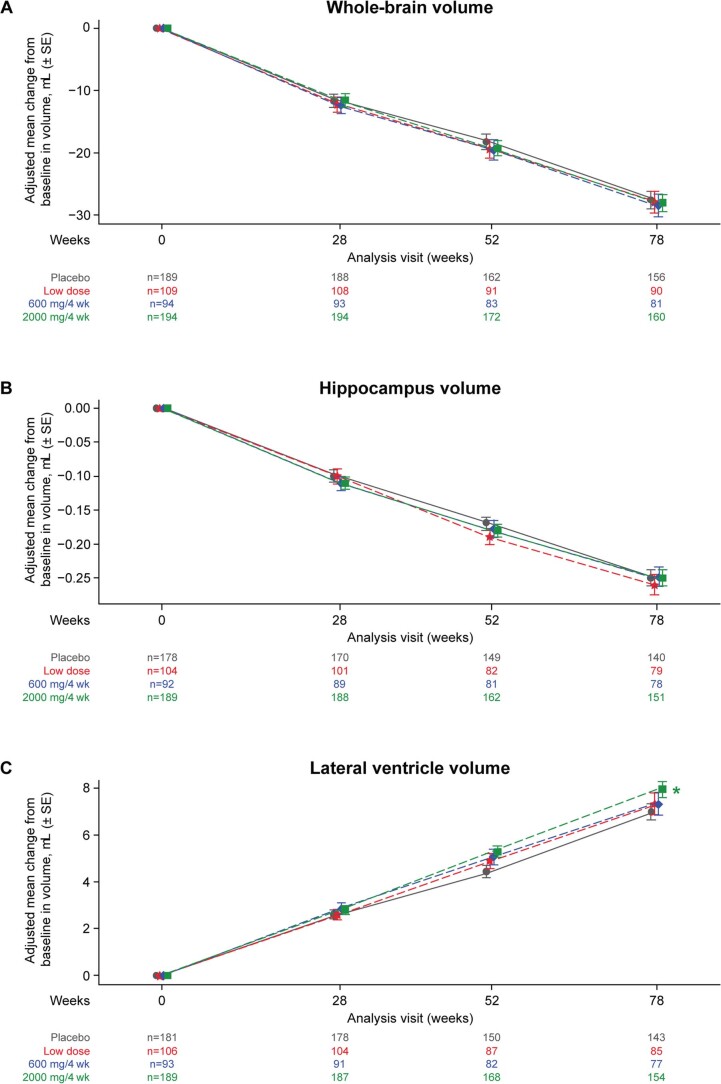
Brain volume changes as measured by structural MRI from baseline to week 78. Adjusted mean change from baseline (±SE) in (**a**) whole brain volume, (**b**) hippocampus volume, and (**c**) lateral ventricle volume. Sample sizes for each group at each time point are listed for each panel. In panel C, the asterisk denotes a statistically significant difference in lateral ventricle volume between the high-dose and placebo groups at week 78 (*P* = 0.0481). Analyses were two-sided at 5% significance level. No adjustments were made for multiple comparisons. Source data

Whole-brain and hippocampal volumes were measured across the treatment and placebo groups. Decreases were observed in all groups and were small and comparable between groups (Extended Data Fig. 4). The lateral ventricle volume increased in all groups, with a <1-cm^3^ statistically significant (*P* = 0.0481) increase observed in the high-dose group relative to the placebo group at week 78 (Extended Data Fig. 4c).

### Post hoc analysis of the aborted LTE period

The LTE period of the study was terminated early owing to the lack of efficacy demonstrated upon readout after the placebo-controlled period. Data from the LTE period were analyzed up to week 104, during which the sample size was still substantial (the *n* for each group was >50% of the total sample size at baseline). The safety profiles in this period were similar to those during the placebo-controlled period. The late-start treatment group (late-start high-dose group) was included in the study to evaluate the safety profile of gosuranemab in participants in whom treatment was initiated later in their disease course (these participants have potentially more advanced disease), allowing for analyses supporting the disease-modifying effects of gosuranemab (delayed-start analysis). No differences in CDR-SB scores or other exploratory efficacy endpoints were observed in the LTE period between the early-start treatment groups (participants who received high-dose, intermediate-dose or low-dose gosuranemab during the placebo-controlled and LTE periods) and the late-start high-dose group (participants who initially received placebo and were switched to high-dose gosuranemab during the LTE period) (*P* > 0.05). At week 104, the adjusted mean change in CDR-SB scores from baseline was 2.92 (95% CI 2.34, 3.49) in the low-dose group, 3.04 (95% CI 2.45, 3.62) in the intermediate-dose group, 2.35 (95% CI 1.92, 2.79) in the high-dose group and 2.55 (95% CI 2.10, 2.99) in the late-start high-dose group. For MMSE scores, the adjusted mean change from baseline at week 104 was –5.49 (95% CI −6.52, −4.46) in the low-dose group, –5.51 (95% CI −6.57, −4.44) in the intermediate-dose group, –4.82 (95% CI −5.61, −4.03) in the high-dose group and –4.39 (95% CI −5.18, −3.59) in the late-start high-dose group. For ADCS-ADL scores, the adjusted mean change from baseline at week 104 was –9.59 (95% CI −11.92, −7.26) in the low-dose group, –8.33 (95% CI −10.74, −5.91) in the intermediate-dose group, –8.13 (95% CI −9.89, −6.38) in the high-dose group and –7.91 (95% CI −9.70, −6.12) in the late-start high-dose group. In contrast to week 78, none of the other treatment groups showed a statistically significant difference in the change from the baseline ADAS-Cog13 score at week 104 compared to the late-start high-dose (formerly placebo) group (low-dose group: *n* = 75, adjusted mean change from baseline = 9.55, difference = 1.57, *P* = 0.2105; intermediate-dose group: *n* = 72, adjusted mean change from baseline = 8.78, difference = 0.8, *P* = 0.5296; high-dose group: *n* = 132, adjusted mean change from baseline = 9.78, difference = 1.80, *P* = 0.0907; late-start high-dose group: *n* = 126, adjusted mean change from baseline = 7.98; Supplementary Table 1).

Long-term exposure to gosuranemab was well tolerated by the participants. The safety profiles during the LTE period were similar to those during the placebo-controlled period, and no additional safety concerns were observed with the limited exposure to gosuranemab in the LTE period. The overall incidence of AEs and SAEs in the LTE period was similar between the early-start (*n* = 168) and late-start (*n* = 165) high-dose groups (AEs: 61.3% in the early-start group, 60.0% in the late-start group; SAEs: 6.0% in the early-start group, 7.9% in the late-start group). One SAE in the late-start high-dose group (colon cancer) was considered by the investigator to be treatment-related. In the LTE period, five participants experienced AEs leading to treatment discontinuation; no AEs leading to discontinuation were reported in more than one participant. Six participants died during the LTE period, with one participant (from the high-dose group) dying before receiving any dose in this period; no deaths during the LTE period were considered treatment-related. The most common AEs in the LTE period were similar in profile and incidence to those in the placebo-controlled period.

### Effect of COVID-19

Despite the COVID-19 pandemic, most participants received 16–20 of 20 infusions during the placebo-controlled period (*n* = 538, 82.8%); only 64 participants (9.8%) missed three or more consecutive infusions. A relatively small number of participants took advantage of the provided options for COVID-19 risk mitigation: 19 participants (2.9%) remotely completed 37 scales, and 36 home infusions occurred. Overall, 32 participants (4.9%) discontinued the study treatment during the placebo-controlled period due to reasons related to COVID-19. During the placebo-controlled period, COVID-19 AEs were reported in three participants in the gosuranemab groups and in two participants in the placebo group, and the rates of major protocol deviations related to COVID-19 were balanced between groups.

## Discussion

The TANGO study evaluated the safety and efficacy of gosuranemab in patients with AD. The participants tolerated gosuranemab well at all doses evaluated, and the safety outcomes were consistent with those reported in previous studies. However, no dose produced a favorable separation from placebo on a secondary endpoint: the change in the CDR-SB score from baseline at 78 weeks. Furthermore, none of the treatment groups exhibited an improvement over the placebo group in any of the exploratory efficacy endpoints. In one such assessment (ADAS-Cog13 scale), the high-dose group performed statistically significantly worse than the placebo group at week 78; however, this difference was not statistically significant in the LTE period. The lack of clinical efficacy observed in TANGO is consistent with the results of recent clinical trials investigating the N-terminal anti-tau antibodies semorinemab and tilavonemab in early AD^22–24^. By contrast, in the Lauriet trial, semorinemab demonstrated partial efficacy compared to placebo, with a reduced rate of cognitive decline based on one coprimary endpoint (ADAS-Cog11 scores) in participants with moderate AD^24^.

Treatment with gosuranemab was associated with a robust reduction in the CSF levels of unbound N-terminal tau, confirming the target engagement of this antibody. In the treatment groups, the CSF levels of p-tau181 and total tau were lower at week 76 than at baseline. However, differences in p-tau181 and total tau levels among the treatment and placebo groups were significant only for some dose groups, and no dose–response was observed for either measure. Analysis of the tau PET substudy—the largest [^18^F]MK-6240 tau PET dataset collected to date in the context of a well-controlled clinical trial—demonstrated measurable longitudinal increases in tau PET SUVR over time, as expected, yet no effect of treatment on cerebral tau accumulation in the target brain regions (for example, composite regions corresponding to Braak stages I–VI). Thus, gosuranemab effectively bound extracellular N-terminal tau, but this binding did not reduce the accumulation of pathological tau as detected by tau PET. Improved fluid measures need to be developed to better understand the extracellular availability of pathological tau for future antibody-based therapeutic approaches. Additionally, the ongoing development of assays specific for synaptic and inflammatory-related markers may provide a future potential link to these emerging areas of AD pathophysiology and tau biology.

Preclinical models of AD to date leave room for improved clinical translation. In several preclinical tau transgenic mouse models, the anti-tau antibodies tilavonemab, zagotenemab and semorinemab, which target N-terminal or conformational epitopes of tau, have demonstrated efficacy in reducing tau pathology and, in some cases, providing functional or behavioral improvements^25–28^. However, similar to gosuranemab, these agents have failed to demonstrate a clear clinical benefit in patients with early AD, calling into question the predictive value of these mouse studies for clinical efficacy. The tau transgenic mouse models used in these preclinical studies typically express the human 4R tau isoform with the frontotemporal-dementia-associated P301L or P301S substitution, accelerating the formation of paired helical filaments^29^. This results in robust age-dependent formation of intracellular tau aggregates in the brain. However, owing to the use of exogenous promoters to drive transgene expression, tau pathology does not occur in brain regions typically affected in AD or progress in a neuronal-network-dependent pattern, as would be expected if tau pathology spread were solely due to transneuronal transmission, in these models. Thus, these models are unsuitable for testing therapeutics aimed at intercepting extracellular tau to prevent the transneuronal spread of tau pathology. To circumvent this problem, researchers have developed tau-seeding models in tau transgenic or wild-type mice. In these mice, exogenous tau ‘seeds’ in the form of recombinant fibrils or tau-enriched brain fractions derived from tau transgenic mouse or AD brain are locally injected into a specific brain region, and the progressive spread of tau pathology into anatomically connected brain regions is observed^30–37^. One major limitation of this approach is the nature of brain-derived tau seeds, which are mainly derived from intracellular tau aggregates. It remains unclear whether these aggregated forms of tau exist in human interstitial fluid or CSF. A few anti-tau monoclonal antibodies targeting various epitopes and post-translational modifications have been shown to reduce tau pathology propagation in these tau-spreading models; however, the translatability of these findings to clinical efficacy remains to be determined^38–40^.

All groups exhibited comparable reductions in hippocampal and total brain volumes, consistent with disease progression. A statistically significant increase in the lateral ventricular volume, with unclear clinical significance, was observed in the high-dose treatment group relative to the placebo group.

The pharmacodynamic data from the TANGO study are consistent with those from previous trials investigating gosuranemab. In a single-ascending-dose trial, gosuranemab doses of 70–4,200 mg decreased the CSF levels of unbound N-terminal tau by 67–97% at 4 weeks^16^. In a multiple-ascending-dose trial, gosuranemab doses of 150–2,100 mg administered q4w decreased the CSF levels of unbound N-terminal tau by 90–96% at 4 weeks and by 91–97% at 12 weeks^17^. The magnitude, timing and duration of target engagement observed in the TANGO study are consistent with those observed in these trials.

In the TANGO study, gosuranemab was tested based on the hypothesis that extracellular seeding-competent tau species propagate tau pathology throughout the brain. This study is supported by preclinical data demonstrating the high binding affinity of gosuranemab to monomeric and aggregated forms of tau and its ability to remove seeding-competent forms of tau from AD brain lysate and interstitial fluid derived from tau transgenic mice^18^. Although the exact nature of seeding-competent tau species remains elusive, it is evident that the microtubule-binding region of tau is required for tau fibrillization and seeding activity^41^. Tau fragments in the CSF that span the microtubule-binding region are of low abundance (0.4–3.7 ng ml^−1^, depending on the residue examined), whereas N-terminal and midregion fragments are relatively abundant (8.2–32.0 ng ml^−1^ for midregion fragments)^42^. Thus, targeting an N-terminal tau epitope may not sufficiently capture extracellular tau species responsible for mediating tau pathology propagation. Alternatively, it is conceivable that pathological tau species spread between neurons through pathways not accessible to monoclonal antibodies, such as exosomes or nanotubes^43–45^. Furthermore, most tau protein resides inside neurons, whereas extracellular tau represents only a fraction of all tau forms expressed in the brain. Thus, intracellular pathological tau species might contribute more to overall tau toxicity, and targeting intracellular tau may provide greater therapeutic benefits.

In addition to the biological and technical limitations discussed above, operational limitations should be considered when interpreting the study results. This study was affected by the COVID-19 pandemic. Many efforts were made to minimize the effect of the pandemic on the study participants. As a measure against study withdrawals, participants were allowed flexibility in scheduling their site visits. This flexibility made possible the low discontinuation rate observed in the study, at the expense of increased protocol deviations. However, these deviations were generally balanced across treatment groups, mitigating the potential effect of this limitation on the interpretation of the results. Limited data were collected during the LTE period due to the early termination of the study.

## Methods

The full trial protocol and statistical analysis plan can be downloaded at https://classic.clinicaltrials.gov/ct2/show/NCT03352557 (classic ClinicalTrials.gov).

### Study overview

This randomized, parallel-group study consisted of a 78-week double-blind, placebo-controlled phase and a subsequent dose-blind LTE phase. Enrolled participants were randomized (1:1:2:2) to one of four treatment arms: (1) low-dose gosuranemab (participants in this group were subsequently randomized 1:1 to receive either 125 mg gosuranemab q4w or 375 mg gosuranemab q12w), (2) intermediate-dose gosuranemab (600 mg q4w), (3) high-dose gosuranemab (2,000 mg q4w) or (4) placebo (0.9% NaCl q4w). Randomization was conducted by interactive response technology (IRT); the IRT vendor generated the randomization sequence. Randomization was stratified by region, disease stage (MCI or mild AD), baseline AD medication use and tau PET and/or CSF substudy enrollment (see the ‘Biomarker substudies’ subsection). Treatments were administered intravenously q4w; participants assigned to the low-dose arm who received infusions of 375 mg gosuranemab q12w received placebo at the other 4-week dosing visits to maintain the treatment blind. During the double-blind, placebo-controlled period, all participants and the study staff who performed participant assessments were blinded to the treatment assignments. During the dose-blind LTE period, participants in the placebo group were reassigned to receive high-dose treatment; participants in other groups continued receiving their originally assigned doses. No interim analysis was conducted for TANGO during the placebo-controlled period. At the end of the placebo-controlled period, a prespecified unblinded analysis was performed. Study visits occurred between May 2018 and August 2021. TANGO participants received reimbursement for travel or meals when allowed within local regulations and approved by the Institutional Review Board (IRB).

### Eligibility criteria

Participants were adults aged 50–80 years who had exhibited a progressive decline in memory function for >6 months before screening and had been diagnosed with either MCI due to AD or mild AD dementia according to National Institute on Aging–Alzheimer’s Association criteria^46,47^. Participants must have demonstrated cognitive impairment at the time of screening, defined by an International Shopping List Test—Immediate Recall (ISLT) or International Shopping List Test–Delayed Recall (ISLR) score of 1 s.d. below the age-adjusted normative mean, a CDR global score of 0.5 (for MCI due to AD) or 0.5 or 1.0 (for mild AD dementia), a CDR Memory Box score of at least 0.5 and an MMSE score between 22 and 30 (inclusive). Participants must also have demonstrated evidence of amyloid pathology, confirmed by amyloid PET (visual read) or CSF testing. Finally, consent to apolipoprotein E (ApoE) genotyping was required for participation, as was the presence of a suitable care partner or informant to monitor the participant’s cognitive and functional abilities. The exclusion criteria were any medical or neurological/neurodegenerative conditions that might contribute to the participant’s cognitive impairment; a history of seizures within 10 years before screening visit 1 or epileptic syndrome; a history of a severe brain infection within 5 years before screening visit 1 or severe head trauma; a history of unstable angina, myocardial infarction, chronic heart failure or clinically relevant conduction abnormalities within 1 year before screening visit 1; evidence of impaired renal or liver function; alcohol or substance abuse in the past year; presence of clinically relevant and/or unstable psychiatric illness within 6 months before screening visit 1; known allergy to gosuranemab or a history of hypersensitivity to any of its inactive ingredients; use of AD medications at doses that had not been stable for at least 8 weeks before screening visit 1; and use of any medication that might affect the participant’s cognition.

This study was conducted in accordance with the Declaration of Helsinki and all applicable International Council for Harmonisation and Good Clinical Practice guidelines. Investigators were required to obtain ethics committee approval before beginning the study. For study sites in the USA, the study protocol was approved by Advarra’s central IRB or one of the following local ethics committees: BioMed IRB, San Diego, CA; Biomedical Research Alliance of New York, Lake Success, NY; Western IRB, Puyallup, WA; University of California, Los Angeles, Office of the Human Research Protection Program, Los Angeles, CA; Tufts Health Sciences IRB, Boston, MA; Stanford University Research Compliance Office, Palo Alto, CA; Houston Methodist IRB, Houston, TX; and Human Investigation Committee, Yale University IRB, New Haven, CT. For sites in other countries, the study protocol was approved within each respective country by the following local IRBs or ethics committees: Melbourne Health Human Research Ethics Committee (Australia); Alfred Hospital Ethics Committee (Australia); Eastern Health Research and Ethics Committee (Australia); Austin Health Human Research Ethics Committee (Australia); Comité de Protection des Personnes Ouest I (France); Ethikkommission des Fachbereichs Medizin der Ludwig-Maximilians-Universität München (Germany); Comitato Etico dell’Azienda Ospedaliero Universitaria Policlinico Paolo Giaccone, Palermo (Italy); Comitato Etico Istituzioni Ospedaliere Cattoliche (Italy); Azienda Ospedaliera Universitaria Policlinico Umberto I–Università di Roma La Sapienza (Italy); Comitato Etico IRCCS Ospedale S. Raffaele di Milano (Italy); Comitato Etico per le Sperimentazioni Cliniche della Provincia di Vicenza (Italy); Adachi Kyosai Hospital IRB (Japan); Teikyo University Hospital, Mizonokuchi IRB (Japan); Tokyo Medical University Hospital IRB (Japan); Takeda Hospital Group IRB (Japan); Koseikai Sone Clinic IRB (Japan); National Center for Geriatrics and Gerontology IRB (Japan); Osaka University Hospital IRB (Japan); Bioetyczna przy Okregowej Izbie Lekarskiej w Gdansku (Poland); Hospital Universitari i Politecnic La Fe (Spain); and Etikprövningsmyndigheten (Sweden). All participants provided written informed consent before participating in any study-related activities. An independent data monitoring committee reviewed safety data on an ongoing basis.

### Biomarker substudies

Participants were assigned to either a tau PET substudy (based on the geographical availability of the tau PET radioligand) or a CSF substudy. Participants assigned to the tau PET substudy were provided the option to also participate in the CSF substudy (participants enrolled in both substudies were considered enrolled in the tau PET substudy for randomization purposes).

PET imaging was performed using [^18^F]MK-6240, a highly selective second-generation tau PET tracer that exhibits minimal off-target binding in patients with AD^48–50^. The tau PET SUVR was used to assess tau deposition in several target brain regions, including Braak I–II, III–IV and V–VI composite regions as defined by Maass et al.^51^ and Baker et al.^52^. The tau PET SUVR in a target brain region was calculated as the ratio of [^18^F]MK-6240 binding in the target region to that in a reference region (cerebellum, with superior sections eroded to minimize signal spillover from the occipital cortex).

Participants in the tau PET substudy underwent tau PET scans at baseline, 52 weeks and 78 weeks. Participants in the CSF substudy had CSF samples collected at baseline, 48 weeks and 76 weeks. A small subset of participants (*n* = 20) had measurements taken at 12 weeks instead of 76 weeks for early evaluation of pharmacokinetics and pharmacodynamics; this evaluation was performed as an interim analysis by a separate sponsor team, and the study team remained blinded. All CSF samples were collected by lumbar puncture and assayed for the levels of unbound N-terminal tau (MSD S-PLEX assay), p-tau181, total tau and Aβ_42_ (Lumipulse assay). All participants underwent MRI at baseline and 28, 52 and 78 weeks.

### Primary and secondary endpoints

For the placebo-controlled period of this study, the primary endpoint was the incidence of AEs and SAEs. The secondary endpoints were (1) the change in the CDR-SB score from baseline over time at week 78, and (2) the incidence of anti-gosuranemab serum antibody responses over time up to week 90.

### Exploratory endpoints

Key exploratory endpoints included (1) the change from baseline at week 78 in the ADAS-Cog13, MMSE, ADCS-ADL and FAQ scores; (2) the change from baseline in the CSF levels of unbound N-terminal tau; (3) the change from baseline in tau levels as measured by CSF testing (that is, t-tau, p-tau181) and tau PET; and (4) the change from baseline in brain volume as measured by MRI.

### Post hoc analyses for the LTE period

The primary endpoint for the LTE period in this study was the incidence of AEs and SAEs over the placebo-controlled and LTE periods. Analyses of key exploratory endpoints included continued assessment of the efficacy endpoints from the placebo-controlled period, such as CDR-SB, ADAS-Cog13, MMSE, ADCS-ADL and FAQ scores.

### Sample size

No formal sample size calculation was performed for the primary safety endpoint. Sample size calculation was based on the multiple comparison procedure—modeling approach. A sample size of 528 participants was planned to provide approximately 80% power to detect a dose–response relationship in the change from the baseline CDR-SB score (secondary objective) at 78 weeks, assuming a maximal 40% reduction with the highest gosuranemab dose compared to placebo and an estimated 20% dropout rate at 18 months (week 78) in this study. This calculation assumed an estimated mean change of 1.99 from the baseline CDR-SB score at 78 weeks in the placebo group and a common s.d. of 2.38, based on available data from Alzheimer’s Disease Neuroimaging Initiative (ADNI1, ADNI2 and ADNI GO) studies (amyloid positive from amyloid PET or CSF testing, MMSE score of ≥22, CDR global score of 0.5 for MCI and 0.5 or 1 for mild AD). All analyses were two-sided at a 5% significance level.

### Statistics and reproducibility

Statistical analyses were performed using SAS version 9.4. The analyses were performed by one statistical programmer, and the results were independently programmatically checked by a second statistical programmer and reviewed by two statisticians. The prespecified unblinded analysis was performed at the completion of the placebo-controlled period. Efficacy analyses were performed on the full analysis set (that is, all randomized participants who received the study treatment (gosuranemab or placebo)). Four participants were randomized but not dosed and excluded from the analysis. Secondary objective (CDR-SB) and key exploratory endpoints were analyzed using a mixed model for repeated measures (MMRM), with fixed effects of treatment, time, interaction between treatment and time, baseline value of the parameter of interest, interaction between the baseline value of the parameter of interest and time, baseline MMSE score, region, disease stage and baseline use of AD symptomatic medications. Model diagnostics were performed to evaluate the normality of data distribution and the impact of outliers. Missing data were assumed to be missing at random. Similar models were used for key secondary and exploratory endpoints. Additional analyses of efficacy endpoints from the LTE period were performed using data from the placebo-controlled and LTE periods.

Biomarker analyses (CSF testing, tau PET and structural MRI) were performed on either the evaluable set or the modified evaluable set for each type of analysis, in which the evaluable set consisted of all participants in the full analysis set who underwent the relevant procedure (lumbar puncture, PET or MRI) and the modified evaluable set consisted of the subset of the evaluable set with at least one postbaseline measurement of the specific parameter being analyzed. Biomarker analyses used an MMRM similar to that used for efficacy analyses; however, age was also used as a covariate for tau PET and MRI analyses, and region was not used as a covariate for CSF and tau PET analyses.

All safety analyses, except MRI safety analyses, were performed using data from all randomized participants who received at least one dose of the study treatment. MRI safety analyses were performed using data from all participants who received the study treatment and had at least one safety MRI scan after the baseline visit. Infusion reactions were defined as AEs that occurred on the day of or up to 2 days after an infusion.

### COVID-19

Measures were taken to mitigate risks caused by the COVID-19 pandemic and to circumvent issues related to site closures. Flexibility in site-visit scheduling was allowed, and all resulting protocol deviations had to be reported under the specific category of COVID-19-associated deviations. When in-person visits were not possible, safety surveillance and selected clinical assessments (CDR, ADCS-ADL, ISLT, Category Fluency Test and Letter Fluency Test from the Delis–Kaplan Executive Function System, and Columbia Suicide Severity Rating Scale) were allowed to be performed by telephone. Infusions at home or alternative sites and home-nursing options were permitted in some instances. Visits or procedures missed due to reasons related to the COVID-19 pandemic had to be completed as soon as possible and reported as delayed or missed with appropriate reasons provided.

### Reporting summary

Further information on research design is available in the Nature Portfolio Reporting Summary linked to this article.

### Supplementary information

Supplementary Information Study protocol and statistical analysis plans for the placebo-controlled and LTE periods.

Reporting Summary

Supplementary Table 1 ADAS-Cog13 results in the placebo-controlled and LTE periods. Analysis was performed using a mixed model for repeated measures with data from the placebo-controlled and LTE periods. Analyses were two-sided at a 5% significance level. No adjustments were made for multiple comparisons.

### Source data

Source Data Fig. 1 Statistical source data.

Source Data Fig. 2 Statistical source data.

Source Data Fig. 3 Statistical source data.

Source Data Fig. 4 Statistical source data.

Source Data Extended Data Fig. 1 Statistical source data.

Source Data Extended Data Fig. 2 Statistical source data.

Source Data Extended Data Fig. 3 Statistical source data.

Source Data Extended Data Fig. 4 Statistical source data.

## Data Availability

The trial results are publicly available at ClinicalTrials.gov (https://classic.clinicaltrials.gov/ct2/show/results/NCT03352557) and the EudraCT website (EudraCT no. 2017-002901-37, https://www.clinicaltrialsregister.eu/ctr-search/trial/2017-002901-37/results). Individual participant data collected during the trial may be shared after anonymization and upon approval of the research proposals in accordance with internal policies and procedures. Biogen commits to sharing patient-level data, study-level data, clinical study reports and protocols with qualified scientific researchers who provide a methodologically sound proposal. Biogen internally reviews all data requests based on the review criteria and in accordance with its Clinical Trial Transparency and Data Sharing Policy (available at https://www.biogentrialtransparency.com). Deidentified data and documents will be shared under agreements that further protect against participant reidentification. Access to data can be requested at https://vivli.org/. Source data are provided with this paper.

## References

[1] Long JM, Holtzman DM (2019). Alzheimer disease: an update on pathobiology and treatment strategies. Cell.

[2] Götz J, Halliday G, Nisbet RM (2019). Molecular pathogenesis of the tauopathies. Annu. Rev. Pathol..

[3] Josephs KA (2017). Current understanding of neurodegenerative diseases associated with the protein tau. Mayo Clin. Proc..

[4] Alzheimer’s Association. 2021 Alzheimer’s disease facts and figures. *Alzheimers Dement.* **17**, 327–406 (2021). 10.1002/alz.12328 33756057

[5] Fuster-Matanzo A, Hernández F, Ávila J (2018). Tau spreading mechanisms; implications for dysfunctional tauopathies. Int. J. Mol. Sci..

[6] Wang Y, Mandelkow E (2016). Tau in physiology and pathology. Nat. Rev. Neurosci..

[7] Guo T, Noble W, Hanger DP (2017). Roles of tau protein in health and disease. Acta Neuropathol..

[8] Noble W, Hanger DP, Gallo J-M (2010). Transgenic mouse models of tauopathy in drug discovery. CNS Neurol. Disord. Drug Targets.

[9] Irwin DJ (2016). Tauopathies as clinicopathological entities. Parkinsonism Relat. Disord..

[10] Mudher A (2017). What is the evidence that tau pathology spreads through prion-like propagation?. Acta Neuropathol. Commun..

[11] Trushina NI (2019). The evolution of tau phosphorylation and interactions. Front. Aging Neurosci..

[12] Chastagner P (2020). Fate and propagation of endogenously formed Tau aggregates in neuronal cells. EMBO Mol. Med..

[13] Brunello CA (2020). Mechanisms of secretion and spreading of pathological tau protein. Cell. Mol. Life Sci..

[14] DeVos SL (2018). Synaptic tau seeding precedes tau pathology in human Alzheimer’s disease brain. Front. Neurosci..

[15] Naseri NN (2019). The complexity of tau in Alzheimer’s disease. Neurosci. Lett..

[16] Qureshi IA (2018). A randomized, single ascending dose study of intravenous BIIB092 in healthy participants. Alzheimers Dement. (N. Y.).

[17] Boxer AL (2019). Safety of the tau-directed monoclonal antibody BIIB092 in progressive supranuclear palsy: a randomised, placebo-controlled, multiple ascending dose phase 1b trial. Lancet Neurol..

[18] Sopko R (2020). Characterization of tau binding by gosuranemab. Neurobiol. Dis..

[19] Dam T (2021). Safety and efficacy of anti-tau monoclonal antibody gosuranemab in progressive supranuclear palsy: a phase 2, randomized, placebo-controlled trial. Nat. Med..

[20] VandeVrede L (2020). Four-repeat tauopathies: current management and future treatments. Neurotherapeutics.

[21] Silva MC, Haggarty SJ (2020). Tauopathies: deciphering disease mechanisms to develop effective therapies. Int. J. Mol. Sci..

[22] Florian H (2023). Tilavonemab in early Alzheimer’s disease: results from a phase 2, randomized, double-blind study. Brain.

[23] Teng E (2022). Safety and efficacy of semorinemab in individuals with prodromal to mild Alzheimer disease: a randomized clinical trial. JAMA Neurol..

[24] Monteiro C (2023). A randomized phase II study of the safety and efficacy of semorinemab in participants with mild-to-moderate Alzheimer’s disease: Lauriet. Neurology.

[25] Chai X (2011). Passive immunization with anti-Tau antibodies in two transgenic models: reduction of Tau pathology and delay of disease progression. J. Biol. Chem..

[26] Yanamandra K (2013). Anti-tau antibodies that block tau aggregate seeding in vitro markedly decrease pathology and improve cognition in vivo. Neuron.

[27] Yanamandra K (2015). Anti-tau antibody reduces insoluble tau and decreases brain atrophy. Ann. Clin. Transl. Neurol..

[28] Ayalon G (2021). Antibody semorinemab reduces tau pathology in a transgenic mouse model and engages tau in patients with Alzheimer’s disease. Sci. Transl. Med..

[29] Barghorn S (2000). Structure, microtubule interactions, and paired helical filament aggregation by tau mutants of frontotemporal dementias. Biochemistry.

[30] Clavaguera F (2009). Transmission and spreading of tauopathy in transgenic mouse brain. Nat. Cell Biol..

[31] Ahmed Z (2014). A novel in vivo model of tau propagation with rapid and progressive neurofibrillary tangle pathology: the pattern of spread is determined by connectivity, not proximity. Acta Neuropathol..

[32] Boluda S (2015). Differential induction and spread of tau pathology in young PS19 tau transgenic mice following intracerebral injections of pathological tau from Alzheimer’s disease or corticobasal degeneration brains. Acta Neuropathol..

[33] Gibbons GS, Lee VMY, Trojanowski JQ (2019). Mechanisms of cell-to-cell transmission of pathological tau: a review. JAMA Neurol..

[34] Guo JL (2016). Unique pathological tau conformers from Alzheimer’s brains transmit tau pathology in nontransgenic mice. J. Exp. Med..

[35] Iba M (2013). Synthetic tau fibrils mediate transmission of neurofibrillary tangles in a transgenic mouse model of Alzheimer’s-like tauopathy. J. Neurosci..

[36] Peeraer E (2015). Intracerebral injection of preformed synthetic tau fibrils initiates widespread tauopathy and neuronal loss in the brains of tau transgenic mice. Neurobiol. Dis..

[37] Stancu I-C (2015). Templated misfolding of Tau by prion-like seeding along neuronal connections impairs neuronal network function and associated behavioral outcomes in Tau transgenic mice. Acta Neuropathol..

[38] Albert M (2019). Prevention of tau seeding and propagation by immunotherapy with a central tau epitope antibody. Brain.

[39] Castillo-Carranza DL (2014). Passive immunization with Tau oligomer monoclonal antibody reverses tauopathy phenotypes without affecting hyperphosphorylated neurofibrillary tangles. J. Neurosci..

[40] Sankaranarayanan S (2015). Passive immunization with phospho-tau antibodies reduces tau pathology and functional deficits in two distinct mouse tauopathy models. PLoS ONE.

[41] Falcon B (2015). Conformation determines the seeding potencies of native and recombinant Tau aggregates. J. Biol. Chem..

[42] Horie K (2021). CSF tau microtubule binding region identifies tau tangle and clinical stages of Alzheimer’s disease. Brain.

[43] Rustom A (2004). Nanotubular highways for intercellular organelle transport. Science.

[44] Théry C (2001). Proteomic analysis of dendritic cell-derived exosomes: a secreted subcellular compartment distinct from apoptotic vesicles. J. Immunol..

[45] Ruan Z (2021). Alzheimer’s disease brain-derived extracellular vesicles spread tau pathology in interneurons. Brain.

[46] Albert MS (2011). The diagnosis of mild cognitive impairment due to Alzheimer’s disease: recommendations from the National Institute on Aging–Alzheimer’s Association workgroups on diagnostic guidelines for Alzheimer’s disease. Alzheimers Dement..

[47] McKhann GM (2011). The diagnosis of dementia due to Alzheimer’s disease: recommendations from the National Institute on Aging–Alzheimer’s Association workgroups on diagnostic guidelines for Alzheimer’s disease. Alzheimers Dement..

[48] Betthauser TJ (2019). In vivo characterization and quantification of neurofibrillary tau PET radioligand ^18^F-MK-6240 in humans from Alzheimer disease dementia to young controls. J. Nucl. Med..

[49] Lohith TG (2019). Brain imaging of Alzheimer dementia patients and elderly controls with ^18^F-MK-6240, a PET tracer targeting neurofibrillary tangles. J. Nucl. Med..

[50] Leuzy A (2019). Tau PET imaging in neurodegenerative tauopathies—still a challenge. Mol. Psychiatry.

[51] Maass A (2017). Comparison of multiple tau-PET measures as biomarkers in aging and Alzheimer’s disease. Neuroimage.

[52] Baker SL, Maass A, Jagust WJ (2017). Considerations and code for partial volume correcting [^18^F]-AV-1451 tau PET data. Data Brief.

