# Supplementary material for: TANGO: a placebo-controlled randomized phase 2 study of efficacy and safety of the anti-tau monoclonal antibody gosuranemab in early Alzheimer’s disease
Source: Nat Aging. 2023 Nov 27;3(12):1591–601. doi: 10.1038/s43587-023-00523-w (PMC10724064; doi:10.1038/s43587-023-00523-w)
Supplement: Supplementary file 1 — Study protocol and statistical analysis plans for the placebo-controlled and LTE periods. [file 43587_2023_523_MOESM1_ESM.pdf]

nature aging

Article

https://doi.org/10.1038/s43587-023-00523-w

TANGO: a placebo-controlled randomized phase 2 study of efficacy and safety of the anti-tau monoclonal antibody gosuranemab in early Alzheimer's disease

In the format provided by the authors and unedited

Biogen MA Inc.
225 Binney Street
Cambridge, MA 02142
United States

Biogen Idec Research Limited
Innovation House
70 Norden Road
Maidenhead Berkshire
SL6 4AY
United Kingdom

PROTOCOL NUMBER: 251AD201/NCT03352557

PHASE OF DEVELOPMENT: 2

PROTOCOL TITLE: Randomized, Double-Blind, Placebo-Controlled, Parallel-Group Study to Assess the Safety, Tolerability, and Efficacy of BIIB092 in Subjects with Mild Cognitive Impairment due to Alzheimer's Disease or with Mild Alzheimer's Disease

EUDRA CT NUMBER: 2017-002901-37

IND NUMBER: 136300

DATE: 13 January 2020
Version 4
Final

Supersedes previous Version 3 dated 29 March 2019

CONFIDENTIAL
The information contained herein may not be used, disclosed, or published without the written consent of Biogen MA Inc.

SPONSOR SIGNATURE PAGE

Protocol 251AD201 was approved by:

[redacted] 17 - January - 2020

[redacted], PhD Date

[redacted]

Biogen

TABLE OF CONTENTS

SPONSOR SIGNATURE PAGE 2
1. SYNOPSIS 9
2. LIST OF ABBREVIATIONS 17
3. SPONSOR INFORMATION 19
4. INTRODUCTION 20
4.1. Overview of Alzheimer's Disease 20
4.2. Current Therapies for Alzheimer's Disease 21
4.3. Profile of Previous Experience With BIIB092 21
4.3.1. Nonclinical Experience 21
4.3.2. Clinical Experience 21
4.4. Study Rationale 22
4.5. Overall Benefits and Risks Assessment 24
4.5.1. Overall Benefit 24
4.5.2. Potential Risks 24
4.5.3. Summary 25
5. SCHEDULE OF ACTIVITIES 26
6. STUDY OBJECTIVES AND ENDPOINTS 47
7. STUDY DESIGN 51
7.1. Study Overview 51
7.2. Study Duration for Participants 53
[redacted] 55
7.4. Study Stopping Rules 56
7.5. End of Study 56
8. SELECTION OF PARTICIPANTS 57
8.1. Inclusion Criteria 57
8.2. Exclusion Criteria 58
8.3. Inclusion Criteria for Long-Term Extension Period 64
8.4. Exclusion Criteria for Long-Term Extension Period 65
9. SCREENING AND RANDOMIZATION 67
9.1. Screening 67
9.2. Randomization 68

9.3. Blinding Procedures....69
10. DISCONTINUATION OF STUDY TREATMENT AND WITHDRAWAL OF PARTICIPANTS FROM THE STUDY....71
10.1. Discontinuation of Study Treatment....71
10.2. Lost to Follow-Up....72
10.3. Withdrawal of Participants From Study....72
11. STUDY TREATMENT USE....74
11.1. Regimen....74
11.1.1. Placebo-Controlled Period....74
11.1.2. Long-Term Extension Period....74
11.2. Modification of Dose and/or Treatment Schedule....74
11.2.1. Dose Suspension....75
11.2.2. Infusion Interruption....75
11.3. Compliance....75
11.4. Concomitant Therapy and Procedures....75
11.4.1. Concomitant Therapy....75
11.4.1.1. Allowed Concomitant Therapy....76
11.4.1.2. Disallowed Concomitant Therapy....76
11.4.2. Concomitant Procedures....78
11.4.3. Tobacco Use....78
11.5. Continuation of Treatment....78
12. STUDY TREATMENT MANAGEMENT....79
12.1. BIIB092....79
12.1.1. BIIB092 Preparation....79
12.1.2. BIIB092 Storage....79
12.1.3. BIIB092 Handling and Disposal....80
12.1.4. BIIB092 Accountability....80
12.2. Placebo....80
12.3. Additional Protocol-Designated Products – [redacted]....80
13. EFFICACY, PHARMACOKINETIC, PHARMACODYNAMIC, IMMUNOGENICITY, AND HEALTH OUTCOMES ASSESSMENTS....82
13.1. Clinical Efficacy Assessments....82

13.2. Pharmacokinetic Assessments 82
13.3. Pharmacodynamic Assessments 83
[redacted] [redacted] 83
[redacted] [redacted] 84
13.6. Immunogenicity 84
13.7. Health Outcomes Assessments 85
14. SAFETY ASSESSMENTS 86
14.1. Clinical Safety Assessments 86
14.2. Laboratory Safety Assessments 86
15. SAFETY DEFINITIONS, RECORDING, REPORTING, AND RESPONSIBILITIES 88
15.1. Definitions 88
15.1.1. Adverse Event 88
15.1.2. Serious Adverse Event 88
15.1.3. Prescheduled or Elective Procedures or Routinely Scheduled Treatments 89
15.2. Safety Classifications 89
15.2.1. Investigator Assessment of Events 89
15.2.2. Relationship of Events to Study Treatment [redacted] 90
15.2.3. Severity of Events 91
15.2.4. Expectedness of Events 91
15.3. Monitoring and Recording Events 91
15.3.1. Adverse Events 91
15.3.2. Adverse Events of Special Interest 92
15.3.3. Serious Adverse Events 92
15.3.4. Immediate Reporting of Serious Adverse Events 92
15.3.4.1. Deaths 92
15.3.5. Suspected Unexpected Serious Adverse Reactions 93
15.4. Procedures for Handling Special Situations 93
15.4.1. Pregnancy 93
15.4.2. Overdose 93
15.4.3. Medical Emergency 93
15.4.3.1. Unblinding for Medical Emergency 94

15.5. Contraception Requirements 94
15.6. Safety Responsibilities....95
15.6.1. The Investigator 95
15.6.2. Biogen....96
16. STATISTICAL METHODS AND DETERMINATION OF SAMPLE SIZE....97
16.1. General Considerations....97
16.2. Demography and Baseline Disease Characteristics....97
16.3. Safety 97
16.3.1. Analysis Population 97
16.3.2. Methods of Analysis 97
16.3.2.1. Adverse Events 97
16.3.2.2. Clinical Laboratory Results 98
16.3.2.3. Vital Signs 98
16.3.2.4. Electrocardiograms 98
16.3.2.5. Physical and Neurological Examinations 98
16.3.2.6. Columbia - Suicide Severity Rating Scale....98
16.4. Efficacy....98
16.4.1. Analysis Population 98
16.4.2. Methods of Analysis 99
16.4.2.1. Analysis of Primary Efficacy Endpoint (Clinical Dementia Rating Scale – Sum of Boxes) 99
16.4.2.2. Analysis of Exploratory Clinical Endpoints 99
16.4.3. Analysis for the Long-Term Extension Period....100
16.5. Pharmacokinetics....100
16.5.1. Analysis Population 100
16.5.2. Methods of Analysis 100
16.6. Pharmacodynamics 101
16.6.1. Analysis Population 101
16.6.2. Methods of Analysis for Tau Positron Emission Tomography Results 101
16.6.3. Methods of Analysis for Other Pharmacodynamic Parameters....101
16.7. Health Outcomes 102
16.7.1. Analysis Population 102

16.7.2. Methods of Analysis 102
16.8. Immunogenicity 102
16.8.1. Analysis Population 102
16.8.2. Methods of Analysis 102
16.9. Interim Analyses 102
16.10. Sample Size Considerations 103
17. ETHICAL REQUIREMENTS 105
17.1. Declaration of Helsinki 105
17.2. Ethics Committee 105
17.3. Participant Information and Consent 106
17.4. Participant Data Protection 107
17.5. Compensation for Injury 108
17.6. Conflict of Interest 108
17.7. Registration of Study and Disclosure of Study Results 108
18. ADMINISTRATIVE PROCEDURES 109
18.1. Study Site Initiation 109
18.2. Quality Control and Quality Assurance 109
18.3. Monitoring of the Study 109
18.4. Study Funding 110
18.5. Publications 110
19. FURTHER REQUIREMENTS AND GENERAL INFORMATION 111
19.1. External Contract Organizations 111
19.1.1. Contract Research Organization 111
19.1.2. Interactive Response Technology 111
19.1.3. Electronic Data Capture 111
19.1.4. Central Laboratories for Laboratory Assessments 111
19.1.5. Central Facility for Other Assessments 111
19.1.6. Neurocognitive Assessments 112
19.2. Study Committees 112
19.3. Changes to Final Study Protocol 112
19.4. Ethics Committee Notification of Study Completion or Termination 112
19.5. Retention of Study Data 113

19.6. Study Report Signatory.....113
20. REFERENCES.....114
21. SIGNED AGREEMENT OF THE STUDY PROTOCOL.....115

LIST OF TABLES

Table 1: Schedule of Activities During the Placebo-Controlled Period and the LTE Screening Period.....27
Table 2: Schedule of Activities for Week 80 Through Week 128 of the Long-Term Extension Period.....35
Table 3: Schedule of Activities for Week 132 Through Week 176 of the Long-Term Extension Period.....39
Table 4: Schedule of Activities for Week 180 Through Week 238 or Follow-Up Visit of the Long-Term Extension Period.....43

LIST OF FIGURES

Figure 1: Study Design.....53
Figure 2: Dose-Response Shapes Based on Clinical Dementia Rating Scale – Sum of Boxes Mean Change from Baseline at Week 78.....104

1. SYNOPSIS

Protocol Title:	Randomized, Double-Blind, Placebo-Controlled, Parallel-Group Study to Assess the Safety, Tolerability, and Efficacy of BIIB092 in Subjects with Mild Cognitive Impairment due to Alzheimer's Disease or with Mild Alzheimer's Disease
Protocol Number:	251AD201
Version Number:	4
Name of Study Treatment:	BIIB092 (formerly known as BMS 986168, IPN007)
Study Phase:	2
Study Indication:	Mild cognitive impairment (MCI) due to Alzheimer's disease (AD) or mild AD
Study Rationale:	BIIB092 is a humanized immunoglobulin G4 monoclonal antibody that has been shown to bind tau at the amino terminus. Although tau is primarily an intracellular protein, a portion of tau is secreted by neurons as N-terminal tau fragments.<br><br>BIIB092 has been shown to lower cerebrospinal fluid (CSF) concentrations of N-terminal tau in nonclinical studies, a single-ascending-dose study in healthy participants, and a multiple-ascending-dose study in participants with progressive supranuclear palsy. Based upon the close links between tau pathology, neurodegeneration, and clinical features consistent with AD, coupled with the evidence that tau pathology can spread via neuronal release and uptake of pathological tau species, examination of BIIB092 in the clinical AD setting is strongly supported as a potential disease-modifying therapy. This study will assess the safety and efficacy of BIIB092 in participants with MCI due to AD or with mild AD and help to inform on dose selection in this patient population.
Study Objectives and Endpoints:	**Placebo-Controlled Period**<br><br>The primary objective of the study for the placebo-controlled period is to evaluate the safety and tolerability of BIIB092 in participants with MCI due to AD or with mild AD.<br><br>The primary safety endpoints that relate to this objective are the incidence of adverse events (AEs) and serious AEs (SAEs) during the

placebo-controlled period.

Secondary objectives and endpoints are as follows:

- To evaluate the efficacy of multiple doses of BIIB092 in slowing cognitive and functional impairment in participants with MCI due to AD or with mild AD as measured by the change from Baseline over time at Week 78 on the Clinical Dementia Rating Scale (CDR) - Sum of Boxes (CDR-SB). This is the primary efficacy objective, with the primary efficacy endpoint.

- To evaluate the immunogenicity of BIIB092 after multiple doses in participants with MCI due to AD or with mild AD as measured by the incidence of anti-BIIB092 antibodies in serum over time up to Week 90.

Exploratory objectives and endpoints are as follows:

- To assess the effect of BIIB092 on the clinical progression of AD as measured by changes from Baseline over time up to Week 78 on the Mini-Mental State Examination (MMSE), International Shopping List Test Immediate Recall (ISLT), Category Fluency and Letter Fluency tests from the Delis-Kaplan Executive Function System (DKEFS), Digit Symbol Substitution Test (DSST), Trail Making Test Part A (Trails A), Everyday Cognition (eCog), Alzheimer's Disease Cooperative Study-Activities of Daily Living (ADCS-ADL), Functional Activities Questionnaire (FAQ), Alzheimer's Disease Assessment Scale-Cognitive (ADAS-Cog 13 [13 item]), and Neuropsychiatric Inventory-10 (NPI-10).

- To assess the effect of BIIB092 on changes in caregiver burden and participant quality of life as measured by changes from Baseline over time up to Week 68 on the Zarit Burden Interview (ZBI) and Quality of Life for Alzheimer's Disease (QoL-AD).

- To assess the effect of BIIB092 on resource utilization as measured by the Resource Utilization in Dementia-Lite (RUD-Lite) Version results over time up to Week 68.

- To assess BIIB092 pharmacokinetics (PK) in serum (trough serum BIIB092 concentrations and end-of-infusion serum BIIB092 concentrations) from the samples collected at the

CONFIDENTIAL
The information contained herein may not be used, disclosed, or published without the written consent of Biogen MA Inc.

visits indicated in the Schedule of Activities in participants with MCI due to AD or with mild AD.

- [redacted]
- [redacted]
- To assess the effect of BIIB092 on biomarkers in blood as measured by changes from Baseline over time up to Week 76 on blood levels of disease-related biomarkers, including but not limited to, tau and other markers of neurodegenerative disease.
- [redacted]
- [redacted]
- To assess the effect of BIIB092 on brain structure as measured by changes from Baseline over time up to Week 78 on magnetic resonance imaging (MRI) morphometric measures, including volume and cortical thickness of certain brain areas.

Long-Term Extension Period

The primary objective for the long-term extension (LTE) period is to evaluate the long-term safety and tolerability of BIIB092 in participants with MCI due to AD or with mild AD.

The primary safety endpoint that relates to this objective is the

incidence of AEs and SAEs over the placebo-controlled period and LTE period of the study.

Exploratory objectives and endpoints are as follows:

- To further evaluate the immunogenicity of BIIB092 after multiple doses in participants with MCI due to AD or with mild AD as measured by the incidence of anti-BIIB092 antibodies in serum over time up to Week 238.

- To further assess the effect of BIIB092 on disease progression as measured by fluid and radiological biomarkers, as well as additional clinical and health outcomes as measured by the following:

  - Changes over the placebo-controlled period and LTE period on the CDR-SB, MMSE, ISLT, Category Fluency and Letter Fluency tests from the DKEFS, DSST, Trails A, eCog, ADCS-ADL, FAQ, ADAS-Cog-13 (13-item), and NPI-10

  - Changes over the placebo-controlled period and LTE period on the ZBI, QoL-AD, and RUD-Lite

  - [redacted]

  - Changes over the placebo-controlled period and LTE period on [redacted] MRI brain morphometric measures

- To assess BIIB092 PK in serum (trough serum BIIB092 concentrations) [redacted] from the samples collected at the visits indicated in the Schedule of Activities in participants with MCI due to AD or with mild AD.

Study Design: This is a Phase 2, randomized, double-blind, placebo-controlled, parallel-group study consisting of a double-blind, placebo-controlled period and an LTE period.

[redacted]

[redacted]

Study Location:	Approximately 100 study sites globally are planned.
Number of Planned Participants:	Approximately 528 participants were planned to be randomized. Due to fast recruitment, the study was over-enrolled and 654 participants have been randomized.
Study Population:	This study will be conducted in participants aged 50 to 80 years, inclusive, with MCI due to AD or mild AD according to National Institute on Aging-Alzheimer's Association criteria. [redacted] Participants must also perform at 1 standard deviation below the age-adjusted normative mean on either the ISLT or the International Shopping List Test Delayed Recall and have a CDR global score of 0.5 for MCI due to AD or 0.5 or 1 for mild AD, an MMSE score of 22 to 30 (inclusive), and a CDR Memory Box score of ≥0.5.<br><br>Detailed eligibility criteria are described in Section 8.
Treatment Groups:	During the placebo-controlled period, randomized participants will receive 1 of the following study treatments by intravenous (IV) infusion, starting on Study Day 1:<br><br>• low-dose BIIB092 - 125 mg once every 4 weeks or 375 mg once every 12 weeks (88 participants planned in total, 44 per regimen)<br>• medium-dose BIIB092 - 600 mg once every 4 weeks (88 participants planned)<br>• high-dose BIIB092 - 2000 mg once every 4 weeks (176 participants planned)<br>• placebo (176 participants planned)

During the dose-blinded LTE period, participants will receive BIIB092 by IV once every 4 weeks beginning at Week 80. Participants who were randomized to receive BIIB092 during the placebo-controlled period will continue to receive BIIB092 at the dose they were randomly assigned. Participants randomized to receive placebo during the placebo-controlled period will receive BIIB092 at the high dose (2000 mg) once every 4 weeks during the LTE period.

Duration of Treatment and Follow-up:

The total duration for participants who complete the placebo-controlled period and do not enter the LTE period will be approximately 99 weeks. For participants who complete both the placebo-controlled period and the LTE period, the total study duration will be approximately 247 weeks.

Placebo-Controlled Period

Participation in the placebo-controlled period will be approximately 99 weeks, which includes the Screening Period of approximately 9 weeks, the 76-week Treatment Period, and the End-of-Study (EOS) Visit at Week 78, and for participants not entering the LTE period, a Follow-up Safety Visit at Week 90, or approximately 14 weeks after the last dose of study treatment. It is recommended that all the screening procedures be completed within 65 days; however, the overall Screening Period may be extended up to 90 days in consultation with the Medical Monitor in advance and provided that written documentation is received from the Sponsor or delegate [redacted]. Participants will have approximately 25 outpatient clinic visits:

- Participant eligibility will be determined at up to 3 visits during the Screening Period (Screening Visits 1, 2, and 3).
- On Study Day 1, eligible participants will be randomized, have scheduled assessments per the Schedule of Activities, and receive the first infusion of randomized study treatment.
- Participants will return to the clinic at 4-week intervals during the Treatment Period for infusions of study treatment and study assessments (Weeks 4, 8, 12, 16, 20, 24, 28, 32, 36, 40, 44, 48, 52, 56, 60, 64, 68, 72, and 76). The last dose of study treatment will be administered at Week 76 (End-of-Treatment Visit).
- For a participant who completes the Treatment Period at Week 76, there will be an EOS Visit at Week 78 for final

study assessments of the placebo-controlled period. A Follow-up Safety Visit will occur at Week 90 (14 weeks after the last dose of study treatment is administered), unless the participant elects to enter an extension study following the Week 78 Visit.

- Participants who discontinue study treatment prematurely will be asked to remain in the study and continue all protocol-specified visits and procedures. These participants should be encouraged to attend at least the Week 24, Week 52, and Week 78 visits, depending on when treatment was discontinued, and undergo all the scheduled procedures. At a minimum, these visits should include assessment of AEs/SAEs and concomitant medications and key clinical assessments, including at least the CDR, MMSE, ISLT, ADAS-Cog 13, ADCS-ADL, and FAQ. Participants who discontinue study treatment prematurely will also be asked to return to the study site for a Follow-up Safety Visit 14 weeks after receiving the last dose of study treatment.

- Participants who withdraw from the study prematurely are to return to the study site for an Early Termination (ET) Visit and assessments and for the Follow-up Safety Visit 14 weeks after administration of the final infusion of study treatment.

Long-Term Extension Period

Participants who meet the LTE inclusion and exclusion criteria will be eligible to enter the dose-blinded LTE period, which includes the LTE Screening Period of approximately 4 weeks starting at Week 76, the 144-week Treatment Period starting at Week 80, the EOS Visit at Week 226, and the Follow-up Visit at Week 238.

Participants will have approximately 39 outpatient clinic visits during the LTE period:

- Participant eligibility will be determined at Week 76 or Week 78 of the placebo-controlled period and confirmed at Week 80 of the LTE period.

- At the Week 80 Visit, eligible participants will have scheduled assessments per the Schedule of Activities and receive the first infusion of BIIB092 during the LTE period.

CONFIDENTIAL
The information contained herein may not be used, disclosed, or published without the written consent of Biogen MA Inc.

All participants receiving placebo in the placebo-controlled period will receive BIIB092 at the high dose (2000 mg) once every 4 weeks during the LTE period.

- Participants will return to the clinic at 4-week intervals during the Treatment Period for infusions of study treatment and study assessments.
- The last dose of study treatment will be administered at Week 224 (End-of-Treatment Visit). The EOS Visit will occur at Week 226 and the Follow-up Visit at Week 238.
  - Participants who discontinue study treatment prematurely will be asked to remain in the study and continue all protocol-specified visits and procedures. These participants should be encouraged to attend at least the Week 80, 104, 116, 128, 152, 156, 176, 196, 200, 224, and 226 Visits, depending on when treatment was discontinued, and undergo all the scheduled procedures. At a minimum, these visits should include assessment of AEs/SAEs and concomitant medications and key clinical assessments, including at least the CDR, MMSE, ISLT, ADAS-Cog 13, ADCS-ADL, and FAQ. Participants who discontinue study treatment prematurely will also be asked to return to the study site for a Follow-up Safety Visit 14 weeks after receiving the last dose of study treatment.
  - Participants who withdraw from the study prematurely are to return to the study site for an Early Termination Visit and assessments and for the Follow-up Safety Visit 14 weeks after receiving the last dose of study treatment.

2. LIST OF ABBREVIATIONS

Aβ	amyloid beta
AD	Alzheimer's disease
ADAS-Cog 13	Alzheimer's Disease Assessment Scale – Cognitive (13 item)
ADCS-ADL	Alzheimer's Disease Cooperative Study – Activities of Daily Living
ADNI	Alzheimer's Disease Neuroimaging Initiative
AE	adverse event
ALT	alanine aminotransferase
anti-HBc	hepatitis B core antibody
anti-HBs	hepatitis B surface antibody
[redacted]	[redacted]
AST	aspartate aminotransferase
BUN	blood urea nitrogen
CBC	complete blood count
CDR	Clinical Dementia Rating Scale
CDR–SB	Clinical Dementia Rating Scale – Sum of Boxes
CI	confidence interval
CRF	case report form
CRO	contract research organization
[redacted]	[redacted]
C-SSRS	Columbia – Suicide Severity Rating Scale
DBP	diastolic blood pressure
DHA	Directions for Handling and Administration
DKEFS	Delis-Kaplan Executive Function System
DMC	Data Monitoring Committee
[redacted]	[redacted]
DSST	Digit Symbol Substitution Test
ECG	electrocardiogram
eCog	Everyday Cognition
Emax	maximum response
EOS	end of study
ET	early termination
FAQ	Functional Activities Questionnaire
[redacted]	[redacted]
FSH	follicle-stimulating hormone
GCP	Good Clinical Practice
HBsAg	hepatitis B surface antigen
HCV	hepatitis C virus
HIV	human immunodeficiency virus
ICF	informed consent form
ICH	International Council for Harmonisation

CONFIDENTIAL
The information contained herein may not be used, disclosed, or published without the written consent of Biogen MA Inc.

Ig	immunoglobulin
INR	international normalized ratio
IRT	interactive response technology
ISLR	International Shopping List Test Delayed Recall
ISLT	International Shopping List Test Immediate Recall
ITT	intent-to-treat
IV	intravenous
[redacted]	[redacted]
LS	least squares
LTE	long-term extension
MAD	multiple-ascending-dose
MAO	monoamine oxidase
MCI	mild cognitive impairment
MCP – MOD	multiple comparison procedure – modelling
MMRM	mixed model with repeated measures
MMSE	Mini-Mental State Examination
MRI	magnetic resonance imaging
NFT	neurofibrillary tangle
NIA-AA	National Institute on Aging – Alzheimer's Association
NPI-10	Neuropsychiatric Inventory – 10
PD	pharmacodynamic(s)
PET	positron emission tomography
PK	pharmacokinetic(s)
PSP	progressive supranuclear palsy
QoL-AD	Quality of Life for Alzheimer's Disease
[redacted]	[redacted]
RUD-Lite	Resource Utilization in Dementia-Lite Version
SAD	single-ascending-dose
SAE	serious adverse event
SAP	statistical analysis plan
SBP	systolic blood pressure
SD	standard deviation
SUSAR	suspected unexpected serious adverse reaction
Trails A	Trail Making Test, Part A
ULN	upper limit of normal
UTI	urinary tract infection
UV	unscheduled visit
ZBI	Zarit Burden Interview

CONFIDENTIAL
The information contained herein may not be used, disclosed, or published without the written consent of Biogen MA Inc.

3. SPONSOR INFORMATION

Biogen MA Inc.
225 Binney Street
Cambridge, MA 02142
United States

Biogen Idec Research Limited
Innovation House
70 Norden Road
Maidenhead, Berkshire
SL6 4AY
United Kingdom

Biogen Japan Ltd.
Nihonbashi 1-chome
Mitsui Building 14F
4-1 Nihonbashi 1-chome
Chuo-ku, Tokyo
103-0027 Japan

Biogen Australia PTY Ltd.
Suite 1, Level 3
123 Epping Road
North Ryde, NSW 2113
Australia

For urgent medical issues in which the study Medical Director should be contacted, please see the Study Reference Guide's Official Study Contact List for complete contact information.

Biogen may transfer any or all of its study-related responsibilities to a contract research organization (CRO) and other third parties; however, Biogen retains overall accountability for these activities.

CONFIDENTIAL
The information contained herein may not be used, disclosed, or published without the written consent of Biogen MA Inc.

4. INTRODUCTION

4.1. Overview of Alzheimer's Disease

Alzheimer's disease (AD) is a progressive neurodegenerative disorder clinically characterized by cognitive impairment, behavioral disturbances, psychiatric symptoms, and disability in activities of daily living. These clinical manifestations constitute the syndrome dementia. AD International estimates that the number of people living with dementia worldwide will increase from (the current estimate of) 46.8 million to 131.5 million by 2050 [Alzheimer's Disease International 2015]. As the most common cause of dementia, AD accounts for 60% to 80% of dementia cases [Alzheimer's Association 2015].

The main neuropathological hallmarks of AD are extracellular senile (neuritic) plaques containing aggregated β-amyloid (Aβ) peptides and intraneuronal neurofibrillary tangles (NFTs) composed of abnormal hyperphosphorylated tau protein.

The amyloid hypothesis of AD etiology postulates that misfolded Aβ peptide is a causative factor, which is strongly supported by human genetics [Hardy and Selkoe 2002]. While the evidence for Aβ as the initiator of the pathophysiological cascade leading to AD dementia is strong, the link between Aβ pathology and neurodegeneration in AD and the subsequent clinical progression, both temporally and anatomically, is relatively weak [Musiek and Holtzman 2015; Serrano-Pozo 2011]. Amyloid plaques are present in patients with AD 10 to 15 years before onset of AD symptoms and overt neuronal loss, and they reach a plateau around clinical onset [Jack 2013].

A much stronger correlation has been demonstrated between tau pathology (in the form of NFTs of the aggregated, misfolded microtubule-associated protein tau) and neuronal loss and cognitive decline. In AD, atrophy as seen on magnetic resonance imaging (MRI) and hypometabolism as seen with fluorodeoxyglucose (FDG) positron emission tomography (PET), correlate highly with postmortem histochemical analysis of synaptic and neuron loss and, importantly, tau burden [Jack 2013; Serrano-Pozo 2011]. Imaging data obtained using novel PET tau radioligands alone or in combination with functional and structural imaging methods (e.g., FDG PET or structural MRI) as well as cognitive testing demonstrated stronger correlations between tau PET signal and neurodegeneration, hypometabolism, and cognitive deficits, than for amyloid PET signal [Johnson 2016; Pontecorvo 2017]. While these are early findings, and a better understanding of the existing tau PET imaging agents as well as longitudinal studies are needed, the recent findings lend further support to the close link between tau pathology, neurodegeneration and AD symptoms.

There are currently no approved therapies that modify the course of AD. However, due to the central role of tau in neurodegeneration and its close correlation with cognitive decline in AD, modification of tau pathology has potential for the treatment of AD.

4.2. Current Therapies for Alzheimer's Disease

No new therapies for AD have been approved in more than a decade. Currently approved therapies (and their approval dates) include the central cholinesterase inhibitors (donepezil [Aricept®; 2004], rivastigmine [Exelon®; 2000], and galantamine [Reminyl®; 2001]) and the N-methyl-D-aspartate antagonist memantine (Ebixa®; Namenda®; 2003). These medications provide only symptomatic benefit and do not attenuate the course of the disease [Birks 2006; McShane 2006].

4.3. Profile of Previous Experience With BIIB092

Please see the BIIB092 Investigator's Brochure for detailed information on relevant nonclinical and clinical studies.

4.3.1. Nonclinical Experience

In nonclinical pharmacology studies, BIIB092 was shown to recognize a linear, nonphosphorylated, amino terminal epitope (including amino acid residues 15-24 of full-length human tau) of the microtubule-associated protein tau. BIIB092 exhibited high affinity for human and cynomolgus monkey tau, with binding affinities of 7.10^{-10} M and 6.35^{-10} M, respectively. The primary site of action of BIIB092 is believed to be the interstitial space between neurons where it binds tau, thereby reducing eTau-mediated spread of tau pathology, and this proposed site of action is supported by nonclinical studies.

Pharmacokinetic (PK) and pharmacodynamic (PD) analysis of serum and cerebrospinal fluid (CSF) BIIB092 concentrations from 3 experiments in cynomolgus monkeys (intravenous [IV] injection/infusion doses of 0.5 to 60 mg/kg) showed that CSF N-terminal tau levels were reduced in a dose-dependent manner. The duration of reduction of CSF N-terminal tau was also dose-dependent. Higher doses were associated with longer reductions of N-terminal tau in CSF. For example, the reduction of N-terminal tau in CSF (25% to 50%) persisted for more than 58 days at doses ≥20 mg/kg.

The nonclinical toxicology studies conducted to date have demonstrated an acceptable safety profile for BIIB092 to support continued use in clinical studies.

4.3.2. Clinical Experience

The present study is the first study of BIIB092 in AD. Previous clinical studies have been conducted to support development of BIIB092 for the treatment of patients with progressive supranuclear palsy (PSP).

BIIB092 has been evaluated in 4 completed or ongoing clinical studies (Studies CN002001, CN002003, 251PP201 [CN002004], and 251PP301 [CN002012]):

- Study CN002001, the first-in-human study of BIIB092, was a Phase 1, randomized, double-blind, placebo-controlled, single-ascending-dose (SAD) study in healthy participants to characterize the safety, tolerability, PK, PD, and immunogenicity of

single doses of BIIB092 ranging from 21 mg to 4200 mg. Single doses of BIIB092 were safe and well tolerated at all dose levels tested. The extent and duration of suppression of CSF N-terminal tau increased with increasing dose. Following single doses of BIIB092, the mean suppression of CSF N-terminal tau on Study Day 29 ranged from 65% to 96% at doses ranging from 70 mg to 4200 mg. Greater than 80% suppression of CSF N-terminal tau was achieved with single doses of BIIB092 ≥210 mg. The suppression of N-terminal tau following dosing with BIIB092 persisted over the course of the 12-week study.

- Study CN002003 was a Phase 1b, randomized, double-blind, placebo-controlled multiple-ascending-dose (MAD) study to characterize the safety, tolerability, PK, PD, and immunogenicity of BIIB092 doses of 150 mg, 700 mg, and 2100 mg administered every 4 weeks in participants with PSP. All 3 doses were safe and well tolerated, based on results from the dose-escalation phase of the study. Treatment with multiple monthly doses of BIIB092 decreased CSF N-terminal tau by mean values of approximately 90%, 93%, and 96% on Study Day 29 and 91%, 95%, and 97% on Study Day 85 at doses of 150, 700, and 2100 mg, respectively.

- Study 251PP201 is an ongoing Phase 1b open-label extension study to evaluate the long-term safety and tolerability of multiple doses of BIIB092 in participants with PSP who participated in CN002003.

- Study 251PP301 is an ongoing Phase 2b randomized, double-blind, placebo-controlled, parallel-group study to evaluate the efficacy and safety of intravenously administered BIIB092 2000 mg versus placebo (2:1 randomization ratio) in participants with PSP.

4.4. Study Rationale

BIIB092 is a humanized immunoglobulin (Ig) G4 monoclonal antibody that has been shown to bind tau at the amino terminus. Although tau is primarily an intracellular protein, a portion of tau is secreted by neurons as N-terminal tau fragments.

BIIB092 has been shown to lower CSF concentrations of N-terminal tau in nonclinical studies, a SAD study in healthy participants (Study CN002001), and a MAD study in participants with PSP (Study CN002003). AD is defined by 2 neuropathologic hallmarks: extracellular neuritic plaques containing Aβ peptide and intraneuronal NFTs composed of hyperphosphorylated tau protein. Based upon the close links between tau pathology, neurodegeneration, and clinical features consistent with AD, coupled with the evidence that tau pathology can spread via neuronal release and uptake of pathological tau species, examination of BIIB092 in the clinical AD setting is strongly supported as a potential disease-modifying therapy. This study will assess the safety and efficacy of BIIB092 in participants with mild cognitive impairment (MCI) due to AD or with mild AD, selected by proof of brain amyloid burden. This study will also help to inform on dose selection in this patient population.

CONFIDENTIAL
The information contained herein may not be used, disclosed, or published without the written consent of Biogen MA Inc.

Rationale for BIIB092 dose selection

The present study is primarily designed to assess the safety and tolerability of IV BIIB092 at doses of 125, 600, and 2000 mg administered once every 4 weeks or 375 mg administered once every 12 weeks versus placebo in participants with MCI due to AD or with mild AD. As a secondary objective, the study will evaluate the efficacy of BIIB092 in slowing cognitive and functional impairment in the study participants, as measured by changes from Baseline in the Clinical Dementia Rating Scale (CDR) - Sum of Boxes (CDR-SB), the primary efficacy endpoint.

[redacted]

[redacted]

[redacted]

[redacted]

Rationale for use of placebo

Placebo is included as a randomized treatment in this study to avoid bias in the evaluation of BIIB092, including the reporting of adverse events (AEs) to address the primary objective. Concomitant therapy specifications for the study will protect participant safety by allowing the continuation of medications for chronic conditions as long as dosage has been stable for 4 weeks prior to the first Screening Visit and the use of therapies for AD if the participant was on a stable dose for at least 8 weeks prior to Screening Visit 1 and is expected to stay on a stable dose while in the study. (See Section 11.4.1 for details on concomitant therapy use.)

4.5. Overall Benefits and Risks Assessment

4.5.1. Overall Benefit

BIIB092 has the potential to slow or stop the spread of tau pathology observed in neurodegenerative diseases such as PSP, AD, and other tauopathies.

In participants with PSP, BIIB092 has been evaluated in 2 completed clinical studies (Studies CN002001 and CN002003) and is currently being evaluated in the ongoing Studies 251PP201 and 251PP301. The first-in-human study of BIIB092 (Study CN002001) was designed as a randomized, double-blind, placebo-controlled, SAD study in healthy participants to characterize the safety, tolerability, PK, PD, and immunogenicity of single doses of BIIB092, ranging from 21 to 4200 mg. Study CN002003, a Phase 1b study, was designed as a randomized, double-blind, placebo-controlled MAD study to characterize the safety, tolerability, PK, PD, and immunogenicity of multiple doses of BIIB092, ranging from 150 to 2100 mg, in participants with PSP. In both of these completed studies, BIIB092 was found to be well tolerated at the doses tested in both healthy participants and participants with PSP. The ongoing Phase 1b study, Study 251PP201, is designed as an open-label extension study to evaluate the long-term safety and tolerability of multiple doses of BIIB092 in participants with PSP who participated in Study CN002003. Several interim analyses of the data being collected in this study have been performed to date. Study 251PP301 is an ongoing Phase 2b study designed as a randomized, double-blind, placebo-controlled, parallel-group study to evaluate the efficacy and safety of IV-administered BIIB092 in participants with PSP.

In participants with AD, this study (Study 251AD201) will evaluate the safety and efficacy of BIIB092.

Based on the PK and PD data from Studies CN002001 and CN002003, the mean concentrations of BIIB092 in CSF increased in a generally dose-proportional manner. A robust and persistent lowering of unbound N-terminal tau was observed following administration of single and multiple doses of BIIB092 in healthy participants and in participants with PSP, consistent with the observations in cynomolgus monkeys.

4.5.2. Potential Risks

The nonclinical studies conducted to date have demonstrated an acceptable safety profile for BIIB092 to support continued use in clinical studies.

As of 25 November 2018, an estimated 486 healthy participants or participants with PSP or AD have been exposed to BIIB092 in clinical studies. The following summary of the safety profile of BIIB092 is based on safety data from the completed Studies CN002001 and CN002003 and the ongoing Study 251PP201. In the SAD study in healthy participants, there were no safety findings of note, and development continued in patients with PSP. In participants with PSP, to date, the most commonly reported AEs were fall, urinary tract infection (UTI), contusion, and headache. Most AEs have been reported as mild or moderate in intensity. Serious AEs (SAEs) have generally been consistent with disease (UTI, respiratory arrest, aspiration pneumonia, and progressive PSP) or are not unexpected in the patient population enrolled in the trials

CONFIDENTIAL
The information contained herein may not be used, disclosed, or published without the written consent of Biogen MA Inc.

(cholecystitis, cancer, fractures, and anemia). There have been 3 deaths, all of which were likely related to underlying disease (respiratory arrest, aspiration pneumonia, and progressive disease). None of the SAEs or deaths were considered related to BIIB092 by Investigators. No safety concerns have been identified from laboratory, vital signs, or electrocardiogram (ECG) assessments. Nonserious infusion reactions have been observed and are an identified risk of BIIB092.

BIIB092 is a humanized IgG4 monoclonal antibody. While a low risk of immunogenicity is suggested by preclinical studies, the risk of immunogenicity in humans is unknown. Immunogenicity is an AE of special interest that is under monitoring in all ongoing studies. No safety data regarding this topic have emerged that would change the safety profile of BIIB092.

In general, BIIB092 has been well tolerated in the clinical study participants. There are no important identified or potential risks in the program. The safety profile is acceptable to continue development.

4.5.3. Summary

Currently available treatments for AD offer modest symptomatic relief, but none has the potential to modify the underlying disease pathology or course of the disease. In addition, no medications have been approved for the treatment of PSP. Therefore, there is a significant unmet need for the development of effective disease-modifying therapies in both PSP and AD.

Based on the PK and PD data from Studies CN002001 and CN002003, the mean concentrations of BIIB092 in CSF increased in a generally dose-proportional manner. A robust and persistent lowering of unbound N-terminal tau was observed following administration of single and multiple doses of BIIB092 in healthy participants and in participants with PSP, consistent with the observations in cynomolgus monkeys.

Based on the safety data from the completed Studies CN002001 and CN002003 and the data from an interim analysis of the ongoing Study 251PP201, BIIB092 was generally well tolerated and demonstrated an acceptable safety profile for continued development.

The overall analysis of potential benefits (based on the robust and persistent lowering of unbound N-terminal tau in healthy participants and in participants with PSP, consistent with cynomolgus monkeys) and risks (available safety data indicating that BIIB092 is generally well tolerated) supports the continued development of BIIB092 in both PSP and AD.

CONFIDENTIAL
The information contained herein may not be used, disclosed, or published without the written consent of Biogen MA Inc.

5. SCHEDULE OF ACTIVITIES

The schedule of study activities is presented in Table 1, Table 2, Table 3, and Table 4.

CONFIDENTIAL
The information contained herein may not be used, disclosed, or published without the written consent of Biogen MA Inc.

Table 1: Schedule of Activities During the Placebo-Controlled Period and the LTE Screening Period

	Baseline Screening Period[1, 2] within 65 days[3] of Day 1			Placebo-Controlled Period[4]																					UV for Change in AD Medication[5]	FUV[6]
**Study Week**					**4**	**8**	**12**	**16**	**20**	**24**	**28**	**32**	**36**	**40**	**44**	**48**	**52**	**56**	**60**	**64**	**68**	**72**	**76 EOT**	**78/ EOS or ET[7]**		**90**
**Study Day**	**S1**	**S2**	**S3[8]**	**1**	**29 ±7**	**57 ± 7**	**85 ±7**	**11 3 ±7**	**14 1 ±7**	**169 ±7**	**19 7 ±7**	**22 5 ±7**	**25 3 ±7**	**28 1 ±7**	**30 9 ±7**	**337 ±7**	**36 5 ±7**	**39 3 ±7**	**42 1 ±7**	**44 9 ±7**	**47 7 ±7**	**50 5 ±7**	**533 ±7**	**547 ±7**		**631 ±7**
Study day infusion[4, 9]				X	X	X	X	X	X	X	X	X	X	X	X	X	X	X	X	X	X	X	X			
Initial screening consent (optional)[10]	X																									
Full informed consent[11]	X																						X[12]	X[12]		
Randomization				X																						
Eligibility criteria	X	X	X	X																			X[13]	X[13]		
NIA-AA criteria review	X																									
Medical history	X	X	X	X																						
Body weight	X			X	X	X	X			X				X			X			X			X	X		X
Height	X																									
Pregnancy test[14]	X			X	X	X	X	X	X	X	X	X	X	X	X	X	X	X	X	X	X	X	X	X		X
Follicle-stimulating hormone[15]	X																									
Alcohol/drug screen	X																									
HbA1c	X																									

	**Baseline Screening Period[1, 2] within 65 days[3] of Day 1**			**Placebo-Controlled Period[4]**																				**UV for Change in AD Medication[5]**	**FUV[6]**
**Study Week**					**4**	**8**	**12**	**16**	**20**	**24**	**28**	**32**	**36**	**40**	**44**	**48**	**52**	**56**	**60**	**64**	**68**	**72**	**76 EOT**	**78/ EOS or ET[7]**		**90**
**Study Day**	**S1**	**S2**	**S3[8]**	**1**	**29 ±7**	**57 ± 7**	**85 ±7**	**113 ±7**	**141 ±7**	**169 ±7**	**197 ±7**	**225 ±7**	**253 ±7**	**281 ±7**	**309 ±7**	**337 ±7**	**365 ±7**	**393 ±7**	**421 ±7**	**449 ±7**	**477 ±7**	**505 ±7**	**533 ±7**	**547 ±7**		**631 ±7**
HIV[16]/hepatitis tests	X																									
[redacted]																										
Physical examination	X			X			X			X						X						X		X		X
Neurological examination	X			X			X			X						X						X		X		X
12-lead paper ECG[18]	X			X			X			X						X						X		X		X
Vital signs[19]	X			X	X	X	X	X	X	X	X	X	X	X	X	X	X	X	X	X	X	X	X	X		X
Hematology/ clinical chemistry/ urinalysis	X			X			X			X						X						X		X		X
Blood sample for anti-BIIB092 Ab[4]				X	X					X						X							X			X
Blood sample for BIIB092 concentration				X	X[20]	X	X[20]	X	X	X[20]	X					X[20]			X				X[20]			X
[redacted]																										

	**Baseline Screening Period[1, 2] within 65 days[3] of Day 1**			**Placebo-Controlled Period[4]**																					**UV for Change in AD Medication[5]**	**FUV[6]**
**Study Week**					**4**	**8**	**12**	**16**	**20**	**24**	**28**	**32**	**36**	**40**	**44**	**48**	**52**	**56**	**60**	**64**	**68**	**72**	**76 EOT**	**78/ EOS or ET[7]**		**90**
**Study Day**	**S1**	**S2**	**S3[8]**	**1**	**29 ±7**	**57 ±7**	**85 ±7**	**113 ±7**	**141 ±7**	**169 ±7**	**197 ±7**	**225 ±7**	**253 ±7**	**281 ±7**	**309 ±7**	**337 ±7**	**365 ±7**	**393 ±7**	**421 ±7**	**449 ±7**	**477 ±7**	**505 ±7**	**533 ±7**	**547 ±7**		**631 ±7**
Blood sample for plasma and serum biomarkers[4]				X				X								X			X				X			
[redacted]																										
Brain MRI[22]		X									X						X							X[23]		
[redacted]																										
CDR[28]	X									X							X							X	X	
NPI-10			X							X							X							X		
ISLR	X																									
ISLT	X		X				X				X			X				X			X			X	X	
DKEFS Category Fluency and Letter Fluency tests	X		X				X				X			X				X			X			X		
DSST	X		X				X				X			X				X			X			X		

	**Baseline Screening Period[1, 2] within 65 days[3] of Day 1**			**Placebo-Controlled Period[4]**																					**UV for Change in AD Medication[5]**	**FUV[6]**
**Study Week**					**4**	**8**	**12**	**16**	**20**	**24**	**28**	**32**	**36**	**40**	**44**	**48**	**52**	**56**	**60**	**64**	**68**	**72**	**76 EOT**	**78/ EOS or ET[7]**		**90**
**Study Day**	**S1**	**S2**	**S3[8]**	**1**	**29 ±7**	**57 ± 7**	**85 ±7**	**113 ±7**	**141 ±7**	**169 ±7**	**197 ±7**	**225 ±7**	**253 ±7**	**281 ±7**	**309 ±7**	**337 ±7**	**365 ±7**	**393 ±7**	**421 ±7**	**449 ±7**	**477 ±7**	**505 ±7**	**533 ±7**	**547 ±7**		**631 ±7**
Trails A	X		X				X				X			X				X			X			X		
eCog (39-item version)			X								X							X						X		
ADAS-Cog 13[29]		X	X							X							X							X	X	
FAQ			X							X							X							X	X	
ADCS-ADL			X							X							X							X	X	
C-SSRS[29]		X	X							X							X							X		
MMSE	X						X			X				X			X				X			X	X	
RUD-Lite			X						X							X					X			X if ET		
ZBI			X						X							X					X			X if ET		
QoL-AD			X						X							X					X			X if ET		
AE reporting				Monitor and record continuously beginning on Study Day 1 at the start of dosing																						
[redacted]																										
SAE reporting			Monitor and record continuously throughout the study																							
Concomitant therapy and procedures reporting			Monitor and record continuously throughout the study																							

	Baseline Screening Period[1, 2] within 65 days[3] of Day 1			Placebo-Controlled Period[4]																					UV for Change in AD Medication[5]	FUV[6]
**Study Week**					**4**	**8**	**12**	**16**	**20**	**24**	**28**	**32**	**36**	**40**	**44**	**48**	**52**	**56**	**60**	**64**	**68**	**72**	**76 EOT**	**78/ EOS or ET[7]**		**90**
**Study Day**	**S1**	**S2**	**S3[8]**	**1**	**29 ±7**	**57 ± 7**	**85 ±7**	**113 ±7**	**141 ±7**	**169 ±7**	**197 ±7**	**225 ±7**	**253 ±7**	**281 ±7**	**309 ±7**	**337 ±7**	**365 ±7**	**393 ±7**	**421 ±7**	**449 ±7**	**477 ±7**	**505 ±7**	**533 ±7**	**547 ±7**		**631 ±7**
Tobacco use status[31]			Monitor and record continuously throughout the study																							

Ab = antibodies; AD = Alzheimer's disease; ADAS-Cog 13 = Alzheimer's Disease Assessment Scale-Cognitive (13 item); ADCS-ADL = Alzheimer's Disease Cooperative Study – Activities of Daily Living; AE = adverse event; [redacted]; CBC = complete blood count; CDR = Clinical Dementia Rating Scale; [redacted]; C-SSRS = Columbia - Suicide Severity Rating Scale; DBP = diastolic blood pressure; DKEFS = Delis-Kaplan Executive Function System; DSST = Digit Symbol Substitution Test; ECG = electrocardiogram; eCog = Everyday Cognition; EOS = end of study; EOT = end of treatment; ET = early termination; FAQ = Functional Activities Questionnaire; FUV = Follow-up Visit; HbA1c = glycosylated hemoglobin; HIV = human immunodeficiency virus; [redacted]; ISLR = International Shopping List Test Delayed Recall; ISLT = International Shopping List Test Immediate Recall; [redacted]; LTE = long-term extension; MMSE = Mini-Mental State Examination; MRI = magnetic resonance imaging; NIA-AA = National Institute on Aging-Alzheimer's Association; NPI-10 = Neuropsychiatric Inventory 10; [redacted]; PK = pharmacokinetic; [redacted]; QoL-AD = Quality of Life in Alzheimer's Disease; [redacted]; RUD-Lite = Resource Utilization in Dementia-Lite Version; SAE = serious adverse event; SBP = systolic blood pressure; Trails A = Trail Making Test Part A; UV = unscheduled visit; ZBI = Zarit Burden Interview

1 The screening process will generally involve up to 3 visits, and most screening procedures will be performed within these designated visits (S1 S3). However additional screening visits may be needed for some procedures, e.g., MRI [redacted] In addition, it may sometimes be necessary for participants to return for repeat assessments (e.g., if the MRI scan does not pass the quality control process). Brain MRI (S2) will only be performed after the participant has met the eligibility criteria and has acceptable laboratory tests from Visit S1. [redacted]

2 The clinical assessments performed during Visit S1 are to be administered in the order specified: 1) MMSE; 2) neuropsychological tests in the following order: ISLT, DSST, Trails A, Category Fluency and Letter Fluency tests from the DKEFS, ISLR; 3) CDR. Note that the Visit S1 results from CDR, ISLT/ISLR, and MMSE will be used to determine participant eligibility; Visit S3 ISLT assessments will provide baseline measures. At Visit S3, the clinical assessments will be administered as follows: 1) neuropsychological tests in the following order: ISLT, DSST, Trails A, Category Fluency and Letter Fluency tests from the DKEFS; 2) ADAS-Cog 13; 3) QoL-AD.

3 It is recommended that all the screening procedures be completed within 65 days; however, the overall Screening Period may be extended up to 90 days in consultation with the Medical Monitor in advance and provided that written documentation is received from the Sponsor or delegate ([redacted]). The following screening assessments should be repeated if the Screening Period is >65 days: confirmation of eligibility criteria,

abbreviated medical history, and physical examination. In addition, some cognitive and /or safety assessments may require repeating depending on the duration of the screening window extension, in discussion with the Medical Monitor.

4 All assessments and blood collection during dosing days will be performed prior to infusion, unless otherwise specified. Doses should be administered at least 21 days apart.

5 Participants should have a UV prior to the initiation of, change in dose, or discontinuation of AD medication. That visit should include assessment of AEs/SAEs and concomitant medications and key clinical assessments, including, at a minimum, the CDR, MMSE, ISLT, ADAS-Cog 13, ADCS-ADL, and FAQ. During the UV, these clinical assessments need not be repeated if performed within the previous 30 days. If the visit for a change in AD medication occurs at the same time as another planned visit, all originally scheduled assessments must also take place as required and without repetition. See Section 11.4.1.2 for details.

6 Participants who complete the Treatment Period and do not enter an extension study are to return to the study site for a Follow-up Safety Visit at Week 90. Participants who discontinue study treatment and withdraw from the study early are to have a Follow-up Safety Visit 14 weeks after the final dose. Participants who discontinue study treatment prematurely are to return to the study site for a Follow-up Safety Visit 14 weeks after receiving the last dose of study treatment. See Section 10.1 and Section 10.3 for details.

7 All participants who discontinue study treatment prematurely will be asked to remain in the study and continue all protocol-specified visits and procedures. These participants should be encouraged to attend at least the Week 24, Week 52, and Week 78 visits, depending on when treatment was discontinued, and undergo all the scheduled procedures. At a minimum, these visits should include assessment of AEs/SAEs and concomitant medications and key clinical assessments, including at least the CDR, MMSE, ISLT, ADAS-Cog 13, ADCS-ADL, and FAQ. Participants who discontinue study treatment prematurely will also be asked to return to the study site for a Follow-up Safety Visit 14 weeks after receiving the last dose of study treatment. See Section 10.1 for details. Participants who withdraw from the study prematurely are to return to the study site for an ET Visit and ET assessments. If the withdrawn participant has discontinued treatment within 3 months of the previous primary efficacy assessment (CDR) and no significant changes in cognitive status are suspected by the Investigator, the clinical efficacy assessments specified in the ET visit are not required; the study site should notify the Sponsor in such cases. An MRI scan should be performed if withdrawal occurs ≥6 months after the previous MRI. Blood for biomarker analysis should be collected if the participant withdraws ≥4 weeks after the previous sample was collected. [redacted] See Section 10.3 for details.

8 Visit S3 all clinical assessments must be scheduled within 7 days before Study Day 1 or performed at Study Day 1 before randomization, [redacted] The overall Screening Period may be extended up to 90 days in consultation with the Medical Monitor in advance and provided that written documentation is received from the Sponsor or delegate ([redacted]).

9 All participants should be observed and monitored by study staff for a minimum of 1 hour after the end of an infusion. Participants who experience an AE or SAE related to BIIB092 infusion should remain at the site or be sent to an inpatient monitoring facility until the Investigator has determined that the event(s) has resolved or do not require further monitoring at the site or in an inpatient setting.

10 Participants and the participants' partners/informants or their legally authorized representatives, in countries where applicable laws allow, may sign this optional form for an initial screening which allows administration of the CDR, MMSE, ISLT/ISLR, DKEFS Category Fluency and Letter Fluency tests, DSST, and Trails A to determine eligibility based on cognitive assessments. The order of assessments is as specified in footnote 2.

11 All participants and the participants' partners/informants or their legally authorized representatives, in countries where applicable laws allow, must sign this full informed consent, including those who have previously signed the optional initial screening consent and have met the eligibility criteria based on cognitive assessments.

[12] All participants and the participants' partners/informants or their legally authorized representatives, in countries where applicable laws allow must reconsent by signing this full informed consent before participating in the general [redacted] during the LTE period.

[13] Only for participants entering the LTE period.

[14] Pregnancy testing is required for women of childbearing potential only; for these participants, a serum pregnancy test is to be performed at Screening and EOS (or ET) Visits, and a urine pregnancy test is to be performed at every dosing visit.

[15] Women who report postmenopausal status at Visit S1 must have a follicle-stimulating hormone test at that visit to confirm that they are not of childbearing potential.

[16] To be performed based upon Investigator assessment of HIV risk factors. The requirement for testing during Screening may be omitted if it is not permitted by local regulations.

[redacted]

[18] Triplicate 12-lead (paper) ECGs will be obtained at each specified timepoint. ECGs are to be performed in triplicate, with each ECG performed about 1 minute apart and before blood draws on appropriate days. In addition, triplicate ECGs will also be performed 1 hour after the end of infusion on Study Day 1 only. Each ECG must be performed after the participant has been resting (supine) for 10 minutes. ECGs will be read by a central reading facility. Clinical significance of potential ECG abnormalities will be determined by the Investigator.

[19] Vital signs will include SBP, DBP, heart rate, body temperature, and respiratory rate and will be measured with the participant supine and after the participant has been resting for at least 10 minutes. In addition, blood pressure and heart rate will be measured after the participant has been standing for ≥2 minutes at the following timepoints: preinfusion and 1 hour (±15 minutes) after the end of infusion on Days 1, 29, 57, and 85. Three separate SBP/DBP readings at least 15 minutes apart will be made at the Screening Visit to determine eligibility. Single vital sign readings will be obtained at other timepoints.

[20] Two samples must be obtained: 1 sample taken before infusion and 1 sample taken at ≤15 minutes after the end of infusion.

[21] The results are not required for randomization.

[22] Brain MRI may include, but will not be limited to, the following sequences: 3D T1 weighted, fluid attenuated inversion recovery, T2* weighted, diffusion weighted imaging, and proton density/T2 weighted MRI. For further details on MRI sequences, please see the procedural manual for MRI. To occur after cognitive testing has concluded for the Screening Period. After randomization, the visit date for this MRI can vary by ±10 days from the specified visit day.

[23] Sites should schedule the Week 78 MRI within 10 days prior to the Week 78 visit where possible to allow for MRI results to be available before participants enter the LTE at Week 80.

[redacted]

CONFIDENTIAL
The information contained herein may not be used, disclosed, or published without the written consent of Biogen MA Inc.

[redacted]

[28]It is recommended that postbaseline CDR assessments be conducted at the same time of day at which the baseline assessment was performed to avoid diurnal variation. The rater who conducts the CDR for a participant/informant cannot complete any other rating scales for that same participant/partner or be the study site coordinator and will be blinded to all other study-related data.

[29]During the Screening Period, ADAS-Cog 13 and C-SSRS are scheduled at Visit S2; however, they may be performed at Visit S1, if more convenient per site and participant's availability. If ADAS-Cog 13 and C-SSRS assessments are performed at Visit S1, all other clinical assessments should be performed before ADAS-Cog 13, followed by C-SSRS.

[redacted]

[31]Participants' tobacco use status will be assessed while collecting information on concomitant therapies.

Table 2: Schedule of Activities for Week 80 Through Week 128 of the Long-Term Extension Period

	Long-Term Extension Period													UV for Change in AD Medication[1]
**Study Week**	**80**	**84**	**88**	**92**	**96**	**100**	**104**	**108**	**112**	**116**	**120**	**124**	**128**	
**Study Days (±7 days)**	**561**	**589**	**617**	**645**	**673**	**701**	**729**	**757**	**785**	**813**	**841**	**869**	**897**	
Study day infusion[2, 3]	X	X	X	X	X	X	X	X	X	X	X	X	X	
Eligibility criteria	X													
Pregnancy test[4]	X	X	X	X	X	X	X	X	X	X	X	X	X	
[redacted]														
Physical examination				X			X						X	
Body weight				X			X						X	
Neurological examination				X			X						X	
12-lead paper ECG[6]							X						X	
Vital signs[7]	X	X	X	X	X	X	X	X	X	X	X	X	X	
Hematology/clinical chemistry/ urinalysis						X						X		
Blood sample for anti-BIIB092 Ab	X						X			X				
Blood sample for BIIB092 concentration	X						X			X				
Blood sample for plasma and serum biomarker										X				
Brain MRI[8]							X							
[redacted]														
CDR[12]							X						X	X

	**Long-Term Extension Period**													**UV for Change in AD Medication**[1]
**Study Week**	**80**	**84**	**88**	**92**	**96**	**100**	**104**	**108**	**112**	**116**	**120**	**124**	**128**	
**Study Days (±7 days)**	**561**	**589**	**617**	**645**	**673**	**701**	**729**	**757**	**785**	**813**	**841**	**869**	**897**	
NPI-10							X						X	
ISLT								X						X
DKEFS Category Fluency and Letter Fluency tests								X						
DSST								X						
Trails A								X						
eCog (39-item version)								X						
ADAS-Cog 13							X						X	X
FAQ							X						X	X
ADCS-ADL							X						X	X
C-SSRS							X						X	
MMSE							X						X	X
RUD-Lite												X		
ZBI												X		
QoL-AD												X		
AE reporting	Monitor and record continuously throughout the LTE period													
[redacted]														
SAE reporting	Monitor and record continuously throughout the LTE period													
Concomitant therapy and procedures reporting	Monitor and record continuously throughout the LTE period													
Tobacco use status[14]	Monitor and record continuously throughout the LTE period													

Ab = antibodies; AD = Alzheimer's disease; ADAS-Cog 13 = Alzheimer's Disease Assessment Scale-Cognitive (13 item); ADCS-ADL = Alzheimer's Disease Cooperative Study – Activities of Daily Living; AE = adverse event; CDR = Clinical Dementia Rating Scale; [redacted]; C-SSRS = Columbia-Suicide Severity Rating Scale; DBP = diastolic blood pressure; DKEFS = Delis-Kaplan Executive Function System; DSST = Digit Symbol Substitution Test; ECG = electrocardiogram; eCog = Everyday Cognition; EOS = end of study; EOT = end of treatment; ET = early termination; FAQ = Functional Activities Questionnaire; [redacted]; ISLT = International Shopping List Test Immediate Recall; [redacted];

LTE = long-term extension; MMSE = Mini-Mental State Examination; MRI = magnetic resonance imaging; NPI-10 = Neuropsychiatric Inventory 10; [redacted]; PK = pharmacokinetics; [redacted]; QoL-AD = Quality of Life in Alzheimer's Disease; RUD-Lite = Resource Utilization in Dementia-Lite Version; SAE = serious adverse event; SBP = systolic blood pressure; Trails A = Trail Making Test Part A; UV = unscheduled visit; ZBI = Zarit Burden Interview.

[1] Participants should have a UV prior to the initiation of, change in dose, or discontinuation of AD medication. That visit should include assessment of AEs/SAEs and concomitant medications and key clinical assessments, including, at a minimum, the CDR, MMSE, ISLT, ADAS-Cog 13, ADCS-ADL, and FAQ. During the UV, these clinical assessments need not be repeated if performed within the previous 30 days. If the visit for a change in AD medication occurs at the same time as another planned visit, all originally scheduled assessments must also take place as required and without repetition. See Section 11.4.1.2 for details.

[2] All participants should be observed and monitored by study staff for a minimum of 1 hour after the end of an infusion. Participants who experience an AE or SAE related to BIIB092 infusion should remain at the site or be sent to an inpatient monitoring facility until the Investigator has determined that the event(s) has resolved or do not require further monitoring at the site or in an inpatient setting.

[3] All assessments and blood collection during dosing days will be performed prior to infusion, unless otherwise specified. Doses should be administered at least 21 days apart.

[4] Pregnancy testing is required for women of childbearing potential only; for these participants, a serum pregnancy test is to be performed at EOS [or ET] Visits, and a urine pregnancy test is to be performed at every dosing visit.

[redacted]

[6] Triplicate 12-lead (paper) ECGs will be obtained at each specified timepoint. ECGs are to be performed in triplicate, with each ECG performed about 1 minute apart and before blood draws on appropriate days. Each ECG must be performed after the participant has been resting (supine) for 10 minutes. ECGs will be read by a central reading facility. Clinical significance of potential ECG abnormalities will be determined by the Investigator.

[7] Vital signs will include SBP, DBP, heart rate, body temperature, and respiratory rate and will be measured with the participant supine and after the participant has been resting for at least 10 minutes. In addition, blood pressure and heart rate will be measured after the participant has been standing for ≥2 minutes at the following timepoints: preinfusion and 1 hour (±15 minutes) after the end of infusion on Weeks 80, 84, 88, and 92. Single vital sign readings will be obtained at other timepoints.

[8] Brain MRI may include, but will not be limited to, the following sequences: 3D T1 weighted, fluid attenuated inversion recovery, T2* weighted, diffusion weighted imaging, and proton density/T2 weighted MRI. For further details on MRI sequences, please see the procedural manual for MRI. During the LTE period, the visit date for the MRI can vary by ±10 days from the specified visit day.

[9] [redacted]

[redacted]

[12] It is recommended that postbaseline CDR assessments be conducted at the same time of day at which the baseline assessment was performed to avoid diurnal variation. The rater who conducts the CDR for a participant/informant cannot complete any other rating scales for that same participant/partner or be the study site coordinator and will be blinded to all other study-related data.

[redacted]

[14] Participants' tobacco use status will be assessed while collecting information on concomitant therapies.

Table 3: Schedule of Activities for Week 132 Through Week 176 of the Long-Term Extension Period

	**Long-Term Extension Period**												**UV for Change in AD Medication[1]**
**Study Week**	**132**	**136**	**140**	**144**	**148**	**152**	**156**	**160**	**164**	**168**	**172**	**176**	
**Study Days (±7 days)**	**925**	**953**	**981**	**1009**	**1037**	**1065**	**1093**	**1121**	**1149**	**1177**	**1205**	**1233**	
Study day infusion[2, 3]	X	X	X	X	X	X	X	X	X	X	X	X	
Pregnancy test[4]	X	X	X	X	X	X	X	X	X	X	X	X	
[redacted]													
Physical examination						X						X	
Body weight						X						X	
Neurological examination						X						X	
12-lead paper ECG[6]						X						X	
Vital signs[7]	X	X	X	X	X	X	X	X	X	X	X	X	
Hematology/clinical chemistry/ urinalysis					X						X		
Blood sample for anti-BIIB092 Ab							X					X	
Blood sample for BIIB092 concentration							X					X	
Blood sample for plasma and serum biomarker							X						
Brain MRI[8]							X						
[redacted]													
CDR[12]						X						X	X
NPI-10						X							
ISLT	X						X						X
DKEFS Category	X						X						

	**Long-Term Extension Period**												**UV for Change in AD Medication**[1]
**Study Week**	**132**	**136**	**140**	**144**	**148**	**152**	**156**	**160**	**164**	**168**	**172**	**176**	
**Study Days (±7 days)**	**925**	**953**	**981**	**1009**	**1037**	**1065**	**1093**	**1121**	**1149**	**1177**	**1205**	**1233**	
Fluency and Letter Fluency tests													
DSST	X						X						
Trails A	X						X						
eCog (39-item version)	X						X						
ADAS-Cog 13						X						X	X
FAQ						X							X
ADCS-ADL						X						X	X
C-SSRS						X						X	
MMSE						X						X	X
RUD-Lite											X		
ZBI											X		
QoL-AD											X		
AE reporting	Monitor and record continuously throughout the LTE period												
[redacted]													
SAE reporting	Monitor and record continuously throughout the LTE period												
Concomitant therapy and procedures reporting	Monitor and record continuously throughout the LTE period												
Tobacco use status[14]	Monitor and record continuously throughout the LTE period												

Ab = antibodies; AD = Alzheimer's disease; ADAS-Cog 13 = Alzheimer's Disease Assessment Scale-Cognitive (13 item); ADCS-ADL = Alzheimer's Disease Cooperative Study – Activities of Daily Living; AE = adverse event; CDR = Clinical Dementia Rating Scale; [redacted]; C-SSRS = Columbia - Suicide Severity Rating Scale; DBP = diastolic blood pressure; DKEFS = Delis-Kaplan Executive Function System; DSST = Digit Symbol Substitution Test; ECG = electrocardiogram; eCog = Everyday Cognition; EOS = end of study; EOT = end of treatment; ET = early termination; FAQ = Functional Activities Questionnaire; [redacted]; ISLT = International Shopping List Test Immediate Recall; [redacted]; LTE = long-term extension; MMSE = Mini-Mental State Examination; MRI = magnetic resonance imaging; NPI-10 = Neuropsychiatric Inventory 10; [redacted]; PK = pharmacokinetic; [redacted]; QoL-AD = Quality of Life in Alzheimer's Disease; RUD-Lite = Resource Utilization in Dementia-Lite Version; SAE = serious adverse event; SBP = systolic blood pressure; Trails A = Trail Making Test Part A; UV = unscheduled visit; ZBI = Zarit Burden Interview.

[1] Participants should have a UV prior to the initiation of, change in dose, or discontinuation of AD medication. That visit should include assessment of AEs/SAEs and concomitant medications and key clinical assessments, including, at a minimum, the CDR, MMSE, ISLT, ADAS-Cog 13, ADCS-ADL, and FAQ. During the UV, these clinical assessments need not be repeated if performed within the previous 30 days. If the visit for a change in AD medication occurs at the same time as another planned visit, all originally scheduled assessments must also take place as required and without repetition. See Section 11.4.1.2 for details.

[2] All participants should be observed and monitored by study staff for a minimum of 1 hour after the end of an infusion. Participants who experience an AE or SAE related to BIIB092 infusion should remain at the site or be sent to an inpatient monitoring facility until the Investigator has determined that the event(s) has resolved or do not require further monitoring at the site or in an inpatient setting.

[3] All assessments and blood collection during dosing days will be performed prior to infusion, unless otherwise specified. Doses should be administered at least 21 days apart.

[4] Pregnancy testing is required for women of childbearing potential only; for these participants, a serum pregnancy test is to be performed at EOS (or ET) Visits, and a urine pregnancy test is to be performed at every dosing visit.

[redacted]

[6] Triplicate 12-lead (paper) ECGs will be obtained at each specified timepoint. ECGs are to be performed in triplicate, with each ECG performed about 1 minute apart and before blood draws on appropriate days. Each ECG must be performed after the participant has been resting (supine) for 10 minutes. ECGs will be read by a central reading facility. Clinical significance of potential ECG abnormalities will be determined by the Investigator.

[7] Vital signs will include SBP, DBP, heart rate, body temperature, and respiratory rate and will be measured with the participant supine and after the participant has been resting for at least 10 minutes. Single vital sign readings will be obtained.

[8] Brain MRI may include, but will not be limited to, the following sequences: 3D T1 weighted, fluid attenuated inversion recovery, T2* weighted, diffusion weighted imaging, and proton density/T2 weighted MRI. For further details on MRI sequences, please see the procedural manual for MRI. During the LTE period, the visit date for the MRI can vary by ±10 days from the specified visit day.

[redacted]

[12] It is recommended that postbaseline CDR assessments be conducted at the same time of day at which the baseline assessment was performed to avoid diurnal variation. The rater who conducts the CDR for a participant/informant cannot complete any other rating scales for that same participant/partner or be the study site coordinator and will be blinded to all other study-related data.

[redacted]

[14]Participants' tobacco use status will be assessed while collecting information on concomitant therapies.

Table 4: Schedule of Activities for Week 180 Through Week 238 or Follow-Up Visit of the Long-Term Extension Period

	**Long-Term Extension Period**														**UV for Change in AD Medication**[1]
**Study Week**	**180**	**184**	**188**	**192**	**196**	**200**	**204**	**208**	**212**	**216**	**220**	**224/ EOT**	**226/ EOS or ET**[2]	**238/FUV (14 weeks After Last Dose)**[3]	
**Study Days (±7 days)**	**1261**	**1289**	**1317**	**1345**	**1373**	**1401**	**1429**	**1457**	**1485**	**1513**	**1541**	**1569**	**1583**	**1667**	
Study day infusion[4, 5]	X	X	X	X	X	X	X	X	X	X	X	X			
Pregnancy test[6]	X	X	X	X	X	X	X	X	X	X	X	X	X	X	
[redacted]															
Physical examination						X							X	X	
Body weight						X							X	X	
Neurological examination						X							X	X	
12-lead paper ECG[8]						X							X	X	
Vital signs[9]	X	X	X	X	X	X	X	X	X	X	X	X	X	X	
Hematology/clinical chemistry/ urinalysis					X						X		X	X	
Blood sample for anti-BIIB092 Ab					X							X		X	
Blood sample for BIIB092 concentration					X							X		X	
Blood sample for plasma and serum biomarker					X							X			
Brain MRI[10]													X		
[redacted]															
CDR[14]						X							X		X
NPI-10						X							X		
ISLT							X						X		X
DKEFS Category Fluency and Letter Fluency tests							X						X		

CONFIDENTIAL
The information contained herein may not be used, disclosed, or published without the written consent of Biogen MA Inc.

	**Long-Term Extension Period**														**UV for Change in AD Medication**[1]
**Study Week**	**180**	**184**	**188**	**192**	**196**	**200**	**204**	**208**	**212**	**216**	**220**	**224/ EOT**	**226/ EOS or ET**[2]	**238/FUV (14 weeks After Last Dose)**[3]	
**Study Days (±7 days)**	**1261**	**1289**	**1317**	**1345**	**1373**	**1401**	**1429**	**1457**	**1485**	**1513**	**1541**	**1569**	**1583**	**1667**	
DSST							X						X		
Trails A							X						X		
eCog (39-item version)							X						X		
ADAS-Cog 13						X							X		X
FAQ						X							X		X
ADCS-ADL						X							X		X
C-SSRS						X							X		
MMSE						X							X		X
RUD-Lite													X		
ZBI													X		
QoL-AD													X		
AE reporting	Monitor and record continuously throughout the LTE period														
[redacted]															
SAE reporting	Monitor and record continuously throughout the LTE period														
Concomitant therapy and procedures reporting	Monitor and record continuously throughout the LTE period														
Tobacco use status[16]	Monitor and record continuously throughout the LTE period														

Ab = antibodies; AD = Alzheimer's disease; ADAS-Cog 13 = Alzheimer's Disease Assessment Scale-Cognitive (13 item); ADCS-ADL = Alzheimer's Disease Cooperative Study – Activities of Daily Living; AE = adverse event; CDR = Clinical Dementia Rating Scale; [redacted]; C-SSRS = Columbia-Suicide Severity Rating Scale; DBP = diastolic blood pressure; DKEFS = Delis-Kaplan Executive Function System; DSST = Digit Symbol Substitution Test; ECG = electrocardiogram; eCog = Everyday Cognition; EOS = End of Study; EOT = End of Treatment; ET = early termination; FAQ = Functional Activities Questionnaire; FUV = Follow-up Visit; [redacted]; ISLT = International Shopping List Test Immediate Recall; [redacted]; LTE = long-term extension; MMSE = Mini-Mental State Examination; MRI = magnetic resonance imaging; NPI-10 = Neuropsychiatric Inventory 10; [redacted]; PK = pharmacokinetic; [redacted]; QoL-AD = Quality of Life in Alzheimer's Disease; RUD-Lite = Resource Utilization in Dementia-Lite Version; SAE = serious adverse event; SBP = systolic blood pressure; Trails A = Trail Making Test Part A; UV = unscheduled visit; ZBI = Zarit Burden Interview.

CONFIDENTIAL
The information contained herein may not be used, disclosed, or published without the written consent of Biogen MA Inc.

[1] Participants should have a UV prior to the initiation of, change in dose, or discontinuation of AD medication. That visit should include assessment of AEs/SAEs and concomitant medications and key clinical assessments, including, at a minimum, the CDR, MMSE, ISLT, ADAS-Cog 13, ADCS-ADL, and FAQ. During the UV, these clinical assessments need not be repeated if performed within the previous 30 days. If the visit for a change in AD medication occurs at the same time as another planned visit, all originally scheduled assessments must also take place as required and without repetition. See Section 11.4.1.2 for details.

[2] All participants who discontinue study treatment prematurely will be asked to remain in the study and continue all protocol-specified visits and procedures. These participants should be encouraged to attend at least the Week 80, 104, 116, 128, 152, 156, 176, 196, 200, 224, and 226/EOS Visits, depending on when treatment was discontinued, and undergo all the scheduled procedures. At a minimum, these visits should include assessment of AEs/SAEs and concomitant medications and key clinical assessments, including at least the CDR, MMSE, ISLT, ADAS-Cog 13, ADCS-ADL, and FAQ. Participants who discontinue study treatment prematurely will also be asked to return to the study site for a Follow-up Safety Visit 14 weeks after receiving the last dose of study treatment. See Section 10.1 for details. Participants who withdraw from the study prematurely are to return to the study site for an ET Visit and ET assessments. If the withdrawn participant has discontinued treatment within 3 months of the previous primary efficacy assessment (CDR) and no significant changes in cognitive status are suspected by the Investigator, the clinical efficacy assessments specified in the ET Visit are not required; the study site should notify the Sponsor in such cases. An MRI scan should be performed if withdrawal occurs ≥6 months after the previous MRI. Blood for biomarker analysis should be collected if the participant withdraws ≥4 weeks after the previous sample was collected. [redacted] ee Section 10.3 for details.

[3] Participants who discontinue study treatment and withdraw from the study early are to have a Follow-up Safety Visit (FUV) 14 weeks after the final dose. Participants who discontinue study treatment prematurely are to return to the study site for a Follow-up Safety Visit 14 weeks after receiving the last dose of study treatment. See Section 10.1 and Section 10.3 for details.

[4] All participants should be observed and monitored by study staff for a minimum of 1 hour after the end of an infusion. Participants who experience an AE or SAE related to BIIB092 infusion should remain at the site or be sent to an inpatient monitoring facility until the Investigator has determined that the event(s) has resolved or do not require further monitoring at the site or in an inpatient setting.

[5] All assessments and blood collection during dosing days will be performed prior to infusion, unless otherwise specified. Doses should be administered at least 21 days apart.

[6] Pregnancy testing is required for women of childbearing potential only; for these participants, a serum pregnancy test is to be performed at EOS (or ET) Visits, and a urine pregnancy test is to be performed at every dosing visit.

[redacted]

[8] Triplicate 12-lead (paper) ECGs will be obtained at each specified timepoint. ECGs are to be performed in triplicate, with each ECG performed about 1 minute apart and before blood draws on appropriate days. Each ECG must be performed after the participant has been resting (supine) for 10 minutes. ECGs will be read by a central reading facility. Clinical significance of potential ECG abnormalities will be determined by the Investigator.

[9] Vital signs will include SBP, DBP, heart rate, body temperature, and respiratory rate and will be measured with the participant supine and after the participant has been resting for at least 10 minutes. Single vital sign readings will be obtained.

CONFIDENTIAL
The information contained herein may not be used, disclosed, or published without the written consent of Biogen MA Inc.

[10]Brain MRI may include, but will not be limited to, the following sequences: 3D T1 weighted, fluid attenuated inversion recovery, T2* weighted, diffusion weighted imaging, and proton density/T2 weighted MRI. For further details on MRI sequences, please see the procedural manual for MRI. During the LTE period, the visit date for the MRI can vary by ±10 days from the specified visit day.

[redacted]

[14]It is recommended that postbaseline CDR assessments be conducted at the same time of day at which the baseline assessment was performed to avoid diurnal variation. The rater who conducts the CDR for a participant/informant cannot complete any other rating scales for that same participant/partner or be the study site coordinator and will be blinded to all other study-related data.

[redacted]

[16]Participants' tobacco use status will be assessed while collecting information on concomitant therapies.

6. STUDY OBJECTIVES AND ENDPOINTS

**Placebo-Controlled Period Objectives and Endpoints**	
**Primary Objective**	**Primary Endpoint**
To evaluate the safety and tolerability of BIIB092 in participants with MCI due to AD or with mild AD	Incidence of AEs and SAEs during the placebo-controlled period
**Secondary Objectives**	**Secondary Endpoints**
To evaluate the efficacy of multiple doses of BIIB092 in slowing cognitive and functional impairment in participants with MCI due to AD or with mild AD. This is the primary efficacy objective	Change from Baseline over time at Week 78 on the CDR-SB. This is the primary efficacy endpoint
To evaluate the immunogenicity of BIIB092 after multiple doses in participants with MCI due to AD or with mild AD	Incidence of anti-BIIB092 antibodies in serum over time up to Week 90
**Exploratory Objectives**	**Exploratory Endpoints**
To assess the effect of BIIB092 on the clinical progression of AD	Changes from Baseline over time up to Week 78 on the Mini-Mental State Examination (MMSE), International Shopping List Test Immediate Recall (ISLT), Category Fluency and Letter Fluency tests from the Delis-Kaplan Executive Function System (DKEFS), Digit Symbol Substitution Test (DSST), Trail Making Test, Part A (Trails A), Everyday Cognition (eCog), Alzheimer's Disease Cooperative Study-Activities of Daily Living (ADCS-ADL), Functional Activities Questionnaire (FAQ), Alzheimer's Disease Assessment Scale-Cognitive (ADAS-Cog 13 [13 item]), and Neuropsychiatric Inventory-10 (NPI-10)
To assess the effect of BIIB092 on changes in caregiver burden and participant quality of life	Changes from Baseline over time up to Week 68 on the Zarit Burden Interview (ZBI) and Quality of Life for Alzheimer's Disease (QoL-AD)

CONFIDENTIAL
The information contained herein may not be used, disclosed, or published without the written consent of Biogen MA Inc.

To assess the effect of BIIB092 on resource utilization	Resource Utilization in Dementia-Lite Version (RUD-Lite) results over time up to Week 68
To assess BIIB092 PK in serum in participants with MCI due to AD or with mild AD	Trough serum BIIB092 concentrations and end-of-infusion serum BIIB092 concentrations from the samples collected at the visits indicated in the Schedule of Activities
[redacted]	[redacted]
[redacted]	[redacted]
To assess the effect of BIIB092 on biomarkers in blood	Changes from Baseline over time up to Week 76 on blood levels of disease-related biomarkers, including but not limited to, tau and other markers of neurodegenerative disease
[redacted]	[redacted]
[redacted]	[redacted]
To assess the effect of BIIB092 on brain structure	Changes from Baseline over time up to Week 78 on MRI morphometric measures, including volume and cortical thickness of certain brain areas

CONFIDENTIAL
The information contained herein may not be used, disclosed, or published without the written consent of Biogen MA Inc.

**Long-Term Extension Period Objectives and Endpoints**	
**Primary Objective**	**Primary Endpoint**
To evaluate the long-term safety and tolerability of BIIB092 in participants with MCI due to AD or with mild AD	Incidence of AEs and SAEs over the placebo-controlled period and long-term extension (LTE) period of the study
**Exploratory Objectives**	**Exploratory Endpoints**
To further evaluate the immunogenicity of BIIB092 after multiple doses in participants with MCI due to AD or with mild AD	Incidence of anti-BIIB092 antibodies in serum over time up to Week 238
To further assess the effect of BIIB092 on disease progression as measured by fluid and radiological biomarkers, as well as additional clinical and health outcomes	• Changes over the placebo-controlled period and LTE period on the CDR-SB, MMSE, ISLT, Category Fluency and Letter Fluency tests from the DKEFS, DSST, Trails A, eCog, ADCS-ADL, FAQ, ADAS-Cog 13 (13-item), and NPI-10<br>• Changes over the placebo-controlled period and LTE period on the ZBI, QoL-AD, and RUD-Lite<br>• [redacted]<br>• Changes over the placebo-controlled period and LTE period on [redacted] MRI brain morphometric measures
To assess BIIB092 PK in serum [redacted] in participants with MCI due to AD or with mild AD	• Trough serum BIIB092 concentrations from serum samples collected at the visits indicated in the Schedule of Activities<br>• [redacted]

This clinical study will collect samples that, under separate optional consent, may be used for future scientific and genetic research. Specific objectives related to this future research have not been determined.

7. STUDY DESIGN

See Figure 1 for a schematic of the study design.

7.1. Study Overview

This is a Phase 2, randomized, double-blind, placebo-controlled study of BIIB092 in participants aged 50 to 80 years inclusive, with MCI due to AD or mild AD according to National Institute on Aging-Alzheimer's Association criteria (NIA-AA) [McKhann 2011]. Participants must have [redacted] a CDR Memory Box score of ≥0.5.

The study will be conducted in a parallel-group design, with 3 BIIB092 dose groups (including 4 BIIB092 doses) and a placebo group. Approximately 528 participants were planned to be randomized across approximately 100 study sites globally. Due to fast recruitment, the study was over-enrolled and 654 participants have been randomized. Participants will be stratified by [redacted], region, baseline disease stage (MCI or mild AD), and baseline AD medication use. [redacted]

The study consists of a double-blind, placebo-controlled period and a dose-blinded LTE period. The total duration for participants who complete the placebo-controlled period and do not enter the LTE period will be approximately 99 weeks. For participants who complete both the placebo-controlled period and the LTE period, the total study duration will be approximately 247 weeks. The placebo-controlled period comprises a Screening Period of approximately 9 weeks (65 days), a Treatment Period of 76 weeks, and for participants not entering the LTE period, an End-of-Study (EOS) Visit at Week 78 and a Follow-up Safety Visit at Week 90, or approximately 14 weeks after the last dose of study treatment. However, the Screening Period for the placebo-controlled period may be extended up to 90 days in consultation with the Medical Monitor in advance and provided that written documentation is received from the Sponsor or delegate ([redacted]). At the end of the placebo-controlled period, participants who meet the LTE entry criteria may enter the dose-blinded LTE period. The LTE period comprises an LTE Screening Period of approximately 4 weeks starting at Week 76, a 144-week Treatment Period, an EOS Visit at Week 226, and a Follow-up Safety Visit at Week 238, or approximately 14 weeks after the last dose of study treatment.

Randomized participants will receive 1 of the following study treatments by IV infusion every 4 weeks, starting on Study Day 1 during the placebo-controlled period:

- low-dose BIIB092 - 125 mg once every 4 weeks or 375 mg once every 12 weeks and placebo at the other 4-week dosing visits to maintain the treatment blind (88 participants planned in total, 44 per regimen)
- medium-dose BIIB092 - 600 mg once every 4 weeks (88 participants planned)
- high-dose BIIB092 - 2000 mg once every 4 weeks (176 participants planned)
- placebo (176 participants planned)

Overall, participants will have a 2:1 chance of being randomized to BIIB092 or to placebo during the placebo-controlled period.

During the LTE period, participants who were randomized to receive BIIB092 during the placebo-controlled period will continue to receive BIIB092 at the dose they were randomly assigned. Participants receiving placebo during the placebo-controlled period will receive BIIB092 at the high dose (2000 mg) once every 4 weeks during the LTE period. The BIIB092 dose concentration (i.e. low, medium, or high) to which participants are assigned to receive during LTE period may be changed based on emerging data from the BIIB092 clinical development program.

Throughout the study, all participants should be observed and monitored by study staff for a minimum of 1 hour after the end of an infusion. Participants who experience an AE or SAE related to BIIB092 infusion should remain at the site or be sent to an inpatient monitoring facility until the Investigator has determined that the event(s) has resolved or do not require further monitoring at the site or in an inpatient setting.

Investigators, study staff (except for a designated Pharmacist/Technician), and study participants and their families, caregivers, and legal representatives will be blinded to the participants' randomized treatment assignments and, during the LTE period, the BIIB092 dose.

[redacted]

The schedule of study assessments is presented in Section 5.

Figure 1: Study Design

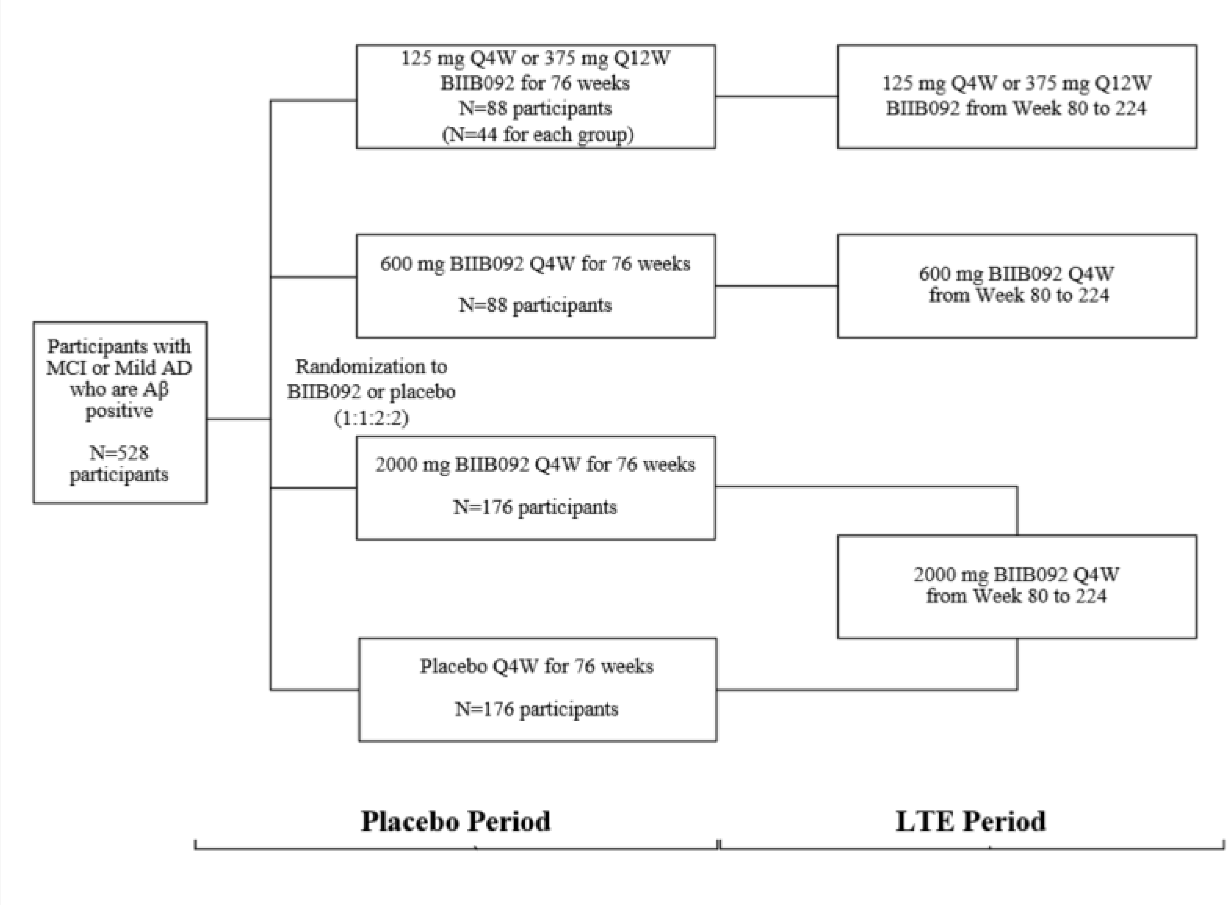

Aβ = amyloid beta; AD = Alzheimer's disease; EOS = End of Study; LTE = long-term extension; MCI = mild cognitive impairment; N = planned number of participants; Q4W = once every 4 weeks; Q12W = once every 12 weeks

Note: Overall, participants will have a 2:1 chance of being randomized to BIIB092 versus placebo during the placebo-controlled period, and all participants will receive BIIB092 during the dose-blinded LTE period.
Note: The study comprises a double-blind, placebo-controlled period and an LTE period. The total duration for participants who complete the placebo-controlled period and do not enter the LTE period will be approximately 99 weeks. The total study duration for participants who complete both the placebo-controlled period and the LTE period will be approximately 247 weeks. The double-blind placebo-controlled period comprises a Screening Period of approximately 9 weeks (65 days), a Treatment Period of 76 weeks, and for participants not entering the LTE period, an EOS Visit at Week 78 and a Follow-up Safety Visit at Week 90, or approximately 14 weeks after the last dose of study treatment. The LTE period comprises an LTE Screening Period of approximately 4 weeks starting at Week 76, a 144-week Treatment Period starting at Week 80, an EOS Visit at Week 226, and a Follow-up Safety Visit at Week 238, or approximately 14 weeks after the last dose of study treatment.

7.2. Study Duration for Participants

The study consists of a double-blind, placebo-controlled period and a dose-blinded LTE period. The total duration for participants who complete the placebo-controlled period and do not enter the LTE period will be approximately 99 weeks. The total study duration for participants who

complete both the placebo-controlled period and the LTE period will be approximately 247 weeks.

Placebo-Controlled Period

Participation in the double-blind, placebo-controlled period will be approximately 99 weeks, which includes the Screening Period of approximately 9 weeks, the 76-week Treatment Period, the EOS Visit at Week 78, and for participants not entering the LTE period, a Follow-up Safety Visit at Week 90, or approximately 14 weeks after the last dose of study treatment (see Table 1). It is recommended that all the screening procedures be completed within 65 days; however, the overall Screening Period may be extended up to 90 days in consultation with the Medical Monitor in advance and provided that written documentation is received from the Sponsor or delegate ([redacted]).

Participants will have approximately 25 outpatient clinic visits:

- Participant eligibility will be determined at up to 3 visits during the Screening Period (Screening Visits 1, 2, and 3).
- On Study Day 1, eligible participants will be randomized, have scheduled assessments per the Schedule of Activities (Table 1), and receive the first infusion of randomized study treatment.
- Participants will return to the clinic at 4-week intervals during the Treatment Period for infusions of study treatment and study assessments (Weeks 4, 8, 12, 16, 20, 24, 28, 32, 36, 40, 44, 48, 52, 56, 60, 64, 68, 72, and 76). The last dose of study treatment will be administered at Week 76 (End-of-Treatment Visit).
- For a participant who completes the Treatment Period at Week 76, there will be an EOS Visit at Week 78 for final study assessments of the placebo-controlled period. A Follow-up Safety Visit will occur at Week 90 (14 weeks after the last dose of study treatment is administered), unless the participant elects to enter an extension study following the Week 78 Visit. Final visit scheduling and procedures are described in Section 10.1 for participants who discontinue study treatment prematurely but remain in the study and in Section 10.3 for participants who withdraw from the study.

All participants should be observed and monitored by study staff for a minimum of 1 hour after the end of an infusion. Participants who experience an AE or SAE related to BIIB092 infusion should remain at the site or be sent to an inpatient monitoring facility until the Investigator has determined that the event(s) has resolved or do not require further monitoring at the site or in an inpatient setting.

CONFIDENTIAL
The information contained herein may not be used, disclosed, or published without the written consent of Biogen MA Inc.

Long-Term Extension Period

Participants who meet the LTE inclusion and exclusion criteria will be eligible to enter the dose-blinded LTE period, which includes the LTE Screening Period of approximately 4 weeks starting at Week 76, the 144-week Treatment Period starting at Week 80, the EOS Visit at Week 226, and the Follow-up Visit at Week 238 (see Table 1, Table 2, Table 3, and Table 4).

Participants will have approximately 39 outpatient clinic visits during the LTE period:

- Participant eligibility will be determined at Week 76 or Week 78 of the placebo-controlled period and confirmed at Week 80 of the LTE period.
- At the Week 80 Visit, eligible participants will have scheduled assessments per the Schedule of Activities (Table 2) and receive the first infusion of BIIB092 during the LTE period. All participants receiving placebo in the placebo-controlled period will receive BIIB092 at the high dose (2000 mg) once every 4 weeks during the LTE period.
- Participants will return to the clinic at 4-week intervals during the Treatment Period for infusions of study treatment and study assessments (Table 2, Table 3, and Table 4).
- The last dose of study treatment will be administered at Week 224 (End-of-Treatment Visit). The EOS Visit will occur at Week 226 and the Follow-up Visit at Week 238.

All participants should be observed and monitored by study staff for a minimum of 1 hour after the end of an infusion. Participants who experience an AE or SAE related to BIIB092 infusion should remain at the site or be sent to an inpatient monitoring facility until the Investigator has determined that the event(s) has resolved or do not require further monitoring at the site or in an inpatient setting.

See Section 5 for the details of the activities to be conducted at each visit.

[redacted]

- [redacted]

[redacted]

[redacted]

[redacted]

[redacted]

[redacted] [redacted]

7.4. Study Stopping Rules

Dosing may be terminated by the Sponsor at the recommendation of the independent Data Monitoring Committee (DMC), based exclusively on safety and tolerability data or following futility analysis, or at the discretion of the Sponsor; therefore, there are no study-specific stopping rules defined in this protocol.

Biogen will notify Investigators when the study is to be placed on hold, completed, or terminated.

7.5. End of Study

The end of study is last participant, last visit.

CONFIDENTIAL
The information contained herein may not be used, disclosed, or published without the written consent of Biogen MA Inc.

8. SELECTION OF PARTICIPANTS

8.1. Inclusion Criteria

To be eligible to participate in this study, candidates must meet the following eligibility criteria at Screening, or at the timepoint specified in the individual eligibility criterion listed:

1. Ability of the participant to understand the purpose and risks of the study and provide signed and dated informed consent and authorization to use confidential health information in accordance with national and local participant privacy regulations. In countries where applicable laws allow, the participant's informant/study partner and/or legally authorized representative may provide informed consent in lieu of the participant's signature.

2. Age 50 to 80 years old, inclusive, at the time of informed consent.

3. All women of childbearing potential and all men with female partners of childbearing potential must practice highly effective contraception during the study and for 6 months (24 weeks) after their last dose of study treatment. For further details of contraceptive requirements for this study, see Section 15.5. Female participants should not become pregnant during the study and for 6 months (24 weeks) after their last dose of study treatment. It is recommended that male participants should not father children during their participation in the study and for 6 months (24 weeks) after their last dose of study treatment.

4. Must have a gradual and progressive change in memory function over more than 6 months, reported by the participant and/or his/her informant/study partner.

5. Must meet all of the clinical criteria for MCI due to AD or mild AD according to the NIA-AA [McKhann 2011], and in addition must have the following at Screening Visit 1:

   - ISLT or ISLR score 1 SD below the age-adjusted normative mean

   - CDR global score of 0.5 for MCI due to AD or 0.5 or 1 for mild AD

   - MMSE score of 22 to 30 (inclusive)

   - CDR Memory Box score of ≥0.5

6. Apart from a clinical diagnosis of MCI due to AD or mild AD, the participant must be in good health as determined by the Investigator, based on medical history and screening assessments.

7. [redacted]

8. Body weight ≥43 kg (95 lbs) and ≤120 kg (265 lbs).

9. Must have 1 informant/study partner who, in the Investigator's judgment, has frequent and sufficient contact with the participant (at least 10 hours/week) as to be able to provide accurate information about the participant's cognitive and functional abilities. The study partner must agree to accompany the participant to clinic visits and/or be available by phone at designated times to provide information to the Investigator and study staff about the participant (and to attend in-person clinic visits that require partner input for scale completion) and must agree to monitor the participant's administration of any prescribed medications. A study partner should be available for the duration of the study, and the participation of the same study partner for the duration of the study is encouraged. The study partner must be literate and give informed consent.

10. [redacted]

    [redacted]

    [redacted]

8.2. Exclusion Criteria

Candidates will be excluded from study entry if any of the following exclusion criteria exist at Screening, or at the timepoint specified in the individual criterion listed:

Medical history

1. History of, or positive test result at Screening Visit 1 for, human immunodeficiency virus (HIV). HIV testing may be completed based upon Investigator assessment of HIV risk factors. The requirement for testing during Screening may be omitted if it is not permitted by local regulations.

2. Current hepatitis C infection (defined as positive hepatitis C virus [HCV] antibody and detectable HCV ribonucleic acid [RNA]). Participants with positive HCV antibody and undetectable HCV RNA are eligible to participate in the study (United States Centers for Disease Control and Prevention).

3. Current hepatitis B infection (defined as positive for hepatitis B surface antigen [HBsAg] and/or total hepatitis B core antibody [anti-HBc]). Participants with immunity to hepatitis B from previous natural infection (defined as negative HBsAg, positive anti-HBc, and positive hepatitis B surface antibody [anti-HBs]) or vaccination (defined as negative HBsAg, negative anti-HBc, and positive anti-HBs) are eligible to participate in the study.

4. Any medical or neurological/neurodegenerative condition (other than AD) that, in the opinion of the Investigator, might be a contributing cause to the participant's cognitive impairment (e.g., current history of substance abuse, uncontrolled vitamin B12 deficiency or abnormal thyroid function, stroke or other cerebrovascular condition, Parkinson's disease, Lewy body dementia, or frontotemporal dementia), or could lead to discontinuation, lack of compliance, interference with study assessments, or safety concerns.

5. History of seizures within 10 years prior to Screening Visit 1 or history of epileptic syndrome (except for history of febrile seizures in childhood).

6. History within 5 years prior to Screening Visit 1 of a serious infectious disease affecting the brain (including neurosyphilis, Lyme disease, meningitis, or encephalitis), or severe head trauma, including concussions, that may have resulted in a protracted loss of consciousness.

7. Presence of clinically significant and/or unstable psychiatric illness, in the Investigator's opinion (e.g., bipolar affective disorder), within the 6 months prior to Screening Visit 1.

8. Any documented prior history of chronic schizophrenia.

9. History of long-term major depression or bipolar affective disorder with an active episode in the past 5 years.

10. Transient ischemic attack or stroke or any unexplained loss of consciousness within 1 year prior to Screening Visit 1.

11. Brain MRI performed at Screening Visit 2 (centrally read) that shows evidence of any of the following:

    - Acute or subacute hemorrhage.

    - Prior macrohemorrhage (defined as >1 cm in diameter on T2* sequence) unless it can be documented that the finding is not due to an underlying structural or vascular abnormality (i.e., finding does not suggest participant is at risk of recurrent hemorrhage).

    - More than 5 microhemorrhages (defined as ≤1 cm in diameter on T2* sequence).

CONFIDENTIAL
The information contained herein may not be used, disclosed, or published without the written consent of Biogen MA Inc.

- Cortical infarct (including cerebellar infarct) [defined as >1.5 cm in diameter] or any infarct in the hippocampus.
- >2 lacunar infarcts (defined as ≤1.5 cm in diameter).
- Superficial siderosis in >1 zone.
- Superficial siderosis >1 cm^3 in any zone.
- Diffuse white matter disease as defined by a score of 3 on the Age-Related White Matter Changes scale [Wahlund 2001].
- Any finding that, in the opinion of the Investigator, might be a contributing cause of the participant's dementia, might pose a risk to the participant, or might prevent a satisfactory MRI assessment.

12. History of bleeding disorder or predisposing conditions, blood clotting, or clinically significant abnormal results on coagulation profile at Screening, as determined by the Investigator.
13. Presence of diabetes mellitus that, in the judgment of the Investigator, is not controlled or adequately managed.
14. History of unstable angina, myocardial infarction, chronic heart failure (New York Heart Association Class III or IV), or clinically significant conduction abnormalities (e.g., unstable atrial fibrillation) within 1 year prior to Screening Visit 1.
15. Clinically significant 12-lead ECG abnormalities, as read by the central reading facility and determined by the Investigator.
16. History of severe allergic or anaphylactic reactions.
17. Known allergy to BIIB092 or a history of hypersensitivity to any of the inactive ingredients in the drug product (see the Investigator's Brochure for information on the BIIB092 clinical formulation).
18. Any major surgery within 12 weeks of Screening Visit 1 or during the Screening Period.
19. Uncontrolled hypertension defined as: an average of 3 systolic blood pressure (SBP)/diastolic blood pressure (DBP) readings >165/100 mm Hg at Screening (blood pressure measurements exceeding these limits may be repeated as warranted by the Investigator, but values must be within the specified limits for the participant to be eligible for the study), or persistent SBP/DBP readings >180/100 mm Hg within 12 weeks prior to randomization (Study Day 1) that, in the opinion of the Investigator, are indicative of chronic uncontrolled hypertension.

CONFIDENTIAL
The information contained herein may not be used, disclosed, or published without the written consent of Biogen MA Inc.

20. History of premalignant or malignant disease. Exceptions to premalignant disease exclusions may be made after discussion with the Sponsor. The following exceptions may be made for malignant disease exclusions after discussion with the Sponsor:

    - Participants with cancers in remission ≥5 years prior to Screening Visit 1.

    - Participants with a history of excised or treated basal cell or squamous carcinoma of the skin.

    - Participants with localized prostate cancer with treatment cycles that completed at least 6 months prior to Screening Visit 1.

21. Indication of impaired liver function as shown by an abnormal liver function profile at Screening (e.g., repeated values of aspartate aminotransferase [AST] and alanine aminotransferase [ALT] ≥2 × the upper limit of normal [ULN]).

22. Indication of impaired renal function at Screening (e.g., repeated values of creatinine and blood urea nitrogen [BUN] ≥1.5 × ULN or estimated glomerular filtration rate <45 mL/minute/1.73 m^2 and corroborating medical history and physical examination).

23. History or evidence of an autoimmune disorder considered clinically significant by the Investigator or requiring long-term use of systemic corticosteroids or other immunosuppressants.

24. History of any clinically significant cardiac, endocrinologic, hematologic, hepatic, immunological, metabolic, urologic, pulmonary, neurologic, dermatologic, psychiatric, and renal, or other major disease, as determined by the Investigator.

25. Recent history (within 1 year of Screening Visit 1) of alcohol or substance abuse as determined by the Investigator, a positive urine drug (due to nonprescription drug) or alcohol test at Screening, or use of cannabinoids (prescription or recreational).

26. Clinically significant systemic illness or serious infection (e.g., pneumonia, septicemia) within 30 days prior to or during the Screening Period.

Medications

27. Use of allowed medications for chronic conditions at doses that have not been stable for at least 4 weeks prior to Screening Visit 1and during the Screening Period up to Study Day 1.

28. Use of AD medications (including but not limited to donepezil, rivastigmine, galantamine, tacrine, and memantine) at doses that have not been stable for at least 8 weeks prior to Screening Visit 1 and during the Screening Period up to Study Day 1.

29. Use of any medications that, in the opinion of the Investigator, may contribute to cognitive impairment, put the participant at higher risk for AEs, or impair the participant's ability to perform cognitive testing or complete study procedures.

30. Use of the following medications:

    - Long-acting benzodiazepines (e.g., valium), except for sedation prior to MRI scans for those participants requiring sedation and should not be administered within 24 hours prior to cognitive testing.

    - Short/medium-acting benzodiazepines (e.g., alprazolam, lorazepam, oxazepam, temazepam) except if used chronically for sleep and on a stable dose for 8 weeks prior to Screening Visit 1 and during the Screening Period up to Study Day 1, or used short term on an as-needed basis. May not be taken within 12 hours prior to cognitive testing.

    - Sedating antihistamines if taken within 12 hours prior to cognitive testing. Nonsedating antihistamines (e.g., fexofenadine, cetirizine) are allowed.

    - Anticonvulsants that have significant effects on cognition per the Investigator's opinion and/or anticonvulsants used for treatment of seizures. Anticonvulsants with limited cognitive effects, such as lamotrigine, pregabalin, levetiracetam for treatment of pain, and other non-epilepsy indications are allowed if, in the opinion of the Investigator, they are not producing sedation or contributing to cognitive impairment. The participant must have been on a stable dose for 8 weeks prior to Screening Visit 1 and during the Screening Period up to Study Day 1.

    - Antidepressants that may, in the Investigator's opinion, affect the participant's cognition (e.g., tricyclic antidepressants). Mild depression or depressive mood arising in the context of AD are not criteria for exclusion. Use of antidepressants is allowed if at stable doses for 8 weeks prior to Screening Visit 1 and during the Screening Period up to Study Day 1.

    - High-dose antipsychotics used on a regular basis. Low doses of atypical and typical antipsychotics if used on an as-needed basis or if used at a stable dose for 8 weeks prior to Screening Visit 1 and during the Screening Period up to Study Day 1. The definition of "low dose" and "high dose" should be judged by the Investigator, and the Medical Monitor can be consulted if needed.

    - Anticholinergics such as benztropine. Anticholinergics for bladder control with limited cognitive effects are permitted but should be avoided if possible.

    - Prior use of levodopa or anti-Parkinsonian medications including dopaminergic agents, amantadine, selegiline, benztropine, and monoamine oxidase (MAO) inhibitors prescribed for the treatment of Parkinsonism or Parkinson's disease.

CONFIDENTIAL
The information contained herein may not be used, disclosed, or published without the written consent of Biogen MA Inc.

- Use of prescription narcotic medications within 4 weeks prior to Screening Visit 1. After randomization, short-term use of prescription narcotics is allowed for specific situations (e.g., after surgical procedures) and if administered at least 24 hours prior to cognitive testing.

- Use of any drug of abuse, including but not limited to, amphetamine, cannabis, cocaine, opiate, propoxyphene, methadone, methaqualone, phencyclidine, or barbiturates.

When necessary, the Medical Monitor should be contacted with any questions.

31. Vaccinations within 10 days prior to randomization (Study Day 1).

32. Prior participation in any active or passive immunotherapy study targeting Aβ or tau, unless documentation of receipt of placebo is available.

33. Last administration of β-secretase inhibitors and γ-secretase inhibitors in a study within 3 months or 5 half-lives (whichever is longer) prior to Screening unless documentation of receipt of placebo is available.

34. Participation in any study involving an investigational treatment targeting tau, unless documentation of receipt of placebo is available.

35. Participation within the 12 months prior to Screening Visit 1 in a study of any other agent(s) not included in exclusion criteria 32, 33, and 34 with a purported disease-modifying effect in AD, unless documentation of receipt of placebo is available.

36. [redacted]

Study Procedures

37. Contraindications to having a brain MRI (e.g., MRI-incompatible pacemaker; MRI-incompatible aneurysm clips, artificial heart valves, or other metal foreign body; claustrophobia that cannot be medically managed). If the MRI compatibility of implanted devices is unknown, the participant must be excluded from the study.

38. [redacted]

39. [redacted]

CONFIDENTIAL
The information contained herein may not be used, disclosed, or published without the written consent of Biogen MA Inc.

40. Has had or plans to have exposure to experimental radiation within 12 months prior to Screening Visit 1 such that regional radiation dosimetry limits would be exceeded by participating in this study.

41. [redacted]

42. Lack of good venous access, such that IV drug delivery or multiple blood draws would be precluded.

Other

43. Female participants who are pregnant or currently breastfeeding or who plan to become pregnant.

44. Participant living in an organized care facility with extensive intervention and/or support of daily living activities (e.g., a nursing home).

45. Blood donation (≥1 unit) within 1 month prior to Screening Visit 1.

46. Current enrollment or plan to enroll in any other interventional clinical study in which an investigational treatment or approved therapy for investigational use is administered.

47. Inability to comply with study requirements.

48. Other unspecified reasons that, in the opinion of the Investigator or Biogen, make the participant unsuitable for the study.

8.3. Inclusion Criteria for Long-Term Extension Period

To be eligible to participate in the LTE period, participants must meet the following eligibility criteria at Week 76 or 78 and confirmed at Week 80:

1. Ability of the participant to understand the purpose and risks of the study and provide signed and dated informed consent and authorization to use confidential health information in accordance with national and local participant privacy regulations. In countries where applicable laws allow, the participant's informant/study partner and/or legally authorized representative may provide informed consent in lieu of the participant's signature (same as above in Section 8.1).

2. Participant must have completed the placebo-controlled period of the study, including the Week 78 Visit. Participant must have taken at least 14 doses of study treatment and not have missed more than 4 consecutive doses of study treatment. Participants who do not meet these criteria may enter the LTE period only with the Sponsor's approval.

3. All women of childbearing potential and all men with female partners of childbearing potential must practice highly effective contraception during the study and for 6 months (24 weeks) after their last dose of study treatment. For further details of contraceptive requirements for this study, see Section 15.5. Female participants should not become pregnant during the study and for 6 months (24 weeks) after their last dose of study treatment. It is recommended that male participants should not father children during their participation in the study and for 6 months (24 weeks) after their last dose of study treatment (same as above in Section 8.1).

4. Medically able to undergo the study procedures and to adhere to the visit schedule at the time of study entry into the LTE period, as determined by the Investigator. Apart from a clinical diagnosis of MCI due to AD or mild AD, the participant must be in good health as determined by the Investigator, based on medical history.

5. Must have the ability to comply with procedures for protocol-related tests.

6. Must have 1 informant/study partner who, in the Investigator's opinion, has frequent and sufficient contact with the participant (at least 10 hours/week) as to be able to provide accurate information about the participant's cognitive and functional abilities. The study partner must agree to accompany the participant to clinic visits and/or be available by phone at designated times to provide information to the Investigator and study staff about the participant (and to attend in-person clinic visits that require partner input for scale completion) and must agree to monitor the participant's administration of any prescribed medications. A study partner should be available for the duration of the study, and the participation of the same study partner for the duration of the study is encouraged. The study partner must be literate and give informed consent (same as above in Section 8.1).

8.4. Exclusion Criteria for Long-Term Extension Period

Participants will be excluded from entering the LTE period if any of the following exclusion criteria exist at Week 76 or 78 and confirmed at Week 80:

1. Any medical or psychiatric contraindication or clinically significant abnormality that, in the opinion of the Investigator, will substantially increase the risk associated with the participant's participation in and completion of the study.

2. Other unspecified reasons that, in the opinion of the Investigator or Biogen, make the participant unsuitable for the study (same as above in Section 8.2).

3. [redacted]

4. [redacted]

9. SCREENING AND RANDOMIZATION

9.1. Screening

Participants or their legally authorized representatives, in countries where applicable laws allow, must provide informed consent before any screening tests are performed (see Section 17.3). [redacted]

Screening assessments, detailed in Table 1 for the placebo-controlled period, will include confirmation of diagnosis, medical history, [redacted], and safety assessments (e.g., laboratory tests, ECG, vital sign measurements, physical and neurological examinations, serum pregnancy test for women of childbearing potential). [redacted] The necessary order of the screening assessments is provided in Table 1.

During the first screening visit, under a separate (optional) initial consent process, participants can complete the cognitive scales (including the CDR, MMSE, DKEFS Category and Letter Fluency tests, DSST, Trails A, and ISLT/ISLR). This initial cognitive screening is intended to reduce the burden on participants and study sites by avoiding unnecessary testing if participants do not meet key inclusion criteria. If the participant meets inclusion criteria for the CDR, MMSE, and ISLT/ISLR, then the full consent process must be completed prior to the administration of further screening assessments. Participants may also proceed directly to the full consent process, which would allow the administration of all screening assessments.

Participant eligibility for the study will be determined during up to 3 screening visits in the approximately 9-week (65-day) Screening Period preceding randomization during the placebo-controlled period.

It is recommended that all the screening procedures for the placebo-controlled period be completed within 65 days; however, the overall Screening Period may be extended up to 90 days in consultation with the Medical Monitor in advance and provided that written documentation is received from the Sponsor or delegate ([redacted]). The following screening tests should be repeated if the Screening Period is >65 days: confirmation of eligibility criteria, abbreviated medical history, and physical examination. In addition, some cognitive assessments and/or safety assessments may require repeating depending on the duration of the screening window extension, in discussion with the Medical Monitor.

Screen failures are defined as participants who provide informed consent but are not subsequently randomized. If a participant is considered a screen failure, the reasons for exclusion must be documented in the participant's source documents and on the screening log. A minimal set of screen failure information is required to ensure transparent reporting of screen failure participants to meet the Consolidated Standards of Reporting Trials publishing requirements and to respond to queries from regulatory authorities. Minimal information includes demography, screen failure details, eligibility criteria, and any SAEs.

Participants who fail screening for the placebo-controlled period may be rescreened once at the Sponsor's discretion, [redacted]

Participants or their legally authorized representatives, in countries where applicable laws allow, who chose to participate in the LTE period must provide informed consent before any screening tests are performed (see Section 17.3). [redacted]

Participants who chose to participate in the LTE period can be screened at Week 76 or Week 78 per Table 1, and confirmation of eligibility will be checked at Week 80 per Table 2.

9.2. Randomization

Participants will be randomized on Study Day 1 after all screening and baseline assessments have been completed and after the Investigator has verified that the participants are eligible per criteria in Sections 8.1 and 8.2. Participants will be assigned a unique identification number that will be used on study-related documents pertaining to the participant. Any participant identification numbers that are assigned will not be reused even if the participant does not receive treatment. Rescreened participants will be assigned a new number.

Randomization will be stratified by [redacted], region, disease stage (MCI or mild AD), and baseline AD medication use. [redacted]

[redacted]

[redacted]

[redacted]

[redacted]

[redacted]

Participants who are participating in the LTE period and who were randomized to receive placebo during the placebo-controlled period will receive BIIB092 at the high dose (2000 mg) once every 4 weeks during the LTE period. Participants who were randomized to receive BIIB092 during the placebo-controlled period will continue to receive BIIB092 at the dose they were randomly assigned.

See the Study Reference Guide for details on randomization.

9.3. Blinding Procedures

This study consists of a randomized, double-blind, placebo-controlled period, followed by a dose-blinded LTE period with all participants receiving BIIB092.

During the double-blind, placebo-controlled period, all study staff who conduct participant assessments will be blinded to the participant treatment assignments. The individuals conducting the rating scale assessments should remain blinded to treatment assignment as well as to participant care management and only have access to the information necessary to carry out their responsibilities. As a placebo match is not provided by the Sponsor for the study (see Section 12.2), unblinded pharmacy staff are required to manage all aspects of study treatment receipt, dispensing, and preparation. To maintain the study blind, it is imperative that participant treatment assignments are not shared with the participants, their families, or any member of the blinded study team, either at the study site or at Biogen or its representatives, except the unblinded pharmacist (or designee), the unblinded pharmacy monitor, and the unblinded CRO or Biogen safety staff. Members of the DMC will also be unblinded.

[redacted]

All participants will receive infusions of study treatment at 4-week intervals during the double-blind placebo-controlled period. Those participants who are randomized to receive BIIB092 once every 12 weeks versus once every 4 weeks will receive placebo at the other 4-week dosing intervals to maintain the treatment blind. See Section 11.1 for details of the treatment regimens.

For the LTE period, the dose information must remain restricted. The study staff, the individuals conducting the rating scale assessments, and the Investigator should remain blinded to dose assignment and only have access to the information necessary to carry out their responsibilities. To maintain the study blind, it is imperative that dose information is not shared with the participants, their families, or any member of the blinded study team, either at the study site or at Biogen or its representatives, except the unblinded pharmacist (or designee), the unblinded pharmacy monitor, and the unblinded CRO or Biogen safety staff.

Once the clinical study report has been finalized, if unblinding will not jeopardize the results of ongoing related studies, Biogen will provide the randomization codes to Investigators, who then can inform their participants about the treatment received.

10. DISCONTINUATION OF STUDY TREATMENT AND WITHDRAWAL OF PARTICIPANTS FROM THE STUDY

10.1. Discontinuation of Study Treatment

A participant must permanently discontinue study treatment for any of the following reasons:

- The participant becomes pregnant. Study treatment must be discontinued immediately, and the pregnancy must be reported according to the instructions in Section 15.4.1.
- The participant withdraws consent to continue study treatment.
- The participant experiences a medical emergency that necessitates permanent discontinuation of study treatment or unblinding of the participant's treatment assignment.
- The participant experiences an AE or SAE that does not resolve or requires continued treatment that meets exclusionary criteria.
- The participant experiences a severe infusion reaction
- At the discretion of the Investigator for medical reasons.
- At the discretion of the Investigator or the Sponsor for noncompliance with the terms of the protocol.

The primary reason for discontinuation of study treatment must be recorded in the participant's case report form (CRF).

All participants who discontinue study treatment prematurely will be asked to remain in the study and continue all protocol-specified visits and procedures for the period in which they discontinued treatment (i.e., placebo-controlled or LTE). Participants who discontinue treatment during the placebo-controlled period should be encouraged to attend at least the Week 24, Week 52, and Week 78/EOS Visits and who discontinue treatment during the LTE should be encouraged to attend at least the Week 80, 104, 116, 128, 152, 156, 176, 196, 200, 224, and 226/EOS Visits, depending on when treatment was discontinued, and undergo all the scheduled procedures. At a minimum, these visits should include assessment of AEs/SAEs and concomitant medications and key clinical assessments, including at least the CDR, MMSE, ISLT, ADAS-Cog 13, ADCS-ADL, and FAQ. Participants who discontinue study treatment prematurely, regardless of the treatment period, will be asked to return to the study site for a Follow-up Safety Visit 14 weeks after receiving the last dose of study treatment. See Table 1, Table 2, Table 3, and Table 4 for the Schedule of Activities.

CONFIDENTIAL
The information contained herein may not be used, disclosed, or published without the written consent of Biogen MA Inc.

10.2. Lost to Follow-Up

Participants will be considered lost to follow-up if they repeatedly fail to return for scheduled visits and cannot be contacted by the study site.

The following actions must be taken if a participant fails to return to the study site for a required study visit:

- The study site must attempt to contact the participant and reschedule the missed visit as soon as possible and counsel the participant on the importance of maintaining the assigned visit schedule and ascertain whether the participant wishes to and/or should continue in the study.
- In cases in which the participant is deemed lost to follow-up, the Investigator or designee must make every effort to regain contact with the participant. These contact attempts should be documented in the participant's medical record.
- Should the participant continue to be unreachable, that participant will be considered to have withdrawn from the study with a primary reason of lost to follow-up.

10.3. Withdrawal of Participants From Study

Participants must be withdrawn from the study for any one of the following reasons:

- The participant withdraws consent.
- The participant enrolls into another interventional clinical study in which an investigational treatment or approved therapy for investigational use is administered.
- The participant is unwilling or unable to comply with the protocol.

The primary reason for the participant's withdrawal from the study must be recorded in the participant's CRF.

Participants who withdraw from the study prematurely are to return to the study site for an Early Termination (ET) Visit and assessments as indicated in Table 1, after the reason for withdrawal is identified. For such participants, clinical efficacy assessments specified at the ET Visit are not required if the participant discontinues treatment within 3 months of the previous primary efficacy (CDR) assessment and no significant changes in cognitive status are suspected by the Investigator; the study site should notify the Sponsor in such cases. An MRI scan should be performed if withdrawal occurs ≥6 months after the previous MRI. Blood for biomarker analysis should be collected if the participant withdraws ≥4 weeks after the previous sample was collected. [redacted]

Participants who are withdrawn from the study are also to return to the study site for a Follow-up Safety Visit 14 weeks after receiving the last dose of study treatment.

[redacted]

[redacted]

11. STUDY TREATMENT USE

11.1. Regimen

Follow the Directions for Handling and Administration (DHA).

11.1.1. Placebo-Controlled Period

Randomized, blinded study treatment will be administered by IV infusion at the study site once every 4 weeks, beginning on Study Day 1 and continuing through Week 76, for a total of 20 infusions (see Table 1).

Participants will receive 1 of the following treatment regimens:

- low-dose BIIB092 - 125 mg once every 4 weeks or 375 mg once every 12 weeks and placebo at the other 4-week dosing visits to maintain the treatment blind
- medium-dose BIIB092 - 600 mg once every 4 weeks
- high-dose BIIB092 - 2000 mg once every 4 weeks
- placebo

11.1.2. Long-Term Extension Period

In the LTE period, participants will receive BIIB092 beginning at Week 80 and continuing through Week 224. Participants who were randomized to receive BIIB092 during the placebo-controlled period will continue to receive BIIB092 at the dose they were randomly assigned. Participants randomized to receive placebo during the placebo-controlled period will receive BIIB092 at the high dose (2000 mg) during the LTE period.

11.2. Modification of Dose and/or Treatment Schedule

See Section 11.2.1 (Dose Suspension) and Section 11.2.2 (Infusion Interruption).

Dosing visits are not to be skipped, but may be delayed within the time window specified in the Schedule of Activities (Table 1). Doses should be administered at least 21 days apart, per the regimen of dosing once every 4 weeks. If the dosing interval cannot be met, the dose administration schedule should be assessed by the study Medical Monitor.

Participants should be carefully monitored for infusion reactions during dose administration. If an acute infusion reaction is observed, the participant should be managed per Section 11.2.2 (Infusion Interruption).

CONFIDENTIAL
The information contained herein may not be used, disclosed, or published without the written consent of Biogen MA Inc.

11.2.1. Dose Suspension

The independent DMC will review safety data on an ongoing basis to ensure safe and proper treatment of participants. The DMC, based on the nature, frequency, and/or severity of an AE(s), may recommend dose suspension or dose termination. See Section 19.2 for additional information about the independent DMC.

11.2.2. Infusion Interruption

The IV administration infusion time for all treatment groups is 1 to 2 hours at approximately 100 mL/hour. No premedications should be used prior to the start of study treatment infusion unless discussed with the Medical Monitor in advance and written documentation is received from Biogen authorizing the use of the premedication.

- If any mild or moderate infusion-related reaction occurs during an infusion, the infusion may be slowed or interrupted and appropriate treatment per local standards of care may be given, at the discretion of the Investigator (or designee). Based on the clinical response, the Investigator or designee will determine if the infusion may be resumed/continued, in consultation with the Medical Monitor as needed. If the infusion is resumed/continued, the infusion rate should not exceed the original infusion rate (see the DHA for infusion rate information).

- If a severe infusion-related reaction occurs during an infusion, the participant should be permanently discontinued from treatment and appropriate supportive care must be initiated in accordance with local practice.

Criteria for determining the severity of events are described in Section 15.2.3.

Discussion with the Medical Monitor can occur as needed, and it should not delay management of the medical emergency.

See Section 15.3 for reporting of AEs and Section 10 for discontinuation of study treatment.

11.3. Compliance

Compliance with treatment dosing is to be monitored and recorded by study site staff.

11.4. Concomitant Therapy and Procedures

11.4.1. Concomitant Therapy

A concomitant therapy is any drug or substance administered between Screening Visit 1 and the Follow-up Safety Visit.

The use of concomitant therapies or procedures as defined below must be recorded on the participant's CRF, according to instructions for CRF completion. AEs related to administration of these therapies or procedures must be documented on the appropriate CRF.

The Medical Monitor should be contacted with any questions about allowed or disallowed concomitant therapies.

11.4.1.1. Allowed Concomitant Therapy

- Medications for chronic conditions are allowed during the study as long as the participant has been on a stable dose of the medication(s) for 4 weeks prior to Screening Visit 1 and during the Screening Period up to Study Day 1.
- AD medications (including, but not limited to, donepezil, rivastigmine, galantamine, tacrine, and memantine) are allowed provided that participants are receiving a stable dose for at least 8 weeks prior to Screening Visit 1 and remains stable during the Screening Period up to Study Day 1 and during the study.
- Vaccination with live or attenuated vaccine is allowed during the study. Administration of any vaccine or booster should not occur <10 days prior to any dosing visit and for 10 days after a dosing visit.

11.4.1.2. Disallowed Concomitant Therapy

Any medications that, in the opinion of the Investigator, may contribute to cognitive impairment, put the participant at higher risk for AEs, or impair the participant's ability to perform cognitive testing or complete study procedures are disallowed.

Use of the following medications is disallowed:

- Long-acting benzodiazepines (e.g., valium), except for sedation prior to MRI scans for those participants requiring sedation and should not be administered within 24 hours prior to cognitive testing.
- Short/medium-acting benzodiazepines (e.g., alprazolam, lorazepam, oxazepam, temazepam) except if used chronically for sleep and on a stable dose for 8 weeks prior to Screening Visit 1 and during Screening or used short term on an as-needed basis. May not be taken within 12 hours prior to cognitive testing.
- Sedating antihistamines if taken within 12 hours prior to cognitive testing. Nonsedating antihistamines (e.g., fexofenadine, cetirizine) are allowed.
- Anticonvulsants that have significant effects on cognition per the Investigator's opinion and/or anticonvulsants used for treatment of seizures. Anticonvulsants with limited cognitive effects, such as lamotrigine, pregabalin, levetiracetam for treatment of pain, and other non-epilepsy indications are allowed if, in the opinion of the Investigator, they are not producing sedation or contributing to cognitive impairment. The participant must have been on a stable dose for 8 weeks prior to Screening Visit 1 and during the Screening Period up to Study Day 1.

CONFIDENTIAL
The information contained herein may not be used, disclosed, or published without the written consent of Biogen MA Inc.

- Antidepressants that may, in the Investigator's opinion, affect the participant's cognition (e.g., tricyclic antidepressants). Mild depression or depressive mood arising in the context of AD are not criteria for exclusion. Use of antidepressants is allowed if at stable doses for 8 weeks prior to Screening Visit 1 and during the Screening Period up to Study Day 1.

- High-dose antipsychotics used on a regular basis. Low doses of atypical and typical antipsychotics if used on an as-needed basis or if used at a stable dose for 8 weeks prior to Screening Visit 1 and during the Screening Period up to Study Day 1. The definition of "low dose" and "high dose" should be judged by the Investigator, and the Medical Monitor can be consulted if needed.

- Anticholinergics such as benztropine. Anticholinergics for bladder control with limited cognitive effects are permitted but should be avoided if possible.

- Prior use of levodopa or anti-Parkinsonian medications include dopaminergic agents, amantadine, selegiline, benztropine, and MAO inhibitors prescribed for the treatment of Parkinsonism or Parkinson's disease.

- Use of prescription narcotic medications within 4 weeks prior to Screening Visit 1. After randomization, short-term use of prescription narcotics if not for specific situations (e.g., after surgical procedures) and if administered within 24 hours prior to cognitive testing.

- Use of any drug of abuse, including but not limited to, amphetamine, cannabis, cocaine, opiate, propoxyphene, methadone, methaqualone, phencyclidine, or barbiturates.

- Immunosuppressive drugs (including systemic corticosteroids). Local corticosteroids may be permitted at Sponsor discretion.

- Parenteral Ig, blood products, plasma derivatives, plasma exchange, and plasmapheresis.

- Any investigational drug other than BIIB092.

[redacted]

- [redacted]

- [redacted]

Participants should be instructed to continue the usual medications they were on at enrollment (see allowed concomitant therapy above) and to avoid starting any new medications or herbal preparations during the study period, as these may confound the results of the study. However, medically indicated medication or treatment should not be withheld. Participants should inform the Investigator of any change in medication. The change should be reviewed by the Investigator and, if needed, the Medical Monitor to determine whether the participant's study treatment should be suspended. Medications used to treat AEs would not result in automatic withdrawal. Biogen may be consulted if required.

Participants should have an unscheduled visit (UV) prior to the initiation of, change in dose, or discontinuation of AD medication. That visit should include assessment of AEs/SAEs and concomitant medications and key clinical assessments, including, at a minimum, the CDR, MMSE, ISLT, ADAS-Cog 13, ADCS-ADL, and FAQ. During the UV, these clinical assessments need not be repeated if performed within the previous 30 days. If the visit for a change in AD medication occurs at the same time as another planned visit, all originally scheduled assessments must also take place as required and without repetition (see Table 1, Table 2, Table 3, and Table 4).

11.4.2. Concomitant Procedures

Concomitant procedures are allowed only when deemed necessary by the participant's healthcare provider. A concomitant procedure is any therapeutic intervention (e.g., surgery/biopsy, physical therapy) or diagnostic assessment (e.g., blood gas measurement, bacterial cultures) performed between the time the participant is enrolled in the study and until the participant's final clinic visit (including the Follow-up Safety Visit), unless the participant is being followed for study-related toxicity.

11.4.3. Tobacco Use

Participants' tobacco use status will be monitored continuously throughout the study and should be assessed while collecting information on concomitant therapies.

11.5. Continuation of Treatment

The participant may elect to enter the LTE following the Week 78 Visit. No further provisions are made for access to the study treatment otherwise.

CONFIDENTIAL
The information contained herein may not be used, disclosed, or published without the written consent of Biogen MA Inc.

12. STUDY TREATMENT MANAGEMENT

Study treatment will be manufactured, handled, and stored in accordance with applicable Good Manufacturing Practice.

Study site staff should follow the DHA for specific instructions on the handling, preparation, administration, and disposal of the study treatment. The DHA will also describe the masking of the IV bags of study treatment (both BIIB092 and placebo) to maintain the treatment blind. The DHA supersedes all other references (e.g., protocol or Investigator's Brochure).

Study treatment must be dispensed only by a pharmacist or appropriately qualified staff. Study treatment is to be dispensed only to participants enrolled in this study. Once study treatment is prepared for a participant, it can be administered only to that participant. Study treatment vials are for one-time use only; do not use any study treatment remaining in the vial for another participant.

12.1. BIIB092

BIIB092 is a humanized, hinge-stabilized IgG4 monoclonal antibody derived from a mouse IgG1 monoclonal antibody (IPN002). BIIB092 is produced from cell culture using a Chinese hamster ovary cell line.

BIIB092 is supplied as a liquid drug product in glass vials containing an extractable dose as per the DHA.

The contents of the BIIB092 label will be in accordance with all applicable regulatory requirements. BIIB092 should not be used after the expiration date.

12.1.1. BIIB092 Preparation

The individual preparing BIIB092 should carefully review the instructions provided in the DHA.

BIIB092 is to be administered by IV infusion following dilution with 0.9% sodium chloride solution.

If the packaging is damaged, or if there is anything unusual about the appearance or attributes of the vials or study treatment, do not use the study treatment. The vial in question should be saved at the study site and the problem immediately reported to Biogen.

12.1.2. BIIB092 Storage

Study treatment must be stored in a secure location.

Vials of BIIB092 are to be stored at 2°C to 8°C (36°F to 46°F), in a locked storage container with limited access. BIIB092 should be protected from light and freezing.

CONFIDENTIAL
The information contained herein may not be used, disclosed, or published without the written consent of Biogen MA Inc.

The administration of BIIB092 by infusion must be completed within 24 hours of preparation. If not used immediately, the infusion solution may be stored under refrigeration at 2°C to 8°C (36°F to 46°F) for up to 24 hours, and a maximum of 4 hours of the total 24-hour period can be at room temperature with exposure to room light. The maximum 4-hour period under room temperature and room light conditions includes the product administration time.

For the most up-to-date storage requirements, follow the instructions provided in the DHA.

12.1.3. BIIB092 Handling and Disposal

The Investigator must return all used and unused vials of BIIB092 as instructed by Biogen unless approved for onsite destruction.

If any BIIB092 supplies are to be destroyed at the study site, the institution or appropriate study site staff must obtain prior approval from Biogen, by providing, in writing, the destruction policy or details of the method of destruction. After such destruction, Biogen must be notified, in writing, of the details of the study treatment destroyed (e.g., lot or kit numbers, quantities), the date of destruction, and proof of destruction.

12.1.4. BIIB092 Accountability

Accountability for study treatment is the responsibility of the Investigator. The study site must maintain accurate records demonstrating dates and amount of study treatment received, to whom dispensed (participant-by-participant accounting), and accounts of any study treatment accidentally or deliberately destroyed or lost.

Unless otherwise notified, all vials, both used and unused, must be saved for study treatment accountability. By the end of the study, reconciliation must be made between the amount of BIIB092 supplied, dispensed, and subsequently destroyed, lost, or returned to Biogen. A written explanation must be provided for any discrepancies.

12.2. Placebo

The placebo (0.9% sodium chloride solution) will be provided by the study site.

12.3. Additional Protocol-Designated Products – [redacted]

[redacted]

[redacted]

[redacted]

[redacted]

13. EFFICACY, PHARMACOKINETIC, PHARMACODYNAMIC, IMMUNOGENICITY, AND HEALTH OUTCOMES ASSESSMENTS

See Section 5 for the timing of all assessments.

13.1. Clinical Efficacy Assessments

The following clinical assessments will be performed to evaluate the efficacy of BIIB092:

- CDR (to derive the primary efficacy endpoint)
- MMSE, ISLT, DKEFS Category Fluency and Letter Fluency tests, DSST, Trails A, eCog, ADCS-ADL, FAQ, ADAS-Cog 13, and NPI-10 (to derive exploratory efficacy endpoints)

Assessments administered to the participant include the CDR, MMSE, ISLT, ISLR (Screening Visit 1 only), DKEFS Category Fluency and Letter Fluency tests, DSST, Trails A, and ADAS-Cog 13. Assessments that require caregiver/informant input include the CDR, eCog, ADCS-ADL, FAQ, and NPI-10.

The clinical assessments must be administered by a trained clinician or rater, preferably by a neuropsychologist, a psychometrician or another qualified person who is experienced in the assessment of participants with cognitive deficits.

It is recommended that postbaseline CDR assessments be conducted at the same time of day at which the baseline assessment was performed to avoid diurnal variation. The rater who conducts the CDR for a participant/informant cannot complete any other rating scales for that same participant/informant or be the study site coordinator.

The rater must be certified before any assessments may be performed. When possible, the same rater should administer a given test across all visits for a given participant. See the Study Reference Guide for specific guidelines required for administration of each test and the order in which the tests should be performed.

13.2. Pharmacokinetic Assessments

BIIB092 concentrations in serum [redacted] will be determined using validated assays.

Serum PK parameters of BIIB092 to be assessed may include, but will not be limited to, the following:

- Trough concentration

- End-of-infusion concentration

[redacted]

See Section 5 for the timing of assessments. The timing for collection of postdose serum samples will be based on the completion of the BIIB092 infusion.

13.3. Pharmacodynamic Assessments

The PD properties of BIIB092 will be assessed as described below.

- Morphometric measures of certain brain areas, including volume and cortical thickness, will be assessed by MRI in all participants. Details of the MRI scanning protocol will be described in the procedural manual for MRI.
- Concentrations of disease-related biomarkers in blood, including but not limited to, tau and other markers of neurodegenerative disease will be assessed in all participants.
- [redacted]
- [redacted]

See Section 5 for the timing of assessments.

[redacted]

[redacted]

[redacted]

[redacted]

[redacted]

[redacted]

[redacted]

[redacted]

[redacted] [redacted]

[redacted]

[redacted]

13.6. Immunogenicity

A validated immunoassay will be used to assay samples for the presence of, and measure titers of, anti-BIIB092 antibodies in serum. Samples will be collected on Study Day 1 and at Weeks 4,

24, 48, 76, 80, 104, 116, 156, 176, 196, 224, and 238/FUV (see Section 5). All samples will be collected predose.

13.7. Health Outcomes Assessments

The following tests will be performed to assess the effects of BIIB092 on caregiver burden, participant quality of life, and resource utilization:

- ZBI
- QoL-AD
- RUD-Lite

The ZBI, QoL-AD, and RUD-Lite are administered to the caregiver; the QoL-AD is also administered to the participant. The recommended order of administration of these assessments is specified in the Study Reference Guide.

See Section 5 for the timing of assessments.

14. SAFETY ASSESSMENTS

See Section 5 for the timing of all safety assessments.

14.1. Clinical Safety Assessments

The following clinical assessments will be performed to evaluate the safety profile of BIIB092:

- Physical examinations, including height and weight measurements.
- Neurological examinations.
- Vital sign measurements: temperature, pulse rate, SBP, DBP, and respiratory rate. Measurements will be recorded after the participant has been resting in a supine position for 10 minutes. At selected timepoints (see Table 1), blood pressure and pulse rate will also be recorded after the participant has been standing for ≥ 2 minutes.
- 12-lead (paper) ECGs, to be performed in triplicate at each timepoint with approximately 1 minute between replicates. Each ECG must be performed after the participant has been resting in a supine position for 10 minutes. The ECGs will be read by a central reader; copies of all raw ECG data must be made available to Biogen.
- Laboratory safety assessments (see Section 14.2).
- Columbia - Suicide Severity Rating Scale (C-SSRS).
- Concomitant therapy and procedure recording.
- AE and SAE monitoring.

14.2. Laboratory Safety Assessments

Samples will be analyzed using Good Laboratory Practice-validated assays.

The following laboratory assessments will be performed to evaluate the safety profile of BIIB092:

- Hematology: red blood cell count, platelet count, hemoglobin level, hematocrit, mean corpuscular volume, mean corpuscular hemoglobin, mean corpuscular hemoglobin concentration, and total white blood cell count with absolute counts and percentages of differential cells (neutrophils, monocytes, lymphocytes, eosinophils, and basophils).

CONFIDENTIAL
The information contained herein may not be used, disclosed, or published without the written consent of Biogen MA Inc.

- Clinical chemistry: total protein, albumin, creatinine, BUN, uric acid, bilirubin (total and direct), alkaline phosphatase, ALT, AST, lactate dehydrogenase, gamma-glutamyl transferase, glucose, calcium, phosphorus, bicarbonate, chloride, sodium, and potassium.

- Urinalysis: color, specific gravity, pH, protein, glucose, leukocyte esterase, blood ketones (and microscopic examination, if abnormal).

- Serum and urine pregnancy tests for women of childbearing potential only.

- At the first screening visit only: testing for HIV (to be performed based upon Investigator assessment of HIV risk factors; the requirement for testing during Screening may be omitted if it is not permitted by local regulations), glycosylated hemoglobin, HBsAg, anti-HBc, and hepatitis C antibody; alcohol/drug screen; and follicle-stimulating hormone (FSH; postmenopausal women only).

- [redacted]

- [redacted]

15. SAFETY DEFINITIONS, RECORDING, REPORTING, AND RESPONSIBILITIES

Throughout the course of the study, every effort must be made to remain alert to possible AEs. If an AE occurs, the first concern should be for the safety of the participant. If necessary, appropriate medical intervention should be provided.

At the signing of the ICF, each participant or his/her legally authorized representative, in countries where applicable laws allow, and/or main caregiver must be given the names and telephone numbers of study site staff for reporting AEs and medical emergencies.

15.1. Definitions

15.1.1. Adverse Event

An AE is any untoward medical occurrence in a patient or clinical investigation participant administered a pharmaceutical product and that does not necessarily have a causal relationship with this treatment. An AE can therefore be any unfavorable and unintended sign (including an abnormal laboratory finding), symptom, or disease temporally associated with the use of a medicinal (investigational) product, whether or not related to the medicinal (investigational) product.

Determination of whether an abnormal laboratory value, vital sign result, and/or ECG result meets the definition of an AE will be made by the Investigator. Abnormal results are not considered AEs unless one or more of the following criteria are met:

- The result meets the criteria for an SAE
- The result requires the participant to receive specific corrective therapy
- The result is considered by the Investigator to be clinically significant

15.1.2. Serious Adverse Event

An SAE is any untoward medical occurrence that at any dose:

- Results in death
- In the view of the Investigator, places the participant at immediate risk of death (a life-threatening event); however, this does not include an event that, had it occurred in a more severe form, might have caused death
- Requires inpatient hospitalization or prolongation of existing hospitalization
- Results in persistent or significant disability/incapacity

- Results in a congenital anomaly/birth defect
- Is a medically important event

A medically important event is an AE that, in the opinion of the Investigator, may jeopardize the participant or may require intervention to prevent one of the other outcomes listed in the definition above. (Examples of such medical events include allergic bronchospasm requiring intensive treatment in an emergency room or convulsions occurring at home that do not require an inpatient hospitalization.)

15.1.3. Prescheduled or Elective Procedures or Routinely Scheduled Treatments

A prescheduled or elective procedure or a routinely scheduled treatment will not be considered an SAE, even if the participant is hospitalized. The study site must document all of the following:

- The prescheduled or elective procedure or routinely scheduled treatment was scheduled (or was on a waiting list to be scheduled) prior to obtaining the participant's consent to participate in the study.
- The condition requiring the prescheduled or elective procedure or routinely scheduled treatment was present before and did not worsen or progress in the opinion of the Investigator between the participant's consent to participate in the study and the time of the procedure or treatment.
- The prescheduled or elective procedure or routinely scheduled treatment is the sole reason for the intervention or hospital admission.
    - If a participant is hospitalized due to local requirements for administration of the study treatment, the hospitalization should not be considered an SAE unless one of the requirements in Section 15.1.2 is met.

15.2. Safety Classifications

15.2.1. Investigator Assessment of Events

All events must be assessed to determine the following:

- If the event meets the criteria for an SAE as defined in Section 15.1.2.
- The relationship of the event to study treatment, [redacted] as defined in Section 15.2.2.
- The severity of the event as defined in Section 15.2.3.

15.2.2. Relationship of Events to Study Treatment, [redacted]

The following definitions should be considered when evaluating the relationship of AEs and SAEs to the study treatment.

Relationship of Event to Study Treatment	
Not related	An AE will be considered "not related" to the use of the investigational product if there is not a reasonable possibility that the event has been caused by the product under investigation. Factors pointing toward this assessment include but are not limited to the lack of reasonable temporal relationship between administration of the investigational product and the AE, the presence of a biologically implausible relationship between the product and the AE, or the presence of a more likely alternative explanation for the AE.
Related	An AE will be considered "related" to the use of the investigational product if there is a reasonable possibility that the event may have been caused by the product under investigation. Factors that point toward this assessment include but are not limited to a positive rechallenge, a reasonable temporal sequence between administration of the investigational product and the AE, a known response pattern of the suspected product, improvement following discontinuation or dose reduction, a biologically plausible relationship between the product and the AE, or a lack of an alternative explanation for the AE.
[redacted]	
[redacted]	[redacted]
[redacted]	[redacted]
[redacted]	
[redacted]	[redacted]

[redacted]	[redacted]

15.2.3. Severity of Events

The following definitions should be considered when evaluating the severity of AEs and SAEs:

Severity of Event	
Mild	Symptoms barely noticeable to participant or does not make participant uncomfortable; does not influence performance or functioning; prescription drug not ordinarily needed for relief of symptoms but may be given because of personality of participant.
Moderate	Symptoms of a sufficient severity to make participant uncomfortable; performance of daily activity is influenced; participant is able to continue in study; treatment for symptoms may be needed.
Severe	Symptoms cause severe discomfort; symptoms cause incapacitation or significant impact on participant's daily life; severity may cause cessation of treatment with study treatment; treatment for symptoms may be given and/or participant hospitalized.

15.2.4. Expectedness of Events

Expectedness of all AEs will be determined by Biogen according to the Investigator's Brochure.

15.3. Monitoring and Recording Events

15.3.1. Adverse Events

Any AE experienced by the participant between the time of first dose of study treatment and the participant's final clinic visit (including the Follow-up Safety Visit) is to be recorded on the CRF, regardless of the severity of the event or its relationship to study treatment, [redacted] At each study visit, the Investigator will assess the participant for AEs and will record any new AEs or updates to previously reported AEs on the CRF.

[redacted]

AEs that are ongoing when the participant completes or discontinues the study will be followed by the Investigator until the event has resolved, stabilized, or returned to baseline status. AE outcome will be recorded on the CRF, as applicable.

15.3.2. Adverse Events of Special Interest

Immunogenicity is an AE of special interest that is under monitoring in all ongoing studies. Participants will also be monitored for possible infusion-associated AEs and/or hypersensitivity reactions during and after completion of the investigational medicinal product infusion.

15.3.3. Serious Adverse Events

Any SAE experienced by the participant between the time of the signing of the ICF and the participant's final clinic visit (including the Follow-up Safety Visit) is to be recorded on an SAE form, regardless of the severity of the event or its relationship to study treatment. SAEs must be reported to Biogen within 24 hours as described in Section 15.3.4. Follow-up information regarding an SAE also must be reported within 24 hours.

Participants will be followed for all SAEs until the final clinic visit (including the Follow-up Safety Visit). Thereafter, the event should be reported to Biogen only if the Investigator considers the SAE to be related to study treatment.

Any SAEs that is ongoing when the participant completes or discontinues the study will be followed by the Investigator until the event has resolved, stabilized, or returned to baseline status.

15.3.4. Immediate Reporting of Serious Adverse Events

In order to adhere to all applicable laws and regulations for reporting an SAE, the study site must formally notify Biogen within 24 hours of the study site staff becoming aware of the SAE. It is the Investigator's responsibility to ensure that the SAE reporting information and procedures are used and followed appropriately.

Reporting Information for SAEs
A report ***must be submitted*** to Biogen regardless of the following:<br>• Whether or not the participant has undergone study-related procedures<br>• Whether or not the participant has received study treatment<br>• The severity of the event<br>• The relationship of the event to study treatment [redacted]<br>To report initial or follow-up information on an SAE, fax a completed SAE form; see the Study Reference Guide's Official Study Contact List for complete contact information.

15.3.4.1. Deaths

Death is an outcome of an event. The event that resulted in death should be recorded on the appropriate CRF. All causes of death must be reported as SAEs within 24 hours of the study site becoming aware of the event. The Investigator should make every effort to obtain and send

CONFIDENTIAL
The information contained herein may not be used, disclosed, or published without the written consent of Biogen MA Inc.

death certificates and autopsy reports to Biogen. The term death should be reported as an SAE only if the cause of death is not known and cannot be determined.

15.3.5. Suspected Unexpected Serious Adverse Reactions

Suspected unexpected serious adverse reactions (SUSARs) are SAEs that are unexpected and judged by the Investigator or Biogen to be related to the study treatment administered.

Appropriate personnel at Biogen will unblind SUSARs for the purpose of regulatory reporting. Biogen will submit SUSARs (in blinded or unblinded fashion) to regulatory agencies according to local law. Biogen will submit SUSARs to Investigators in a blinded fashion.

15.4. Procedures for Handling Special Situations

15.4.1. Pregnancy

Participants should not become pregnant during the study and for 6 months (24 weeks) after their last dose of study treatment. If a female participant becomes pregnant, study treatment must be discontinued *immediately*. It is recommended that male participants should not father children during their participation in the study and for 6 months (24 weeks) after their last dose of study treatment.

The Investigator must report a pregnancy occurring in a female participant by faxing the appropriate form to Biogen within 24 hours of the study site staff becoming aware of the pregnancy. See the Study Reference Guide's Official Study Contact List for complete contact information. The Investigator or study site staff must report the outcome of the pregnancy to Biogen. A pregnancy is not considered an AE and should not be recorded on the AE CRF.

Congenital abnormalities and birth defects in the offspring of male or female participants should be reported as an SAE if conception occurred during the study Treatment Period.

15.4.2. Overdose

An overdose is any dose of study treatment administered to a participant or taken by a participant that exceeds the dose assigned to the participant according to the protocol. Overdoses are not considered AEs and should not be recorded as an AE on the CRF; however, all overdoses must be recorded on an Overdose form and faxed to Biogen within 24 hours of the study site becoming aware of the overdose. An overdose must be reported to Biogen even if the overdose does not result in an AE. If an overdose results in an AE, the AE must be recorded. If an overdose results in an SAE, both the SAE and Overdose forms must be completed and faxed to Biogen. All study treatment related dosing information must be recorded on the dosing CRF.

15.4.3. Medical Emergency

In a medical emergency requiring immediate attention, study site staff will apply appropriate medical intervention, according to current standards of care. The Investigator (or designee)

CONFIDENTIAL
The information contained herein may not be used, disclosed, or published without the written consent of Biogen MA Inc.

should contact Biogen 24-hour emergency medical support: 1-973-659-6677. See the Study Reference Guide's Official Study Contact List for complete contact information.

15.4.3.1. Unblinding for Medical Emergency

In a medical emergency when knowledge of the participant's treatment assignment may influence the participant's clinical care, the Investigator or appropriate designee may access the participant's treatment assignment in the IRT system by accessing the internet or using a phone-based interface. Further information about the IRT unblinding function or 24-hour, 7-day-a-week support contact information is available in the IRT manual for the study.

The Investigator must document the reasons for unblinding in the participant's source documents. The Investigator is strongly advised not to divulge the participant's treatment assignment to any individual not directly involved in managing the medical emergency, or to personnel involved with the analysis and conduct of the study.

The Investigator can contact Biogen or its designee to discuss such situations, but such a discussion should not delay management of the medical emergency. The Investigator should inform Biogen or its designee as soon as possible if unblinding occurs.

15.5. Contraception Requirements

All women of childbearing potential and all men must ensure that highly effective contraception is used during the study and for 6 months (24 weeks) after their last dose of study treatment. In addition, participants should not donate sperm or eggs for the duration of the study and for at least 5 times the half-life of BIIB092 or 6 months, whichever is longer, after their last dose of study treatment.

For the purposes of this study, women who do not meet one of the following criteria are considered to be physiologically capable of becoming pregnant and are, therefore, defined as women of childbearing potential:

- Postmenopausal:
  - 12 continuous months of natural (spontaneous) amenorrhea without an alternative medical cause and a serum FSH level >40 mIU/mL at Screening Visit 1.
  - 6 weeks after surgical bilateral oophorectomy with or without hysterectomy.
- Posthysterectomy.
- Female surgical sterilization (e.g., bilateral tubal ligation).

For the purposes of the study, highly effective contraception is defined as use of 2 of the following:

For female participants:

- Established use of oral, intravaginal, or transdermal combined (estrogen and progestogen containing) hormonal methods of contraception associated with the inhibition of ovulation.
- Established use of oral, injected, or implanted hormonal methods of contraception.
- Placement of an intrauterine device or intrauterine hormone-releasing system.
- Barrier methods of contraception with use of a spermicide, including condom, nonprescription sponge, or occlusive cap (diaphragm or cervical vault cap) used with spermicidal foam, gel, film, or cream suppository.
- Sex with a male who has undergone surgical sterilization (with the appropriate postvasectomy documentation of the absence of sperm in the ejaculate).

For male participants:

- Vasectomy with negative semen analysis at follow-up.
- Condoms with spermicide.
- Sex with a woman who uses the methods described for females if she is of childbearing potential.

For all participants, true abstinence, when this is consistent with the preferred and usual lifestyle of the participant, can be considered an acceptable method of contraception based on the evaluation of the Investigator who should also take into consideration the duration of the clinical study. Periodic abstinence (e.g., calendar, ovulation, symptothermal, postovulation methods) and withdrawal are not considered acceptable methods of contraception.

Pregnancy reporting is described in Section 15.4.1.

15.6. Safety Responsibilities

15.6.1. The Investigator

The Investigator's responsibilities include the following:

- Monitor and record all AEs, including SAEs, on the CRF regardless of the severity or relationship to study treatment, [redacted].
- Determine the seriousness, relationship to study treatment, [redacted], and severity of each event.

- Determine the onset and resolution dates of each event.
- Monitor and record all pregnancies in female participants and follow up on the outcome of all pregnancies.
- Complete an SAE form for each SAE and fax or email it to Biogen within 24 hours of the study site staff becoming aware of the event.
- Pursue SAE follow-up information actively and persistently. Follow-up information must be reported to Biogen within 24 hours of the study site staff becoming aware of new information.
- Ensure all AE and SAE reports are supported by documentation in the participants' medical records.
- Pursue AE follow-up information, if possible, until the event has resolved or become stable. Record AE follow-up information, including resolution, on the CRF, as applicable.
- Report SAEs to local ethics committees, as required by local law.

15.6.2. Biogen

Biogen's responsibilities include the following:

- Before a study site can enroll any participants, the Clinical Monitor is responsible for reviewing with study site staff the definitions of AE and SAE, as well as the instructions for monitoring, recording, and reporting AEs and SAEs.
- Biogen is to notify all appropriate regulatory authorities, central ethics committees, and Investigators of SAEs, as required by local law, within required time frames.

16. STATISTICAL METHODS AND DETERMINATION OF SAMPLE SIZE

The objectives of the study and the endpoints to be analyzed are listed in Section 6.

16.1. General Considerations

Data will be collected and analyzed separately for the placebo-controlled period and LTE period:

1. After the placebo-controlled period is completed, efficacy and safety analyses will be performed.
2. Analyses will be performed as needed during the LTE period. Descriptive statistics will be used to evaluate the long-term safety and efficacy data of BIIB092.

Summary statistics will be presented. For continuous endpoints, summary statistics will generally include: number of participants with data, mean, SD, median, and range. For categorical endpoints, this will generally include: number of participants randomized or dosed, number of participants with data, and the percentage of those with data in each category. Data for participants who were randomized to BIIB092 low dose (125 mg once every 4 weeks or 375 mg once every 12 weeks) will be pooled for statistical testing and modeling for efficacy, PD, and health outcome analyses, unless otherwise specified in the placebo-controlled period. All statistical tests will be 2-sided.

Similar analyses will be applied to both the placebo-controlled and LTE periods, except for efficacy analyses, which will be detailed in the statistical analysis plan (SAP).

16.2. Demography and Baseline Disease Characteristics

Demographics and baseline data will be summarized by treatment group with summary statistics or with frequency distributions.

16.3. Safety

16.3.1. Analysis Population

The population for safety analyses is defined as all participants who were randomized and who received at least 1 dose of study treatment.

16.3.2. Methods of Analysis

16.3.2.1. Adverse Events

All analyses of AEs will be based on the principle of treatment emergence. An AE is considered to be treatment emergent if it has an onset date on or after the date of first dosing, or if it was

present prior to the first dose and subsequently worsened. The incidence of all AEs will be presented by system organ class and preferred term by dose group and for the overall active treatment group. In addition, the incidence of all AEs will be presented by severity, by relationship to study treatment, [redacted].

16.3.2.2. Clinical Laboratory Results

Clinical laboratory evaluations include hematology, blood chemistry, and urinalysis evaluations. Analyses of clinically significant abnormalities, shifts from baseline to postbaseline relative to the normal range, as well as changes from baseline by visit, will be presented by dose group and for the overall active treatment group.

16.3.2.3. Vital Signs

The analysis of vital signs will focus on clinically relevant abnormalities, which will be defined in more detail in the SAP. The incidence of clinically relevant abnormalities in vital signs will be summarized by dose group and for the overall active treatment group.

16.3.2.4. Electrocardiograms

The analysis of ECG data will focus on clinically relevant abnormalities, which will be defined in more detail in the SAP. ECG changes from baseline may be summarized using descriptive statistics and presented by dose group, overall active treatment group, and visit.

16.3.2.5. Physical and Neurological Examinations

Abnormal findings during physical and neurological examinations will be recorded as AEs and will be reflected in the summary of AEs.

16.3.2.6. Columbia - Suicide Severity Rating Scale

C-SSRS data will be summarized using descriptive statistics and presented by dose group and for the overall active treatment group.

16.4. Efficacy

16.4.1. Analysis Population

Efficacy analyses will use the intent-to-treat (ITT) population, defined as all participants who were randomized and who received at least 1 dose of study treatment. For each endpoint, additional conditions may apply to the definition of the population for the analysis. Participants will be analyzed in the groups to which they were randomized. Subgroup analysis may be done for selected endpoints (e.g., CDR-SB).

16.4.2. Methods of Analysis

16.4.2.1. Analysis of Primary Efficacy Endpoint (Clinical Dementia Rating Scale – Sum of Boxes)

The CDR-SB is the primary efficacy endpoint. The population for the analysis of CDR-SB will be participants in the ITT population who have a baseline and at least 1 postbaseline CDR-SB score.

A mixed model with repeated measures (MMRM) will be used to analyze changes from Baseline in CDR-SB using fixed effects of treatment, time, treatment-by-time interaction, baseline CDR-SB, baseline CDR-SB-by-time interaction, region, disease stage (MCI versus mild AD), and baseline AD medication use. The correlation between repeated measures of the outcomes will be taken into consideration. The least-squares (LS) means, the differences in LS means between each treatment group versus placebo at Weeks 24, 52, and 78, 95% confidence intervals (CIs), and p-values will be presented. Changes from baseline in CDR subscores will be analyzed in a similar model. Additional details will be provided in the SAP.

The multiple comparison procedure modelling (MCP-MOD) method will be used to assess and model dose-response relationships (potential candidate models will include linear, linear-log, quadratic, maximum response [E_{max}], and logistic models as specified in Section 16.10) while controlling for multiplicity. The dose-response parameter of interest for MCP-MOD will be the LS means at Week 78 for each dose group from the mixed-effects model. Pairwise comparisons of each BIIB092 dose group versus placebo will also be conducted.

16.4.2.2. Analysis of Exploratory Clinical Endpoints

The clinical endpoints assessing AD progression from Baseline include the results of the MMSE, ISLT, DKEFS, DSST, Trails A, eCog, ADCS-ADL, FAQ, ADAS-Cog 13, and NPI-10.

These clinical endpoints will be analyzed using data from participants in the ITT population who have a baseline and at least 1 postbaseline score for the specific endpoint being analyzed. As these are exploratory endpoints in this study, no additional multiplicity adjustment procedures will be applied to the analyses of these endpoints.

An MMRM approach will be used to analyze changes from baseline using fixed effects of treatment, time, treatment-by-time interaction, baseline score, baseline score-by-time interaction, region, disease stage (MCI versus mild AD), and baseline AD medication use. The correlation between repeated measures of the outcomes will be taken into consideration. The LS means, the differences in LS means between each treatment group versus placebo at Weeks 24, 52, and 78, and the 95% CIs and p-values will be presented.

Exploratory analysis may be performed to develop a composite cognitive score participants with MCI due to AD and/or participants with mild AD.

16.4.3. Analysis for the Long-Term Extension Period

The analysis will include all participants who received at least 1 dose of study treatment in the LTE period.

The endpoints for the LTE period are change from baseline over the placebo-controlled and LTE periods of the study. Analyses will be presented by treatment group in the LTE period using the placebo-controlled period baseline. Details of the analyses will be prespecified in the SAP.

16.5. Pharmacokinetics

16.5.1. Analysis Population

The population for serum PK analyses is defined as all participants in the ITT population who have at least 1 measurable postbaseline BIIB092 concentration in serum.

[redacted]

Participants who receive BIIB092 125 mg once every 4 weeks will be analyzed separately from those who receive 375 mg once every 12 weeks.

16.5.2. Methods of Analysis

Samples for measuring serum [redacted] concentrations of BIIB092 will be collected as specified in Section 5. BIIB092 concentrations in serum [redacted] will be summarized using descriptive statistics for the first 20 participants in the PK/PD analysis at Week 12 and at the end of the study, by dose group.

This study will collect only sparse PK samples, thus the serum [redacted] concentration data will be summarized descriptively by visit and dose group. No noncompartmental or compartmental methods will be used to analyze the PK data for presentation in the clinical study report. Details of the PK analysis will be described in the SAP.

Mean serum [redacted] concentrations of BIIB092 will be plotted versus time by dose group on both a linear and a logarithmic scale. No dose proportionality assessments will be conducted due to the sparse PK data sampling.

Atypical drug concentrations (e.g., very low or very high) will be excluded from the analysis, if no apparent explanation exists. Concentration observations will also be removed from the data set if corresponding dosing or sampling times are missing or cannot be reconstructed. Concentration values below the limit of quantification will be appropriately handled per the SAP. All deletions of data points will be appropriately documented.

Population PK analysis may be conducted to estimate BIIB092 population PK parameters and to identify potential covariates (e.g., demographics, body weight, and anti-BIIB092 antibodies) on the variability of BIIB092 PK. Results will be presented in a separate report.

16.6. Pharmacodynamics

16.6.1. Analysis Population

The PD analysis population is defined as all participants who in the ITT population who have at least 1 post baseline assessment of the specific parameter being analyzed.

[redacted]

16.6.3. Methods of Analysis for Other Pharmacodynamic Parameters

MRI scans and blood [redacted] samples will be collected as specified in Section 5. MRI results analyzed may include, but will not be limited to, MRI morphometric measures including volume and cortical thickness of certain brain areas. [redacted]

Data for these exploratory potential biomarker candidates related to BIIB092 biological activity or disease progression will be summarized using descriptive statistics and will be presented by dose group.

16.7. Health Outcomes

16.7.1. Analysis Population

The ITT population will be used for the analysis of health outcomes data.

16.7.2. Methods of Analysis

The scores for and changes from baseline in the ZBI and QoL-AD, and cumulative resource utilization collected from the RUD-Lite assessments up to Week 72, will be summarized by treatment group.

16.8. Immunogenicity

16.8.1. Analysis Population

The population for analyses of anti-BIIB092 antibodies is defined as all participants who were randomized and received at least 1 dose of study treatment and who have at least 1 postdose serum sample evaluated for anti-BIIB092 antibodies.

16.8.2. Methods of Analysis

The incidence of anti-BIIB092 antibodies will be summarized by treatment group over time.

16.9. Interim Analyses

Interim analyses may be performed after 50% to 100% of participants have completed the Week 52 visit (or discontinued) for the purpose of future study planning and/or futility analyses. In order to maintain the treatment blind, an independent group external to Biogen that will not be involved in the conduct of the study after unblinding will perform the interim analyses. This independent group will present the unblinded interim analyses to the DMC for review. After the DMC review, a small internal independent team (separate from the study team) may review the unblinded results for the purpose of future study planning under a study integrity charter. No Type I error adjustment will be made. No changes will be made to this study based on the interim analysis results.

[redacted]

After all participants have completed the placebo-controlled period, the Sponsor may perform an unblinded analysis. The Sponsor may perform additional interim analyses thereafter.

16.10. Sample Size Considerations

There was no formal sample size calculation for the primary endpoint of safety.

The planned sample size is 528 participants, randomized in a 1:1:2:2 ratio, with 88 participants assigned to the BIIB092 low-dose group (44 assigned to 125 mg once every 4 weeks and 44 assigned to 375 mg once every 12 weeks), 88 participants assigned to the medium-dose group (600 mg once every 4 weeks), 176 participants assigned to the high-dose group (2000 mg once every 4 weeks), and 176 participants assigned to the placebo group. This sample size provides approximately 80% power to detect a dose-response relationship in the change from baseline in CDR-SB (primary efficacy endpoint) at 18 months (Week 78), assuming a mean change of 1.99 from baseline in CDR-SB at 18 months in the placebo group and a common SD of 2.38, a maximal 40% reduction for the highest BIIB092 dose group compared with the placebo group, and an estimated 20% dropout rate at 18 months (Week 78) in this study. Six different dose-response relationships will be tested at the 2-sided 5% significance level, using the MCP-MOD method to control for multiplicity [Bretz 2005]. Optimal contrasts will be constructed to detect potential dose-response trends under common dose-response curves (e.g., Emax, exponential, logistic, linear in log dose, and quadratic model) which are illustrated with the parameters shown in Figure 2.

The mean and SD of the change from baseline in CDR-SB at 18 months for the placebo group is based on available Alzheimer's Disease Neuroimaging Initiative (ADNI) data from ADNI 1, ADNI 2, and [redacted].

[redacted]

[redacted]

[redacted]

In the LTE period, the actual sample size, [redacted], is dependent on the enrollment rate, and thus, sample size calculation is not needed.

Figure 2: Dose-Response Shapes Based on Clinical Dementia Rating Scale – Sum of Boxes Mean Change from Baseline at Week 78

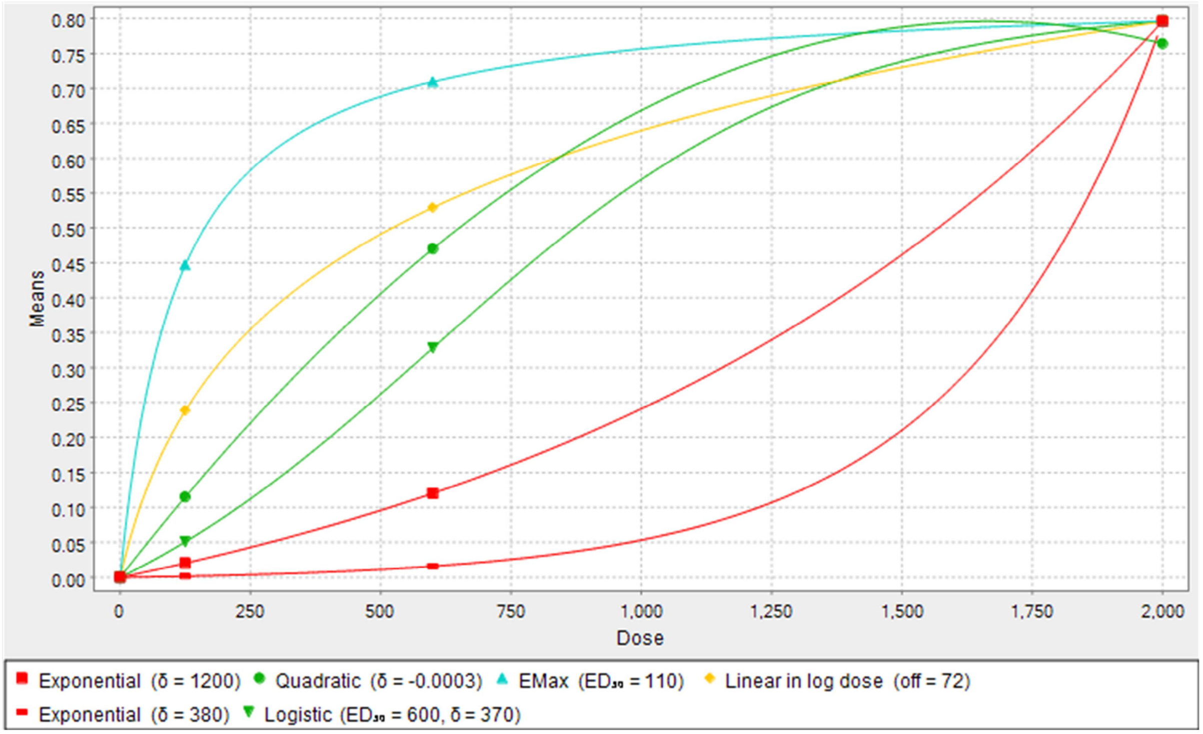

ED_{50} = median effective dose; Emax = maximum response
Note: This figure was generated using ADDPLAN® DF Version 3.1.8, by Aptiv solutions.

17. ETHICAL REQUIREMENTS

Biogen, a CRO, and the Investigator must comply with all instructions, regulations, and agreements in this protocol and applicable International Council for Harmonisation (ICH) and Good Clinical Practice (GCP) guidelines and conduct the study according to local regulations.

The Investigator is responsible for endorsing all data on completed CRFs electronically, prior to any Interim lock or Database lock.

The Investigator may delegate responsibilities for study-related tasks where appropriate to individuals sufficiently qualified by education, training, and experience, in accordance with applicable ICH and GCP guidelines. The Investigator should maintain a list of the appropriately qualified persons to whom significant study-related duties have been delegated. The Investigator is responsible for supervising those individuals and for implementing procedures to ensure the integrity of the tasks performed and any data generated.

17.1. Declaration of Helsinki

This study will be performed in alignment with the ethical principles outlined in the Declaration of Helsinki.

17.2. Ethics Committee

The Investigator must obtain ethics committee approval of the protocol, ICF, and other required study documents prior to starting the study. Biogen will submit documents on behalf of the study sites worldwide in compliance with local regulations.

If the Investigator makes any changes to the ICF, Biogen must approve the changes before the ICF is submitted to the ethics committee. A copy of the approved ICF must be provided to Biogen. After approval, the ICF must not be altered without the agreement of the relevant ethics committee and Biogen.

It is the responsibility of the Investigators to ensure that all aspects of institutional review are conducted in accordance with current applicable regulations.

Biogen must receive a letter documenting ethics committee approval, which specifically identifies the protocol, protocol number, and ICF, prior to the initiation of the study. Protocol amendments will be participant to the same requirements as the original protocol.

A progress report must be submitted to the ethics committee at required intervals and not less than annually.

At the completion or termination of the study, the study site must submit a close-out letter to the ethics committee and Biogen.

CONFIDENTIAL
The information contained herein may not be used, disclosed, or published without the written consent of Biogen MA Inc.

17.3. Participant Information and Consent

Prior to performing any study-related activities under this protocol, including screening tests and assessments, written informed consent with the approved ICF must be obtained from the participant or participant's legally authorized representative (e.g., legal guardian), as applicable, in accordance with local practice and regulations.

During the first screening visit, under a separate (optional) initial consent process, participants can complete the cognitive scales. If the participant meets inclusion criteria for the CDR, MMSE, and ISLT/ISLR, then the full consent process must be completed prior to the administration of further screening assessments. Participants may also proceed directly to the full consent process, which would allow the administration of all screening assessments. See Section 9.1 for details of the screening process.

The background of the proposed study, the procedures (including ApoE testing), the benefits and risks of the study, and that study participation is voluntary for the participant must be explained to the participant (or the participant's legally authorized representative, in countries where applicable laws allow). The participant must be given sufficient time to consider whether to participate in the study.

Participants will be informed that their race and ethnicity will be collected during the study (unless the collection is not permitted by applicable law or not approved by the governing ethics committee) and the data will be used during analysis of study results.

Participants will also be informed that audio recordings may be made of some clinical assessments in order to allow for central review for standardization of test administration, where allowable by country and/or local authorities.

[redacted]

Participants or their legally authorized representatives, in countries where applicable laws allows, who chose to participate in the LTE period must provide informed consent before any LTE period screening tests are performed. [redacted] A participant's informant/study partner must also provide consent.

[redacted]

[redacted]

Copies of the participant's signed and dated ICF(s) (optional initial consent, if applicable, and full consent) and a copy of the signed and dated optional genetic sampling consent form, if applicable, must be given to the participant or the participant's legally authorized representative, in countries where applicable laws allow. The original signed and dated ICFs will be retained with the study records. Local regulations must be complied with in respect to the final disposition of the original (wet signature) and copies of the signed and dated ICFs.

Confirmation of informed consent must also be documented in the participant's medical record.

The participant's study partner/informant must also provide written informed consent to participate in the study and be reconsented to participate in the LTE period. The original forms will be managed and archived in the same manner as the participants' ICFs, as described above.

Participants or their informant/study partner can withdraw consent to participate in the study at any time. [redacted]

17.4. Participant Data Protection

Prior to any testing under this protocol, including screening tests and assessments, candidates must also provide all authorizations required by local law (e.g., Protected Health Information authorization in North America).

During Screening, participants' race and ethnicity will be collected (unless the collection is not permitted by applicable law or not approved by the governing ethics committee). Race and ethnicity data will be used to describe the demographic profile of the study population and to evaluate the balance of demographic characteristics across the randomized treatment groups. These data may also be used in the analysis of the safety and/or PK profile of the study treatment. It is unknown whether the effects of the study treatment are influenced by race or ethnicity.

Audio recordings may be made of some clinical assessments in order to allow for central review for standardization of test administration, where allowable by country and/or local authorities.

Study reports will be used for research purposes only. The participant will not be identified by name in CRFs, study-related forms, study reports, or any related publications. Biogen, its partners and designees, ethics committees, and various government health agencies may inspect the records of this study. Every effort will be made to keep the participant's personal medical data confidential.

17.5. Compensation for Injury

Biogen maintains appropriate insurance coverage for clinical studies and will follow applicable local compensation laws.

17.6. Conflict of Interest

The Investigators should address any potential conflicts of interest (e.g., financial interest in Biogen) with the participant before the participant makes a decision to participate in the study.

17.7. Registration of Study and Disclosure of Study Results

Biogen will register the study and post study results regardless of outcome on a publicly accessible website in accordance with the applicable laws and regulations.

CONFIDENTIAL
The information contained herein may not be used, disclosed, or published without the written consent of Biogen MA Inc.

18. ADMINISTRATIVE PROCEDURES

18.1. Study Site Initiation

The Investigator must not screen any participants prior to completion of a study initiation visit, conducted by Biogen. This initiation visit will include a detailed review of the protocol and study procedures.

18.2. Quality Control and Quality Assurance

Quality control procedures will be implemented at each stage of data handling to ensure that all data are reliable and have been processed correctly. Data anomalies will be communicated to the study sites for clarification and resolution, as appropriate. The Investigator is responsible for endorsing all CRF data prior to any interim or final database lock.

During and/or after completion of the study, quality assurance officers named by Biogen or the regulatory authorities may wish to perform onsite audits or inspections. The Investigator will be expected to cooperate with any audit or inspection and to provide assistance and documentation (including source data) as requested.

18.3. Monitoring of the Study

The Investigator must permit study-related monitoring by providing direct access to source data and to the participants' medical histories. Source data must be attributable, legible, contemporaneous, original, accurate, and complete. Changes to source data must be traceable, not obscure the original entry, and be explained if necessary (e.g., with an audit trail). The Investigator should maintain a record of the location(s) of essential documents.

The Clinical Monitors will visit the study sites at regular intervals during the study and after the study has completed, as appropriate. A clinical site monitoring plan will detail who performs the monitoring, how often, and the extent of review. It also will provide the monitoring strategy, with emphasis on participant safety, data integrity, and critical data and processes.

During these visits, CRFs, supporting documentation, and essential documentation related to the study will be reviewed and any discrepancies or omissions will be resolved.

Monitoring visits must be conducted according to the applicable ICH and GCP guidelines to ensure the protection of participant rights and well-being, protocol adherence, quality of data (accurate, complete, and verifiable), study treatment accountability, compliance with regulatory requirements, and continued adequacy of the study site and its facilities.

CONFIDENTIAL
The information contained herein may not be used, disclosed, or published without the written consent of Biogen MA Inc.

18.4. Study Funding

Biogen is the Sponsor of the study and is funding the study. All financial details are provided in the separate contracts between the institution, Investigator, and Biogen.

18.5. Publications

Details are included in the clinical trial agreement for this study.

19. FURTHER REQUIREMENTS AND GENERAL INFORMATION

19.1. External Contract Organizations

19.1.1. Contract Research Organization

A CRO will be responsible for administrative aspects of the study including but not limited to study initiation, monitoring, and data management. Before participants are screened at each study site, the CRO will review study responsibilities with the Investigators and other study site staff, as appropriate.

19.1.2. Interactive Response Technology

IRT will be used in this study. Before participants are screened or enrolled, the IRT vendor will provide each study site with the necessary training, a user manual, and access rights to the system.

19.1.3. Electronic Data Capture

Participant information will be captured and managed by study sites in source documents with study data variables entered in electronic CRFs by a web-based electronic data capture tool developed and supported by Medidata Rave and configured by a CRO.

All clinician-reported and caregiver-reported outcomes will be captured in an electronic format and managed by Cogstate.

19.1.4. Central Laboratories for Laboratory Assessments

A central laboratory has been selected by Biogen to analyze all standard hematology, blood chemistry, and urinalysis samples collected for this study.

The central laboratory will also receive, track and ship all blood samples for specialized [redacted], and anti-drug antibody testing [redacted]

Laboratories performing specialized testing will be identified in regulatory documentation. These laboratories will use appropriately validated or qualified assays to test study samples.

19.1.5. Central Facility for Other Assessments

All ECGs will be read and interpreted by a central reading facility selected by Biogen. Readings from this central facility will prevail over those conducted by the Investigator.

CONFIDENTIAL
The information contained herein may not be used, disclosed, or published without the written consent of Biogen MA Inc.

[redacted]

19.1.6. Neurocognitive Assessments

Biogen has selected a rater management group to establish rater qualification, study-specific training, and oversight. The study raters are required to complete qualifications steps and required training prior to administering study assessments. The rater management group will oversee the assessments per project-specific plans.

19.2. Study Committees

An independent DMC will be established to review safety data on an ongoing basis to ensure safe and proper treatment of participants. The DMC, based on the nature, frequency, and/or severity of an AE(s), may recommend that the study continue without modification or may recommend protocol modification(s), dose suspension, or dose termination.

The DMC charter will provide full guidance on the function and practices to be followed by the DMC.

19.3. Changes to Final Study Protocol

All protocol amendments must be submitted to the ethics committee and regulatory authorities if required by local law. Protocol modifications that affect participant safety, the scope of the investigation, or the scientific quality of the study must be approved by the ethics committee before implementation of such modifications to the conduct of the study. If required by local law, such modifications must also be approved by the appropriate regulatory agency prior to implementation.

However, Biogen may, at any time, amend this protocol to eliminate an apparent immediate hazard to a participant. In this case, the appropriate regulatory authorities will be notified subsequent to the modification.

In the event of a protocol modification, the ICF may require similar modifications (see Section 17).

19.4. Ethics Committee Notification of Study Completion or Termination

Where required, the regulatory authorities and ethics committees must be notified of completion or termination of this study, and sent a copy of the study synopsis in accordance with necessary timelines.

19.5. Retention of Study Data

The minimum retention time for study records will meet the strictest standard applicable to that study site, as dictated by any institutional requirements or local, national, or regional laws or regulations. Prior to proceeding with destruction of records, the Investigator must notify Biogen in writing and receive written authorization from Biogen to destroy study records. In addition, the Investigator must notify Biogen of any changes in the archival arrangements including but not limited to archival at an offsite facility or transfer of ownership if the Investigator leaves the study site.

19.6. Study Report Signatory

Biogen will designate 1 of the participating Investigators as a signatory for the study report. This determination will be made by several factors, including, but not limited to, the Investigator's experience and reputation in the studied indication, the Investigator's contribution to the study in terms of design, management, and/or participant enrollment, or by other factors determined to be relevant by Biogen. Biogen will follow all applicable local regulations pertaining to study report signatories.

CONFIDENTIAL
The information contained herein may not be used, disclosed, or published without the written consent of Biogen MA Inc.

20. REFERENCES

Alzheimer's Association. 2015 Alzheimer's Disease Facts and Figures. Alzheimers Dement. 2015;11(3):332+.

Alzheimer's Disease International. World Alzheimer Report 2015: The Global Impact of Dementia. London: Alzheimer's Disease International (ADI); 2015.

Birks J. Cholinesterase inhibitors for Alzheimer's disease. Cochrane Database Syst Rev. 2006(1):CD005593.

Bretz F, Pinheiro JC, Branson M. Combining multiple comparisons and modeling techniques in dose-response studies. Biometrics. 2005;61(3):738-48.

Hardy J, Selkoe DJ. The amyloid hypothesis of Alzheimer's disease: progress and problems on the road to therapeutics. Science. 2002;297(5580):353-6. Epub 2002 Jul 20.

[redacted]

[redacted]

McKhann GM, Knopman DS, Chertkow H, et al. The diagnosis of dementia due to Alzheimer's disease: recommendations from the National Institute on Aging-Alzheimer's Association workgroups on diagnostic guidelines for Alzheimer's disease. Alzheimers Dement. 2011;7(3):263-9.

McShane R, Areosa Sastre A, Minakaran N. Memantine for dementia. Cochrane Database Syst Rev. 2006(2):1-80.

[redacted]

Musiek ES, Holtzman DM. Three dimensions of the amyloid hypothesis: time, space and 'wingmen'. Nat Neurosci. 2015;18(6):800-6.

[redacted]

Serrano-Pozo A, Frosch MP, Masliah E, et al. Neuropathological alterations in Alzheimer disease. Cold Spring Harb Perspect Med. 2011;1(1):a006189.

Wahlund LO, Barkhof F, Fazekas F, et al. A new rating scale for age-related white matter changes applicable to MRI and CT. Stroke. 2001;32(6):1318-22.

CONFIDENTIAL
The information contained herein may not be used, disclosed, or published without the written consent of Biogen MA Inc.

21. SIGNED AGREEMENT OF THE STUDY PROTOCOL

I have read the foregoing protocol, "Randomized, Double-Blind, Placebo-Controlled, Parallel-Group Study to Assess the Safety, Tolerability, and Efficacy of BIIB092 in Subjects with Mild Cognitive Impairment due to Alzheimer's Disease or with Mild Alzheimer's Disease," and agree to conduct the study according to the protocol and the applicable ICH guidelines and GCP regulations, and to inform all who assist me in the conduct of this study of their responsibilities and obligations.

____

Investigator's Signature Date

____

Investigator's Name (Print)

____

Study Site (Print)

CONFIDENTIAL
The information contained herein may not be used, disclosed, or published without the written consent of Biogen MA Inc.

Biogen MA Inc.
225 Binney Street
Cambridge, MA 02142
United States

Biogen Idec Research Limited
Innovation House
70 Norden Road
Maidenhead Berkshire
SL6 4AY
United Kingdom

AMENDMENT SUMMARY

Biogen Protocol 251AD201

Randomized, Double-Blind, Placebo-Controlled, Parallel-Group Study to Assess the Safety, Tolerability, and Efficacy of BIIB092 in Subjects with Mild Cognitive Impairment due to Alzheimer's Disease or with Mild Alzheimer's Disease

Version 2

Date: 07 September 2018

EUDRA CT Number: 2017-002901-37

Version 2.0 of the protocol has been prepared for this amendment, which supersedes Version 1.

CONFIDENTIAL
The information contained herein may not be used, disclosed, or published without the written consent of Biogen MA Inc.

PRIMARY REASON FOR AMENDMENT

The primary reason for this amendment to Protocol 251AD201 is to update the list of allowed concomitant medications for subjects enrolled in this study, washout period of Alzheimer's disease (AD) medications, and to add the requirement for stable doses of key concomitant medications prior to and during Screening and for the duration of the study.

New text is shown in **bold** type; deleted text is shown with a ~~strikethrough~~.

<u>Section 8.2, Exclusion Criteria</u>

Now reads:

Candidates will be excluded from study entry if any of the following exclusion criteria exist at Screening, or at the timepoint specified in the individual criterion listed:

….

~~24.~~**26** Use of allowed medications for chronic conditions at doses that have not been stable for at least 4 weeks prior to Screening Visit 1 **and during the screening period up to Study Day 1.**~~,~~

27. ~~or use~~**Use** of AD medications (including but not limited to donepezil, rivastigmine, galantamine, tacrine, and memantine) at doses that have not been stable for at least 8 weeks prior to Screening Visit 1 **and during the screening period up to Study Day 1**.

~~25~~.**28.** Use of any medications that, in the opinion of the Investigator, may contribute to cognitive impairment, put the subject at higher risk for AEs, or impair the subject's ability to perform cognitive testing or complete study procedures. ~~Such medications include, but are not limited to, the following:~~

29. Use of the following medications:

- Long-acting benzodiazepines **(e.g., valium), except for sedation prior to MRI scans for those subjects requiring sedation and should not be administered within 24 hours prior to cognitive testing**.
- Short/medium-acting benzodiazepines (e.g., alprazolam, lorazepam, oxazepam, temazepam) except if used ~~on an as-needed basis~~**chronically for sleep and on a stable dose for 8 weeks prior to Screening Visit 1 and during the screening period up to Study Day 1, or used short term on an as-needed basis. May not be taken within 12 hours prior to cognitive testing**.

CONFIDENTIAL
The information contained herein may not be used, disclosed, or published without the written consent of Biogen MA Inc.

- ~~Short/medium acting benzodiazepines (e.g., alprazolam, lorazepam, oxazepam, temazepam)~~ **Sedating antihistamines** if taken within 12 hours prior to cognitive testing. **Nonsedating antihistamines (e.g., fexofenadine, cetirizine) are allowed.**

~~– Sedating antihistamines if used on a regular basis or if taken more than 3 times per week or if taken within 24 hours prior to cognitive testing. Nonsedating antihistamines (e.g., fexofenadine, cetirizine) are allowed.~~

- Anticonvulsants ~~used for treatment of seizures and anticonvulsants~~ that have significant effects on cognition **per the Investigator's opinion and/or anticonvulsants used for treatment of seizures**. Anticonvulsants with limited cognitive effects, such as lamotrigine, pregabalin, levetiracetam for treatment of pain, and other non-epilepsy indications are allowed **if, in the opinion of the Investigator, they are not producing sedation or contributing to cognitive impairment**. The subject must have been on a stable dose for 8 weeks prior to Screening Visit 1 **and during the screening period up to Study Day 1**.

- ~~Sedating antidepressants~~**Antidepressants that may, in the Investigator's opinion, affect the subject's cognition** (e.g., tricyclic antidepressants). Mild depression or depressive mood arising in the context of AD are not criteria for exclusion. Use of~~nonsedating~~ antidepressants is allowed if at stable doses for 8 weeks prior to Screening Visit 1 **and during the screening period up to Study Day 1**.

- Antipsychotics used on a regular basis~~. Low~~**, except for low** doses of atypical antipsychotics (e.g., risperidone, aripiprazole, or quetiapine) ~~are allowed~~**if used** on an as-needed basis **or if used at a stable dose for 8 weeks prior to Screening Visit 1 and during the screening period up to Study Day 1. The definition of "low doses" should be judged by the Investigator, and the Medical Monitor can be consulted if needed**.

…

~~27~~.**31.** Prior participation in any active or passive immunotherapy study targeting Aβ or tau, unless documentation of receipt of placebo is available.

32. Last administration of β-secretase inhibitors and γ-secretase inhibitors in a study within 3 months or 5 half-lives (whichever is longer) prior to Screening Visit 1unless documentation of receipt of placebo is available.

33. Participation in any study involving an investigational treatment targeting tau, unless documentation of receipt of placebo is available.

CONFIDENTIAL
The information contained herein may not be used, disclosed, or published without the written consent of Biogen MA Inc.

~~28.~~**34.** Participation within the 12 months prior to Screening Visit 1 in a study of any **other** agent(s) with a purported disease-modifying effect in AD ~~(e.g., β-secretase inhibitors, γ secretase inhibitors)~~, unless documentation of receipt of placebo is available.

35. [redacted]

Rationale: Overall, the eligibility criteria regarding medications was updated to better reflect the target population and to extend the requirement for stable doses through Study Day 1. Subjects are expected to continue on the stable doses of concomitant medications throughout the study treatment period.

The modifications in use of benzodiazepines, antihistamines, anticonvulsants, antidepressants, and antipsychotics while on study are not expected to represent any changes in the safety risk for subjects with AD participating in this study.

Benzodiazepines, antihistamines, anticonvulsants, antidepressants, and antipsychotics as well as medications with platelet anti-aggregant or anticoagulant properties are widely used to manage symptoms in the target patient population.

The restrictions in use of psychoactive medications that are currently included together with the Investigator's assessment and avoidance of concomitant medications that may contribute to cognitive impairment and interfere with cognitive assessments are expected to limit variability of data.

[redacted]

The 12-month washout period was changed to a 3 month or 5 half-lives (whichever is longer) washout period from the last administration for β-secretase inhibitors and γ-secretase inhibitors. Based on the current knowledge, these agents are not expected to represent any change to the safety risk or confounding residual efficacy for the subjects participating in this study after this period.

This change also affects Section 11.4.1.1, Allowed Concomitant Therapy, and Section 11.4.1.2, Disallowed Concomitant Therapy.

CONFIDENTIAL
The information contained herein may not be used, disclosed, or published without the written consent of Biogen MA Inc.

SUMMARY OF MAJOR CHANGES TO THE PROTOCOL

Changes to the protocol are presented chronologically. New text is shown in **bold** type; deleted text is shown with a ~~strikethrough~~.

Section 3, Synopsis

The Synopsis was revised to reflect changes made throughout the protocol.

Section 4.5, Overall Benefits and Risks Assessment

Change: Text was added regarding the benefits and risks to subjects participating in this clinical study.

Now reads:

4.5 Overall Benefits and Risks Assessment

Due to its eTau-lowering effects, BIIB092 has the therapeutic potential to prevent transmission of tau pathology in neurodegenerative disorders known as tauopathies, which include PSP and AD. The proposed indications under the current program of research are for use in patients with PSP and AD.

To date, 2 clinical studies have been completed with BIIB092. These include the first-in-human study, CN002001 (SAD study in healthy adult subjects) and CN002003 (MAD study in subjects with PSP). In addition to this study, 2 studies are ongoing: Study 251PP201 (formerly referred to as CN002004), an open-label extension study, and Study 251PP301 (formerly referred to as CN002012), a Phase 2b randomized controlled efficacy and safety study in subjects with PSP. Please refer to Section 4.3.2 for more information.

All available clinical benefit and risk information to date has been derived from studies in healthy volunteers or subjects with PSP. PSP is a rare neurodegenerative disease that results in a rapidly progressing, fatal movement disorder that includes cognitive and behavioral abnormalities. There are currently no approved or effective treatments for PSP. Nonclinical models support the anti-eTau mechanism as potentially efficacious in the treatment of PSP.

Binding to eTau, the murine antibody from which BIIB092 is derived, prevented tau transmission in nonclinical studies. Furthermore, the murine antibody from which BIIB092 was derived prevented tau-dependent behavioral and pathologic changes in a mouse model of tauopathy. Finally, BIIB092 reduced free eTau levels in the CSF of cynomolgus monkeys following a single IV administration. For additional details, please refer to Section 4.3.1. By binding eTau, it is expected that BIIB092 will prevent tau

CONFIDENTIAL
The information contained herein may not be used, disclosed, or published without the written consent of Biogen MA Inc.

transmission in diseases such as AD and PSP and thereby potentially provide therapeutic benefit.

The nonclinical toxicity profile of BIIB092 was used to determine the starting dose and eligibility criteria and to develop appropriate safety monitoring for BIIB092 studies. Clinical data from the completed first-in-human SAD study in healthy adult subjects (CN002001) demonstrated that single doses of BIIB092 up to 4200 mg in Caucasian subjects and up to 2100 mg in Japanese subjects were generally safe and well tolerated. The completed (MAD) study in subjects with PSP (CN002003) evaluated multiple doses of BIIB092 (150, 700, and 2100 mg) or placebo administered once every 4 weeks for 3 months. The available data suggest that multiple doses of BIIB092 up to 2100 mg are safe and well tolerated in subjects with PSP. Correspondingly, the current ongoing open-label, long-term extension, safety and tolerability study (251PP201) also suggests that multiple doses of BIIB092 up to 2100 mg are safe and well tolerated in subjects with PSP.

Frequent safety assessments will be utilized by the Sponsor/Medical Monitor and Investigators to determine whether dose modification, additional safety measures, or termination of the ongoing studies is required at any time. Serum immunogenicity sampling will be performed to monitor for the emergence of anti-drug antibodies. Thorough evaluation of safety monitoring procedures and of AEs and serious adverse events (SAEs) will be reviewed on an ongoing basis by the Sponsor's Medical Monitor and Global Pharmacovigilance representatives to monitor for any safety signals or trends.

In addition, an independent Data Monitoring Committee (DMC) will also be established for Studies 251PP201 and 251PP301 to monitor the benefit/risk profile of BIIB092.

For information on specific risk mitigation strategies, please refer to Section 10, Section 11.2.2, and Section 15.4.

A need exists for disease-modifying therapies for subjects with AD and those with PSP. The doses of BIIB092 currently being explored have the potential to benefit study subjects. Furthermore, the nonclinical efficacy profile and the evidence for anti-eTau activity, together with the benign safety profile, indicate that the balance of benefit to risk is likely to be favorable for study subjects.

Rationale: The additions to text on the topic of the benefits and risks to subjects participating in this clinical study have been extracted from the Investigational Medicinal Product Dossiers (IMPD) and added to the protocol at the request of the Swedish Medical Products Agency and now been have added it to global protocol.

CONFIDENTIAL
The information contained herein may not be used, disclosed, or published without the written consent of Biogen MA Inc.

Section 5, Schedule of Activities

Change:

The screening period window was increased up to 90 days with Sponsor approval.

The list of assessments to be performed, in case the screening period was increased up to 90 days, was modified.

The timing of clinical assessments at Visit S3 was updated.

The timing window for coagulation tests after randomization was updated.

The timing window for the electrocardiogram (ECG) performed after the end of infusion on Study Day 1 was updated.

The fasting requirement for subjects before blood samples for hematology, clinical chemistry and urinalysis laboratory assessments was removed.

The timing of the postbaseline Clinical Dementia Rating Scale (CDR) assessment was updated.

The timing of the Alzheimer's Disease Assessment Scale-Cognitive (13 item) [ADAS-Cog 13] and Columbia - Suicide Severity Rating Scale (C-SSRS) assessments during the screening period was updated.

The reporting of serious AEs and concomitant medication was updated.

CONFIDENTIAL
The information contained herein may not be used, disclosed, or published without the written consent of Biogen MA Inc.

Now reads:

Table 1: Schedule of Activities

Study Week	Baseline Screening Period[1,2] within 65 days[3] of Day 1			Placebo-controlled Period[4,5,6]																					FU[7]
					4	8	12	16	20	24	28	32	36	40	44	48	52	56	60	64	68	72	76 EOT	78/ EOS or ET[8]	90
Study Day	S1	S2	S3[9]	1	29 ±7	57 ±7	85 ±7	113 ±7	141 ±7	169 ±7	197 ±7	225 ±7	253 ±7	281 ±7	309 ±7	337 ±7	365 ±7	393 ±7	421 ±7	449 ±7	477 ±7	505 ±7	533 ±7	547 ±7	631 ±7
Study day infusion				X	X	X	X	X	X	X	X	X	X	X	X	X	X	X	X	X	X	X	X		
Initial screening consent (optional) [10]	X																								
Full informed consent[11]	X																								
Randomization				X																					
Eligibility criteria	X	X	X	X																					
NIA-AA criteria review	X																								
Medical history	X	X	X	X																					
Body weight	X			X	X	X	X			X				X			X			X			X	X	X
Height	X																								
Pregnancy test[12]	X			X	X	X	X	X	X	X	X	X	X	X	X	X	X	X	X	X	X	X	X	X	X
Follicle-stimulating hormone[13]	X																								
Alcohol/drug screen	X																								
HbA_{1c}	X																								
HIV[14]/ hepatitis tests	X																								
[redacted]																									
Physical examination	X			X			X			X						X						X		X	X
Neurological examination	X			X			X			X						X						X		X	X

CONFIDENTIAL
The information contained herein may not be used, disclosed, or published without the written consent of Biogen MA Inc.

Study Week	Baseline Screening Period[1,2] within 65 days[3] of Day 1			Placebo-controlled Period[4,5,6]																					FU[7]
					4	8	12	16	20	24	28	32	36	40	44	48	52	56	60	64	68	72	76 EOT	78/ EOS or ET[8]	90
Study Day	S1	S2	S3[9]	1	29 ±7	57 ±7	85 ±7	113 ±7	141 ±7	169 ±7	197 ±7	225 ±7	253 ±7	281 ±7	309 ±7	337 ±7	365 ±7	393 ±7	421 ±7	449 ±7	477 ±7	505 ±7	533 ±7	547 ±7	631 ±7
12-lead paper ECG[16]	X			X			X			X						X						X		X	X
Vital signs[17]	X			X	X	X	X	X	X	X	X	X	X	X	X	X	X	X	X	X	X	X	X	X	X
Hematology/ clinical chemistry/ urinalysis~~[8]~~	X			X			X			X						X						X		X	X
Blood sample for anti-BIIB092 Ab[4]				X	X					X						X							X		X
Blood sample for BIIB092 concentration				X	X~~[19]~~[18]	X	X~~[19]~~[18]	X	X	X~~[19]~~[18]	X					X~~[19]~~[18]			X				X~~[19]~~[18]		X
[redacted]																									
Blood sample for plasma and serum biomarkers[4]				X				X								X			X				X		
[redacted]																									
Brain MRI~~[21]~~[20]		X									X						X							X	
[redacted]																									
CDR~~[26]~~[25]	X									X							X							X	
NPI-10			X							X							X							X	
ISLR	X																								
ISLT	X		X				X				X			X				X			X			X	

Study Week	Baseline Screening Period[1,2] within 65 days[3] of Day 1			Placebo-controlled Period[4,5,6]																					FU[7]
					4	8	12	16	20	24	28	32	36	40	44	48	52	56	60	64	68	72	76 EOT	78/ EOS or ET[8]	90
Study Day	S1	S2	S3[9]	1	29 ±7	57 ±7	85 ±7	113 ±7	141 ±7	169 ±7	197 ±7	225 ±7	253 ±7	281 ±7	309 ±7	337 ±7	365 ±7	393 ±7	421 ±7	449 ±7	477 ±7	505 ±7	533 ±7	547 ±7	631 ±7
DKEFS Category Fluency and Letter Fluency tests	X		X				X				X			X				X			X			X	
DSST	X		X				X				X			X				X			X			X	
Trails A	X		X				X				X			X				X			X			X	
eCog (39-item version)			X								X							X						X	
ADAS-Cog- 13[26]		X	X							X							X							X	
FAQ			X							X							X							X	
ADCS-ADL			X							X							X							X	
C-SSRS[26]		X	X							X								X						X	
MMSE	X						X			X				X			X				X			X	
RUD-Lite			X						X							X					X			X if ET	
ZBI			X						X							X					X			X if ET	
QoL-AD			X						X							X					X			X if ET	
AE reporting				Monitor and record continuously beginning on Day 1 at the start of dosing																					
[redacted]																									
**SAE reporting**	**Monitor and record continuously throughout the study**																								
**Concomitant therapy and procedures reporting**	**Monitor and record continuously throughout the study**																								

Ab = antibodies; AD = Alzheimer's disease; ADAS-Cog **13** = Alzheimer's Disease Assessment Scale-Cognitive (13 item); ADCS-ADL = Alzheimer's Disease Cooperative Study – Activities of Daily Living; AE = adverse event; [redacted]; CDR = Clinical Dementia Rating Scale; [redacted]

[redacted]; C-SSRS = Columbia - Suicide Severity Rating Scale; DBP = diastolic blood pressure; DKEFS = Delis-Kaplan Executive Function System; DSST = Digit Symbol Substitution Test; ECG = electrocardiogram; eCog = Everyday Cognition; EOS = end of study; EOT = end of treatment; ET = early termination; FAQ = Functional Activities Questionnaire; FU = follow up; HbA1c = glycosylated hemoglobin; HIV = human immunodeficiency virus; **ICF = informed consent form;** [redacted]; ISLR = International Shopping List Test Delayed Recall; ISLT = International Shopping List Test Immediate Recall; [redacted]; MMSE = Mini-Mental State Examination; MRI = magnetic resonance imaging; NIA-AA = National Institute on Aging – Alzheimer's Association; NPI-10 = Neuropsychiatric Inventory-10; [redacted]; QoL-AD = Quality of Life in Alzheimer's Disease; [redacted]; RUD-Lite = Resource Utilization in Dementia-~~Light~~**Lite** Version; SAE = serious adverse event; SBP = systolic blood pressure; Trails A = Trail Making Test Part A; ZBI = Zarit Burden Interview

1 The screening process will generally involve up to 3 visits, and most screening procedures will be performed within these designated visits (S1-S3). However additional screening visits may be needed for some procedures, e.g., MRI [redacted]. In addition, it may sometimes be necessary for subjects to return for repeat assessments (e.g., if the MRI scan does not pass the quality control process). Brain MRI (S2) will only be performed after the subject has met the eligibility criteria and has acceptable laboratory tests from Visit S1. [redacted]

2 The clinical assessments performed during Visit S1 are to be administered in the order specified: 1) MMSE; 2) neuropsychological tests in the following order: ISLT, DSST, Trails A, Category Fluency and Letter Fluency tests from the DKEFS, ISLR; 3) CDR. Note that the Visit S1 results from CDR, ISLT/ISLR, and MMSE will be used to determine subject eligibility; Visit S3 ISLT assessments will provide baseline measures. At Visit S3, the clinical assessments will be administered as follows: 1) neuropsychological tests in the following order: ISLT, DSST, Trails A, Category Fluency and Letter Fluency tests from the DKEFS; 2) ADAS-Cog 13; 3) QoL-AD.

3 ~~The nominal~~ **It is recommended that all the screening procedures be completed within** 65~~-day~~ **days; however, the overall** screening period may be ~~increased with the permission of~~ **extended up to 90 days in consultation with** the Medical Monitor **in advance and provided that written documentation is received from the Sponsor or delegate (**[redacted]**)**.The following screening assessments should be repeated if the screening period is >65 days: confirmation of eligibility criteria, abbreviated medical history, **and** physical examination~~, ECG, hematology, clinical chemistry, and serum pregnancy test (women of childbearing potential)~~. In addition, some cognitive **and /or safety** assessments may require repeating depending on the duration of the screening window extension, in discussion with the Medical Monitor.

4 All assessments and blood collection during dosing days will be performed prior to infusion, unless otherwise specified. Doses should be administered at least 21 days apart.

5 The visits at Weeks 32, 36, 44, and 64 may qualify for home visits, if appropriate; see Section 7.1 for details.

6 Prior to initiation of and/or a change in AD medication, a subject should have an unscheduled visit for assessment of AEs/~~serious~~ **S**AEs and concomitant medications and key clinical assessments, including, at a minimum, the CDR, MMSE, ISLT, ADAS-Cog 13, ADCS-ADL, and FAQ.

7 Subjects who complete the Treatment Period and do not enter an extension study ~~(separate protocol, to be initiated at the Sponsor's discretion)~~ are to return to the study site for a Follow-up Safety Visit at Week 90. Subjects who discontinue study treatment and withdraw from the study early are to have a Follow-up Safety Visit 14 weeks after the final dose. Subjects who discontinue study treatment prematurely are to return to the study site for a Follow-up Safety Visit 14 weeks after receiving the last dose of study treatment.

8 All subjects who discontinue study treatment prematurely will be asked to remain in the study and continue all protocol-specified visits and procedures. These subjects should be encouraged to attend at least the Week 24, Week 52, and Week 78 visits, depending on when treatment was discontinued, and undergo all the scheduled procedures. At a minimum, these visits should include assessment of AEs/SAEs and concomitant medications and key clinical assessments, including at least the CDR, MMSE, ISLT, ADAS-Cog 13, ADCS-ADL, and FAQ. Subjects who discontinue study treatment prematurely will also be asked to return to

CONFIDENTIAL
The information contained herein may not be used, disclosed, or published without the written consent of Biogen MA Inc.

the study site for a Follow-up Safety Visit 14 weeks after receiving the last dose of study treatment (see Table 1 for the schedule of assessments). Subjects who withdraw from the study prematurely are to return to the study site for an ET visit and ET assessments. If the withdrawn subject has discontinued treatment within 3 months of the previous primary efficacy assessment (CDR) and no significant changes in cognitive status are suspected by the Investigator, the clinical efficacy assessments specified in the ET visit are not required; the study site should notify the Sponsor in such cases. An MRI scan should be performed if withdrawal occurs ≥6 months after the previous MRI. Blood for biomarker analysis should be collected if the subject withdraws ≥4 weeks after the previous sample was collected. [redacted]

[9] Visit S3 **all clinical assessments** must be scheduled within 7 days before **Study** Day 1 **or performed at Study Day 1 before randomization,** [redacted]

[10] Subjects **and the subjects' partners/informants** or their legally authorized representatives~~, and the subjects' partners/informants~~**in countries where applicable laws allow**, may sign this optional form for an initial screening which allows administration of the CDR, MMSE, ISLT/ISLR, DKEFS Category Fluency and Letter Fluency tests, DSST, and Trails A to determine eligibility based on cognitive assessments. The order of assessments is as specified in footnote 2.

[11] All subjects **and the subjects' partners/informants** or their legally authorized representatives~~, and the subjects' partners/informants~~**in countries where applicable laws allow**, must sign this full informed consent, including those who have previously signed the optional initial screening consent and have met the eligibility criteria based on cognitive assessments.

[12] Pregnancy testing is required for women of childbearing potential only; for these subjects, a serum pregnancy test is to be performed at Screening and EOS (or ET) Visits, and a urine pregnancy test is to be performed at every dosing visit.

[13] Women who report postmenopausal status at Visit S1 must have a follicle-stimulating hormone test at that visit to confirm that they are not of childbearing potential.

[14] To be performed based upon Investigator assessment of HIV risk factors. The requirement for testing during Screening may be omitted if it is not permitted by local regulations.

[redacted]

[16] Triplicate 12-lead (paper) ECGs will be obtained at each specified timepoint. ECGs are to be performed in triplicate, with each ECG performed about 1 minute apart and before blood draws on appropriate days. In addition, triplicate ECGs will also be performed ~~15 minutes~~**1 hour** after the end of infusion on **Study** Day

CONFIDENTIAL
The information contained herein may not be used, disclosed, or published without the written consent of Biogen MA Inc.

1 only. Each ECG must be performed after the subject has been resting (supine) for 10 minutes. ECGs will be read by a central reading facility. Clinical significance of potential ECG abnormalities will be determined by the Investigator.

[17]Vital signs will include SBP, DBP, heart rate, body temperature, and respiratory rate and will be measured with the subject supine and after the subject has been resting for at least 10 minutes. In addition, blood pressure and heart rate will be measured after the subject has been standing for ≥2 minutes at the following timepoints: preinfusion and 1 hour (±15 minutes) after the end of infusion on Days 1, 29, 57, and 85. Three separate SBP/DBP readings at least 15 minutes apart will be made at the Screening Visit to determine eligibility. Single vital sign readings will be obtained at other timepoints.

~~[18]Fasting is required prior to safety laboratory tests.~~

[~~19~~18]Two samples must be obtained: 1 sample taken before infusion and 1 sample taken at ≤10 minutes after the end of infusion.

[~~20~~19]The results are not required for randomization.

[~~21~~20]Brain MRI may include, but will not be limited to, the following sequences: 3D T1 weighted, fluid attenuated inversion recovery, T2* weighted, diffusion weighted imaging, and proton density/T2 weighted MRI. For further details on MRI sequences, please see the procedural manual for MRI. To occur after cognitive testing has concluded for the ~~s~~Screening ~~p~~Period. After randomization, the visit date for this MRI can vary by ±10 days from the specified visit day.

[redacted]

[~~26~~25]~~Postbaseline~~ **It is recommended that postbaseline** CDR assessments ~~should~~ be conducted ~~within ±1 hour of~~ **at** the **same** time **of day** at which the baseline assessment was performed **to avoid diurnal violation**. The rater who conducts the CDR for a subject/~~partner~~ **informant** cannot complete any other rating scales for that same subject/partner or be the study site coordinator and will be blinded to all other study-related data.

[26]During the screening period, ADAS-Cog 13 and C-SSRS are scheduled at Visit S2; however, they may be performed at Visit S1, if more convenient per site and subject's availability. If ADAS-Cog 13 and C-SSRS assessments are performed at Visit S1, all other clinical assessments should be performed before ADAS-Cog 13, followed by C-SSRS.

[redacted]

Rationale:

In footnote #3 and footnote #22 (*now footnote #21*), text was added to clarify that the screening period may be extended to 90 days, if needed, [redacted], pending the Sponsor's approval.

Footnote #3 was modified to clarify the assessments that are to be repeated if the screening period is increased to 90 days.

In footnote #9, the timing of clinical assessments at Screening Visit 3 was updated to provide flexibility to be performed at Study Day 1, in case of site's and/or subject's availability, [redacted].

In footnote #15, the timing window was increased to reduce burden on subjects and to allow appropriate time for review of the coagulation tests results. Additional text was added to clarify that the repeats test may be performed locally.

Footnote #16 was updated to increase the timing window and facilitate performing the ECG after the end of infusion on Study Day 1, now timed with vital sign assessment.

Footnote #18 (Version 1) was deleted. No safety concerns are expected with removal of the fasting requirement. It would be difficult to confirm compliance with the requirement of fasting across the study and the number of protocol deviations for blood sample collection. Furthermore, cognitive testing and, particularly, the primary efficacy endpoint, the CDR sum of boxes, are often performed on the same visit as the collection of blood samples for hematology and clinical chemistry laboratory assessments. Requiring this elderly subject population to fast before performing cognitive assessment tests may increase the variability in the data collected at these visits.

In footnote #25, the timing of the postbaseline CDR assessments was updated to allow additional flexibility in the schedule of study visits and reduce burden on subjects.

The timing of ADAS-Cog-13 and C-SSRS assessments during the screening period was updated to allow some flexibility and therefore reduce burden on subjects (footnote # 26 was added).

Clarification was made to the serious AE and concomitant medication reporting in the Schedule of Activities table, that it will be collected throughout the study, including during the screening period and made consistent with Section 15.3.3, Serious Adverse Events.

This change also affects Section, 7.1, Study Overview; Section 7.2, Study Duration for Subjects; Section 9.1, Screening; Section 13.1, Clinical Efficacy Assessments; Section 14.2, Laboratory Safety Assessments; and Section 15.3.1, Adverse Events.

CONFIDENTIAL
The information contained herein may not be used, disclosed, or published without the written consent of Biogen MA Inc.

Section 6, Study Objectives and Endpoints

Change: An update was made to primary safety endpoints, pharmacokinetic (PK) and pharmacodynnamic objectives and endpoints.

Now reads:

Primary Objective	Primary Endpoints
To evaluate the safety and tolerability of BIIB092 in subjects with MCI due to AD or with mild AD	Incidence of AEs **and** ~~/serious AEs (~~SAEs~~)~~ **from Baseline to the end of the study treatment period.**~~; incidence of abnormalities and changes from Baseline over time in laboratory safety assessments (including clinical chemistry, hematology, and urinalysis), vital signs, and 12-lead electrocardiograms (ECGs); and changes in physical examination findings.~~

Exploratory Objectives	Exploratory Endpoints
……	
To assess BIIB092 ~~concentrations~~ **PK** in serum ~~following multiple doses~~ **in subjects with MCI due to AD or with mild AD.**	~~Changes from, Baseline over time up to Week 90 in serum BIIB092 concentration~~ **Trough serum BIIB092 concentrations and end-of-infusion serum BIIB092 concentrations from the samples collected at the visits indicated in the schedule of activities.**
[redacted]	[redacted]
…..	
[redacted]	[redacted]

Rationale: The primary endpoint text was updated to facilitate handling of data disclosed to ClinicalTrials.gov and other registries under transparency requirements.

The exploratory PK objectives and endpoints were updated to streamline the generated data and PK evaluation. For the PK evaluation, the actual drug levels are used, not the changes from baseline.

[redacted]

[redacted]

<u>Section 8.1, Inclusion Criteria</u>

Change:

The eligibility criteria text related to informed consent was updated.

The International Shopping List Test Immediate Recall (ISLT) or International Shopping List Test Delayed Recall (ISLR) score requirement was modified.

Now reads:

To be eligible to participate in this study, candidates must meet the following eligibility criteria at Screening, or at the timepoint specified in the individual eligibility criterion listed:

1. Ability of the subject ~~or his/her informant/study partner and/or legally authorized representative~~ to understand the purpose and risks of the study and provide signed and dated informed consent and authorization to use confidential health information in accordance with national and local subject privacy regulations. **In countries where applicable laws allow, the participant's informant/study partner and/or legally authorized representative may provide informed consent in lieu of the subject's signature.**

   …..

5. Must meet all of the clinical criteria for MCI due to AD or mild AD according to the NIA-AA [McKhann 2011], and in addition must have the following at Screening Visit 1:

   – ISLT or ISLR score ~~<~~1 SD **below** ~~of~~ the age-adjusted normative mean

   – CDR global score of 0.5 for MCI due to AD or 0.5 or 1 for mild AD

   – MMSE score of 22 to 30 (inclusive)

   – CDR Memory Box score of ≥0.5

……

Rationale: The text was updated to clarify that applicable laws will be followed in case of subject's loss of capacity of consent.

The ISLT or ISLR score was clarified as per recommendation from the Cogstate Science Team.

This change affects Section 5, Schedule of Activities (Footnote #10 and Footnote #11), Section 9.1, Screening, Section 15, Safety Definitions, Recording, Reporting, and Responsibilities and Section 17.3, Subject Information and Consent.

<u>Section 8.2, Exclusion Criteria</u>

Change: The eligibility criterion regarding vaccination was updated.

Now reads:

~~26~~**.30**. Vaccinations within ~~7~~**10** days prior to randomization (Study Day 1).

Rationale: Exclusion criterion #26 (*now exclusion criterion #30*), was modified to exclude vaccinations within 10 days prior to randomization to be consistent with allowed vaccinations during the study as outlined in Section 14.1.1.1, Allowed Concomitant Therapy.

<u>Section 9.1, Screening</u>

Change: Rescreening requirements for subjects were updated.

Now reads:

Subjects who fail screening may be rescreened once at the Sponsor's discretion, [redacted] **MMSE**, hepatitis B or C criteria, **having a CDR global score >1**, or abnormal

MRI findings. **Note: Subjects with a CDR global score of 0 but who meet the other entry criteria may repeat the screening after 6 months from the initial evaluation.**

Rationale: Screening failures due to failed key clinical assessments will not be allowed to rescreen. Rescreening for subjects who meet entry criteria except having a normal CDR global score of 0 is allowed to capture subjects who are at risk of cognitive decline over the following 6 months.

Section 9.2, Randomization

Change: [redacted]

Now reads:

[redacted]

Rationale: [redacted]

Section 11.2.2, Infusion Interruption

Change: Additional text related to infusion time, use of premedication, and management of infusion-related reactions was added.

Now reads:

The IV administration infusion time for all treatment groups is 1 to 2 hours at approximately 100 mL/hour. No premedications should be used prior to the start of study treatment infusion unless discussed with the Medical Monitor in advance and written documentation is received from Biogen authorizing the use of the premedication.

- If any mild or moderate infusion-related reactions occurs during an infusion, ~~the infusion should be slowed or interrupted, and supportive treatment should be instituted. Upon resolution of symptoms, if the infusion had been slowed, the original infusion rate may be resumed;~~ **the infusion may be slowed or interrupted and appropriate treatment per local standards of care may be given at the discretion of the Investigator (or designee). Based on the clinical response, the Investigator or designee will determine if the infusion may be resumed/continued in consultation with the Medical Monitor as needed. I**~~i~~f the infusion is

resumed/continued~~had been interrupted~~, the infusion **rate** ~~may be restarted at a rate that does~~ **should** not exceed the original infusion rate ~~.~~(~~s~~See the DHA for infusion rate information~~.~~)**.**

- If a severe infusion-related reaction occurs during an infusion, the subject should be permanently discontinued from treatment~~. Severity~~**and appropriate supportive care must be initiated in accordance with local practice.**

Criteria for determining the severity of events ~~is~~**are** described in Section 15.2.3.

Discussion with the Medical Monitor can occur as needed, and it should not delay management of the medical emergency.

Refer to Section 15.3 for reporting of AEs and Section 10 for discontinuation of study treatment.

Rationale: Additional text was added to provide details on administration of BIIB092 and management of infusion reactions, should a subject experience an infusion reaction event during the study. This change is provided at the request of the Swedish Medical Products Agency. Because this change relates to clarification of safety measures, it is also made available to all the participating sites within the global protocol.

<u>Section 13.2, Pharmacokinetic Assessments</u>

Change: The methodology of PK assessments was updated.

Now reads:

Serum PK parameters of BIIB092 **to** ~~will~~ be ~~calculated using a nonlinear mixed effects approach. These parameters~~ **assessed** may include, but will not be limited to, the following:

- Maximum **observed** concentration **(C_{max})**
- ~~Area under the concentration-time curve~~ **End of infusion concentration**

[redacted]

Rationale: The text was updated to make the analysis methodology compatible with the data to be generated in this study. This study will only inform about the peak and trough concentrations, which will not be enough for a nonlinear mixed effects model.

A nonlinear mixed effects model is planned as a meta-analysis, combining data from multiple other studies, and will be conducted and reported separately.

CONFIDENTIAL
The information contained herein may not be used, disclosed, or published without the written consent of Biogen MA Inc.

Section 15.2.1, Investigator Assessment of Events

Change: [redacted]

Now reads:

[redacted]

- [redacted]
- [redacted]
- [redacted]

Rationale: [redacted]

[redacted]

[redacted]

Section 15.4.3.1, Unblinding for Medical Emergency

Change: The text regarding different ways of accessing the interactive response technology (IRT) system was modified.

Now reads:

In a medical emergency when knowledge of the subject's treatment assignment may influence the subject's clinical care, the Investigator **or appropriate designee** may access the subject's treatment assignment ~~by~~**in the** IRT **system by accessing the internet or using a phone-based interface**. **Further information about the IRT unblinding function or 24-hour, 7-day-a-week support contact information is available in the IRT manual for the study.**

CONFIDENTIAL
The information contained herein may not be used, disclosed, or published without the written consent of Biogen MA Inc.

The Investigator must document the reasons for unblinding in the subject's source documents. The Investigator is strongly advised not to divulge the subject's treatment assignment to any individual not directly involved in managing the medical emergency, or to personnel involved with the analysis and conduct of the study.

The Investigator can contact Biogen or its designee to discuss such situations, but such a discussion should not delay management of the medical emergency. The Investigator should inform Biogen or its designee as soon as possible if unblinding occurs.

Rationale: This change was made to clarify how to access the IRT system in the case of unblinding for a medical emergency and that unblinding for a medical emergency can be completed at any time. This change is provided at the request of the Swedish Medical Products Agency. Because this change relates to clarification of the process of unblinding for a medical emergency, it is also made available to all the participating sites within the global protocol.

Section 16.9, Interim Analyses

Change: Text regarding review by an independent team of interim analysis data for the purpose of future study planning was added.

Text regarding the interim analyses was modified.

Now reads:

Interim analyses may be performed after ~~50% to 70% of, or all,~~ **50% to 100% of** subjects have completed the Week 52 visit (or discontinued) for the purpose of future study planning and**/or** futility analyses. In order to maintain the treatment blind, an independent group external to Biogen that will not be involved in the conduct of the study after unblinding will perform the interim analyses. This independent group will present the unblinded interim analyses to the DMC for review. **After the DMC review, a small internal independent team (separate from the study team) may review the unblinded results for the purpose of future study planning under a study integrity charter.** No Type I error adjustment will be made. No changes will be made to this study based on the interim analysis results.

Rationale: The text was added to clarify how unblinded outputs from interim analysis that may support clinical study design of future studies will be evaluated. A small internal team independent from the study team will be involved in this evaluation to avoid any influence of this assessment on study conduct.

The text regarding interim analyses was modified to clarify that one or more interim analyses may be performed when between 50% and 100% of subjects have completed the Week 52 visits (or discontinued).

CONFIDENTIAL
The information contained herein may not be used, disclosed, or published without the written consent of Biogen MA Inc.

SUMMARY OF MINOR CHANGES TO THE PROTOCOL

The following minor changes were made to the protocol, as appropriate:

- The version number and date were updated throughout the protocol.
- On the Sponsor Signature Page, the signatory for this protocol was changed.
- Resource Utilization in Dementia (RUD)-Light was corrected to RUD-Lite throughout the protocol.
- [redacted]
- ADAS-Cog-13 was updated to ADAS-Cog 13, throughout the protocol.
- Section 2, List of Abbreviations was updated.
- In Section 8.2, Exclusion Criteria, eligibility requirement related medical history was clarified (exclusion criterion 6 was updated and exclusion criterion 7 was called out to be a separate criterion.
- In Section 8.2, Exclusion Criteria #9 (now reads #10), eligibility requirement related to brain magnetic resonance imaging (MRI) was updated.
- In Section 8.2, Exclusion Criteria, eligibility requirement related to impaired liver and renal function were separately called out for clarification) now reads as exclusion criteria #20 and #21).
- In Section 8.2, Exclusion Criteria #38 (now reads #45), eligibility requirement related to enrollment any other interventional clinical study was modified.
- In Section 11.5, Continuation of Treatment, text was updated to clarify the planned long-term extension of this study. This changes also affects Section 5, Schedule of Activities (footnote 7), and Section 7.2, Study Duration for Subjects.
- In Section 12.1, BIIB092, the amount per vial and details regarding excipients/components were removed.
- In Section 15, Safety Definitions, Recording, Reporting, and Responsibilities, an administrative change was made to notify Biogen instead of Quintiles in case of a safety event. This also affects Section, 19.1.1, Contract Research Organization.
- In Section 19.1.3, Electronic Data Capture, text was modified to clarify the Sponsor's definition of source data in the study.

CONFIDENTIAL
The information contained herein may not be used, disclosed, or published without the written consent of Biogen MA Inc.

- In Section 17 and Section 19, reference to Quintiles was updated to Contract Research Organization (CRO) to avoid a protocol amendment if there is a change in vendor.
- Typographical errors and formatting were corrected.

CONFIDENTIAL
The information contained herein may not be used, disclosed, or published without the written consent of Biogen MA Inc.

Biogen MA Inc.
225 Binney Street
Cambridge, MA 02142
United States

Biogen Idec Research Limited
Innovation House
70 Norden Road
Maidenhead Berkshire
SL6 4AY
United Kingdom

AMENDMENT SUMMARY

Biogen Protocol 251AD201

Randomized, Double-Blind, Placebo-Controlled, Parallel-Group Study to Assess the Safety, Tolerability, and Efficacy of BIIB092 in Subjects with Mild Cognitive Impairment due to Alzheimer's Disease or with Mild Alzheimer's Disease

Version 3

Date: 29 March 2019

EUDRA CT Number: 2017-002901-37

Version 3 of the protocol has been prepared for this amendment, which supersedes Version 2.

CONFIDENTIAL
The information contained herein may not be used, disclosed, or published without the written consent of Biogen MA Inc.

PRIMARY REASON FOR AMENDMENT

The primary reason for this amendment to Protocol 251AD201 is to add a dose-blinded long-term extension (LTE) period of approximately 3 years. The primary purpose of the LTE is to obtain long-term safety and tolerability information on BIIB092 and further explore its effects on immunogenicity, disease progression, and additional clinical and health outcomes.

New text is shown in **bold** type; deleted text is shown with a ~~strikethrough~~.

Section 7.1, Study Overview

Now reads:

…

The study **consists of a double-blind, placebo-controlled period and a dose-blinded LTE period. The total duration for participants who complete the placebo-controlled period and do not enter the LTE period will be approximately 99 weeks. For participants who complete both the placebo-controlled period and the LTE period, the total study duration will be approximately 247 weeks. The placebo-controlled period** comprises a Screening Period of ~~up to~~ **approximately** 9 weeks (65 days), a Treatment Period of 76 weeks, **and for participants not entering the LTE period,** an End-of-Study (EOS) Visit at Week 78 and a Follow-up Safety Visit at Week 90, or approximately 14 weeks after the last dose of study treatment. However, the ~~overall s~~Screening **Period for the placebo-controlled** period may be extended up to 90 days in consultation with the Medical Monitor in advance and provided that written documentation is received from the Sponsor or delegate (e.g., for logistical issues such as PET radioligands). **At the end of the placebo-controlled period, participants who meet the LTE entry criteria may enter the dose-blinded LTE period. The LTE period comprises an LTE Screening Period of approximately 4 weeks starting at Week 76, a 144-week Treatment Period, an EOS Visit at Week 226, and a Follow-up Safety Visit at Week 238, or approximately 14 weeks after the last dose of study treatment.**

…

During the LTE period, participants who were randomized to receive BIIB092 during the placebo-controlled period will continue to receive BIIB092 at the dose they were randomly assigned. Participants receiving placebo during the placebo-controlled period will receive BIIB092 at the high dose (2000 mg) once every 4 weeks during the LTE period. The BIIB092 dose concentration (i.e. low, medium, or high) to which participants are assigned to receive during the LTE period may be changed based on emerging data from the BIIB092 clinical development program.

The visits at Weeks 84, 88, 96, 112, 116, 120, 136, 140, 144, 160, 164, 168, 184, 188, 192, 208, 212, and 216 of the LTE period may be conducted as home visits, if the Investigator is in

agreement and appropriate services are available to perform the required study procedures and adequately monitor for potential safety events. These are study visits that do not require extensive assessments, e.g., administration of clinical scales or the conduct of [redacted], MRI, [redacted]. However, if the participant experienced a clinically significant infusion reaction during the first 24 weeks of the LTE period, then the BIIB092 infusion should not be administered in the home unless previously approved by the Investigator.

Throughout the study, all participants should be observed and monitored by study staff for a minimum of 1 hour after the end of an infusion. Participants who experience an AE or SAE related to BIIB092 infusion should remain at the site or be sent to an inpatient monitoring facility until the Investigator has determined that these event(s) has resolved or do not require further monitoring at the site or in an inpatient setting.

Investigators, study staff (except for a designated Pharmacist/Technician), and study ~~subjects~~**participants** and their families, caregivers, **and** legal representatives will be blinded to the ~~subjects'~~**participants'** randomized treatment assignments **and, during the LTE period, the BIIB092 dose**.

[redacted]

The schedule of study assessments is presented in Section 5.

Figure 1: Study ~~Schematic~~**Design**

Deleted Figure:

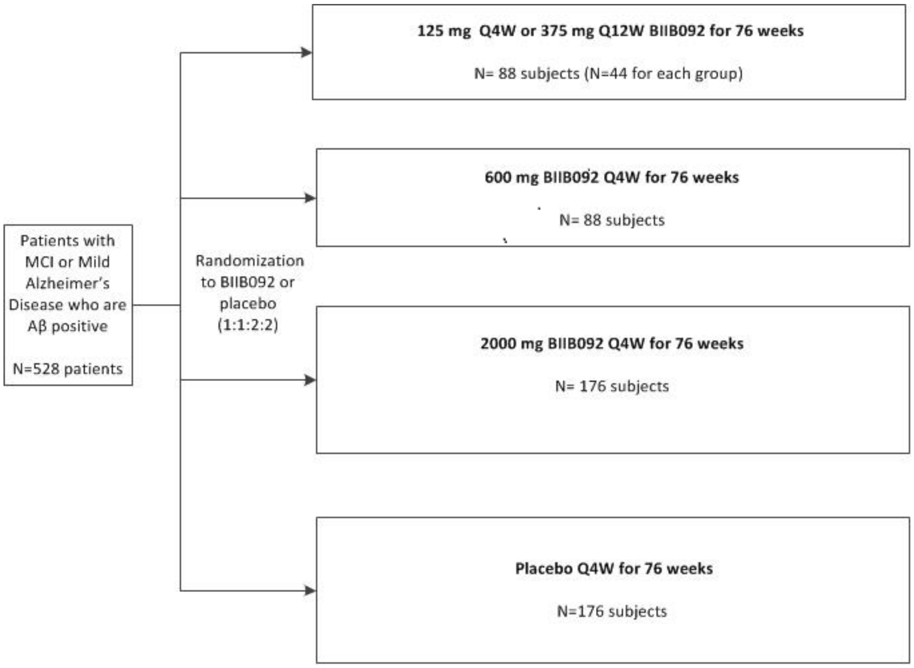

Inserted Figure:

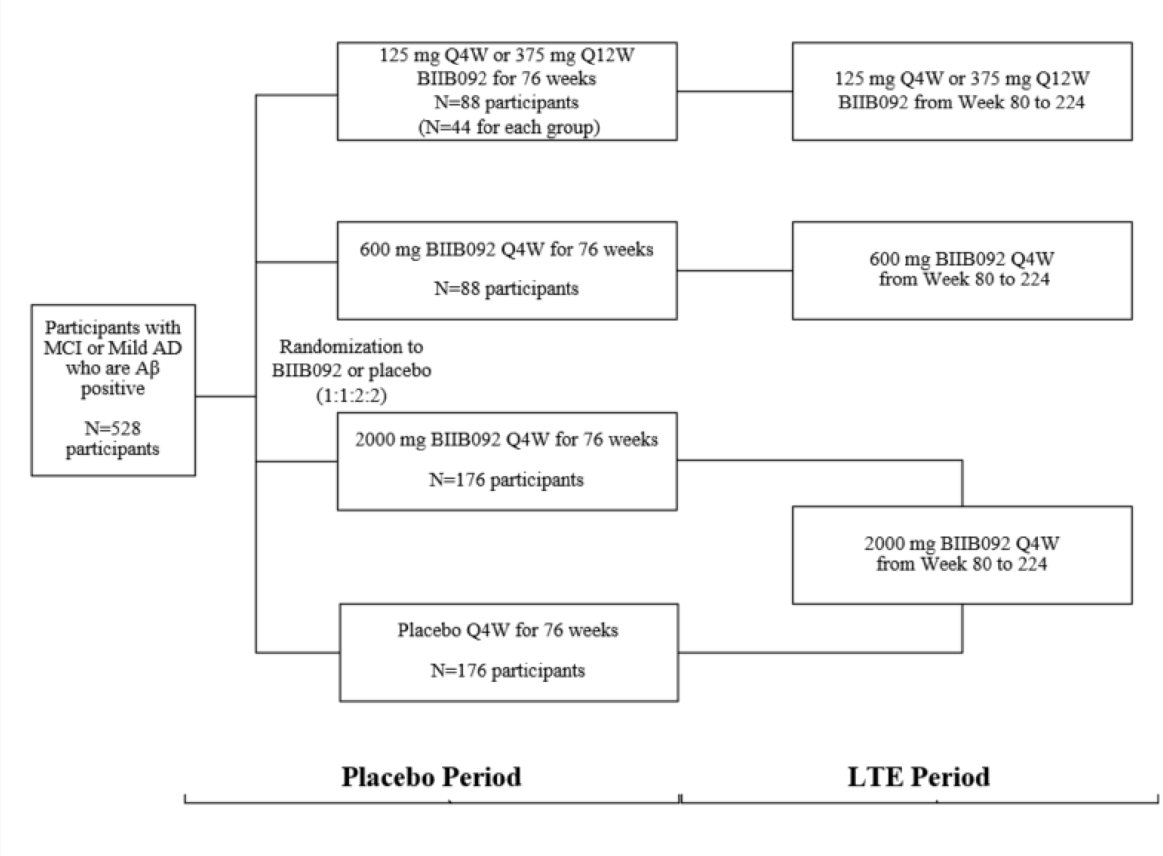

Aβ = amyloid beta; **AD = Alzheimer's disease; EOS = End of Study; LTE = long-term extension;** MCI = ~~M~~**m**ild ~~C~~**c**ognitive ~~I~~**i**mpairment; N = number of ~~subjects~~**participants**; Q4W = once every 4 weeks; Q12W = once every 12 weeks

Note: Overall, ~~participating subjects~~**participants** will have a 2:1 chance of being randomized to BIIB092 versus placebo **during the placebo-controlled period, and all participants will receive BIIB092 during the dose-blinded LTE period**.
Note: The study comprises a **double-blind, placebo-controlled period and an LTE period. The total duration for participants who complete the placebo-controlled period and do not enter the LTE period will be approximately 99 weeks. The total study duration for participants who complete both the placebo-controlled period and the LTE period will be approximately 247 weeks. The double-blind placebo-controlled period comprises a** Screening Period **of** ~~up to~~ **approximately** 9 weeks (65 days), a Treatment Period of 76 weeks, **and for participants not entering the LTE period,** an ~~End of Study~~ **EOS** Visit at Week 78, and a Follow-up Safety Visit at Week 90, or approximately 14 weeks after the last dose of study treatment. **The LTE period comprises an LTE Screening Period of approximately 4 weeks starting at Week 76, a 144-week Treatment Period starting at Week 80, an EOS Visit at Week 226, and a Follow-up Safety Visit at Week 238, or approximately 14 weeks after the last dose of study treatment.**

CONFIDENTIAL
The information contained herein may not be used, disclosed, or published without the written consent of Biogen MA Inc.

Rationale: To date, BIIB092 has been generally well tolerated in the clinical study participants. There are no important identified or important potential risks in the program, including in the completed studies CN002001 and CN002003 and in the ongoing studies 251PP201, 251PP301, and 251AD201. The current safety profile supports continued investigation of the proposed doses of 125 to 2000 mg. The LTE will provide additional long-term safety data.

Switching all participants who received placebo during the placebo-controlled period to 1 group (2000 mg every 4 weeks) in the LTE period allows delayed start analysis to be maximized for 1 of the dose groups.

This change also affects Section 5, Schedule of Activities (addition of 3 new tables for the LTE period); Section 6, Study Objectives and Endpoints; Section 7.2, Study Duration for Participants; [redacted] addition of Section 8.3, Inclusion Criteria for Long-Term Extension Period and Section 8.4, Exclusion Criteria for Long-Term Extension Period; Section 9.1 Screening; Section 9.2, Randomization; Section 9.3, Blinding Procedures; Section 10.1, Discontinuation of Study Treatment; Section 11.1, Regimen; Section 11.5, Continuation of Treatment; Section 13.6, Immunogenicity; and Section 16, Statistical Methods and Determination of Sample Size.

CONFIDENTIAL
The information contained herein may not be used, disclosed, or published without the written consent of Biogen MA Inc.

SUMMARY OF MAJOR CHANGES TO THE PROTOCOL

Changes to the protocol are presented chronologically. New text is shown in **bold** type; deleted text is shown with a ~~strikethrough~~.

Section 1, Synopsis

The Synopsis was revised to reflect changes made throughout the protocol.

Section 4.5, Overall Benefits and Risk Assessment

Change: Updated the benefits and risks assessment information.

Now reads:

~~Due to its eTau-lowering effects, BIIB092 has the therapeutic potential to prevent transmission of tau pathology in neurodegenerative disorders known as tauopathies, which include PSP and AD. The proposed indications under the current program of research are for use in patients with PSP and AD.~~

~~To date, 2 clinical studies have been completed with BIIB092. These include the first in human study, CN002001 (SAD study in healthy adult subjects) and CN002003 (MAD study in subjects with PSP). In addition to this study, 2 studies are ongoing: Study 251PP201 (formerly referred to as CN002004), an open-label extension study, and Study 251PP301 (formerly referred to as CN002012), a Phase 2b randomized controlled efficacy and safety study in subjects with PSP. Please refer to Section 4.3.2 for more information.~~

~~All available clinical benefit and risk information to date has been derived from studies in healthy volunteers or subjects with PSP. PSP is a rare neurodegenerative disease that results in a rapidly progressing, fatal movement disorder that includes cognitive and behavioral abnormalities. There are currently no approved or effective treatments for PSP. Nonclinical models support the anti-eTau mechanism as potentially efficacious in the treatment of PSP.~~

~~Binding to eTau, the murine antibody from which BIIB092 is derived, prevented tau transmission in nonclinical studies. Furthermore, the murine antibody from which BIIB092 was derived prevented tau-dependent behavioral and pathologic changes in a mouse model of tauopathy. Finally, BIIB092 reduced free eTau levels in the CSF of cynomolgus monkeys following a single IV administration. For additional details, please refer to Section 4.3.1. By binding eTau, it is expected that BIIB092 will prevent tau transmission in diseases such as AD and PSP and thereby potentially provide therapeutic benefit.~~

CONFIDENTIAL
The information contained herein may not be used, disclosed, or published without the written consent of Biogen MA Inc.

~~The nonclinical toxicity profile of BIIB092 was used to determine the starting dose and eligibility criteria and to develop appropriate safety monitoring for BIIB092 studies. Clinical data from the completed first-in-human SAD study in healthy adult subjects (CN002001) demonstrated that single doses of BIIB092 up to 4200 mg in Caucasian subjects and up to 2100 mg in Japanese subjects were generally safe and well tolerated. The completed (MAD) study in subjects with PSP (CN002003) evaluated multiple doses of BIIB092 (150, 700, and 2100 mg) or placebo administered once every 4 weeks for 3 months. The available data suggest that multiple doses of BIIB092 up to 2100 mg are safe and well tolerated in subjects with PSP. Correspondingly, the current ongoing open-label, long-term extension, safety and tolerability study (251PP201) also suggests that multiple doses of BIIB092 up to 2100 mg are safe and well tolerated in subjects with PSP.~~

~~Frequent safety assessments will be utilized by the Sponsor/Medical Monitor and Investigators to determine whether dose modification, additional safety measures, or termination of the ongoing studies is required at any time. Serum immunogenicity sampling will be performed to monitor for the emergence of anti-drug antibodies. Thorough evaluation of safety monitoring procedures and of AEs and serious adverse events (SAEs) will be reviewed on an ongoing basis by the Sponsor's Medical Monitor and Global Pharmacovigilance representatives to monitor for any safety signals or trends.~~

~~In addition, an independent Data Monitoring Committee (DMC) was established to monitor the benefit/risk profile of BIIB092.~~

~~For information on specific risk mitigation strategies, please refer to Section 10, Section 11.2.2, and Section 15.4.~~

~~A need exists for disease-modifying therapies for participants with AD and those with PSP. The doses of BIIB092 currently being explored have the potential to benefit study subjects. Furthermore, the nonclinical efficacy profile and the evidence for anti-eTau activity, together with the benign safety profile, indicate that the balance of benefit to risk is likely to be favorable for study subjects.~~

4.5.1. Overall Benefit

BIIB092 has the potential to slow or stop the spread of tau pathology observed in neurodegenerative diseases such as PSP, AD, and other tauopathies.

In participants with PSP, BIIB092 has been evaluated in 2 completed clinical studies (Studies CN002001 and CN002003) and is currently being evaluated in the ongoing Studies 251PP201 and 251PP301. The first-in-human study of BIIB092 (Study CN002001) was designed as a randomized, double-blind, placebo-controlled, SAD study in healthy participants to characterize the safety, tolerability, PK, PD, and immunogenicity of single doses of BIIB092, ranging from 21 to 4200 mg. Study CN002003, a Phase 1b study, was designed as a randomized, double blind, placebo-controlled MAD study to characterize the safety, tolerability, PK, PD, and immunogenicity of multiple doses of BIIB092, ranging from 150 to 2100 mg, in participants with PSP. In both of these completed studies, BIIB092

was found to be well tolerated at the doses tested in both healthy participants and participants with PSP. The ongoing Phase 1b study, Study 251PP201, is designed as an open-label extension study to evaluate the long term safety and tolerability of multiple doses of BIIB092 in participants with PSP who participated in Study CN002003. Several interim analyses of the data being collected in this study have been performed to date. Study 251PP301 is an ongoing Phase 2b study designed as a randomized, double blind, placebo-controlled, parallel-group study to evaluate the efficacy and safety of IV administered BIIB092 in participants with PSP.

In participants with AD, this study (Study 251AD201) will evaluate the safety and efficacy of BIIB092.

Based on the PK and PD data from Studies CN002001 and CN002003, the mean concentrations of BIIB092 in CSF increased in a generally dose-proportional manner. A robust and persistent lowering of unbound N-terminal tau was observed following administration of single and multiple doses of BIIB092 in healthy participants and in participants with PSP, consistent with the observations in cynomolgus monkeys.

4.5.2. Potential Risks

The nonclinical studies conducted to date have demonstrated an acceptable safety profile for BIIB092 to support continued use in clinical studies.

As of 25 November 2018, an estimated 486 healthy participants or participants with PSP or AD have been exposed to BIIB092 in clinical studies. The following summary of the safety profile of BIIB092 is based on safety data from the completed Studies CN002001 and CN002003 and the ongoing Study 251PP201. In the SAD study in healthy participants, there were no safety findings of note, and development continued in patients with PSP. In participants with PSP, to date, the most commonly reported AEs were fall, urinary tract infection (UTI), contusion, and headache. Most AEs have been reported as mild or moderate in intensity. Serious AEs (SAEs) have generally been consistent with disease (UTI, respiratory arrest, aspiration pneumonia, and progressive PSP) or are not unexpected in the patient population enrolled in the trials (cholecystitis, cancer, fractures, and anemia). There have been 3 deaths, all of which were likely related to underlying disease (respiratory arrest, aspiration pneumonia, and progressive disease). None of the SAEs or deaths were considered related to BIIB092 by Investigators. No safety concerns have been identified from laboratory, vital signs, or electrocardiogram (ECG) assessments. Nonserious infusion reactions have been observed and are an identified risk of BIIB092.

BIIB092 is a humanized IgG4 monoclonal antibody. While a low risk of immunogenicity is suggested by preclinical studies, the risk of immunogenicity in humans is unknown. Immunogenicity is an AE of special interest that is under monitoring in all ongoing studies. No safety data regarding this topic have emerged that would change the safety profile of BIIB092.

CONFIDENTIAL
The information contained herein may not be used, disclosed, or published without the written consent of Biogen MA Inc.

In general, BIIB092 has been well tolerated in the clinical study participants. There are no important identified or potential risks in the program. The safety profile is acceptable to continue development.

4.5.3. Summary

Currently available treatments for AD offer modest symptomatic relief, but none has the potential to modify the underlying disease pathology or course of the disease. In addition, no medications have been approved for the treatment of PSP. Therefore, there is a significant unmet need for the development of effective disease-modifying therapies in both PSP and AD.

Based on the PK and PD data from Studies CN002001 and CN002003, the mean concentrations of BIIB092 in CSF increased in a generally dose-proportional manner. A robust and persistent lowering of unbound N-terminal tau was observed following administration of single and multiple doses of BIIB092 in healthy participants and in participants with PSP, consistent with the observations in cynomolgus monkeys.

Based on the safety data from the completed Studies CN002001 and CN002003 and the data from an interim analysis of the ongoing Study 251PP201, BIIB092 was generally well tolerated and demonstrated an acceptable safety profile for continued development.

The overall analysis of potential benefits (based on the robust and persistent lowering of unbound N terminal tau in healthy participants and in participants with PSP, consistent with cynomolgus monkeys) and risks (available safety data indicating that BIIB092 is generally well tolerated) supports the continued development of BIIB092 in both PSP and AD.

Rationale: Updated the benefit and risk assessment information for BIIB092 to align with the Investigational Medicinal Product Dossier. Note that the overall risk and benefit assessment remains unchanged as a result of this update.

Section 7.1, Study Overview

Change: Require that participants be observed for a minimum of 1 hour after the end of infusion on dosing visits.

Now reads:

…

Throughout the study, all participants should be observed and monitored by study staff for a minimum of 1 hour after the end of an infusion. Participants who experience an AE or SAE related to BIIB092 infusion should remain at the site or be sent to an inpatient

monitoring facility until the Investigator has determined that the event(s) has resolved or do not require further monitoring at the site or in an inpatient setting.

Rationale: The requirement to monitor participants for 1 hour after dosing was added to further ensure the safety of the participants.

This change also affects Section 5, Schedule of Activities and Section 7.2, Study Duration of Participants.

Section 8.1, Inclusion Criteria

Change: [redacted]

Now reads:

[redacted]

Rationale: [redacted]

Section 8.2, Exclusion Criteria

Change: Exclusion criteria regarding the participants' hepatitis B and C status, medical history, medication use, and study procedure were updated and clarified. Additionally, criterion 2was split into criteria 2 and 3, and as a result, all subsequent exclusion criteria were renumbered.

Now reads:

Medical history

2. ~~History of, or positive test result at Screening for,~~ **Current** hepatitis C ~~virus antibody or~~ **infection (defined as positive hepatitis C virus [HCV] antibody and detectable HCV ribonucleic acid [RNA]). Participants with positive HCV antibody and undetectable HCV RNA are eligible to participate in the study (United States Centers for Disease Control and Prevention).**

3. Current hepatitis B ~~virus~~ **infection** (defined as positive for ~~both~~ hepatitis B surface antigen **[HBsAg]** and**/or total** hepatitis B core antibody **[anti-HBc]**) ~~unless fully recovered with no active infection indicated by a serology panel~~. **Participants with immunity to hepatitis B from previous natural infection (defined as negative HBsAg, positive anti-HBc, and positive hepatitis B surface antibody [anti-HBs]) or vaccination (defined as negative HBsAg, negative anti-HBc, and positive anti-HBs) are eligible to participate in the study.**

…..

~~10.~~**11.** Brain MRI performed at Screening Visit 2 (centrally read) that shows evidence of any of the following:

…..

- Cortical infarct (**including cerebellar infarct**) [defined as >1.5 cm in diameter] or any infarct in the hippocampus.

…..

~~19~~.**20.** History of premalignant or malignant disease. **Exceptions to premalignant disease exclusions may be made after discussion with the Sponsor.** The following exceptions may be made **for malignant disease exclusions** after discussion with the Sponsor:

- ~~Subjects~~**Participants** with cancers in remission ≥5 years prior to Screening Visit 1.
- ~~Subjects~~**Participants** with a history of excised or treated basal cell or squamous carcinoma of the skin.
- ~~Subjects~~**Participants** with localized prostate cancer with treatment cycles that completed at least 6 months prior to Screening Visit 1.

…..

CONFIDENTIAL
The information contained herein may not be used, disclosed, or published without the written consent of Biogen MA Inc.

Medications

~~29~~**30**. Use of the following medications:

…..

– ~~Antipsychotics~~ **High-dose antipsychotics** used on a regular basis~~, except for low.~~ **Low** doses of atypical **and typical** antipsychotics ~~(e.g., risperidone, aripiprazole, or quetiapine)~~ if used on an as-needed basis or if used at a stable dose for 8 weeks prior to Screening Visit 1 and during the screening period up to Study Day 1. The definition of "low dose~~s~~" **and "high dose"** should be judged by the Investigator, and the Medical Monitor can be consulted if needed.

…..

~~34.~~**35.** Participation within the 12 months prior to Screening Visit 1 in a study of any other agent(s) **not included in exclusion criteria 32, 33, and 34** with a purported disease-modifying effect in AD, unless documentation of receipt of placebo is available.

…..

Study Procedures

…..

~~40.~~**41.** [redacted]

Rationale: [redacted]

[redacted]

<u>Section 9.2, Randomization</u>

Change: A slight change in the ratio of study participants with mild cognitive impairment (MCI) due to AD versus mild AD was made.

CONFIDENTIAL
The information contained herein may not be used, disclosed, or published without the written consent of Biogen MA Inc.

Now reads:

Enrollment will be monitored, via the interactive response technology (IRT) system, so that the population of ~~subjects~~**participants** with mild AD represents ~~about~~**approximately** ~~60~~**50%** of the total number of ~~subjects~~**participants** enrolled in the study.

Rationale: A reassessment of the ratio of study participants with MCI due to AD versus mild AD was completed. The ratio was changed as a result and is not expected to affect the objectives of the study.

Section 14.2, Laboratory Safety Assessments

Change: [redacted]

Now reads:

- [redacted]

Rationale: [redacted]

[redacted]

[redacted]

Section 15.3.2, Adverse Events of Special Interest

Change: Immunogenicity was added as an adverse event (AE) of special interest.

Now reads:

~~No AEs of special interest have been identified for BIIB092 to date.~~ **Immunogenicity is an AE of special interest that is under monitoring in all ongoing studies. Participants will also be monitored for possible infusion-associated AEs and/or hypersensitivity reactions during and after completion of the investigational medicinal product infusion.**

Rationale: The addition of immunogenicity as an AE of special interest was done to align with updates made to the Investigator's Brochure, Section 6.3.2.5 Adverse Events of Special Interest.

This changes also affects Section 4.5.2, Potential Risks and Section 5, Schedule of Activities.

Section 17.3, Participant Information and Consent

Change: Updated language to state that participants can withdraw at any time from the study and how withdrawal from substudies impacts their participation in the overall study.

Now reads:

....

Participants or their legally authorized representatives, in countries where applicable laws allows, who chose to participate in the LTE period must provide informed consent before any LTE period screening tests are performed. [redacted]

....

The participant's study partner/informant must also provide written informed consent to participate in the study **and be reconsented to participate in the LTE period**. The original

forms will be managed and archived in the same manner as the participants' ICFs, as described above.

Participants or their informant/study partner can withdraw consent to participate in the study at any time. [redacted]

Rationale: Language was added to clarify that participants can withdraw at any time from the study and clarify how withdrawal from substudies during the placebo-controlled period and LTE period impacts their participation in the overall study.

This change also affects Section 7.1, Study Overview and [redacted]

CONFIDENTIAL
The information contained herein may not be used, disclosed, or published without the written consent of Biogen MA Inc.

SUMMARY OF MINOR CHANGES TO THE PROTOCOL

The following minor changes were made to the protocol, as appropriate:

- The version number and date were updated throughout the protocol.
- Sponsor Signature Page was updated.
- Typographical errors (i.e. Section 5, Schedule of Activities Table 1, CSSR-S assessment was moved from Week 56 to Week 52 Visit) and formatting were corrected.
- The term "subject" was replaced with "participant" throughout the document to reflect current standards when referring to participants in a study.
- Section 2, List of Abbreviations, was updated.
- Section 7.1, Study Overview, the number of study sites was increased from 90 to 100.
- [redacted]
- Section 8.1, Inclusion Criteria, inclusion criterion 9 was updated to clarify that informant/study partner is required to give informed consent. Informant/study partner consent was previously required per in protocol version 2, Section 9.1, but it is now clarified in criterion 9.
- Section 10.1, Discontinuation of Study Treatment, was updated to clarify that participants who experiences an AE or an SAE that does not resolve or requires continued treatment that meets exclusionary criteria must be permanently discontinued from study treatment.
- Section 13.2, Pharmacokinetic Assessments, was corrected to include both trough and end-of-infusion BIIB092 concentrations.
- Section 16.8.1, Analysis Population, the term "immunogenicity" was replaced with "anti-BIIB092 antibody" as appropriate throughout the document when referring to anti-drug antibody assessment to avoid confusion with the AE of special interest similarly referred to as immunogenicity.

CONFIDENTIAL
The information contained herein may not be used, disclosed, or published without the written consent of Biogen MA Inc.

Biogen MA Inc.
225 Binney Street
Cambridge, MA 02142
United States

Biogen Idec Research Limited
Innovation House
70 Norden Road
Maidenhead, Berkshire
SL6 4AY
United Kingdom

AMENDMENT SUMMARY

Biogen Protocol 251AD201

Randomized, Double-Blind, Placebo-Controlled, Parallel-Group Study to Assess the Safety, Tolerability, and Efficacy of BIIB092 in Subjects with Mild Cognitive Impairment due to Alzheimer's Disease or with Mild Alzheimer's Disease

Version 4

Date: 13 January 2020

EUDRA CT Number: 2017-002901-37

Version 4 of the protocol has been prepared for this amendment, which supersedes Version 3.

CONFIDENTIAL
The information contained herein may not be used, disclosed, or published without the written consent of Biogen MA Inc.

PRIMARY REASON FOR AMENDMENT

The primary reason for this amendment to Protocol 251AD201 is to update the number of randomized participants from 528 to 654.

New text is shown in **bold** type; deleted text is shown with a ~~strikethrough~~.

<u>Section 7.1, Study Overview</u>

Now reads:

The study will be conducted in a parallel-group design, with 3 BIIB092 dose groups (including 4 BIIB092 doses) and a placebo group. Approximately 528 participants ~~will~~**were planned to** be randomized across approximately 100 study sites globally. **As a result of fast recruitment leading to over-enrollment, the final number of randomized subjects is 654.** [redacted]

…

Randomized participants will receive 1 of the following study treatments by IV infusion every 4 weeks, starting on Study Day 1 during the placebo-controlled period:

- low-dose BIIB092 - 125 mg once every 4 weeks or 375 mg once every 12 weeks and placebo at the other 4-week dosing visits to maintain the treatment blind (88 participants **planned** in total, 44 per regimen)
- medium-dose BIIB092 - 600 mg once every 4 weeks (88 participants **planned**)
- high-dose BIIB092 - 2000 mg once every 4 weeks (176 participants **planned**)
- placebo (176 participants **planned**)

Rationale: The number of study participants was updated to reflect the final number of participants randomized after over-enrollment. The increase from 528 to 654 participants represents a significant change to the study plan. Because the statistical analysis is based on a sample size of 528, language emphasizing the planned sample size was added for clarification.

This change also affects Section 13.3, Pharmacodynamic Assessments.

CONFIDENTIAL
The information contained herein may not be used, disclosed, or published without the written consent of Biogen MA Inc.

SUMMARY OF MAJOR CHANGES TO THE PROTOCOL

Changes to the protocol are presented chronologically. New text is shown in **bold** type; deleted text is shown with a ~~strikethrough~~.

Section 1, Synopsis

The synopsis was revised to reflect changes made throughout the protocol.

Section 5, Schedule of Activities

Change: The following changes were made to the timing of scheduled assessments:

Safety Procedures

The frequency of MRIs was adjusted by removing MRI from Week 176 and Week 128 and adding it to Week 156. MRI will now be performed at Week 104 (6 months after start of LTE), Week 156 (18 months after start of LTE), and Week 226 (36 months after start of LTE). A row for body weight was added to Tables 2 through 4. Body weight measurements were added to all clinical visits that include a physical examination. A row for tobacco use status was added to Tables 1 through 4 below concomitant therapy and procedures reporting.

Biomarker

The frequency of PK/biomarkers/anti-BIIB092 antibodies collection during the LTE was adjusted for multiple study visits. PK/biomarkers/anti-BIIB092 antibodies will now be assessed at Week 116, Week 156, Week 196, and Week 224.

Clinical Assessments

Removal of NPI-10 and FAQ at Week 176 and ISLT, DKEFS, DSST, Trails A, and eCog at Week 180.

Rationale: To reduce the burden to participants, the original 4 MRIs planned during the LTE at Weeks 104, 128, 176, and 226 (at 6, 12, 24, and 36 months after starting the LTE, respectively) were reduced to 3 MRIs at Weeks 104, 156, and 226 (at 6, 18, and 36 months after starting the LTE, respectively). The Week 104 MRI represents a critical safety assessment for participants switching from placebo to treatment during the LTE. The Week 226 MRI is key for PD evaluation and for safety assessment at the end of the study. The Week 128 and Week 176 MRIs were condensed into a single Week 156 MRI in order to minimize participant burden. Based on

CONFIDENTIAL
The information contained herein may not be used, disclosed, or published without the written consent of Biogen MA Inc.

current evaluations, this frequency of MRI monitoring is not expected to significantly affect the participants' benefit-risk. In addition, ad hoc MRI visits are possible as needed.

The frequency of biomarker and clinical assessments was reduced to decrease the burden to participants, particularly towards the end of the study.

A row for body weight was added to Tables 2 through 4 to provide further clarification on the frequency of body weight measurements.

A row for tobacco use status was added to Tables 1 through 4 to provide clarification on frequency and timing of the assessment.

This change also affects Section 11.4.3, Tobacco Use.

Section 5, Schedule of Activities

Change: A footnote was added to Table 1 for the Week 78 MRI.

Now reads:

[23]Sites should schedule the Week 78 MRI within 10 days prior to the Week 78 visit where possible to allow for MRI results to be available before participants enter the LTE at Week 80.

Rationale: This change was made to improve the efficiency of eligibility assessments by allowing MRI results to be available before entering the LTE at Week 80.

Section 5, Schedule of Activities

Change: The footnote in Table 1 regarding blood samples for assessment of BIIB092 concentration on days of infusion was revised.

Now reads:

[20]Two samples must be obtained: 1 sample taken before infusion and 1 sample taken at ≤~~10~~**15** minutes after the end of infusion.

Rationale: This change was made to increase flexibility by providing a larger time window for sample collection with minimal impact on PK.

CONFIDENTIAL
The information contained herein may not be used, disclosed, or published without the written consent of Biogen MA Inc.

[redacted]

[redacted]	[redacted]

[redacted]

Section 7.1, Study Overview

Change: All references to optional home visits were removed.

Now reads:

~~The visits at Weeks 32, 36, and 64 of the placebo controlled period may be conducted as home visits, if the Investigator is in agreement and appropriate services are available to perform the required study procedures and adequately monitor for potential safety events. These are study visits that do not require extensive assessments, e.g., administration of clinical scales~~ [redacted]~~. However, if the participant experienced a clinically significant infusion reaction during the first 24 weeks of the study, then the BIIB092 infusion should not be administered in the home unless previously approved by the Investigator.~~

…

~~The visits at Weeks 84, 88, 96, 112, 116, 120, 136, 140, 144, 160, 164, 168, 184, 188, 192, 208, 212, and 216 of the LTE period may be conducted as home visits, if the Investigator is in agreement and appropriate services are available to perform the required study procedures and adequately monitor for potential safety events. These are study visits that do not require extensive assessments, e.g., administration of clinical scales~~ [redacted]~~. However, if the participant experienced a clinically significant infusion reaction during the first 24 weeks of the study or LTE period, then the BIIB092 infusion should not be administered in the home unless previously approved by the Investigator.~~

CONFIDENTIAL
The information contained herein may not be used, disclosed, or published without the written consent of Biogen MA Inc.

Rationale: These changes were made for logistical reasons and are not expected to affect patient safety.

This change also affects Section 5, Schedule of Activities and Section 7.2, Study Duration for Participants.

Section 10.1, Discontinuation of Study Treatment

Change: Changes were made to recommended visits for participants who discontinue study treatment to reflect changes in the Schedule of Activities.

Now reads:

All participants who discontinue study treatment prematurely will be asked to remain in the study and continue all protocol-specified visits and procedures for the period in which they discontinued treatment (i.e., placebo-controlled or LTE). Participants who discontinue treatment during the placebo-controlled period should be encouraged to attend at least the Week 24, Week 52, and Week 78/EOS Visits and who discontinue treatment during the LTE should be encouraged to attend at least the Week **80,** 104, **116,** 128, 152, **156,** 176, **196,** 200, **224,** and 226/EOS Visits, depending on when treatment was discontinued, and undergo all the scheduled procedures. At a minimum, these visits should include assessment of AEs/SAEs and concomitant medications and key clinical assessments, including at least the CDR, MMSE, ISLT, ADAS-Cog 13, ADCS-ADL, and FAQ. Participants who discontinue study treatment prematurely, regardless of the treatment period, will be asked to return to the study site for a Follow-up Safety Visit 14 weeks after receiving the last dose of study treatment. See Table 1, Table 2, Table 3, and Table 4 for the Schedule of Activities.

Rationale: Revision made to account for updates to timing of significant assessments in Table 2, Table 3, and Table 4.

Section 11.4.1.2, Disallowed Concomitant Therapy

Change: Details were added regarding UVs for a change in AD medication.

CONFIDENTIAL
The information contained herein may not be used, disclosed, or published without the written consent of Biogen MA Inc.

Now reads:

Participants should have an unscheduled visit **(UV)** prior to **the** initiation of, ~~and/or a~~ change in~~,~~ **dose, or discontinuation of** AD medication. That visit should include assessment of AEs/SAEs and concomitant medications and key clinical assessments, including, at a minimum, the CDR, MMSE, ISLT, ADAS-Cog 13, ADCS-ADL, and FAQ. **During the UV, these clinical assessments need not be repeated if performed within the previous 30 days. If the visit for a change in AD medication occurs at the same time as another planned visit, all originally scheduled assessments must also take place as required and without repetition (see Table 1, Table 2, Table 3, and Table 4).**

Rationale: Details were added to minimize participant burden and to optimize the assessments performed during a change in AD medication as well as provide clarification for study sites.

This change also affects Section 5, Schedule of Activities.

CONFIDENTIAL
The information contained herein may not be used, disclosed, or published without the written consent of Biogen MA Inc.

SUMMARY OF MINOR CHANGES TO THE PROTOCOL

The following minor changes were made to the protocol, as appropriate:

- The version number and date were updated throughout the protocol.
- Section 2, List of Abbreviations, was updated.
- Abbreviations were updated throughout the protocol where appropriate.
- The abbreviation list in the Figure 1 footnotes was updated to clarify that N represents the **planned** number of participants.
- All references to free eTau were changed to N-terminal tau or removed.
- Section 16.5.2, Methods of Analysis, was revised to clarify that concentration values below the limit of quantification will be appropriately handled per the Statistical Analysis Plan.
- Internal references were added as hyperlinks where appropriate.
- Typographical errors and formatting were corrected.

CONFIDENTIAL
The information contained herein may not be used, disclosed, or published without the written consent of Biogen MA Inc.

LIST OF ABBREVIATIONS

AD	Alzheimer's disease
ADAS-Cog 13	Alzheimer's Disease Assessment Scale – Cognitive (13 item)
ADCS-ADL	Alzheimer's Disease Cooperative Study – Activities of Daily Living
AE	adverse event
CDR	Clinical Dementia Rating Scale
[redacted]	[redacted]
DKEFS	Delis-Kaplan Executive Function System
DSST	Digit Symbol Substitution Test
eCog	Everyday Cognition
EOS	end of study
[redacted]	[redacted]
FAQ	Functional Activities Questionnaire
ISLT	International Shopping List Test Immediate Recall
IV	intravenous
[redacted]	[redacted]
LTE	long-term extension
MCI	mild cognitive impairment
MMSE	Mini-Mental State Examination
MRI	magnetic resonance imaging
NPI-10	Neuropsychiatric Inventory – 10
PD	pharmacodynamic(s)
[redacted]	[redacted]
PK	pharmacokinetic(s)
SAE	serious adverse event
Trails A	Trail Making Test, Part A
UV	unscheduled visit

CONFIDENTIAL
The information contained herein may not be used, disclosed, or published without the written consent of Biogen MA Inc.

DocuSign Envelope ID: 7A1F456A-919D-4916-B66C-75B177AF3423

Tango

251AD201/NCT03352557

Statistical Analysis Plan

Placebo-Controlled Period

CONFIDENTIAL
The information contained herein may not be used, disclosed, or published without the written consent of Biogen MA Inc.

STATISTICAL ANALYSIS PLAN
Placebo-Controlled Period

Product Studied: BIIB092
Protocol Number: 251AD201

Randomized, Double-Blind, Placebo-Controlled, Parallel-Group Study to Assess the Safety, Tolerability, and Efficacy of BIIB092 in Subjects with Mild Cognitive Impairment due to Alzheimer 's disease or with Mild Alzheimer's Disease

Protocol Version: Version 4
Date of Protocol: 13 January 2020

Date of Statistical Analysis Plan: 26 March 2021, Version 2.0

Authors: [redacted], PhD (Biogen); [redacted], MS ([redacted])

Approved By:

DocuSigned by: [redacted] — 31-Mar-2021
[redacted], PhD — Date

DocuSigned by: [redacted] — 31-Mar-2021
[redacted], PhD — Date

DocuSigned by: [redacted] — 31-Mar-2021
[redacted], MD — Date

DocuSigned by: [redacted] — 31-Mar-2021
[redacted] — Date

CONFIDENTIAL
The information contained herein may not be used, disclosed, or published without the written consent of Biogen MA Inc.

Table of Contents

LIST OF ABBREVIATIONS 6

1 INTRODUCTION 8

2 STUDY OVERVIEW 8

2.1 PRIMARY OBJECTIVE AND ENDPOINT 8
2.2 SECONDARY OBJECTIVES AND ENDPOINTS 8
2.3 EXPLORATORY OBJECTIVES AND ENDPOINTS 8
2.4 STUDY DESIGN 9
2.5 STUDY SCHEMATIC 12
2.6 SAMPLE SIZE JUSTIFICATION 13

3 DEFINITIONS 14

3.1 DATES AND POINTS OF REFERENCE 14
3.2 STUDY TREATMENT 15
3.3 KEY DERIVED VARIABLES 15
3.4 STRATIFICATION FACTORS AND SUBGROUP VARIABLES 19
3.5 ANALYSIS SETS 20

4 LIST OF PLANNED STUDY ANALYSES 22

4.1 INTERIM ANALYSIS 22
4.2 FINAL ANALYSIS 22

5 STATISTICAL ANALYSIS METHODS 22

5.1 GENERAL CONSIDERATIONS 22
5.2 STUDY SUBJECTS 22
5.2.1 Accounting of Subjects 22
5.2.2 Demographics and Baseline Characteristics 23
5.2.3 Concomitant Medications and Non-drug Therapies 24
5.2.4 Protocol Deviations 25
5.2.5 Study Drug Exposure and Study Drug Compliance 25
5.2.6 COVID-19 Related Analysis 25
5.2.6.1 Accounting of subjects who discontinued due to Covid-19 25
5.2.6.2 Concomitant Non-Drug Treatment for Covid-19 26
5.2.6.3 Covid-19 Effect on Drug Compliance 26
5.2.6.4 Covid-19 Protocol Deviation 26
5.2.6.5 Protocol Alternation Due to Covid-19 26
5.3 EFFICACY ANALYSIS 26
5.3.1 General Considerations 26
5.3.1.1 Covid-19 consideration 27
5.3.2 Considerations for base MMRM model for change from baseline analyses 28
5.3.3 Primary Efficacy Endpoint 28
5.3.3.1 Primary analysis 28
5.3.3.2 Sensitivity analysis 29
5.3.3.3 Per-protocol analysis 31
5.3.4 Exploratory Efficacy Endpoints 32
5.3.5 Subgroup Analysis 32
5.3.6 Additional Analysis 32
5.3.6.1 CDR Subscores, CDR Global Score, ADCS-bADL, ADCS-iADL and individual scores for CDR, ADCS-ADL and ADAS-Cog 13 32
5.3.6.2 Slope analysis 33
5.3.6.3 Summary of CDR-SB, ADAS-Cog 13 total score, and MMSE by tau PET reduction at Week 78 33
5.3.6.4 Responder analysis of CDR-SB 34
5.4 SAFETY ANALYSIS 34
5.4.1 General Considerations 34
5.4.1.1 Analysis Set 34
5.4.1.2 Safety treatment groups 34
5.4.1.3 Incidence, incidence proportion and incidence rate 35

5.4.2 Clinical Adverse Events ... *35*
5.4.2.1 Treatment-emergent AEs (TEAEs) ... 35
5.4.2.2 Summary and incidence analysis ... 36
5.4.2.3 Incidence rate analysis ... 37
5.4.2.4 Infusion reactions ... 38
5.4.2.5 Adverse event of special interest ... 38
5.4.3 Clinical Laboratory Data ... *38*
5.4.3.1 Quantitative analyses ... 39
5.4.3.2 Qualitative analyses ... 39
5.4.4 C-SSRS Data ... *41*
5.4.5 ECG Data ... *41*
5.4.6 Vital Sign Data ... *42*
5.4.7 Physical Examination ... *43*
5.4.8 Neurological Examination ... *43*
5.4.9 Safety MRI Data ... *43*
5.4.10 COVID-19 Related Safety analysis ... *44*
5.5 PHARMACOKINETICS ANALYSIS ... 44
5.5.1 Serum and CSF PK Concentration Data ... *45*
5.5.2 Serum PK Parameters ... *45*
5.6 BIOMARKER ... 46
5.6.1 Pharmacodynamics Analysis ... *46*
5.6.2 Structural MRI Analysis ... *47*
5.6.2.1 By visit summary and MMRM model ... 47
5.6.2.2 Pearson and Spearman correlation ... 47
5.6.3 Tau PET Analysis ... *47*
5.6.3.1 Tau PET substudy ... 47
5.6.3.2 Tau PET target and reference regions ... 48
5.6.3.3 Tau PET primary analysis ... 48
5.6.3.4 Correlation between Tau PET vs. Cognitive results and other assessments ... 49
5.6.3.5 Subgroup analysis ... 50
5.7 ANTI-DRUG ANTIBODY ANALYSIS ... 50
5.7.1 Analysis Methods for Anti-drug antibody Data ... *50*
5.8 HEALTH OUTCOMES ... 51
5.8.1 QoL-AD and ZBI ... *51*
5.8.2 RUD-Lite ... *51*

6 CHANGES FROM PROTOCOL-SPECIFIED ANALYSES ... 53

6.1 MMRM MODEL ... 54
6.2 MCP-MOD MODEL ... 54

7 APPENDIX I: VISIT WINDOW MAPPING ... 55

8 APPENDIX II: DESCRIPTION OF MCP-MOD METHOD ... 59

9 APPENDIX III: QUESTIONNAIRE ... 60

9.1 CLINICAL DEMENTIA RATING SCALE (CDR) ... 60
9.2 MINI-MENTAL STATE EXAMINATION (MMSE) ... 60
9.3 INTERNATIONAL SHOPPING LIST TEST IMMEDIATE RECALL (ISLT) ... 60
9.4 DELIS-KAPLAN EXECUTIVE FUNCTION SYSTEM (DKEFS) ... 61
9.5 DIGIT SYMBOL SUBSTITUTION TEST (DSST) ... 61
9.6 TRAIL MAKING TEST, PART A (TRAIL A) ... 62
9.7 EVERYDAY COGNITION (ECOG) ... 62
9.8 ALZHEIMER'S DISEASE COOPERATIVE STUDY – ACTIVITIES OF DAILY LIVING (ADCS-ADL) ... 63
9.9 FUNCTIONAL ACTIVITIES QUESTIONNAIRE (FAQ) ... 63
9.10 ALZHEIMER'S DISEASE ASSESSMENT SCALE –COGNITIVE (13 ITEM) (ADAS-COG 13) ... 63
9.11 NEUROPSYCHIATRIC INVENTORY – 10 (NPI-10) ... 63
9.12 ZARIT BURDEN INTERVIEW (ZBI) ... 64
9.13 QUALITY OF LIFE FOR ALZHEIMER'S DISEASE (QOL-AD) ... 64
9.14 RESOURCE UTILIZATION IN DEMENTIA-LITE VERSION (RUD-LITE) ... 64

CONFIDENTIAL
The information contained herein may not be used, disclosed, or published without the written consent of Biogen MA Inc.

10 APPENDIX IV: ADVERSE EVENT OF SPECIAL INTEREST CUSTOM SEARCH CRITERIA 66

11 APPENDIX V: INFUSION REACTIONS CUSTOM SEARCH ... 67

12 REFERENCES ... 68

List of Abbreviations

AD	Alzheimer's Disease
ADA	Anti-Drug Antibody
ADAS-Cog 13	Alzheimer's Disease Assessment Scale-Cognitive Subscale (13 Items)
ADCOMS	Ad Composite Score
ADCS-ADL	Alzheimer's Disease Cooperative Study - Activities Of Daily Living Inventory
ADCS-iADL	ADCS-ADL Instrumental Total Score
ADCS-bADL	ADCS-ADL Basic Total Score
ADNI	Alzheimer's Disease Neuroimaging Initiative
AE	Adverse Event
ALP	Alkaline Phosphatase
ALT	Alanine Aminotransferase
ANCOVA	Analysis of Covariance
ApoE	Apolipoprotein E
APTT	Activated Partial Thromboplastin Tim
AST	Aspartate Aminotransferase
BLQ	Below the Limit of Quantification
BMI	Body Mass Index
bpm	Beats Per Minute
BUN	Blood Urea Nitrogen
CDR	Clinical Dementia Rating
CDR-SB	Clinical Dementia Rating-Sum of Boxes
CI	Confidence Interval
CIR	Copy Increment from Reference
C_{max}	Observed Maximum Serum BIIB092 Concentration
C_{min}	Observed Minimum Serum BIIB092 Concentration
CSF	Cerebrospinal Fluid
C-SSRS	Columbia Suicide Severity Rating Scale
CV	Coefficient of Variation
DKEFS	Delis-Kaplan Executive Function System
DSST	Digit Symbol Substitution Test
ECG	Electrocardiogram
EMACC	Early Ad/ MCI Alzheimer's Cognitive Composite
Emax	Maximum Response
EOS	End of Study
EOT	End of Treatment
ET	Early Termination
FAQ	Functional Activities Questionnaire
FAS	Full Analysis Set
eCOG	Everyday Cognition
eCRF	Electronic Case Report Form
ICE	Intercurrent Event
ICH	International Conference on Harmonisation
INR	Prothrombin Intl. Normalized Ratio
IRT	Interactive Response Technology
LS	Lease Square Mean

iADRS	The Integrated Alzheimer's Disease Rating Scale
ISLR	International Shopping List Test Delayed Recall
ISLT	International Shopping List Test Immediate Recall
LLOQ	Lower Limit of Quantification
LTE	Long-Term Extension
LP	Lumbar Puncture
MA	Macrohemorrhage
MCI	Mild Cognitive Impairment
MCMC	Markov Chain Monte Carlo
MCP-MOD	Multiple Comparison Procedure - Modelling
MedDRA	Medical Dictionary for Regulatory Activities
mH	Microhemorrhages
MMRM	Mixed-Model Repeated Measures
MMSE	Mini-Mental State Examination
MRI	Magnetic Resonance Imaging
NIA-AA	National Institute on Aging-Alzheimer's Association
NPI-10	Neuropsychiatric Inventory-10
PCS	Potentially Clinically Significant
PD	Pharmacodynamics(S)
PET	Positron Emission Tomography
pH	Potential of Hydrogen
PLS	Partial Least Squares
PMM	Pattern Mixture Model
PK	Pharmacokinetic(S)
PPS	Per-Protocol Analysis Set
PT	Preferred Term
PT	Prothrombin Time
Qol-AD	Quality of Life for Alzheimer's Disease
QTcF	Corrected QT Interval by Fredericia
RC_{min}	Accumulation Ratio Using C_{min}
RC_{max}	Accumulation Ratio Using C_{max}
ROI	Region of Interest
RUD-Lite	Resource Utilization In Dementia – Lite Version
SAE	Serious Adverse Event
SAP	Statistical Analysis Plan
SD	Standard Deviation
SOC	System Organ Class
SS	Superficial Siderosis
SUVR	Standard Uptake Value Ratio
TEAE	Treatment-Emergent Adverse Event
TIV	Total Intracranial Volume
Trails A	Trail Making Test, Part A
ULN	Upper Limit of Normal
VE	Vasogenic Edema
WHO	World Health Organization
ZBI	Zarit Burden Interview

CONFIDENTIAL
The information contained herein may not be used, disclosed, or published without the written consent of Biogen MA Inc.

1 Introduction

This statistical analysis plan (SAP) only covers the analyses for the primary, secondary and exploratory objectives for the placebo-controlled period of 251AD201. Hereafter, the placebo-controlled period of the study will be referred to as "the study" in the rest of this SAP (e.g., completion of the study means completion of the placebo-controlled portion). A separate SAP will be prepared for the analyses of the LTE period and integrated analyses across both portions of the study.

Unless stated otherwise, all statistical tests will be 2-sided with statistical significance level of 0.05.

The statistical software, SAS® will be used for all summaries and analyses.

2 Study Overview

2.1 Primary Objective and Endpoint

The primary objective of the study for the placebo-controlled period is to evaluate the safety and tolerability of BIIB092 in participants with MCI due to AD or with mild AD.

The primary safety endpoints that relate to this objective are the incidence of adverse events (AEs) and serious AEs (SAEs) during the placebo-controlled period.

2.2 Secondary Objectives and Endpoints

- To evaluate the efficacy of multiple doses of BIIB092 in slowing cognitive and functional impairment in participants with MCI due to AD or with mild AD as measured by the change from Baseline over time at Week 78 on the Clinical Dementia Rating Scale (CDR) -Sum of Boxes (CDR-SB). This is the primary efficacy objective, with the primary efficacy endpoint.
- To evaluate the immunogenicity of BIIB092 after multiple doses in participants with MCI due to AD or with mild AD as measured by the incidence of anti-BIIB092 antibodies in serum over time up to Week 90.

2.3 Exploratory objectives and endpoints

- To assess the effect of BIIB092 on the clinical progression of AD as measured by changes from Baseline over time up to Week78 on the Mini-Mental State Examination (MMSE), International Shopping List Test Immediate Recall (ISLT), Category Fluency and Letter Fluency tests from the Delis-Kaplan Executive Function System (DKEFS), Digit Symbol Substitution Test (DSST), Trail Making Test Part A(Trails A), Everyday Cognition(eCog), Alzheimer's Disease Cooperative Study-Activities of Daily Living (ADCS-ADL), Functional Activities Questionnaire (FAQ), Alzheimer's Disease Assessment Scale-Cognitive (ADAS-Cog13 [13item]), and Neuropsychiatric Inventory-10 (NPI-10).

- To assess the effect of BIIB092 on changes in caregiver burden and participant quality of life as measured by changes from Baseline over time up to Week 68 on the Zarit Burden Interview (ZBI) and Quality of Life for Alzheimer's Disease (QoL-AD).
- To assess the effect of BIIB092 on resource utilization as measured by the Resource Utilization in Dementia-Lite (RUD-Lite) Version results over time up to Week 68.
- To assess BIIB092 pharmacokinetics (PK) in serum (trough serum BIIB092 concentrations and end-of-infusion serum BIIB092 concentrations) from the samples collected at the visits indicated in the Schedule of Activities in participants with MCI due to AD or with mild AD.
- To assess BIIB092 PK in CSF from the samples collected at the visits indicated in the Schedule of Activities in participants with MCI due to AD or with mild AD who consent to participate in the CSF sampling substudy.
- To assess the effect of BIIB092 on tau protein concentrations in CSF (in subjects consenting to participate in the CSF sampling substudy) as measured by changes from Baseline over time up to Week 76 in CSF N-terminal eTau concentration.
- To assess the effect of BIIB092 on biomarkers in blood as measured by changes from Baseline over time up to Week76 on blood levels of disease-related biomarkers, including but not limited to, tau and other markers of neurodegenerative disease.
- To assess the effect of BIIB092 on disease-related biomarkers in CSF (only for participants who consent to participate in the CSF sampling substudy) as measured by changes from Baseline over time up to Week76 in disease-related CSF biomarkers, including but not limited to, phosphor-tau, neurogranin, and neurofilament (neurofilament light and phospho-neurofilament heavy).
- To assess the effect of BIIB092 on cerebral tau changes (in participants consenting to participate in the tau positron emission tomography [PET] substudy) as measured by changes from Baseline over time up to Week 78 on ^{18}F-MK6240 PET binding signal in certain brain regions.
- To assess the effect of BIIB092 on brain structure as measured by changes from Baseline over time up to Week 78 on magnetic resonance imaging (MRI) morphometric measures, including volume of certain brain areas.

2.4 Study Design

Study 251AD201 (TANGO) is a Phase 2, randomized, double-blind, placebo-controlled study of BIIB092 in subjects aged 50 to 80 years inclusive, with MCI due to AD or mild AD according to National Institute on Aging-Alzheimer's Association criteria (NIA-AA). Subjects must have Aβ positivity confirmed at Screening by either CSF sampling or an amyloid PET scan. Subjects must also perform at 1 standard deviation (SD) below the age-adjusted normative mean on either the ISLT or the International Shopping List Test Delayed Recall (ISLR) and have a Clinical CDR global score of 0.5 for MCI due to AD or 0.5 or 1 for mild AD, an MMSE score of 22 to 30 (inclusive), and a CDR Memory Box score of ≥0.5.

The study is conducted in a parallel-group design, with 3 BIIB092 dose groups (including 4 BIIB092 doses) and a placebo group. Approximately 528 subjects was planned to be randomized across approximately 90 study sites globally. Subjects were stratified by tau PET/CSF sampling substudy enrollment (see substudy description below), region, baseline disease stage (MCI or mild AD), and baseline AD symptomatic medication use. Subjects who enroll in both substudies will be considered as enrolled in the tau PET substudy for randomization purposes.

The study consists of a double-blind, placebo-controlled period and a dose-blinded LTE period. The total duration for participants who complete the placebo-controlled period and do not enter the LTE period will be approximately 99 weeks. For participants who complete both the placebo-controlled period and the LTE period, the total study duration will be approximately 247 weeks. The placebo-controlled period comprises a Screening Period of approximately 9 weeks (65 days), a Treatment Period of 76 weeks, and for participants not entering the LTE period, an End-of-Study (EOS) Visit at Week 78 and a Follow-up Safety Visit at Week 90, or approximately 14 weeks after the last dose of study treatment. However, the Screening Period for the placebo-controlled period may be extended up to 90 days in consultation with the Medical Monitor in advance and provided that written documentation is received from the Sponsor or delegate (e.g., for logistical issues such as PET radioligands). At the end of the placebo-controlled period, participants who meet the LTE entry criteria may enter the dose-blinded LTE period. The LTE period comprises an LTE Screening Period of approximately 4 weeks starting at Week 76, a 144-week Treatment Period, an EOS Visit at Week 226, and a Follow-up Safety Visit at Week 238, or approximately 14 weeks after the last dose of study treatment.

Randomized participants will receive 1 of the following study treatments by IV infusion, starting on Study Day 1 during the placebo-controlled period:

- low-dose BIIB092 -125mg once every 4 weeks or 375mg once every 12 weeks and placebo at the other 4-week dosing visits to maintain the treatment blind (88 subjects in total, 44 per regimen)
- medium-dose BIIB092 -600mg once every 4 weeks (88 subjects)
- high-dose BIIB092 -2000mg once every 4 weeks (176 subjects)
- placebo (176 subjects)

Overall, participants will have a 2:1 chance of being randomized to BIIB092 or to placebo during the placebo-controlled period.

During the LTE period, participants who were randomized to receive BIIB092 during the placebo-controlled period will continue to receive BIIB092 at the dose they were randomly assigned. Participants receiving placebo during the placebo-controlled period will receive BIIB092 at the high dose (2000 mg) once every 4 weeks during the LTE period. The BIIB092 dose concentration (i.e. low, medium, or high) to which participants are assigned to receive during LTE period may be changed based on emerging data from the BIIB092 clinical development program.

Throughout the study, all participants should be observed and monitored by study staff for a minimum of 1 hour after the end of an infusion. Participants who experience an AE or SAE related to BIIB092 infusion should remain at the site or be sent to an inpatient monitoring

facility until the Investigator has determined that the event(s) has resolved or do not require further monitoring at the site or in an inpatient setting.

Investigators, study staff (except for a designated Pharmacist/Technician), and study subjects and their families, caregivers, legal representatives will be blinded to the subjects' randomized treatment assignments.

This study includes 2 substudies to address exploratory study objectives: a tau PET substudy and a CSF sampling substudy. The tau PET substudy is mandatory at study sites that have access to the ^{18}F-MK6240 PET radioligand and have the capability to perform ^{18}F-MK6240 PET scans. During the placebo-controlled period, the CSF sampling substudy is mandatory at all study sites that do not have access to the ^{18}F-MK6240 PET radioligand. Participants will provide consent during Screening to participate in at least 1 of these substudies during the placebo-controlled period. Participants enrolled in the LTE period and who are participating in the tau PET substudy are required to continue participation in the tau PET substudy. Participation in the CSF sampling substudy during the LTE will be optional for all study participants, although participation will be encouraged. Participants may withdraw consent to participate in the substudies at any time.

CONFIDENTIAL
The information contained herein may not be used, disclosed, or published without the written consent of Biogen MA Inc.

2.5 Study Schematic

Figure 1. Study Design with Planned Sample Size

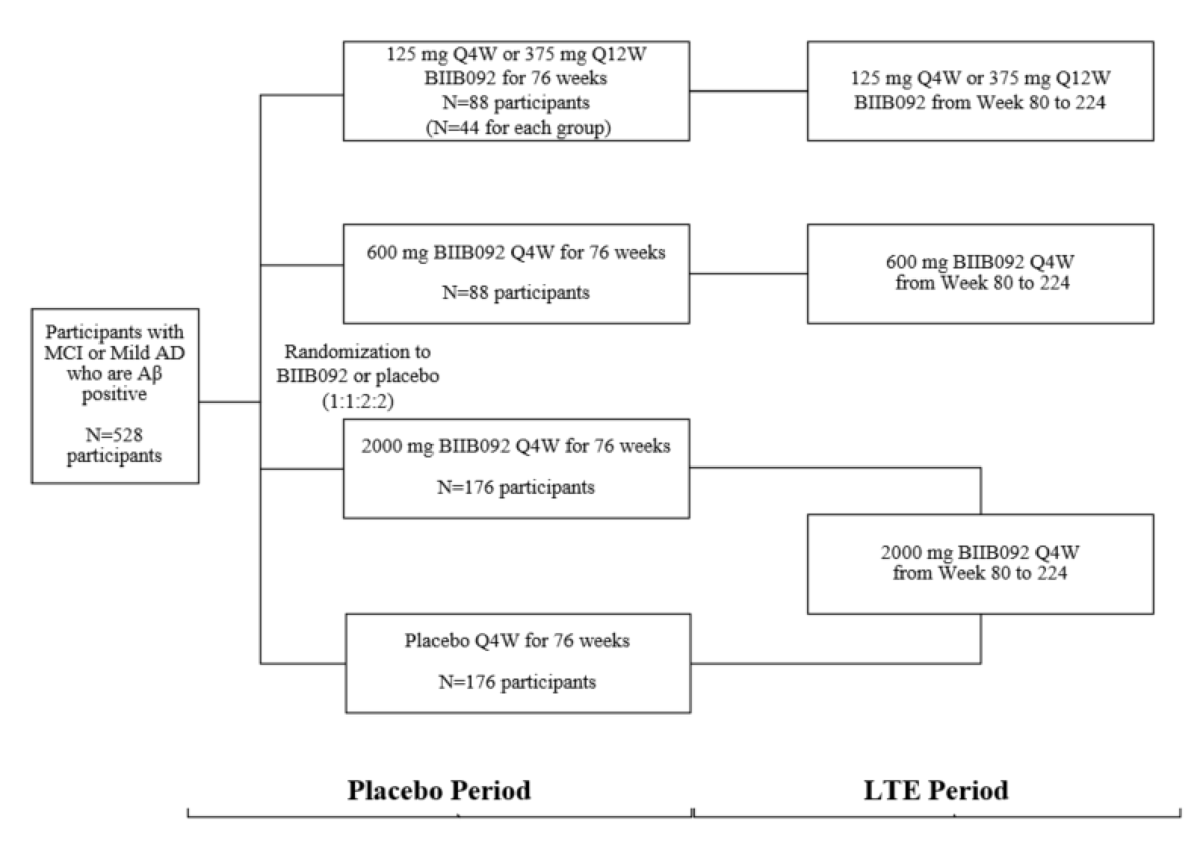

Aβ = amyloid beta; AD = Alzheimer's disease; EOS = End of Study; LTE = long-term extension; MCI = mild cognitive impairment; N = planned number of participants; Q4W = once every 4 weeks; Q12W = once every 12 weeks

Note: Overall, participants will have a 2:1 chance of being randomized to BIIB092 versus placebo during the placebo-controlled period, and all participants will receive BIIB092 during the dose-blinded LTE period.
Note: The study comprises a double-blind, placebo-controlled period and an LTE period. The total duration for participants who complete the placebo-controlled period and do not enter the LTE period will be approximately 99 weeks. The total study duration for participants who complete both the placebo-controlled period and the LTE period will be approximately 247 weeks. The double-blind placebo-controlled period comprises a Screening Period of approximately 9 weeks (65 days), a Treatment Period of 76 weeks, and for participants not entering the LTE period, an EOS Visit at Week 78 and a Follow-up Safety Visit at Week 90, or approximately 14 weeks after the last dose of study treatment. The LTE period comprises an LTE Screening Period of approximately 4 weeks starting at Week 76, a 144-week Treatment Period starting at Week 80, an EOS Visit at Week 226, and a Follow-up Safety Visit at Week 238, or approximately 14 weeks after the last dose of study treatment.

2.6 Sample Size Justification

There was no formal sample size calculation for the primary endpoint of safety.

The planned sample size is 528 subjects, randomized in a 1:1:2:2 ratio, with 88 subjects assigned to the BIIB092 low-dose group (44 assigned to 125mg once every 4weeks and 44 assigned to 375mg once every 12 weeks), 88 subjects assigned to the medium-dose group (600mg once every 4weeks), 176 subjects assigned to the high-dose group (2000mg once every 4 weeks), and 176 subjects assigned to the placebo group. This sample size provides approximately 80% power to detect a dose-response relationship in the change from baseline in CDR-SB (primary efficacy endpoint) at 18 months (Week 78), assuming a mean change of 1.99 from baseline in CDR-SB at 18 months in the placebo group and a common SD of 2.38, a maximal 40% reduction for the highest BIIB092 dose group compared with the placebo group, and an estimated 20% dropout rate at 18 months (Week 78) in this study. Six different dose-response relationships will be tested at the 2-sided 5% significance level, using the MCP-MOD method to control for multiplicity. Optimal contrasts will be constructed to detect potential dose-response trends under common dose-response curves (e.g., Emax, exponential, logistic, linear in log dose, and quadratic model) which are illustrated with the parameters shown in Figure2.

The mean and SD of the change from baseline in CDR-SB at 18 months for the placebo group is based on available Alzheimer's Disease Neuroimaging Initiative (ADNI) data from ADNI1, ADNI2, and ADNI GO (amyloid positive from amyloid PET or CSF, MMSE ≥22, CDR global score of 0.5 for late MCI and 0.5 or 1 for mild AD).

Based on mean changes in tau PET from Baseline to 9 months and 18 months in the Longitudinal Flortaucipir A05 study, it is assumed, by linear extrapolation, that the mean (SD) change from Baseline to 12 months (Week 52) for the placebo group in this study is assumed to be 0.034(0.033). Following the same MCP-MOD method under the same dose-response curves and parameters, the planned sample size for the tau PET substudy is 330 subjects, in a 1:1:2:2 randomization ratio, with 55 subjects assigned to the BIIB092 low-dose group (27 assigned to 125mg once every 4weeks and 28 assigned to the 375 mg once every 12weeks), 55 subjects assigned to the medium-dose group (600 mg once every 4weeks), 110 subjects assigned to the high-dose group (2000 mg once every 4weeks), and 110 subjects assigned to the placebo group to provide approximately 80% power to detect a maximal 40% reduction (mean change of 0.0136) and a common SD (0.033) for the highest BIIB092 dose group compared with the placebo group, with an estimated 15% dropout rate at 12 months (Week 52). Similarly, 6 different dose-response relationships will be tested at the 2-sided 5% significance level using the MCP-MOD method to control for multiplicity.

There was no formal sample size calculation for the CSF sampling substudy.

Sample size re-estimation was not done. However, blinded power was re-evaluated based on final randomized number of subjects after enrollment completed. Detailed information was documented in Note to File (251ad201 NTF_power re-estimation in CMF).

DocuSign Envelope ID: 7A1F456A-919D-4916-B66C-75B177AF3423

Figure 2 Dose-Response Shapes based on Clinical Dementia Rating Scale – Sum of Boxes Mean Change from Baseline at Week 78

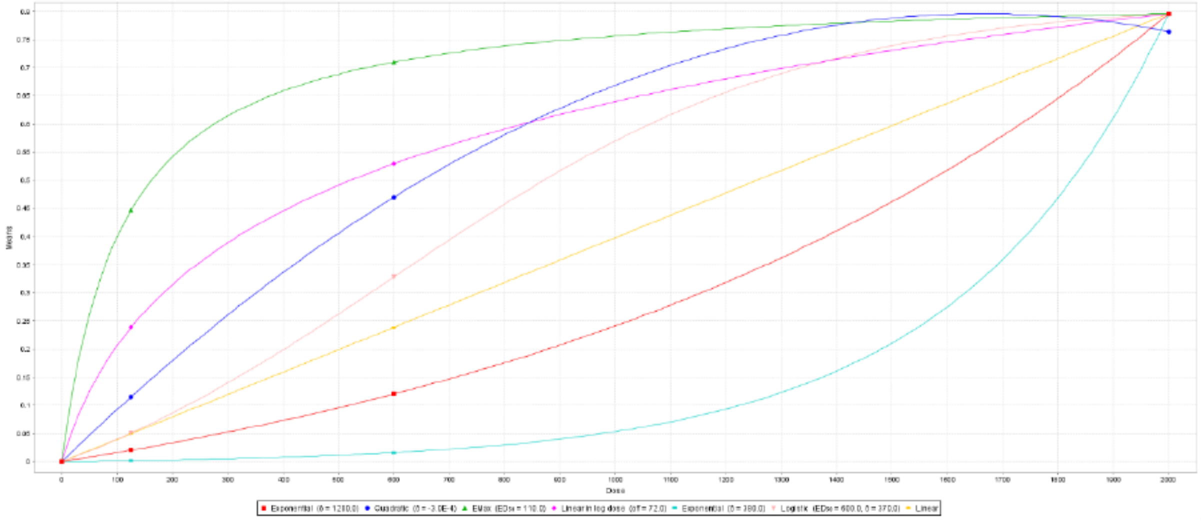

ED_{50} = median effective dose; Emax = maximum response
Note: This figure was generated using ADDPLAN® DF Version 3.1.8, by Aptiv solutions.

3 Definitions

3.1 Dates and Points of Reference

- Study Day 1: the date of the first dose of study treatment
- Study Day
  - For a date on or after Study Day 1

    Study Day = (Date of Interest) – (Study Day 1) + 1
  - For a date before Study Day 1

    Study Day = (Date of Interest) – (Study Day 1)

- Baseline value is defined as the most recent non-missing measurement collected prior to the first dose, unless otherwise specified

- Change from baseline will be defined as post-baseline value minus baseline value

- Percent change from baseline will be defined as post-baseline value minus baseline value then divided by baseline value

- Visit windows for analysis:

  For data that are summarized by visit, assessment from all scheduled visits, EOS visit and unscheduled visits will be mapped to an appropriate analysis visit using a windowing scheme as describe in Appendix I

3.2 Study Treatment

For efficacy, health outcome, statistical modeling of fluid PD and imaging biomarker analyses, the following treatment groups of BIIB092 (per randomization) will be evaluated and compared with placebo:

- Placebo
- low-dose BIIB092 -125mg once every 4weeks or 375mg once every 12weeks
- medium-dose BIIB092 - 600mg once every 4 weeks
- high-dose BIIB092 - 2000mg once every 4 weeks

The following treatment groups of BIIB092 will be evaluated for other analyses, such as study subject accounting, safety, fluid PD biomarkers and PK analyses, low dose group will be split out for different frequencies. If determined necessary, statistical modeling of fluid PD biomarkers may be conducted using the following treatment groups, as well.

- Placebo
- low-dose BIIB092 -125mg once every 4weeks
- low-dose BIIB092 - 375mg once every 12weeks
- medium-dose BIIB092 - 600mg once every 4 weeks
- high-dose BIIB092 - 2000mg once every 4 weeks

3.3 Key Derived Variables

- Handling of missing items for scales
  If any of the individual items for the primary efficacy endpoint and exploratory efficacy endpoints is missing, the total score of the corresponding endpoint will be imputed by prorating the observed scores [van Ginkel 2010].

  - For ADAS-Cog13, if 3 or fewer of 13 items (<25%) are missing, the total score will be imputed by the following algorithm: Total score = total score from the completed items * [maximum total score (=85) / maximum total score for the completed items]. The imputed number will be rounded up to the nearest integer. If more than 3 items are missing, the total score of ADAS-Cog 13 at that visit will be considered missing.
  - For ADCS-ADL, if 8 or fewer of 32 items (<25%) are missing, the total score will be imputed by a similar algorithm as that for ADAS-Cog 13. The imputed number will be rounded down to the nearest integer. If more than 8 items are missing, the total score for ADCS-ADL at that visit will be considered missing. Following the similar idea, for ADCS-iADL, if 6 or fewer out of 26 items are missing, the score will be imputed. For ADCS-bADL, if only 1 out of 6 items is missing, the total score will be imputed.
  - The same imputation algorithm will be applied to CDR-SB and MMSE, if only 1 box (of 6) of CDR is missing or if only 2 or fewer items (out of 11) are missing

for MMSE. The imputed CDR-SB will be rounded up to the nearest half integer, and the imputed MMSE will be rounded down to the nearest integer. If the score from more than 1 box of CDR or more than 2 items of MMSE is not available, the CDR-SB or MMSE at that visit will be considered missing.

- The total score of the exploratory endpoint NPI-10 and FAQ will be imputed using the same prorating principle and round up to the nearest integer if only 1 item (out of 10) is missing.

- iADRS derivation

The iADRS is a composite score based on ADAS-Cog and ADCS-iADL (instrumental ADCS-ADL) [Wessels et al. 2015, Wessels et al. 2018]. The iADRS is calculated as a linear combination of total scores of the two individual components, the ADAS-Cog13 (score range 0 to 85) and the ADCS-iADL (score range 0 to 56). Because higher score on the ADAS-Cog13 reflect worse performance, whereas higher scores on the iADCS-ADL reflect better performance, the ADAS-Cog score is multiplied by (-1) in the calculation of the integrated scale. To anchor the ADAS-Cog at 0, a constant (85) is added. The iADRS score is then computed as the sum of the transformed ADAS-Cog13 and the ADCS-ADL, as shown in the formula below:

iADRS score = [(-1) (ADAS-Cog13) + 85] + ADCS-iADL

The iADRS score ranges from 0 to 141 with lower scores indicating worse performance. If either ADAS-Cog13 or ADCS-iADL is missing, the iADRS score will be considered missing.

- EMACC derivation

The EMACC is a new and sensitive composite of well-known and validated neuropsychological tests that is suitable for examining the effect of disease modifying compounds on cognitive decline in the early Alzheimer Disease or MCI stage of Alzheimer's disease [Jaeger et al. 2018].

Cognitive variables (ISLT, DKEFS Category Fluency total correct score, DKEFS Letter Fluency total correct score, DSST total score, Trails A total time to complete) will be z-score transformed using the baseline score's mean and SD. For Trails A total time to complete, negative one (-1) will be multiplied when calculate the z-score. The EMACC score will be computed by taking the average of the z-scores across the five tests. Since the direction of Trails A total time to complete will be reversed, the higher EMACC score means cognitive improvement. If Trails A is missing, EMACC will be computed by taking the average of the z-scores across the remaining four tests. For the rest 4 component scores, if any of them is missing then the composite score will be missing.

- ADCOMS derivation

ADCOMS is a novel instrument developed to improve the sensitivity of currently available cognitive and functional measures for subjects in the prodromal stage of AD and mild AD dementia. It consists of 4 Alzheimer's Disease Assessment Scale–

cognitive subscale items, 2 Mini-Mental State Examination items, and all 6 Clinical Dementia Rating—Sum of Boxes items (Table 1). The composite score is a weighted linear combination of the individual scale's items using the corresponding PLS coefficients as weighting factors as listed in Table 1 [Wang et al. 2016]. The lower ADCOMS score shows clinical improvement. If any of the individual item is missing, then the composite score will be missing. The range of ADCOMS is between 0 and 1.97.

The formula for ADCOMS composite score is as below:

ADCOMS = ADCDRL * 0.008 + ADCOR * 0.017 + ADCRG * 0.004 + ADCDIF * 0.016 + (5 – MMS101) * 0.042 + (1 – MMS111) *0.038 + CDR0106 * 0.054 + CDR0104 * 0.109 + CDR0105 * 0.089 + CDR0103 * 0.069 + CDR0101 * 0.059 + CDR0102 * 0.078

Table 1. Items included in ADCOMS and their corresponding PLS coefficients

Scale	Item ID	Item name	PLS coefficients
ADAS-cog	ADCDRL	Delayed word recall	0.008
	ADCOR	Orientation	0.017
	ADCRG	Word recognition	0.004
	ADCDIF	Word finding difficulty	0.016
MMSE	MMS101	Orientation time	0.042
	MMS111	Drawing	0.038
CDR-SB	CDR0106	Personal care	0.054
	CDR0104	Community affairs	0.109
	CDR0105	Home and hobbies	0.089
	CDR0103	Judgement and problem solving	0.069
	CDR0101	Memory	0.059
	CDR0102	Orientation	0.078

ADAS-cog, Alzheimer's Disease Assessment Scale–cognitive subscale; CDR-SB, Clinical Dementia Rating, sum of boxes; MMSE, Mini-Mental State Exam; PLS, partial least squares.

- Adjusted structural MRI volume (% of TIV)

The adjusted structural MRI volume is defined as the percentage of ROI volume to the total intracranial volume (TIV). For example, the adjusted lateral ventricles volume at week 78 = (lateral ventricles volume at week 78/TIV) * 100%. The TIV (total intracranial volume) is measured at baseline and kept the same for all post-baseline visit.

3.4 Stratification Factors and Subgroup Variables

Stratification factors are:

- Tau PET/CSF substudy enrollment (If subjects enrolled in both substudies, subjects will be counted as tau PET substudy)
- Region [US, Australia, Japan, EU (EU countries include France, Germany, Italy, Spain, and Sweden), and Poland]
- Baseline disease stage (MCI or mild AD)
- Baseline AD symptomatic medication use (Yes or No)

Subgroup variables for the subgroup analysis in efficacy (Section 5.3.5)

- Baseline clinical stage (MCI due to AD or mild AD) per the Investigator's assessment based on the National Institute on Aging-Alzheimer's Association (NIA-AA) criteria
- Tau level at baseline measuring by tau PET (e.g. SUVR in primary target regions with primary reference region), CSF p-tau, or blood p-tau
- TIV adjusted hippocampal volume at baseline (quartiles of total hippocampal volume expressed as a % of TIV)
- Laboratory ApoE ε4 status (carrier/non-carrier)
- Use of AD symptomatic medication at baseline (yes or no)
- Age category (e.g. < 65, 65-<70, 70-<75, >=75)
- Gender (female or male)
- MMSE at baseline (e.g. $\geq$ median MMSE score at baseline vs. < median MMSE score at baseline)
- CDR global score at baseline (0.5 vs 1)
- Health Care Regions (US vs. other countries):
  - US
  - Non-US (Australia, Japan, France, Germany, Italy, Spain, Sweden, and Poland)

  * may analyze a country or countries separately if their data is not consistent comparing to other countries within the same category

Additional subgroup variables for subgroup analysis in efficacy:

- Magnitude of change on tau PET measures for the primary targets and reference regions at Week 78:
  - Change from baseline less than (mean - one standard deviation) of the placebo group at Week 78 vs.
  - Change from baseline greater than or equal (mean - one standard deviation) of the placebo group at Week 78

Subgroup variables for the subgroup analysis in Tau PET (Section 5.6.3.5) for primary target regions [Braak 1 and 2, Braak 3 and 4, Braak 5 and 6] with primary reference region (Cerebellum [superior section eroded]). Other target regions and reference regions may be explored.

- Baseline clinical stage (MCI due to AD and mild)
- Tau PET level at baseline (e.g. quartiles),

- Baseline amyloid PET level (where available), for Amyloid Composite ROI SUVR measure as the target region with Cerebellum as the reference region using florbetapir tracer only [e.g. quartiles] (subjects who provided baseline amyloid level)
- Use of AD symptomatic medication at baseline (yes or no)
- Age category (e.g. < 65, 65-<70, 70-<75, >=75)
- Gender (female or male)
- MMSE at baseline (e.g. > median MMSE score at baseline vs. < median MMSE score at baseline)

In addition, the following subgroup analysis may be explored for analysis:

- AD symptomatic concomitant medication change during the study duration (Yes vs. No)
- Subjects who received >=10 infusions vs subjects who received <10 infusions
- Baseline ISLT score
- Time since AD diagnosis
- Time since AD symptom onset

3.5 Analysis Sets

- Enrolled subjects: all subjects who signed informed consent and were assigned a subject identification number.
- Randomized subjects: enrolled subjects who received a randomization treatment assignment from the Interactive Response Technology (BIIB092 or placebo).
- Full Analysis Set (FAS): The Full Analysis Set (FAS) includes all randomized subjects who received study treatment (BIIB092 or placebo). In analyses performed on the FAS, participants will be analyzed, based on the intention-to-treat principle, according to their randomized treatment assignment regardless of treatment received.
- Per-Protocol Analysis Set:
  The Per-Protocol Analysis Set is defined as all subjects in the FAS and
  - had no violations of the following inclusion criteria:
    - Must have evidence of cerebral Aβ accumulation, as determined by an amyloid PET scan or by CSF testing
    - Must have the following at baseline:
      - CDR global score of 0.5 or 1
      - MMSE score of 22 to 30 (inclusive)
      - CDR Memory Box score of ≥0.5
  - had at least 14 infusions

- did not miss ≥4 infusions consecutively
- did not make any change to concomitant AD symptomatic medications during the study

- Safety Analysis Set:

  The Safety Analysis Set includes all randomized subjects who received at least one dose of study treatment (BIIB092 or placebo), essentially the same set of participants included in the FAS.

- Safety MRI Evaluable Set:
  The Safety MRI Evaluable Set is defined as subjects in the FAS who had at least one post-baseline safety MRI scan.

- Serum PK Evaluable Set:
  The serum PK evaluable set is defined as subjects in the FAS who had at least one measurable post-baseline BIIB092 concentration in serum before the End of Study of PCP.

- CSF PK Evaluable Set:
  The CSF PK evaluable set is defined as subjects in the FAS who had at least one measurable post-baseline BIIB092 concentration in CSF.

- Blood PD Evaluable Set:
  The blood PD Evaluable Set is defined as subjects in the FAS who had baseline and at least one post baseline assessment of the specific parameter being analyzed in blood before the End of Study of PCP.

- CSF PD Evaluable Set:
  The CSF PD Evaluable Set is defined as subjects in the FAS who had lumbar puncture (LP), which will be used in analyses, such as subject accounting and summary of AE related to LP. The CSF PD Modified Evaluable Set is defined as subjects in the FAS who have baseline and at least one post baseline assessment of the specific parameter being analyzed in CSF.

- Tau PET Evaluable Set:
  The tau PET Evaluable Set is defined as subjects in the FAS who had tau PET, which will be used in analyses, such as subject accounting and summary of AE related to tau PET. The tau PET Modified Evaluable Set is defined as subjects in the FAS who had a valid baseline and a post-baseline tau PET SUVR measure using the 18F-MK6240 tracer.

- Structural MRI Evaluable Set:
  The Structural MRI Evaluable Set is defined as subjects in the FAS who have an evaluable baseline and a post-baseline structural MRI scan.

- Anti-drug antibody (ADA) Evaluable Set:
  The ADA Evaluable Set is defined as subjects in the FAS who have an evaluable post-baseline ADA sample.

CONFIDENTIAL
The information contained herein may not be used, disclosed, or published without the written consent of Biogen MA Inc.

4 List of Planned Study Analyses

4.1 Interim Analysis

The protocol of 251AD201 covers PC and LTE periods. Therefore, end of PC period analysis will be considered as an interim analysis for this protocol. All planned analyses in this SAP will be finalized.

At the time of PC database lock, an interim analysis of LTE period will be conducted. A separate SAP will be prepared for the analyses of the LTE period and integrated analyses across both portions of the study.

4.2 Final analysis

At the end of the long-term extension after which the study will be finally locked and analyzed.

5 Statistical Analysis Methods

5.1 General Considerations

Summary tables will be presented using descriptive summary statistics. For continuous variables, summary statistics will generally include: number of subjects with data, mean, standard deviation, median, 25% percentile, 75% percentile, minimum and maximum. For categorical variables, this will generally include: number of subjects randomized or dosed, number with data, and the percent of those with data in each category.

5.2 Study Subjects

The summaries in this section will be based on the FAS. Unless otherwise specified, summary tables will be presented by treatment group: placebo, BIIB092 125 mg/4wk, BIIB092 375 mg/12wk, BIIB092 600 mg/4wk, BIIB092 2000 mg/4wk, and total. All of the listings will be presented by treatment group, unless otherwise specified.

5.2.1 Accounting of Subjects

Disposition of subjects will be summarized and the summary data will include number (%) of subjects randomized and dosed, number (%) of subjects in each analysis set, number (%) of subjects who completed the treatment/study, number (%) of subjects who are active in the treatment period, number (%) of subjects who discontinued treatment and/or withdrew from study, and number (%) of subjects who discontinued treatment yet completed the placebo-controlled period. For subjects who discontinued treatment and/or withdrew from study, the reasons for discontinuation and/or withdrawal will be summarized and listed. Subjects excluded from the per-protocol analysis set will also be listed.

In addition, the following will be summarized for country, substudies (CSF and Tau PET), and baseline clinical stage (MCI and Mild):

- Number of subjects dosed
- Number of subjects who completed treatment
- Number of subjects who completed the study

5.2.2 Demographics and Baseline Characteristics

The demographic data including age, gender, ethnicity, race, height, weight, body mass index (BMI), years of formal education, substudy enrollment, and region will be summarized. Age will also be categorized and presented using the following grouping: < 65, 65-<70, 70-<75, >=75. In addition, Asian group will also be summarized for the following subclassification: Chinese, Indian, Japanese, Korean, and other.

Summary of the baseline characteristics of AD includes baseline clinical stage (MCI due to AD or mild AD), years since first AD symptom(s), years since diagnosis of AD, laboratory ApoE status (carrier, non-carrier, and undetermined), baseline AD symptomatic medication use (yes or no; see Section 4.2.3 for definition of AD symptomatic medication use at baseline), ISLT raw score, ISLT z-score, ISLR raw score, ISLR z-score, CDR global score, CDR memory box score, CDR sum of boxes score, CDR cognitive subscore, CDR functional subscore, MMSE, ADAS-Cog 13, ADCS-ADL, ADCS-iADL, ADCS-bADL, EMACC, ADCOMS and iADRS. ApoE carrier will be further classified as homozygote and heterozygote. MMSE will also be categorized by the following subgroups: <22, 22-24, 25-27, and 28-30. Baseline Amyloid PET level will be summarized for Amyloid Composite ROI SUVR measure as the target region with Cerebellum as the reference region, using florbetapir tracer only.

Subject listings will be generated for demographics and baseline characteristics.

The same summary will be also conducted for MCI subjects, Mild subjects, tau PET evaluable set (including subjects enrolled in both tau PET and CSF substudies) and CSF PD evaluable set.

Medical history will be coded using the Medical Dictionary for Regulatory Activities (MedDRA 23.1). The number (%) of subjects with history (including both ongoing and not ongoing medical conditions) will be summarized by system organ class and preferred term. A listing of medical history will be generated.

Previous treatment of AD stopped prior to the date of first infusion, duration of previous therapies and reason for stopping treatment will be summarized by treatment group. Listing of previous AD treatment will also be generated.

Note that ApoE status is defined as below:

- ApoE carrier: E2/E4, E3/E4, E4/E4
  - Heterozygote: E2/E4, E3/E4
  - Homozygote: E4/E4
- ApoE non-carrier: E2/E2, E2/E3, E3/E3

5.2.3 Concomitant Medications and Non-drug Therapies

All concomitant medications will be coded using the World Health Organization (WHO) medication dictionary (version: WHODrug Globa B3 MAR20). All concomitant non-drug therapies will be coded using the MedDRA dictionary. A concomitant medication/therapy will be defined as any therapy that was taken on or after the day of the first dose of study drug. This includes therapies that start prior to the initiation of the first dose if their use continues on or after the date of first dose. To define concomitant use for therapies with missing start or stop dates, the following additional criteria will be used:

- If both the start and stop dates of a therapy are missing, that therapy will be considered concomitant.
- If the start date of a therapy is missing and the stop date of that therapy fall on or after the date of the first dose, that therapy will be considered concomitant.
- If the start date of a therapy is prior to the date of the first dose and the stop date of that therapy is missing and the therapy is listed as continuing, that therapy will be considered concomitant, or
- If the start date of a therapy is prior to the date of the first dose and the stop date of that therapy is missing and the therapy is not listed as continuing, that therapy will be considered non-concomitant.

For a therapy with a partial start and/or stop date, the following imputation method will be used to determine if the event is concomitant:

- If the start day is missing, then impute the first day of the month (01) as the day
- If the start day and month are missing then impute the first day of the year (01January) as the start day
- If the stop day is missing then impute the last day of the month as the day
- If the stop day and month are missing then impute the last day (31December) of the year as the stop day

The number (%) of subjects taking concomitant medication and non-drug therapies will be summarized by treatment group. Listings of concomitant medications and non-drug therapies will be presented.

AD symptomatic medications taken concomitantly at baseline are defined as AD symptomatic medications that were being taken at the time of the first dose, i.e., started prior to the first dose and continued until after the first dose. The number (%) of subjects taking AD symptomatic medications concomitantly at baseline will be summarized. Subjects who have any new or change in AD symptomatic medications after the initiation of study treatment will be summarized by the timing of change, i.e., the number of subjects first changing between Day 1 and Week 24, the number of subjects changing between Week 24 and Week 52, etc., subjects who have changes in multiple intervals may be counted in each interval. The start and stop date/time of AD symptomatic medication will be listed for these subjects.

AD symptomatic medications including the following terms will be considered:

Anticholinesterases, Donepezil, Donepezil hydrochloride, Galantamine, Galantamine hydrobromide, Huperzine A, Mimopezil, Nivabex, Rivastigmine, Rivastigmine tartrate, Robinulneostigmine, Tacrine, Tacrine hydrochloride, Mortha, Energix, Memantine, Memantine hydrochloride, Donamem, and Gemcitabine Hydrochloride.

5.2.4 Protocol Deviations

Protocol deviations identified during site monitoring will be captured in a Protocol Deviation log and categorized as major or minor deviations based on Protocol Deviation Classification. The major protocol deviations will be summarized and listed. The minor protocol deviations will also be listed. Subjects who had incorrect dose assigned by interactive response technology (IRT) will be summarized and listed. This data will be provided by the unblinded monitors after database lock.

5.2.5 Study Drug Exposure and Study Drug Compliance

Number of infusions (BIIB092 or placebo) received will be summarized as a categorical variable (categories as integers from 1 to 20, and 1-5, 6-10, 11-15, and 15-20) as well as a continuous variable. Number of consecutively missed infusions will be summarized as a categorical variable (categories as consecutively missed 2, 3, 4, >=5). Number of weeks on study treatment (BIIB092 or placebo), calculated as (date of last dose – date of first dose +1)/7, will be summarized as a categorical variable (every 8 weeks from 0 to >= 72 weeks) as well as a continuous variable. Percentage of study treatment taken up to the last dose, calculated as (the actual number of infusions / by the number of infusions a subject is expected to take until the date of last infusion)*100, will be summarized as a continuous variable. This table will be presented by treatment group.

A listing of study drug administration records, including dose level at each infusion, cumulative number of infusions and cumulative dose will be provided.

A listing of study drug administration records for placebo subjects who received any doses of active treatment will be provided.

5.2.6 COVID-19 Related Analysis

5.2.6.1 Accounting of subjects who discontinued due to Covid-19

Disposition of subjects will be summarized for subjects who discontinued due to Covid-19. The summary data will include number (%) of subjects who died due to Covid-19, AE due to Covid-19 and all the other reasons which led to discontinue treatment and/or study because of Covid-19.
For subjects who discontinued treatment and/or withdrew from study due to covid-19, the reasons for discontinuation and/or withdrawal will be listed with days on treatment and days on study.

CONFIDENTIAL
The information contained herein may not be used, disclosed, or published without the written consent of Biogen MA Inc.

5.2.6.2 Concomitant Non-Drug Treatment for Covid-19

The number (%) of subjects using concomitant Non-Drug Treatment for Covid-19 will be summarized by treatment group.
Date imputation will be done as per Section 5.2.3

5.2.6.3 Covid-19 Effect on Drug Compliance

Effect of Covid-19 on Drug compliance may be assessed by a summary table. Total number of expected infusions, Number of Missed doses due to covid-19, Average missed dose per subject with its SD, Number of infusions delayed, Average delay in dosing per subject with its SD, and Number of alternate methods of infusion due to covid-19, Average alternate dosing per subject with its SD may be presented.

5.2.6.4 Covid-19 Protocol Deviation

Covid-19 protocol deviation will be summarized. Number of subjects with any protocol deviation, list of Major protocol deviation including Non-Data protocol deviation, Not done, Out of window, Performed at alternate location, Performed by caregiver, Performed remotely, Self-administered, Rater change and Other will be presented.
PD due to covid-19 will be listed including all major and minor deviations by treatment group and subject. Type of PD, PD category, PD text and deviation day will be presented in the listing.

5.2.6.5 Protocol Alternation Due to Covid-19

Protocol alternation due to covid-19 will be listed by treatment group and subject. Type of protocol alternation, Protocol alternation category, Protocol alternation text and Alternation day will be presented in the listing.

5.3 Efficacy Analysis

5.3.1 General Considerations

The efficacy analysis will be presented by treatment group (per randomization). The change from baseline scores of primary efficacy and exploratory efficacy endpoints will be summarized by treatment group at each post-baseline visit for FAS.

The primary, sensitivity, supplementary and additional analyses for the primary efficacy and exploratory efficacy secondary endpoints are listed in Table 2.

Table 2. Analysis for Primary efficacy and exploratory efficacy Endpoints

Endpoint	Analysis	Analysis Set
**CDR-SB**	Primary: Analysis of change from baseline at Week 78 (MMRM)	FAS

	Primary: Dose-response (MCP-MOD)	FAS
	Sensitivity: Censoring after intercurrent events (MMRM), Pattern mixture model; copy increment from reference (CIR) method	FAS
	Per-protocol analysis (MMRM)	Per-Protocol
	Additional: Responder analysis (Logistic regression)	FAS
	Subgroup analysis	FAS
	Slope analysis	FAS
**MMSE, ADAS-Cog13, ADCS-ADL, ISLT, eCog, FAQ**	Primary: Analysis of change from baseline at Week 78 (MMRM)	FAS
	Primary: Dose-response (MCP-MOD) (1)	FAS
	Subgroup analysis (2)	FAS
	Slope analysis	FAS
**EMACC, ADCOMS, iADRS**	Primary: Analysis of change from baseline at Week 78 (MMRM)	FAS
	Subgroup analysis	FAS
**CDR individual domain score, CDR Cognitive score, CDR functional score, ADCS-ADL individual item score, ADCS-bADL, ADCS-iADL, ADAS-Cog 13 individual item score, NPI-10, ISLT, DKERS, DSST and Trails A**	Analysis of change from baseline at Week 78 (MMRM)	FAS

(1) MCP-MOD may be conducted for eCog, FAQ, and other endpoints as exploratory analysis.
(2) Subgroup analysis will only be conducted for MMSE, ADAS-Cog13, ADCS-ADL.

5.3.1.1 Covid-19 consideration

If a participant and his/her study partner is unable to attend a scheduled study visit, selected prioritized clinical assessments (e.g. CDR, ADCS-ADL, ISLT, DKEFS CFT and LFT, CSSRS) may be able to be performed via telephone. These assessments will be included in efficacy analysis.

CONFIDENTIAL
The information contained herein may not be used, disclosed, or published without the written consent of Biogen MA Inc.

5.3.2 Considerations for base MMRM model for change from baseline analyses

A mixed model with repeated measures (MMRM) will be used to analyze changes from baseline in a parameter of interest using fixed effects of treatment, time, treatment-by-time interaction, baseline of the parameter of interest, baseline of the parameter of interest-by-time interaction, baseline MMSE, region (Japan and Australia will be combined), disease stage (MCI versus mild AD), and baseline AD symptomatic medication use. The correlation between repeated measures of the outcomes will be taken into consideration. An unstructured covariance matrix will be used to model the within-subject variance-covariance errors. If the unstructured covariance structure matrix results in a lack of convergence, the heterogeneous first-order autoregressive covariance structure followed by the heterogeneous Toeplitz covariance structure will be used. The least-squares (LS) means, the differences in LS means between each treatment group versus placebo at post-baseline visits, 95% confidence intervals (CIs), and p-values will be presented. In the primary analysis of each endpoint, missing data are assumed to be missing at random [Rubin 1976].

5.3.3 Primary Efficacy Endpoint

5.3.3.1 Primary analysis

Estimand 1: The difference in change from baseline CDR-SB scores at Week 78 in subjects assigned to BIIB92, comparing to placebo group, regardless of what actual treatment is received, acknowledging a participant may miss $\geq$4 infusions, consecutively), change AD symptomatic medication and/or discontinue treatment early.

- Analysis set: all subjects in the FAS
- Variable: The change from baseline CDR-SB scores at Week 78 regardless of intercurrent events (ICEs)
- Analysis set level summary: Least square (LS) mean difference from MMRM model in change from baseline between BIIB092 and placebo
- ICEs and Strategies for Addressing ICEs:

  ICEs include

  - AD symptomatic medication change (treatment policy strategy)
  - Treatment discontinuation (treatment policy strategy)
  - Missing ≥ 4 infusions consecutively (treatment policy strategy)

Since a treatment policy strategy will be used for all ICEs in this estimand, all observed data will be included regardless of miss more than or equal to four infusions consecutively, treatment discontinuation or AD symptomatic medication change. The primary analysis is the mean difference of the change from baseline CDR-SB scores at Week 78 between treatment groups in the FAS who have a baseline and at least one post-baseline CDR-SB score [ICH E9 (R1) Addendum 2014, 2017]. All observed data will be included in the primary analysis,

including data collected after intercurrent events [ICH E9 (R1) Addendum 2017], i.e., treatment discontinuation or a change in concomitant use of AD symptomatic medication.

A mixed model with repeated measures (MMRM) will be used to analyze changes from Baseline in CDR-SB as described in Section 5.3.2. A line plot of adjusted mean change from baseline over time will be provided.

The multiple comparison procedure modelling (MCP-MOD) method (Appendix II) will be used to assess and model dose-response relationships (potential candidate models will include linear, linear-log, quadratic, maximum response [Emax], and logistic models as specified in protocol Section 16.10) while controlling for multiplicity. The dose-response parameter of interest for MCP-MOD will be the LS means at Week 78 for each dose group from the mixed-effects model. Pairwise comparisons of each BIIB092 dose group versus placebo will also be conducted. A line plot of final dose-response relationship may be provided.

If model assumption for MMRM does not hold, nonparametric analysis will be conducted.

5.3.3.2 Sensitivity analysis

The following sensitivity analyses may be performed to assess the robustness of the primary analysis to deviation from the missing-at-random assumption. All the sensitivity analyses will be conducted for the FAS.

Pattern mixture model

The pattern mixture model (PMM) represents a general and flexible framework to model the predictive distribution of missing data conditional on the observed data [Little 1993, 1994]. It allows formulating assumptions regarding missing data in a transparent and clinically interpretable manner.

In the PMM framework, subjects are grouped into missing patterns so that subjects in the same pattern share similar missingness characteristics. In this analysis, missing patterns will be defined according to the reasons for early withdrawal from study as reported on the electronic case report form (eCRF). The following two patterns will be considered:

- Subjects who withdraw due to reasons that may be related to efficacy, including:
    - withdrawal by subject - desire for change in treatment (unrelated to safety)
    - withdrawal by subject – other
    - withdrawal by caregiver - desire for change in treatment (unrelated to safety)
    - withdrawal by caregiver - unable to continue to enable participation due to illness/hospitalization/death
    - withdrawal by caregiver - other
    - physician decision - unrelated to safety
    - death
    - non-compliance with study drug treatment
    - disease progression
    - other
- Subjects who withdraw due to reasons that are unlikely related to efficacy, including:
    - adverse event
    - randomized by mistake
    - lost to follow-up

- pregnancy
- withdrawal by subject - planning for pregnancy
- withdrawal by subject - study visit burden/scheduling conflicts
- withdrawal by subject - concern about study procedures/perceived risks
- withdrawal by subject - relocation (moving or has moved)
- withdrawal by caregiver - study visit burden/scheduling conflicts
- withdrawal by caregiver - concern about study procedures/perceived risks
- withdrawal by caregiver -relocation (moving or has moved)
- protocol deviation
- site terminated by sponsor
- study terminated by sponsor
- technical problems

The pattern (time and rate) of study withdrawal will be displayed by Kaplan-Meier plot for each missing pattern within each treatment group. Subjects who withdraw due to reasons that may be related to efficacy will be penalized and their missing data will be imputed using the copy increment from reference (CIR) method [Carpenter et al. 2013]. Subjects who withdraw due to reasons that are unlikely related to efficacy will be handled using the missing-at-random assumption and their missing data will be imputed using the standard multiple imputation method [Rubin 1987]. Subjects that do not have change from baseline CDR-SB at Week 78 due to reasons other than early withdrawal (such as item missing, out of window, etc.) will be handled using the missing-at-random assumption. For both imputation methods, the following covariates will be included in the imputation model: treatment, baseline of the parameter of interest, baseline MMSE, region, disease stage (MCI versus mild AD), and baseline AD symptomatic medication use.

The imputed datasets will be analyzed by an analysis of covariance (ANCOVA) model adjusting fixed effects of treatment, baseline of the parameter of interest, region, disease stage (MCI versus mild AD), baseline MMSE, and baseline AD symptomatic medication use. The analyzed results from the imputed datasets will be combined based on Rubin's rules [Rubin 1987] assuming that the statistics estimated from each imputed dataset are normally distributed.

If intermediate missing values are encountered (such as data missing at Week 26 but are available at subsequent visits), a Markov chain Monte Carlo (MCMC) method that assumes multivariate normality will be used to impute the intermediate missing values and produce a monotone missing pattern (data with only terminal missing and no intermediate missing) [Li 1988; Schafer 1997].

Copy increment from reference method

The copy increment from reference (CIR) method may be applied to impute the post-withdrawal data for any BIIB92 treated subject who withdraws from study early based on data from the placebo group rather than the subject's own randomized treatment group [Carpenter et al. 2013]. Specifically, for a subject on BIIB92 who withdraws early, his or her mean trajectory after early withdrawal is assumed to be parallel to the mean trajectory of the placebo group, and the difference between the two means is the same as the difference at the time of withdrawal. This method assumes that any benefit gained from previous treatment will be retained, but subjects progress as if they were on placebo after withdrawal from study. For any subject on placebo who withdraws early, his or her post-withdrawal profile will be

imputed following the missing-at-random principle. After all missing data have been imputed, an ANCOVA model adjusting treatment group, baseline of the parameter of interest, baseline MMSE, region, disease stage (MCI versus mild AD), and baseline AD symptomatic medication use will be applied to analyze the change from baseline of CDR-SB.

Censoring after intercurrent events

Estimand 2: The treatment effects of BIIB92 had all participants never missed ≥4 infusions consecutively, change AD symptomatic medication and/or discontinue treatment early

- Analysis set: all subjects in the FAS
- Variable: The change from baseline CDR-SB scores at Week 78 with data after ICEs are set to missing
- Analysis set level summary: Least square (LS) mean difference from MMRM model in change from baseline between BIIB92 and placebo
- ICEs and Strategies for Addressing ICEs:

  ICEs include

  - AD symptomatic medication change (hypothetical strategy)
  - Treatment discontinuation (hypothetical strategy)
  - Missing more ≥ 4 infusions consecutively (hypothetical strategy)

The estimand of this analysis reflects the treatment effect of BIIB092 if the drug is taken as directed. The primary analysis for Estimand 1 will be repeated with the data censored after any of the following intercurrent events (if multiple events occur to the same subject, data after the earliest event will be censored):

- premature discontinuation of the study treatment
- any change to concomitant AD symptomatic medications after the initiation of study treatment
- missing more than or equal to four infusions consecutively

5.3.3.3 Per-protocol analysis

Estimand 3: The treatment effect of BIIB92 in subjects included in PPS regardless if subjects discontinue treatment early

- Analysis set: all subjects in the PPS
- Variable: The change from baseline CDR-SB scores at Week 78 regardless of intercurrent events (ICEs)
- Analysis set level summary: Least square (LS) mean difference from MMRM model in change from baseline between BIIB92 and placebo
- ICEs and Strategies for Addressing ICEs:

  ICEs include

- Treatment discontinuation (treatment policy strategy)

Since a treatment policy strategy will be used for all ICEs in this estimand, all observed data will be included regardless of treatment discontinuation early.

The per-protocol analysis will be done using the same model as the primary analysis (Section 4.3.2.1) and applying in the Per-Protocol Analysis Set (Section 3.5).

5.3.4 Exploratory Efficacy Endpoints

The clinical endpoints assessing AD progression from Baseline include the results of the MMSE, ADAS-Cog13, ADCS-ADL, ISLT, DKEFS Category Fluency, DKEFS Letter Fluency, DSST, Trails A, eCog, FAQ, NPI-10, iADRS, EMACC and ADCOMS.

A mixed model with repeated measures (MMRM) may be used to analyze changes from Baseline as described in Section 5.3.2. A line plot of adjusted mean change from baseline over time will be provided.

For MMSE, ADAS-Cog13, ADCS-ADL, ISLT, eCog, FAQ, and other exploratory efficacy endpoints multiple comparison procedure modelling (MCP-MOD) method (Appendix II) may be used to assess and model dose-response relationships (potential candidate models will include linear, linear-log, quadratic, maximum response [Emax], and logistic models as specified in Section16.10) while controlling for multiplicity. The dose-response parameter of interest for MCP-MOD will be the LS means at Week78 for each dose group from the mixed-effects model. Pairwise comparisons of each BIIB092 dose group versus placebo will also be conducted. A line plot of final dose-response relationship will be provided.

5.3.5 Subgroup Analysis

Subgroup analyses will be performed for the primary endpoint (CDR-SB) and exploratory endpoints (e.g. MMSE, ADAS-Cog13, ADCS-ADL, iADRS, EMACC, and ADCOMS). The subgroups to be considered are in Section 3.4.

Subjects in each subgroup category will be analyzed separately using the same mixed model with repeated measures (MMRM) used to analyze changes from Baseline as described in Section 5.3.2. A forest plots of adjusted mean change from baseline at Week 78 will be provided.

5.3.6 Additional Analysis

5.3.6.1 CDR Subscores, CDR Global Score, ADCS-bADL, ADCS-iADL and individual scores for CDR, ADCS-ADL and ADAS-Cog 13

The CDR is comprised of 6 domains: Memory, Orientation, Judgment and Problem Solving, Community Affairs, Home and Hobbies, and Personal Care. CDR-SB is the sum of the scores for these 6 domains. In addition, two CDR subscores have been proposed [Tractenberg 2005]: a "cognitive" subscore equaling the sum of the Memory, Orientation, and Judgment and Problem Solving box scores, and a "functional" subscore equaling the sum of Community Affairs, Home and Hobbies and Personal Care box scores. For each of the 6 domains, and the

CDR cognitive subscore and CDR functional subscore, the actual value and the change from baseline at each visit will be summarized by treatment group. The same MMRM model as the primary analysis will also be applied to the CDR individual domains, cognitive subscore and CDR functional subscore.

The CDR global score is a composite score obtained by combining the 6 sub-domain scores using a scoring algorithm that weights memory as the primary domain and all other domains as secondary [Morris 1993]. The actual and change from baseline in CDR global score will be summarized (as a categorical variable) by treatment group at each visit. In addition, subjects with ≥ 0.5 worsening in CDR global score may be summarized by treatment group at each post-baseline visit.

ADCS-ADL basic total score (ADCS-bADL) and ADSL-ADL instrumental total score (ADCS-iADL) will also be calculated in addition of ADCS-ADL total score. ADCS-bADL, which contains 6 out of 23 ADCS-ADL items, is measuring the basic self-care tasks which are generally recognizable in all cultures such as feeding, mobility, toileting, bathing, grooming and dressing. The measure and conceptualization of ADCS-iADL, which contains 17 out of 23 ADCS-ADL items, is more complex due to the influence of cultural norms and gender roles that may impact which tasks are even attempted by a patient. As such, scales that measure iADLs tend to include a broad range of activities. The same MMRM model as the primary analysis will also be applied for ADCS-ADL, ADCS-ADL individual items, ADCS-bADL and ADCS-iADL.

Similarly, the same MMRM model used for ADAS-Cog 13, will be applied for ADAS-Cog 13 individual items.

5.3.6.2 Slope analysis

Slope analysis will be conducted to assess the difference between each BIIB092 treatment group and placebo in the slope of clinical measures from baseline up to Week 78. A reduction in the slope of clinical worsening in the BIIB092 treatment group compared with placebo would indicate a slower rate of disease progression, thus reflecting a disease modifying effect of BIIB092. This analysis will be conducted for the CDR-SB, MMSE, ISLT, eCog, ADCS-ADL, FAQ, ADAS-Cog 13, NPI-10, and may be conducted for other exploratory endpoints. A linear mixed model will be used, with change from baseline as dependent variable at each visit and with fixed effects of treatment, time (as a continuous variable), treatment-by-time interaction, baseline of the parameter of interest, baseline of the parameter of interest-by-time interaction, baseline MMSE, region, disease stage (MCI versus mild AD), and baseline AD symptomatic medication use. Random intercept and slope will also be included in the model to characterize subject-specific progression. In addition, a quadratic linear mixed model may also be fitted to address potential non-linearity associated with natural disease progression and/or non-constant treatment effect over the treatment course.

5.3.6.3 Summary of CDR-SB, ADAS-Cog 13 total score, and MMSE by tau PET reduction at Week 78

Subjects may be separated into 2 subgroups based on the magnitude of change on tau PET measures for the primary target and reference regions at Week 78: (1) change from baseline

less than (mean - one standard deviation) of the placebo group at Week 78; (2) change from baseline greater than or equal (mean - one standard deviation) of the placebo group at Week 78. MMRM model or by visit summaries for CDR-SB, ADAS-Cog 13 total score, and MMSE may be conducted on these 2 subgroups by treatment groups.

5.3.6.4 Responder analysis of CDR-SB

To further assess whether subjects on BIIB092 progress differently from those on placebo, responder analysis may be conducted. The responders will be determined by a threshold of the primary endpoint, i.e., subjects whose change from baseline CDR-SB at Week 78 is smaller than the threshold will be classified as responders and otherwise will be classified as non-responders. All subjects with missing data at Week 78 will be classified as non-responders.

The continuous responder curve that displays the percentage of responders under a wide range of threshold values will be presented by treatment group. The responder analysis may be conducted for threshold value 0.5 or 1.5, i.e., subjects whose change from baseline CDR-SB at Week 78 $\leq$ 0.5 may be classified as responders. The number of responders and the response rate may be summarized by treatment group. In addition, the dichotomized response, responder vs. non-responder, may be modeled using a logistic regression with the covariates: fixed effects of treatment, baseline of CDR-SB, baseline of MMSE, region, disease stage (MCI versus mild AD), and baseline AD symptomatic medication use (Y/N). Since all missing data will be considered as non-response, which is a special form of missing-not-at-random, this analysis can provide additional insights for the robustness of the primary analysis results.

5.4 Safety Analysis

5.4.1 General Considerations

For subjects who enrolled in LTE period, AEs and SAEs started after Week 78 will be entered and reported in LTE period.

5.4.1.1 Analysis Set

The Safety Analysis Set will be used for safety analyses of AEs, SAEs, clinical laboratory data, Columbia Suicide Severity Rating Scale (C-SSRS) data, ECG data and vital sign data. The safety MRI evaluable set will be used for the analysis of safety MRI data.

5.4.1.2 Safety treatment groups

Different from the randomization treatment groups, if a subject who was randomized to placebo group accidentally received one or more doses of the active treatment during the study, he/she will be classified as either low (BIIB092 125 mg/4wk or BIIB092 375 mg/12wk), medium or high dose group according to the majority of active treatment dose he/she received, for all the safety analyses (Section 3.5). If a subject randomized to BIIB092 treatment groups but received all placebo throughout the study, this subject will be counted in placebo group. A listing of such subjects will be provided, as described in Section 5.2.5. Safety treatment groups will be

the same as the randomization treatment groups for other subjects (subjects randomized to active treatment groups, and subjects randomized to placebo without any accidental active dose).

5.4.1.3 Incidence, incidence proportion and incidence rate

- Incidence and incidence proportion will be provided in incidence tables. Incidence is defined as the number of subjects who experienced an event. Incidence proportion is defined as the number of subjects who experienced an event divided by total number of subjects in the analysis set, i.e., percentage. Each subject will be counted only once within each category.
- Incidence and incidence rate will be provided in incidence rate tables. Incidence rate of an event based on the entire follow-up time – defined as the number of subjects who experienced an event divided by the total of entire follow-up time among the subjects in the analysis set (e.g., incidence rate per 100 subject-years). The entire follow-up time for a subject (subject-years) is defined as the sum of all subjects' follow-up time, where a subject's follow-up time is calculated as the number of days (inclusive) from first dose of study drug until the last day on study, divided by 365.25. Each subject will be counted only once within each category.

5.4.2 Clinical Adverse Events

5.4.2.1 Treatment-emergent AEs (TEAEs)

All AEs will be analyzed based on the principle of treatment emergence. A treatment-emergent AE is defined as an AE that started or worsened after the start of first infusion of study treatment.

To define treatment emergence for AEs with missing start or stop date or time the following additional criteria will be used:

- if both the start and stop dates for a particular AE are missing, then that AE is considered treatment emergent
- if the start date for a particular AE is missing and the stop date/time falls after the first dose date/time, then that AE is considered treatment emergent
- if the start date for a particular AE was the same as the first dose date, and the start time was missing, then that event is considered treatment emergent

For AEs with a partial start date, the following imputation method will be used to determine if the event is treatment emergent:

- When only the day is missing, and the year/month is equal to the year/month of first dose date, and the AE stop date is missing or on/after the first dose date, then use the first dose date as AE start date. Otherwise impute the AE start date as the first day (1) of the AE start month
- When the AE start month is missing, and the year equals to the year of first dose date, and the AE stop date is missing or on/after the first dose date, then use the first dose date as AE start date. Otherwise impute the AE start date as the first day (01January) of the AE start year

For AEs with a partial end date, the following imputation method will be used:

- When only the day is missing, and the year/month equal to the year/month of the last date on study, then use the last date on study as AE stop date. Otherwise impute the AE stop date as the last day of the AE stop month
- When the AE stop month is missing, and the year equals to the year of last date on study, then use the last date on study as AE stop date. Otherwise impute the AE stop date as the last day (31December) of the AE stop year

Only TEAEs will be included in the tables, unless otherwise specified. All SAEs (including pre-dosing SAEs) and AEs (including pre-dosing AEs related to tau PET) will be included in the listing, with an indicator of pre-dosing or treatment emergent.

5.4.2.2 Summary and incidence analysis

Overall summary of AE table will summarize the number of subjects with any AE, with any AE by maximum severity, the number of subjects with any related AE (related to study drug as assessed by investigator), the number of subjects with SAE, the number of subjects with related SAE, the number of subjects with AE leading to drug withdrawal, the number of subjects with AE leading to of subjects with a fatal event. This table will be done by treatment group.

The sorting order for AE incidence tables, unless otherwise specified, will be by decreasing frequency order of “BIIB092 total” column within each category in the tables presented by treatment group. A subject is counted only once within each category in each table. For example, for the table of AEs by system organ class and preferred terms sorted by decreasing frequency presented by treatment group, system organ class will be presented in decreasing frequency order of BIIB092 total column, and within each system organ class, preferred terms will be presented in decreasing frequency order of BIIB092 total column. A subject is counted only once within each system organ class and preferred term.

The following AE incidence tables will be provided (presented by treatment group, unless otherwise specified):

1. AEs by system organ class and preferred term sorted by decreasing frequency
2. AEs by system organ class and preferred term sorted by alphabetical order
3. AEs by system organ class
4. AEs at least 2% higher in incidence by system organ class and preferred term for BIIB092 2000 mg/4wk compared to placebo
5. AEs by preferred term
6. Adverse events with an incidence of 5% or more in any treatment group by preferred term
7. AEs by maximum severity by system organ class and preferred term by decreasing frequency (System organ class will be presented alphabetically. Preferred terms will be presented in decreasing frequency order. Maximum severity will be presented within each preferred term in the order of mild, moderate, severe, unknown and total. A subject will be counted only once at the maximum severity within each system organ class and preferred term.)

8. AEs by maximum severity by preferred term
9. Severe AEs by system organ class and preferred term by decreasing frequency
10. Severe AEs by preferred term
11. Related AEs by system organ class and preferred term by decreasing frequency
12. AEs related to tau PET ligand by system organ class and preferred term by decreasing frequency (Subjects in tau PET evaluable set)
13. SAEs by system organ class and preferred term by decreasing frequency
14. SAEs by preferred term
15. Related SAEs by system organ class and preferred term by decreasing frequency
16. SAEs with fatal outcome by system organ class and preferred term by decreasing frequency
17. AEs that led to drug interrupted by system organ class and preferred term by decreasing frequency
18. AEs that led to discontinuation of study treatment by system organ class and preferred term by decreasing frequency
19. AEs that led to withdrawal from study by system organ class and preferred term by decreasing frequency
20. AEs related to lumbar puncture (LP) by system organ class and preferred term (Subjects in CSF PD evaluable set)

The following listings will be provided.

1. Listing of AEs
2. Listing of SAEs (including pre-dosing SAEs)
3. Listing of AEs that led to infusion interruption
4. Listing of AEs that led to discontinuation of study drug
5. Listing of AEs that led to withdrawal from study
6. Listing of AEs related to tau PET ligands
7. Listing of SAEs with fatal outcome
8. Listing of AEs related to lumbar puncture (LP)
9. Listing of death

5.4.2.3 Incidence rate analysis

Follow-up adjusted incidence rate of AEs for the placebo-controlled period may be summarized by SOC and PT as below

- Incidence rate of an event based on the entire follow-up time – defined as the number of subjects who experienced an event divided by the total of entire follow-up time among the subjects in the analysis population. The entire follow-up time for a subject

is defined as the time from the first dose until the last day on study. Each subject will be counted only once within each category.

5.4.2.4 Infusion reactions

Infusion reactions will be identified through 1) temporal association, defined as those adverse events which occur on the day of an infusion or the subsequent two calendar days after an infusion; and 2) through a custom MedDRA search of preferred terms. A serious infusion reaction is a serious adverse event which is identified by one or both methods. An overall summary of infusion reactions will be provided with the number of subjects (n, %) with any infusion reaction; with any infusion reaction identified by temporal association only; with any infusion reaction identified through the custom search (Appendix V) only; and any infusion reaction identified through both methods. A listing of infusion reactions will be provided. Additionally, the following incidence proportion tables will be provided:

1. Infusion reactions that occurred in temporal association to an infusion by SOC and PT
2. Serious infusion reactions that occurred in temporal association to an infusion by SOC and PT
3. Infusion reactions identified through custom search criteria by PT
4. Serious infusion reactions identified through custom search criteria by PT
5. Infusion reactions (temporal association or custom search) by 12-week intervals by PT

5.4.2.5 Adverse event of special interest

Anti-drug antibody is an AESI in the BIIB092 clinical development program. Adverse events representing potential immune/hypersensitivity reactions will be identified using customized MedDRA search criteria, including: Anaphylactic reaction SMQ narrow, Allergic conditions NEC HLT, and miscellaneous PTs. Further details are given in (Appendix IV)10

An incidence proportion table for potential immune/hypersensitivity reactions by PT, and similarly for potential immune/hypersensitivity reactions for subjects with and without treatment emergent positive anti-drug antibody (ADA) results (as defined in Section 5.7) by PT may be presented. A listing of potential immune/hypersensitivity reactions will be provided.

The following analyses (incidence proportion only) may be performed to explore the relationship between ADAs and the safety of BIIB092:

- AEs for subjects with and without positive treatment emergent ADAs
- SAEs for subjects with and without positive treatment emergent ADAs

5.4.3 Clinical Laboratory Data

The following clinical laboratory parameters are assessed in the protocol:

- Hematology: red blood cell count, platelet count, hemoglobin level, hematocrit, mean corpuscular volume, mean corpuscular hemoglobin, mean corpuscular hemoglobin

concentration, and total white blood cell count with absolute counts and percentages of neutrophils, monocytes, lymphocytes, eosinophils, and basophils

- Blood chemistry: total protein, albumin, creatinine, BUN, uric acid, bilirubin (total and direct), alkaline phosphatase, ALT, AST, lactate dehydrogenase, gamma-glutamyl transferase, glucose, calcium, phosphorus, bicarbonate, chloride, sodium, and potassium
- Urinalysis: color, specific gravity, pH, protein, glucose, leukocyte esterase, blood, and ketones (and microscopic examination, if abnormal)
- Coagulation: Activated Partial Thromboplastin Time (APTT), Prothrombin Intl. Normalized Ratio (INR), Prothrombin Time (PT)

All the laboratory tables and listings, unless otherwise specified, will be presented by treatment group.

5.4.3.1 Quantitative analyses

For numeric laboratory parameters, actual values, change from baseline and percent change from baseline will be summarized by visit. number of evaluable subjects, mean, standard deviation, 25% and 75% quartiles, min and max values will be presented at each visit.

For coagulation, only actual value summaries will be presented.

Plots of actual values and change from baseline for numeric laboratory parameters at each visit will be provided.

Listings of individual laboratory measurements by patients for all the parameters will be provided.

Visit Windows for by visit summaries

For Laboratory data that are summarized by visit, assessment from all scheduled visits, EOT visit, EOS visit and unscheduled visits will be mapped to an appropriate analysis visit using a windowing scheme (Appendix I).

5.4.3.2 Qualitative analyses

For all qualitative analyses, all values will be included (not just the "analyzed record" within each visit window in the quantitative analyses).

Shift analyses

Laboratory data will be summarized using shift tables where appropriate. Each subject's hematology, blood chemistry and urinalysis values will be flagged as "low", "normal", or "high" relative to the normal ranges of the central laboratory or as "unknown" if no result is available.

For each parameter, the analysis will be based on subjects with at least one post-baseline value. Shifts from baseline to high/low status will be presented for hematology, blood chemistry

coagulation, serology, and urinalysis. Shift to low includes normal to low, high to low, and unknown to low; Shift to high includes normal to high, low to high, and unknown to high. Subjects need to have at least one post-baseline evaluation and a baseline value not low or high (including missing) in order to be included in the analysis for corresponding categories in the analyses.

Potentially Clinically Significant laboratory abnormalities analyses

For hematology, blood chemistry and urinalysis, the number of subjects with potentially clinically significant laboratory abnormalities post-baseline will be summarized for the parameters provided in Table 3. Subjects need to have at least one post-baseline evaluation and a baseline value not potentially clinically significant (including missing) in order to be included in the analysis.

Table 3. Criteria to Determine Potentially Clinically Significant (PCS) Laboratory Abnormalities

**Clinical Laboratory Outlier Criteria**		
**Parameter name**	**PCS Low**	**PCS High**
**HEMATOLOGY**		
White blood cells	<3.0 x 10^9/L	>16 x 10^9/L
Lymphocytes	<0.8 x 10^9/L	>12 x 10^9/L
Neutrophils	<1.5 x 10^9/L	>13.5 x 10^9/L
Monocytes	N/A	>2.5 x 10^9/L
Eosinophils	N/A	>1.6 x 10^9/L
Basophils	N/A	>1.6 x 10^9/L
Red blood cells	≤3.5 x 10^{12}/L	≥6.4 x 10^{12}/L
Hemoglobin - Females	≤95 g/L	≥175 g/L
Hemoglobin - Males	≤115 g/L	≥190 g/L
Hematocrit - Females	≤32%	≥54%
Hematocrit - Males	≤37%	≥60%
Platelet count	≤75 x 10^9/L	≥700 x 10^9/L
**BLOOD CHEMISTRY**		
Alanine aminotransferase (ALT)	N/A	>3 x ULN
Aspartate aminotransferase (AST)	N/A	>3 x ULN
Alkaline phosphatase (ALP)	N/A	>3 x ULN
Total bilirubin	N/A	>1.5 x ULN
Blood urea nitrogen (BUN)	N/A	≥10.7 mmol/L
Creatinine	N/A	≥176.8 umol/L
Sodium	≤126 mmol/L	≥156 mmol/L
Potassium	≤3 mmol/L	≥6 mmol/L
Chloride	≤90 mmol/L	≥118 mmol/L
Bicarbonate	≤16 mmol/L	≥35 mmol/L
Glucose	≤2.2 mmol/L	≥9.7 mmol/L
Calcium	≤2 mmol/L	≥3 mmol/L
Phosphorus	≤0.6 mmol/L	≥1.7 mmol/L
Albumin	≤25 g/L	≥625 g/L
Total protein	≤45 g/L	≥100 g/L
**URINALYSIS**		
Glucose	N/A	≥ ++++
Ketones	N/A	≥ ++++
Protein	N/A	≥ ++

Clinical Laboratory Outlier Criteria		
**Parameter name**	**PCS Low**	**PCS High**
ULN = upper limit of normal		

Potential serious hepatotoxicity

Potential serious hepatotoxicity is defined as ALT or AST > 3x ULN and total bilirubin > 2x ULN at any time post-baseline, not necessarily concurrent. A scatterplot of the maximum post-baseline ALT or AST value relative to ULN and maximum post-baseline total bilirubin value relative to ULN (not necessarily concurrent) for each subject will be provided. A line plot of ALT, AST, ALP and total bilirubin values over time for subjects with potential serious hepatotoxicity will be provided. In addition, a summary of subjects with ALT > 1x ULN, >3x ULN, >5x ULN, >10x ULN or >20x ULN, subjects with AST > 1x ULN, >3x ULN, >5x ULN, >10x ULN or >20x ULN, subjects with total bilirubin >1x ULN or >2x ULN, subjects with ALP >1x ULN or >1.5x ULN, and subjects with AST or ALT > 3x ULN post-baseline accompanied by concurrently elevated total bilirubin >1.5x ULN or > 2x ULN will be presented. A listing of subjects with potential serious hepatotoxicity will be provided with the concurrent records labeled. Concurrent is defined as on the same day.

5.4.4 C-SSRS Data

Suicide-related events based on the Columbia Suicide Severity Rating Scale (CSSRS) at post-baseline visits will be summarized by treatment group. Suicidal ideation events include: (1) Wish To Be Dead, (2) Non-Specific Active Thoughts, (3) Active Thoughts Without Intent To Act, (4) Active Thoughts With Some Intent—No Plan, (5) Active Thoughts with Plan and Intent. Suicidal behavior events include: (6) Preparatory acts or behavior, (7) Aborted Attempt, (8) Interrupted Attempt, (9) Actual Attempt, (10) Suicidal behavior, (11) Completed Suicide. Another “Yes/No” question of whether the subject has engaged in non-suicidal self-injurious behavior is also collected. The analysis will be based on the Safety Analysis Set.

Number of subjects in each ideation category at any post-baseline visit, number of subjects in each suicidal ideation and suicidal behavior category at any post-baseline visit, number of subjects with at least one suicidal ideation or behavior event at any post-baseline visit, and subjects who have engaged in non-suicidal self-injurious behavior at any post-baseline visit will be summarized. A listing of subjects with suicide-related events will be provided.

5.4.5 ECG Data

Actual and change from baseline values of numeric ECG parameters will be summarized by visit. number of evaluable subjects, mean, standard deviation, 25% and 75% quartiles, min and max values will be presented at each visit.

Summary of ECGs (number of normal, <abnormal, not adverse event>, or <abnormal, adverse event>) at scheduled visits will be presented by treatment group.

Shift table from normal or unknown ECG at baseline to abnormal post-baseline ECG (<abnormal, not adverse event>, or <abnormal, adverse event>) will be summarized. Denominator will be number of subjects who have a baseline value not abnormal (including unknown) and at least one post-baseline value.

In addition, PR and QTcF values will be summarized for below categories:

- QTcF interval (msec) at post-baseline in below categories:
  - >450
  - >480
  - >500
- QTcF increase from baseline (msec) in below categories:
  - >30
  - >60
- PR interval (msec) at post-baseline in below categories:
  - ≤ 200
  - > 200

Visit Windows for By Visit Summaries

For ECG data that are summarized by visit, assessment from all scheduled visits, EOT visit, EOS visit and unscheduled visits will be mapped to an appropriate analysis visit using a windowing scheme (Appendix I).

5.4.6 Vital Sign Data

Vital sign parameters include temperature, diastolic blood pressure, systolic blood pressure, heart rate, and respiration rate. The descriptive statistics for actual values and change from baseline will be summarized over time for each treatment group. Plot of mean vital sign values at each visit will be provided. Vital signs will be measured with the subject supine and after the subject has been resting for at least 10 minutes. A by-patient listing will also be presented.

The analysis of vital signs will also focus on the incidence of clinically relevant outliers based on the following criteria (Table 4 – study specific and Table 5 – required by STAN V2). The number of clinically relevant outliers determined by each criterion will be summarized by treatment group.

Table 4. Criteria Used to Assess Potential Clinically Relevant Outliers in Vital Signs

Variable	Low	High
Systolic Blood Pressure	<90 mm Hg post-BL or ⩾20 mm Hg decrease from Baseline (BL)	>180 mm Hg post-BL or ⩾20 mm Hg increase from BL
Diastolic Blood Pressure	<50 mm Hg post-BL or ≥15 mm Hg decrease from BL	>105 mg Hg post-BL or ⩾15 mm Hg increase from BL
Heart Rate	<50 bpm post-BL or ⩾ 15 bpm decrease from BL	>120 bpm post-BL or ≥15 bpm increase from BL
Temperature	>2 degree C decrease from BL	>38 .5℃ or >2℃ increase from BL
Respiration Rate	< 10 breaths per minute or ≥ 50% decrease from BL	>25 breaths per minute or ≥50% increase from BL

BL= baseline; bpm = beats per minute

Table 5. Criteria for post-baseline vital sign abnormalities

Variable	Criteria
Temperature	<36 degrees C<br>>38 degrees C
Heart rate	<60bpm<br>>100bpm
Systolic blood pressure	<90 mmHg<br>>140 mmHg<br>>160 mmHg
Diastolic blood pressure	<50 mmHg<br>>90 mmHg<br>>100 mmHg
Weight	7% or more increase from BL<br>7% or more decrease from BL
Respiratory rate	<12 breaths/min<br>>20 breaths/min
BL= baseline; bpm = beats per minute	

The number of subjects evaluated and the number and percentage of subjects with clinically relevant outliers will be presented by treatment group.

Visit Windows for By Visit summaries

For vital sign data that are summarized by visit, assessment from all scheduled visits, EOT visit, EOS visit and unscheduled visits will be mapped to an appropriate analysis visit using a windowing scheme (Appendix I).

5.4.7 Physical Examination

Since clinically significant findings during physical examinations will be recorded as AEs, a listing of physical examination data will be presented.

5.4.8 Neurological Examination

Since clinically significant findings during neurological examinations will be recorded as AEs, a listing of neurological examination data will be presented.

5.4.9 Safety MRI Data

The following MRI data will be summarized by treatment group using safety MRI evaluable set:

- Number of subjects with new or worsening of vasogenic edema (VE) at post-baseline
  - New VE or questionable VE is considered as subjects who has a VE which is worsening as post-baseline or for the following:
    - The subject has answered "Not-Applicable" at any post-baseline visit for question: VE on this MRI as compared to previous MRI

  - The subject has answered "VE present and increased in size" at any post-baseline visit for question: VE on this MRI as compared to previous MRI
  - Worsening of VE from baseline is defined as any severity increase compared to baseline. The following sequence indicates the increase of the severity:

    No VEVE present < Questionable presence of VE (VE (Mild Severity) < Questionable presence of VE (Moderate Severity) < Questionable presence of VE (VE (Severe Severity) < VE present ((Mild Severity) < VE present ((Moderate Severity) < VE present (Severe Severity)

- Number of subjects with new microhemorrhages (mH) findings at post-baseline
  - New mH is define as subjects who has mH increased in number and/or size at any post-baseline comparing to the baseline. If a subject has no mHs at baseline, then new mH is defined as any initial identification at any post-baseline visit.
- Number of subjects with macrohemorrhages (MA) (>1cm) at post-baseline
- Number of subjects with superficial siderosis (SS) (>1cm^3) at post-baseline
- Number of subjects with SS (≤1cm^3) at post-baseline

5.4.10 COVID-19 Related Safety analysis

Overall summary of COVID-19 adverse events will be presented. Number of subjects with Covid-19 AE, severity, related events, serious events, related serious events, events leading to drug withdrawal, events leading to study discontinuation and AE with Fatal outcomes may be included in the summary.

AEs by system organ class and preferred term sorted by decreasing frequency will also be presented.

5.5 Pharmacokinetics Analysis

The Serum PK evaluable set as defined in Section 3.5, will be used for the description of the serum concentration data, and for the estimation of serum PK parameters. The CSF PK evaluable set as defined in Section 3.5, will be used for the description of CSF concentration data. PK analysis will be conducted with serum and CSF concentrations of BIIB092 by visit and dose group. Subjects who receive BIIB092 125mg once every 4 weeks will be analyzed separately from those who receive 375mg once every 12 weeks. For subjects whose serum and CSF sample collected after LTE first infusion will be excluded.

Atypical drug concentrations (e.g., very low or very high) will be excluded from the analysis, if no apparent explanation exists. Concentration observations will also be removed from the data set if

i) corresponding dosing or sampling times are missing or cannot be reconstructed,
ii) large deviations between actual administered dose and nominal dose exists,
iii) large deviations between scheduled and actual sampling days or times exists,
iv) large deviations between actual and nominal dose administration time exists.

All deletions of data points will be appropriately documented.

Population PK analysis may be conducted to estimate BIIB092 population PK parameters and to identify potential covariates (e.g., demographics, body weight, and anti-BIIB092 antibodies) on the variability of BIIB092 PK. Results will be presented in a separate report.

CONFIDENTIAL
The information contained herein may not be used, disclosed, or published without the written consent of Biogen MA Inc.

In addition, an exposure-response (E-R) analysis will be conducted by Pharmacometrics group to detect any E-R relationship trend and potential covariate using any primary or secondary endpoint and BIIB092 exposure metrics as deemed appropriate. Other tables and figures based on the population PK and E-R analyses will be generated by Clinical Pharmacology and Pharmacometrics (CPP) team and will be included in a separate report from CPP. This analysis may not be included in the CSR.

5.5.1 Serum and CSF PK Concentration Data

Individual serum and CSF concentrations will be listed for BIIB092. Concentrations below the lower limit of quantification (LLOQ) will be indicated by "BLQ". Differences between scheduled and actual sampling times may be listed for these subjects, along with the percentage differences between nominal and actual dose amount. Additional listings may be generated as deemed necessary.

Descriptive statistics (N, arithmetic mean, standard deviation, geometric mean, CV, median, minimum, and maximum), will also be used to summarize serum PK and CSF concentrations of BIIB092 by visit/scheduled time points and dose group. For the purpose of calculating typical descriptive statistics (n, mean, SD, %CV, geometric mean, geometric %CV, median, minimum, and maximum) for serum PK, BLQ value on pre-day 1 of study treatment will be set to 0, and all post first dose of study treatment BLQ values will be set to half the LLOQ value. In linear and semi-logarithmic plots, all BLQ values will be treated as LLOQ/2. Mean CSF concentrations that are BLQ will be presented as BLQ, and the SD and %CV will be reported as not applicable. When summarizing concentrations or PK parameters in serum and CSF, a minimum of 2 values are required to show the arithmetic mean and geometric mean, and at least 3 values are required to show the standard deviation and coefficient of variation (CV).
Serum and CSF concentrations of BIIB092 will be plotted versus time by dose group on both a linear and a logarithmic scale. Additional plots may be included as deemed necessary.

CSF to serum concentration ratio will be computed using pre-infusion concentrations at week 12, 48, and 76. Individual ratios along with descriptive statistics (N, arithmetic mean, standard deviation, geometric mean, CV, median, minimum, and maximum) at 12, 48, and 76 weeks, by dose will be listed. Any BLQ value will be excluded from CSF to serum ratio calculation, and exclusions will be appropriately documented. Individual CSF vs. serum concentrations above LLOQ (for both serum and CSF) will be plotted (scatter plot) by week, color coded by dose.

5.5.2 Serum PK Parameters

The following PK parameters will be listed per visit, as data permits, from serum concentration data:

Parameter	Definition/Calculation	Units
C_{max}	Observed maximum serum BIIB092 concentration collected at end of infusion	ug/mL
C_{trough}	Observed troughtrough serum BIIB092 concentration collected at end of dosing interval (before next infusion starts)	ug/mL

Individual PK parameter data will be listed. Descriptive statistics (N, mean, standard deviation, CV, median, minimum, and maximum) will be used to summarize the PK parameters by visit

and dose. Geometric means (by visit and dose) will also be presented. Box plots and plots of individual C_{max} and C_{trough} over time will be provided.

5.6 Biomarker

5.6.1 Pharmacodynamics Analysis

The CSF PD evaluable set or CSF PD modified evaluable set as defined in Section 3.5, will be used for the statistical modeling of CSF PD data.

The actual, change from baseline, and percentage change from baseline of CSF N-terminal tau and CSF p-tau, and possibly CSF markers of synaptic change or neurofilament will be summarized to evaluate pharmacodynamics effect after multiple IV infusions of BIIB092. For descriptive statistics, BLQ is imputed as LLOQ/2. For individual subject listings, BLQs are listed as BLQ. For CSF to serum ratio calculation, BLQ are excluded, as a ratio using the imputed values will lead to unreasonable estimates. Summary statistics will be generated showing N, mean, median, standard deviation, Q1, Q3, minimum and maximum results over time by treatment group for CSF N-terminal tau.

Pearson and Spearman correlations between PD parameters (CSF n-terminal tau, CSF p-tau and possibly CSF synaptic or neurofilament marker change from baseline values) and CDR-SB, ADAS-Cog-13 total score, ADCS-ADL total score and MMSE scores change from baseline values may be evaluated using Pearson and Spearman correlation at Week 52 and Week 78, respectively. Pearson and Spearman partial correlations adjusting for age, corresponding baseline PD parameters and baseline clinical assessment may also be explored.

For subjects who had both CSF and tau PET assessments. Their data may be used to evaluate the correlation between CSF PD parameters and key tau PET parameters at Baseline, 1 year (Week 48 for CSF and Week 52 for tau PET) and 18 months (Week 76 for CSF and Week 78 for tau PET). Pearson and Spearman correlations and partial correlations adjusting for age and corresponding baseline values may be evaluated.

In addition, MMRM model specified in Section 5.3.2 without region as one of the covariates will be done for p-tau. Age may be considered as one of the covariates. In addition, these analyses might be considered for total tau, N-terminal tau and other fluid biomarkers. For PD biomarker by visit summaries and MMRM models, the analysis visit should be defined using visit windows (Table 16 in Appendix I). Week 12 data will not be used in the MMRM due to small sample size.

Adjusted mean (± standard error) plots of change from baseline of CSF N-terminal tau and p-tau over time by treatment group and mean % change of CSF N-terminal tau and p-tau over time by treatment group may be presented. The exposure response relationships may be explored graphically as appropriate.

Other Fluid Biomarker Analysis

Other PD parameters analyzed may include, but not limited to, other measures of phospho-tau, biomarkers of synaptic change and neurofilament in CSF and in some cases blood if available.

Data for these exploratory potential biomarker candidates related to BIIB092 biological activity or disease progression may be summarized using descriptive statistics and will be presented by treatment group.

5.6.2 Structural MRI Analysis

Structural magnetic resonance imaging (MRI) results analyzed may include, but will not be limited to, volume measures of hippocampus, whole brain, whole cortex and lateral ventricles. The analyses will be based on Structural MRI evaluable set as defined in Section 3.5.

Structural MRI is performed on all randomized subjects at screening visit, Week 28, Week 52, Week 78 and early termination visits. Structural MRI readings with QC failure or major scanner upgrade will not be included in the analysis.

5.6.2.1 By visit summary and MMRM model

The actual values and change from baseline values will be summarized by treatment groups (placebo, low dose, medium dose and high dose) and by visit for each of the ROI measurements up to Week 78 for Structural MRI evaluable set.

In addition, some of the parameters measuring volumetric MRI ROIs will be analyzed as a percentage of total intracranial volume at baseline (i.e. "% of TIV"). The selected ROIs (e.g., lateral ventricles) volumes (% of TIV) will also by summarized by treatment for each visit for Structural MRI evaluable set.

A mixed model with repeated measures (MMRM) will be used to analyze changes from baseline as described in Section 5.3.2, including age as covariate. The MMRM analyses will be performed on both the raw volume (all ROIs) and the adjusted volume (% of TIV, for selected ROIs only, e.g. lateral ventricles).

5.6.2.2 Pearson and Spearman correlation

Pearson and Spearman correlations between change from baseline volume MRI at Week 78 and change from baseline CDR-SB at Week 78 will be conducted by treatment groups. Pearson and Spearman partial correlation adjusting for age, corresponding baseline volume MRI measure, baseline TIV and baseline CDR-SB will also be conducted by treatment groups at Week 78. The estimated correlation coefficients, p-values and the 95% confidence intervals will be provided. Given that reductions in volume MRI may precede clinical effects (e.g., slowing of clinical decline), Pearson and Spearman correlations and partial correlations between change from baseline volume MRI at Week 52 and change from baseline CDR-SB at Week 78 might also be conducted. Similar models may be conducted for MMSE, ADAS-Cog-13 total score, and ADCS-ADL total score.

5.6.3 Tau PET Analysis

5.6.3.1 Tau PET substudy

The tau PET substudy (using ^{18}F-MK6240 tracer only) will include a subset of subjects in USA and Australia. Subjects enrolled into the tau PET substudy will receive serial ^{18}F-MK6240 Tau

PET scans at Screening, Week 52 and Week 78/Early Termination to investigate the effect of BIIB092 on cerebral tau pathology. The analyses for Tau PET will be based on Tau PET evaluable set or Tau PET modified evaluable set as defined in Section 3.5.

5.6.3.2 Tau PET target and reference regions

Tau PET standardized uptake value ratio (SUVR) is a quantitative measure of cerebral tau level. The SUVR was calculated as the ratio of tracer binding in target brain region (brain regions expected to harbor tau pathology) to reference region (brain region devoid of tau pathology).

For the SUVR measures the following target and reference regions may be analyzed.

- The primary target regions include: Braak 1 and 2, Braak 3 and 4, and Braak 5 and 6.
- Secondary target regions may include: Posterior composite, Temporal composite, Amyloid composite, Frontal cortex, Parietal cortex, Occipital cortex, Anterior cingulate cortex, Posterior cingulate cortex, Lateral temporal cortex, Inferior temporal cortex, Medial temporal lobe, Lateral temporal lobe.
- The primary reference region is Cerebellum (superior section eroded).
- Other exploratory reference regions may include: cortex (superior section eroded) and Cerebellum (superior and inferior section eroded).

In addition to SUVR measures, Tau PET extent measures may also be analyzed. The extent measure is an exploratory approach of quantifying the spatial extent of tau pathology in brain regions of interest. The Tau PET extent measures for Braak 1 and 2, Braak 3 and 4, Braak 5 and 6, Whole cortex using Cerebellum (superior section eroded) as reference region may be analyzed.

SUVR and extent measures derived from additional target and reference region combinations may be explored.

5.6.3.3 Tau PET primary analysis

The actual and change from baseline tau PET measures will be summarized by treatment groups and by visit for each target region and the primary reference region.

For tau PET by visit summaries and MMRM models, the analysis visit should be defined using visit windows (see 0 in Appendix I). The rationale is to use consistent analysis visit windows as for the efficacy endpoints for Week 52 and Week 78. If there is more than 1 value in the same analysis visit window (>1 value for the same analysis visit) for a certain parameter for a subject, then select the closest record to the target visit day for the by visit analysis. If they are with the same distance from the target visit day, then select the later one for the by visit analysis.

MMRM model

A mixed model with repeated measures (MMRM) will be used to analyze changes from Baseline in tau PET measures as described in Section 5.3.2 without geographic region as one of the covariates for all tau PET primary target regions and primary reference regionregion. Age is adjusted in the model as a continuous covariate. A line plot of adjusted mean change

from baseline over time will be provided. A supplementary MMRM analysis may also be performed using the primary target regions and primary reference region on the Tau PET population excluding subjects with change in smoking status during the study duration.

A forest plot of adjusted mean change from baseline at Week 78 may be provided for other secondary target regions (using primary reference region) and the primary target regions (using other exploratory reference regions).

MCP-MOD model

The multiple comparison procedure modelling (MCP-MOD) method (Appendix II) will be used to assess and model dose-response relationships (potential candidate models will include linear, linear-log, quadratic, maximum response [Emax], and logistic models as specified in Section16.10) while controlling for multiplicity for the primary regions (Braak 1 and 2, Braak 3 and 4, Braak 5 and 6) using the primary reference region (Cerebellum, superior section eroded).) The dose-response parameter of interest for MCP-MOD will be the LS means at Week78 for each dose group from the mixed-effects model. Pairwise comparisons of each BIIB092 dose group versus placebo will also be conducted. A line plot of final dose-response relationship will be provided.

5.6.3.4 Correlation between Tau PET vs. Cognitive results and other assessments

Pearson and Spearman correlation

Pearson and Spearman correlations between change from baseline tau PET primary summary measures at Week 78 and change from baseline CDR-SB at Week 78 may be conducted by treatment groups in subjects participating in tau PET substudy. Pearson and Spearman partial correlation adjusting for age, corresponding baseline tau PET measure and baseline CDR-SB will also be conducted by treatment groups at Week 78. The estimated correlation coefficients, p-values and the 95% confidence intervals will be provided. Same analyses will be used to analyze the correlations between change from baseline of tau PET at Week 52 and change from baseline of CDR-SB at Week 52. Given that reductions in tau burden may precede clinical effects (e.g., slowing of clinical decline), Pearson and Spearman correlations and partial correlations between change from baseline tau PET composite ROI at Week 52 and change from baseline CDR-SB at Week 78 will also be conducted. Similar models maybe conducted for MMSE, ADAS-Cog-13 total score, and ADCS-ADL total score.

ANCOVA model

An Analysis of Covariance (ANCOVA) approach might be used to explore the treatment effect with respect to change from baseline of tau PET at week 78 and change from baseline of CDR-SB at week 78. If a significant treatment benefit on CDR-SB is detected at week 78, three ANCOVA models will be included: Model 1 for change from baseline of tau PET at week 78, with factors of treatment, corresponding tau PET baseline value, age, baseline disease stage (MCI vs. mild AD), and baseline AD symptomatic medication use; Model 2 for change from baseline of CDR-SB at week 78, with factors of treatment, baseline CDR-SB, baseline tau PET value, age, baseline disease stage (MCI vs. mild AD), and baseline AD symptomatic medication use; Model 3 for change from baseline of CDR-SB at week 78, with factors in model 2, plus change from baseline of tau PET at week 78. The estimated treatment

effect, the associated 95% CI and p-value of the coefficient will be presented to show how much of the treatment effect with respect to CDR-SB can be explained by the treatment effect with respect to tau PET.

The same analyses might be explored for other clinical assessments: ADAS-Cog 13 total score, MMSE score and ADCS-ADL score. The same ANCOVA model might be used to analyze the treatment effect with respect to clinical endpoint and tau PET at different timepoint (both at week 52, or change from baseline of tau PET at week 52 with change from baseline of clinical assessment at week 78).

Correlation between tau PET and structural MRI

Pearson and Spearman correlations between change from baseline tau PET primary summary measures at Week 78 and change from baseline structural MRI at Week 78 may be conducted by treatment groups for key tau PET and structural MRI parameters. Pearson and Spearman partial correlation adjusting for age, corresponding baseline tau PET measure and baseline structural MRI measures may also be conducted by treatment groups at Week 78.

5.6.3.5 Subgroup analysis

Subgroup analysis may be conducted using the same MMRM model for the subgroup defined Section 3.4. Categorical covariates will be moved out of the model if they are used as a subgroup factor; for example, AD stage at baseline and AD symptomatic medication use at baseline.

5.7 Anti-drug Antibody Analysis

5.7.1 Analysis Methods for Anti-drug antibody Data

Anti-drug antibody (ADA) evaluable set will be used to analyze ADA data. For ADA endpoints, the following treatment groups will be presented in both tables and listings: placebo, BIIB092 125 mg/4wk, BIIB092 375 mg/12wk, BIIB092 600 mg/4wk, BIIB092 2000 mg/4wk, and BIIB092 total. Associations of ADA measures with PK and/or select AEs may be explored as needed.

The baseline value is defined as the last available value prior to first dose of placebo or BIIB092. For subjects with missing baseline assessment, the most conservative approach will be taken, and they will be considered negative for ADA at baseline.

Treatment emergent positive: Anti-BIIB092 antibody responses in antibody-positive subjects are defined as treatment emergent if a patient is:

- ADA-negative at baseline and ADA-positive post-baseline, or
- ADA-positive at baseline and had a greater than or equal to 4-fold increase in antibody titer post-baseline. If the titer value is not available for a positive baseline result, then the baseline titer value will be imputed as the minimum required dilution (MRD); or
- A positive post-baseline result where no titer is available, regardless of baseline value

Persistently positive: Anti-BIIB092 antibody responses in antibody-positive subjects are defined as persistent, if more than one positive evaluation occurs ≥16 weeks apart, regardless of the number of negative results between positive results, or if a positive evaluation occurs at the last time point with no further samples available.

Transiently positive: Anti-BIIB092 antibody responses in antibody-positive subjects are defined as transient, if a subject is treatment emergent positive at a single timepoint not including the last sampling timepoint, or treatment emergent with multiple positive anti-BIIB092 results <16 weeks apart, with the final timepoint being ADA negative.

Summary table with the number and percentage of all anti-BIIB092 positive antibody events by treatment group and visit will be displayed. In addition, a summary table of patients with treatment-emergent, persistent, and transient responses will be presented by treatment group. A listing of anti- BIIB092 antibody results will also be provided.

Visit windows for by visit summaries

For ADA data that are summarized by visit, assessment from all scheduled visits, EOT visit, and unscheduled visits will be mapped to an appropriate analysis visit using a windowing scheme (Appendix I).

5.8 Health Outcomes

The FAS will be used for the analysis of health outcomes data.

5.8.1 QoL-AD and ZBI

The actual and changes from baseline scores for health outcome measures in the ZBI total score, QoL-AD total score up to Week 68, will be summarized by treatment group.

A mixed model with repeated measures (MMRM) will be used to analyze changes from Baseline in ZBI and QoL-AD measures as described in Section 4.3.2 for ZBI and QOL-AD.

5.8.2 RUD-Lite

RUD-Lite is a brief measure of resource utilization developed for use in clinical trials for Alzheimer's disease. The RUD-Lite is completed by the caregiver and allows for cost calculation of caregiver burden. Components of the RUD-Lite include informal caregiver time, living accommodations and long-term care, use of respite, home nursing, and day care. Outpatient, hospital, and social services visits, as well as formal and informal caregiver time, are also captured. There are two different forms of the RUD-Lite: one for use at baseline and one for use at subsequent visits.

RUD-Lite consists of continuous and categorical items (see Table 5).

Table 6. RUD-Lite Items

Item	Parameter (Units)	Type
**Caregiver Items in Last 30 Days**		
1	Time per day or night spent asleep (hours)	Continuous

Item	Parameter (Units)	Type
2A	Time spent per day assisting subject with tasks (toilet visit, eating, etc.) (hours)	Continuous
2B	Days spent assisting subject with tasks (toilet visit, eating, etc.) (days)	Continuous
3A	Time spent per day assisting subject with tasks (shopping, food preparation, etc.) (hours)	Continuous
3B	Days spent assisting subject with tasks (shopping, food preparation, etc.) (days)	Continuous
4A	Time spent per day supervising subject (hours)	Continuous
4B	Days spent supervising subject (days)	Continuous
**Caregiver Work Status in Last 30 Days**		
2A	Missed any whole days of work	Continuous
2B	Missed any part of days of work	Continuous
**Patient Current Living Accommodation**		
1	Any permanent changes since last visit	Categorical (yes, no), post-baseline only
1[a], 2	Current living accommodation	Categorical (own home, ..., other)
2[a]	With whom subject lives	Categorical (alone, ..., not applicable)
3[a], 4	Temporary living accommodation	Categorical (own home, ..., other)
**Patient Health Care Resource Utilization in Last 30 Days (Baseline) or Since Last Visit (Post-Baseline)**		
1	How many times admitted to hospital	Continuous
2	Ward type, if admitted to hospital	Categorical (geriatric, ..., other)
3	How many times received care in hospital emergency room	Continuous
4	Visited health care professional (doctor, physiotherapist, etc.)	Categorical (yes, no)
4	Type of care, if visited health care professional	Categorical (general practitioner, ..., other)
5	Received nursing service	Categorical (yes, no)
5	Type of care, if received nursing service	Categorical (district nurse, ..., other)

[a] Baseline

For Caregiver Items in Last 30 Days, Items 1, 2A, 3A, and 4A, the total time spent (hours) in the last 30 days will be calculated as time per day (or night) x 30 days. The actual score for all the items in this section will be summarized by visit.

For caregiver work status, the percentage of caregivers working for pay will be summarized by treatment group at each time point. In addition, among those caregivers working for pay, the following summary will be provided by treatment group at each time point:

- The number of entire work days missed
- The percentage of caregivers of those who are working, who missed at least 1 entire day of work
- The number of partial work days missed
- The percentage of caregivers of those are working, who missed at least part of a day of work

For subject current living accommodation, the proportion of subjects who have a change in permanent living accommodation will be summarized by treatment group at each time point. Number of nights spent in the temporary living accommodation for subjects with a temporary change will also be summarized.

For subject health care resource utilization, the following summary will be provided by treatment group at each time point.

- Percentage of subjects admitted to hospital; number of times and nights admitted to hospital for subjects admitted to hospital
- Percentage of subjects received care in hospital emergency room; number of times of emergency room care for subjects received care in hospital emergency room
- Percentage of subjects visited health care professional (doctor, physiotherapist, etc.); number of times visited for subjects visited health care professional

Percentage of subjects received nursing care; number of visits and hours of nursing care for subjects received nursing care.

6 Changes from Protocol-Specified Analyses

In order to follow Biogen standard, the below population names have been changed. However, the definition remains the same.

**Protocol specified name**	**SAP name**
Intent-to-treat population	Full Analysis Set
Per-protocol population	Per-Protocol Analysis Set
Safety population	Safety Analysis Set
Safety MRI population	Safety MRI Evaluable Set
Serum PK analysis population	Serum PK Evaluable Set
CSF PK analysis population	CSF PK Evaluable Set
Serum PD population	Serum PD Evaluable Set
CSF PD population	CSF PD Evaluable Set
Tau PET population	Tau PET Evaluable Set

Structural MRI population	Structural MRI Evaluable Set
Anti-drug antibody population	Anti-Drug Antibody Evaluable Set

6.1 MMRM model

The MMRM model specified in SAP section includes "baseline MMSE" as one more fixed effect than protocol section 16.4.2.1 and 16.4.2.2.

6.2 MCP-MOD model

Besides six planed dose-response relationships as illustrated in Figure2, linear dose-response relationship was added for both models to detect the change from baseline in CDR-SB and tau PET. The approximately power remains at 80%.

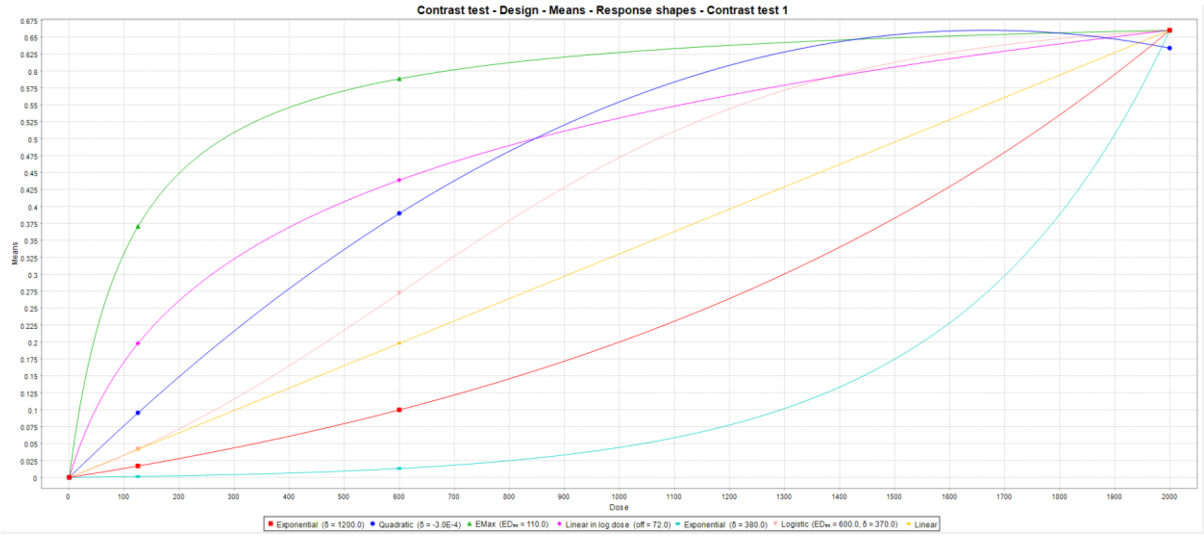

7 Appendix I: Visit Window Mapping

For data that are summarized by visit and longitudinal analysis, assessment from all scheduled visits including EOT visit and EOS visit, and all unscheduled visits will be mapped to an appropriate analysis visit using a windowing scheme. Analysis visit windows are defined in [Table 7 - Table 15] for different endpoints. To define analysis visit window, the target visit day is calculated as (week number*7+1). The lower bound of visit window is calculated as target day−(target day−target day of previous visit)/2+1, except for the first post-baseline visit window whose lower bound is set as Day 2; the upper bound of visit window is calculated as target day+(target day of next visit−target day)/2.

If there are 2 or more assessments from visits, including EOT and EOS visits, mapped to the same analysis visit for a subject, the assessment that is closest to the target visit day will be used for analysis. If there are 2 or more assessments from visits, including EOT and EOS visits, mapped to the same analysis visit with the same distance from the target visit day, then select the later one(s) for the analysis. If there are 2 or more assessments from visits, including EOT and EOS visits, mapped to the same analysis visit and on the same day, use the average value for quantitative parameters and the worst value for qualitative parameters for analysis.

Table 7. Visit Windows for Efficacy Endpoints

Efficacy Endpoint	Analysis visit	Target visit day	Analysis visit window
CDR, NPI-10, ADAS-Cog13, FAQ, and ADCS-ADL	Baseline	1	Most recent non-missing pre-dose value
	Week 24	169	[2, 267]
	Week 52	365	[268, 456]
	Week 78	547	[457, *]
ISLT, DKEFS, DSST, Trails A,	Baseline	1	Most recent non-missing pre-dose value
	Week 12	85	[2, 141]
	Week 28	197	[142, 239]
	Week 40	281	[240, 337]
	Week 56	393	[338, 435]
	Week 68	477	[436, 512]
	Week 78	547	[513, *]
eCog	Baseline	1	Most recent non-missing pre-dose value
	Week 28	197	[2, 295]
	Week 56	393	[296, 470]
	Week 78	547	[471, *]
MMSE	Baseline	1	Most recent non-missing pre-dose value
	Week 12	85	[2, 127]

CONFIDENTIAL
The information contained herein may not be used, disclosed, or published without the written consent of Biogen MA Inc.

Efficacy Endpoint	Analysis visit	Target visit day	Analysis visit window
	Week 24	169	[128, 225]
	Week 40	281	[226, 323]
	Week 52	365	[324, 421]
	Week 68	477	[422, 512]
	Week 78	547	[513, *]
	* Up to the end day of the placebo-controlled period.		

Table 8. Visit Windows for Laboratory by Visit Summaries

Analysis visit	Target visit day	Analysis visit window
Baseline	1	Most recent non-missing pre-dose value
Week 12	85	[2, 127]
Week 24	169	[128, 253]
Week 48	337	[254, 421]
Week 72	505	[422, 526]
Week 78	547	[527, 589]<br>If subjects who enter LTE, visit window is: [527, the end day of the placebo-controlled period]
Week 90	631	[590, the end day of the placebo-controlled period]

Table 9. Visit Windows for ECG by Visit Summaries

Analysis visit	Target visit day	Analysis visit window
Baseline	1	Most recent non-missing pre-dose value
D1 Post dose	1	Any day 1 post dose infusion
Week 12	85	[2, 127]
Week 24	169	[128, 253]
Week 48	337	[254, 421]
Week 72	505	[422, 526]
Week 78	547	[527, 589]<br>If subjects who enter LTE, visit window is: [527, the end day of the placebo-controlled period]
Week 90	631	[590, the end day of the placebo-controlled period]

Table 10. Visit Windows for Vital Sign by Visit Summaries

Analysis visit	Target visit day	Analysis visit window
Baseline	1	Most recent non-missing pre-dose value
D1 Post dose	1	Day 1 post dose infusion (if more than one Day 1 post dose results available, the average of the available results will be used)

Analysis visit	Target visit day	Analysis visit window
Week 4 – Pre dose	29	[2, 43] (Pre-dose assessment)
Week 4 – Post dose	29	[2, 43] (Post dose infusion)
Week 8 – Pre dose	57	[44, 71] (Pre-dose assessment)
Week 8 – Post dose	57	[44, 71] (Post dose infusion)
Week 12 – Pre dose	85	[72, 99] (Pre-dose assessment)
Week12 – Post dose	85	[72, 99] (Post dose infusion)
Week 16	113	[100, 127]
Week 20	141	[128, 155]
Week 24	169	[156, 183]
Week 28	197	[184, 211]
Week 32	225	[212, 239]
Week 36	253	[240, 267]
Week 40	281	[268, 295]
Week 44	309	[296, 323]
Week 48	337	[324, 351]
Week 52	365	[352, 379]
Week 56	393	[380, 407]
Week 60	421	[408, 435]
Week 64	449	[436, 463]
Week 68	477	[464, 491]
Week 72	505	[492, 519]
Week 76	533	[520, 540]
Week 78	547	[541, 589]<br>If subjects who enter LTE, visit window is: [541, the end day of the placebo-controlled period]
Week 90	631	[590, the end day of the placebo-controlled period]

Table 11. Visit Windows for ADA by Visit Summarized

Analysis visit	Target visit day	Analysis visit window
Baseline	1	See baseline definition in Section 5.7
Week 4	29	[2, 99]
Week 24	169	[100, 253]
Week 48	337	[254, 435]
Week 76	533	[436, 582]<br>If subjects who enter LTE, visit window is: [436, the end day of the placebo-controlled period]
Week 90	631	[583, the end day of the placebo-controlled period]

Table 12. Visit Windows for tau PET data

Analysis visit	Target visit day	Analysis visit window
Baseline	1	Most recent non-missing pre-dose value
Week 52	365	[120, 456]
Week 78	547	[457, *]
* The upper bound of Week 78, for subjects not enrolled into LTE, is the last day in study for PC period; for subjects enrolled into LTE, their Week 78 upper bound is less than the day of third infusion in LTE (Due to Covid-19, subjects could complete Week 78 tau PET after week 78 but before third infusion in LTE.)		

Table 13. Visit Windows for structural MRI data

Analysis visit	Target visit day	Analysis visit window
Baseline	1	Most recent non-missing pre-dose value
Week 28	197	[90, 281]
Week 52	365	[282, 456]
Week 78	547	[457, the end day of the placebo-controlled period]

Table 14. Visit Windows for C-SSRS data

Analysis visit	Target visit day	Analysis visit window
Baseline	1	Most recent non-missing pre-dose value
Week 24	169	[2, 267]
Week 52	365	[268, 456]
Week 78	547	[457, the end day of the placebo-controlled period]

Table 15. Visit Windows for Health Outcome (RUD-Lite, ZBI, and Qol-AD) data

Analysis visit	Target visit day	Analysis visit window
Baseline	1	Most recent non-missing pre-dose value
Week 20	141	[2, 239]
Week 48	337	[240, 407]
Week 68	477	[408, the end day of the placebo-controlled period]

Table 16. Visit Windows for CSF PD biomarker data

Analysis visit	Target visit day	Analysis visit window
Baseline	1	Most recent non-missing pre-dose value
Week 12	85	[2, 211]
Week 48	337	[212, 456]
Week 76	533	[457, the end day of the placebo-controlled period]

Table 17. Visit Windows for Serum PD biomarker data

Analysis visit	Target visit day	Analysis visit window
Baseline	1	Most recent non-missing pre-dose value

Analysis visit	Target visit day	Analysis visit window
Week 16	113	[2, 225]
Week 48	337	[226, 379]
Week 60	421	[380, 477]
Week 76	533	[478, the end day of the placebo-controlled period]

8 Appendix II: Description of MCP-MOD method

MCP-MOD method works in the following steps.

Step 1: Set of candidate models

Candidate models include linear, exponential (δ = 1200), quadratic (δ=-0.0003), Emax (ED50=110), linear in log dose (off = 72), exponential (δ=380) and logistic (ED50 = 600, δ=370). The response shapes are displayed in 0.

Step 2: Optimal model contrast

The LS means at Week 78 and the covariance matrix of the LS means will be estimated from the mixed model and used to determine the optimal contrasts. The coefficients of the contrasts are pre-specified during the design stage once the candidate models are selected in Step 1.

Step 3: Testing for dose response signal

A multiple contrast test will be used to test the overall dose response signal and to identify all contrasts that have adjusted p-values<0.1 for one sided. As a result, the significance of the dose response signal was established and all models with a significant contrast will be considered in the next step.

Step 4: Model selection

AIC criterion will be used to select the best model for Step 5.

Step 5: Dose estimation

The model selected in Step 4 will be used to fit the data. The fitted model and corresponding confidence interval will be displayed graphically. The minimum effective dose giving the 40% reduction or the clinically relevant improvement 25% reduction over placebo will be estimated along with the 90% and 95% confidence interval. This step may be explored if there is a significant dose response.

9 Appendix III: Questionnaire

9.1 Clinical Dementia Rating Scale (CDR)

The CDR is derived from a semi-structured interview with the patient and an appropriate informant and rates impairment in each of six cognitive categories (Memory, Orientation, Judgment and Problem Solving, Community Affairs, Home and Hobbies, and Personal Care) on a five-point scale in which none = 0, questionable = 0.5, mild = 1, moderate = 2, and severe = 3. (Note: Personal Care has no questionable impairment level.) From the six individual category ratings, or "box scores", the global CDR is established by clinical scoring rules where CDR 0 = no dementia and CDR 0.5, 1, 2, or 3 indicates questionable, mild, moderate, or severe dementia. The usefulness of the CDR may result from several factors: (1) it is clinically based (i.e., independent of psychometric test scores); (2) the six categories used for rating dementia severity are directly linked to validated clinical diagnostic criteria for AD; (3) it has high inter-rater reliability for physicians and non-physicians; and (4) and expanded and more quantitative version of the scale can be achieved by summing the ratings in each of the six categories to provide the Sum of Bases. [Morris 1993]

9.2 Mini-Mental State Examination (MMSE)

The Mini-Mental State Examination (MMSE) includes eleven questions, requires only 5-10 min to administer. It is "mini" because it concentrates only on the cognitive aspects of mental functions, and excluded questions concerning mood, abnormal mental experiences and the form of thinking. But within the cognitive realm it is thorough.

The MMS is divided into two sections, the first of which requires vocal responses only and covers orientation, memory, and attention; the maximum score is 21. The second part tests ability to name, follow verbal and written commands, write a sentence spontaneously, and copy a complex polygon similar to a Bender-Gestalt Figure; the maximum score is nine. Because of the reading and writing involved in Part II, patients with severely impaired vision may have some extra difficulty that can usually be eased by large writing and allowed for in the scoring. Maximum total score is 30. [Folstein MF 1975]

9.3 International Shopping List Test Immediate Recall (ISLT)

The International Shopping List Test (ISLT) is a 12-word, 3-trial word learning test with established construct and criterion validity, and high reliability for the detection of memory impairment in AD

The design of the ISLT allows it to be used for the assessment of memory in individuals from different languages, cultures or geographic regions without the necessity of the complex translations or cultural adaptions that must be applied to other verbal memory tests, such as the Free and Cued Selective Reminding Test (FCSRT) or Wechsler Memory Scale Logical Memory Test (LM), which were developed and validated in well-educated and middle-class settings

The ISLT has been used extensively to measure the magnitude and nature of memory impairment in experimental and clinical studies; however, there is less information about the

utility of the ISLT to screen individuals' memory for entry into clinical trials in prodromal AD [Paul Maruff 2017].

9.4 Delis-Kaplan Executive Function System (DKEFS)

The Delis-Kaplan Executive Function System (D-KEFS; Delis, Kaplan, & Kramer, 2001) provides a standardized assessment of higher-level cognitive functions, called executive functions, in children, adolescents, and adults between the ages of 8 and 89. The D-KEFS is composed of the following nine stand-alone tests that can be individually or group administered: Trail Making Test; Verbal Fluency Test; Design Fluency Test; Color-Word Interference Test; Sorting Test; Twenty Questions Test; Word Context Test; Tower Test; and Proverb Test. Most of these D-KEFS tests use a game-like format without employing right/wrong feedback procedures; this is intended to reduce unproductive discouragements and frustrations caused by repeated negative feedback during testing.

The D-KEFS Verbal Fluency Test (VF) is comprised of three testing conditions: Letter Fluency, Category Fluency, and Category Switching. The VF measures multiple aspects of verbal behavioral productivity and cognitive flexibility. It evaluates effectiveness of novel and semantic search strategies and assesses flexibility in the implementation of semantic search strategies. The process approach enables further evaluation of self-monitoring of information search, as well as difficulties related to initiation and sustaining effort. There are three conditions in the VF in which the examinee must say as many words as they can by letter, category, and category switching prompts.

1. Letter Fluency: The examinee says words beginning with a specified letter as quickly as possible;

2. Category Fluency: The examinee is asked to say words belonging to a designated semantic category; and

3. Category Switching: The examinee must alternate between saying words from two different; semantic categories.

In this study, only Letter Fluency and Category Fluency tests are used, but NOT Category Switching. The Letter Fluency test measures the examinee's ability to generate words fluently in an effortful, phonemic format. The Category Fluency test measures the examinee's ability to generate words fluently from overlearned concepts.

9.5 Digit Symbol Substitution Test (DSST)

The Digit Symbol Substitution Test (DSST) was initially developed as an experimental tool over a century ago by researchers seeking to understand human associative learning. The DSST is a paper-and-pencil cognitive test presented on a single sheet of paper that requires a subject to match symbols to numbers according to a key located on the top of the page. The subject copies the symbol into spaces below a row of numbers. The number of correct symbols within the allowed time, usually 90 to 120 seconds, constitutes the score.

This study is using the most recent version of Wechsler Adult Intelligence Scale (WAIS), the fourth edition (WAIS IV). The duration of DSST in this version is 120 seconds. The DSST measures a range of cognitive operations. Good performance on the DSST requires intact motor speed, attention, and visuo perceptual functions, including scanning and the ability to write or

draw (ie, basic manual dexterity). That the DSST is sensitive to age effects is well known. The DSST has been a useful tool to demonstrate age differences in the effects of drugs on cognition, for example, owing to reduced clearance resulting in higher plasma concentrations in older than in younger subjects. [Judith Jaeger, 2018]

9.6 Trail Making Test, Part A (Trail A)

Trail Making Test consist of 25 circles distributed over a sheet of paper. In Part A, the circles are numbered 1 – 25, and the patient should draw lines to connect the numbers in ascending order, and the participants were asked to connect the numbers in order as quickly as possible. The total completion time will be evaluated as final result. The maximal allowed time is <5mins in our study. Longitudinal studies have shown that the Trail Making Test is capable of forecasting clinical and functional changes in patients with Alzheimer's disease (Chen et al., 2001). Moreover, in older adults, Trail Making Test performance has been related to physical decline and higher risk of mortality [Vazzana et al., 2010, Jordi Llinàs-Reglà, 2017].

9.7 Everyday Cognition (eCog)

The Everyday Cognition (eCog) scales were developed in response to some of the limitations of existing instruments. One goal in developing the eCog was to measure relatively mild functional changes that may predate loss of independence in major activities of daily living. A second aim in developing the eCog was to assess functional abilities that are clearly linked to specific cognitive abilities, in other words, the everyday correlates of specific neuropsychological impairments.

The original version of the eCog is an informant-based measure of cognitively-relevant everyday abilities comprised of 39 items, covering six cognitively-relevant domains: Everyday Memory, Everyday Language, Everyday Visuospatial Abilities, and Everyday Planning, Everyday Organization, and Everyday Divided Attention. For each item, informants compare the participant's current level of everyday functioning with how he or she functioned 10 years earlier. In this way, individuals serve as their own control. Ratings are made on a four-point scale: 1 = better or no change compared to 10 years earlier, 2 = questionable/occasionally worse, 3 = consistently a little worse, 4 = consistently much worse. The original eCog was developed through a rigorous process that included initial pilot testing of a larger potential pool of items. A goal of that initial pilot testing was to identify and discard items with obvious poor psychometric properties. For example, items were not retained if they were associated with a high percentage of "I don't know" responses – indicating the item did not readily apply to many individuals or was not frequently observed by an informant. However, few functional abilities will universally apply to all individuals so an 'I don't know' response option was retained. The original version has been shown to have excellent psychometric properties including good test-retest reliability (r = 0.82, p<.001) as well as evidence of various aspects of validity including content, construction, convergent and divergent, and external validity [Sarah Tomaszewski Farias, 2011].

9.8 Alzheimer's disease Cooperative Study – Activities of Daily Living (ADCS-ADL)

The ADCS-ADL was designed to assess patients' ability to complete activities relevant to elderly individuals. It is a subjective assessment, with the patient rated by an informant, based on their performance over the previous 4 weeks.

The ADCS-ADL has 23-item which focuses on complex items, such as reading, pastime activities, and household chores, and is appropriate for the assessment of mild to moderate AD. Each item has descriptions of performance levels and corresponding scores; the informant is asked to choose the most accurate description of the patient's performance during the past 4 weeks. The ADCS-ADL23 Total score ranges from 0 (worst) to 78 (best). [Galasko et al. 1997; Robert et al. 2010]. ADCS-ADL basic (ADCS-bADL) total score includes questions 1 (eating), 2 (walking), 3 (bowel and bladder function at the toilet), 4 (bathing), 5 (grooming), and 6b (getting dressed). ADCS-ADL instrumental (ADCS-iADL) total score includes 6a (selecting his/her first set of clothes), and the remaining questions from 7 -23 in ADCS-ADL.

9.9 Functional Activities Questionnaire (FAQ)

The Functional Activities Questionnaire (FAQ) is not self-administered but is completed by a lay informant such as the spouse, a relative, or a close friend. There are 10 questions included. For each activity, four levels ranging from dependence (scored 3) to independence (scored 0) are specified. For activities not normally undertaken by the person, the informant must specify whether the person would be unable to undertake the task if required (scored 1) or could do so if required (0). The total score is the sum of individual item scores; higher scores reflect greater dependency. [Robert I. Pfeffer, 1982]

9.10 Alzheimer's disease Assessment Scale –Cognitive (13 item) (ADAS-Cog 13)

The modified ADAS-Cog 13-item scale (Mohs et al. 1997) includes all original ADAS-Cog items with the addition of a number cancellation task and a delayed free recall task, for a total of 85 points. As in the parent instrument, higher scores indicated greater severity. According to Mohs and colleagues, the purpose of these additional items was to increase the number of cognitive domains and range of symptom severity without a substantial increase in the time required for administration. [Jeannine Skinner, 2012]

9.11 Neuropsychiatric Inventory – 10 (NPI-10)

The Neuropsychiatric Inventory (NPI) was developed by Cummings et al. (1994) to assess dementia-related behavioral symptoms which they felt other measures did not sufficiently address. The NPI originally examined 10 sub-domains of behavioral functioning: delusions, hallucinations, agitation/aggression, dysphoria, anxiety, euphoria, apathy, disinhibition, irritability/lability, and aberrant motor activity. The NPI is administrated to caregivers of dementia patients. A screening question is asked about each sub-domain. If the responses to these questions indicate that the patient has problems with a particular sub-domain of behavior, the caregiver is only then asked all the questions about that domain, rating the frequency of the symptoms on a 4-point scale, their severity on a 3-point scale, and the distress the symptom

causes them on a 5-point scale. The score of each item is then calculated by multiplying severity by frequency, thus obtaining a score ranging between 0 and 12. The total NPI score is finally obtained by adding all the single item scores (thus, ranging from 0 to 120). Higher scores indicate greater psychopathology.

9.12 Zarit Burden Interview (ZBI)

The Zarit Burden Interview (ZBI), which provides a comprehensive assessment of both objective and subjective burden, is one of the most commonly used burden measures and has been validated in many culturally or ethnically different populations.

The Zarit Burden Interview (ZBI) is a 22-item instrument for measuring the caregiver's perceived burden of providing family care. The 22 items are assessed on a 5-point Likert scale, ranging from 0 = 'never' to 4 = 'nearly always'. Item scores are added up to give a total score ranging from 0 to 88, with higher scores indicating greater burden. The questions focus on major areas such as caregiver's health, psychological well-being, finances, social life and the relationship between the caregiver and the patient [Boon Kheng Seng, 2010].

9.13 Quality of Life for Alzheimer's disease (Qol-AD)

QOL is an important consideration in AD because of the devastating impact of this currently incurable disease on patients and caregivers. From the patient's perspective, QOL measures may assist understanding of the magnitude of the impact of treatment intervention, whereas, from a payer perspective, QOL measures can provide a common metric of comparison across disease states. The QOL-AD has been shown to have excellent internal consistency reliability for both patient and caregiver reports (a 5 0.84 and 0.86, respectively) at all levels of cognitive functioning and good validity as indicated by correlations with measures of depression, day-to-day functioning, and pleasant events frequency. Thorgrimsen and colleagues [2003] reported the QOL-AD to have good content validity, construct validity, interrater reliability (all Cohen's kappa values .0.70), test-retest reliability, and internal consistency (Cronbach a coefficient of 0.82). QOL is measured using the 13-item QOL-AD scale (total score range 13–52; higher scores indicate better QOL). The QOL-AD scale uses a scale of 1–4 (poor, fair, good, or excellent) to rate a variety of life domains, including the patient's physical health, mood, relationships, activities, and ability to complete tasks [Kristin Kahle-Wrobleski, 2017].

9.14 Resource Utilization in Dementia-Lite Version (RUD-Lite)

The RUD was designed for use in clinical trials to assess the level of resource usage among patients with dementia. As the full RUD tool is extensive, a shortened version was created – the RUD Lite.

The RUD Lite consists of two sections: one about the caregiver (including questions about the caregiver him- or herself, and time spent caring for the patient), and one about the patient (including questions about the patient's living arrangements and their healthcare resource usage).

The RUD Lite is a questionnaire rather than an assessment scale, and, as such, it is not 'scored'. Instead, individual questions of the patient component provide resource use information, such as the number of hospitalizations, and the number of nights spent in hospital.

Regarding the caregiver component, the number of hours per day that the caregiver cares for the patient is recorded, based on a typical care day during the past month. This is broken down into time spent assisting with personal ADLs (such as bathing and dressing), time spent assisting with instrumental ADLs (more complex activities such as shopping and housekeeping), and time spent supervising the patient. [Wimo & Winblad. Brain Aging 2003;3(1):48–59]

10 Appendix IV: Adverse event of special interest custom search criteria

Anti-drug antibody is an AESI in the BIIB092 clinical development program. Adverse events representing potential immune/hypersensitivity reactions will be identified using the following customized MedDRA (23.1) search criteria:

Immunogenicity is an AESI in the BIIB092 clinical development program. Adverse events representing potential immune/hypersensitivity reactions will be identified using the following customized MedDRA (23.1) search criteria:

- Anaphylactic reaction SMQ narrow and broad
- Angioedema SMQ narrow and broad
- Severe cutaneous adverse reactions SMQ narrow and broad
- Eosinophilia (PT terms: Eosinophilia; Eosinophil count increased; Allergic eosinophilia; Pulmonary eosinophilia)
- HLT Allergic conditions NEC
- Miscellaneous terms
    - Antibody test
    - Antibody test abnormal
    - Antibody test positive
    - Cytokine release syndrome
    - Documented hypersensitivity to administered product
    - Drug specific antibody present
    - Infusion related reaction
    - Infusion site reaction
    - Neutralising antibodies
    - Neutralising antibodies positive
    - Non-neutralising antibodies positive
    - Respiratory dyskinesia
    - Rheumatoid arthritis
    - Systemic lupus erythematosus
    - Vasculitis

11 Appendix V: Infusion reactions custom search

• Infusion site reaction
• Infusion site rash
• Infusion site dermatitis
• Infusion site hypersensitivity
• Infusion site photosensitivity reaction
• Infusion site urticaria
• Infusion site eczema
• Infusion site vasculitis
• Infusion site recall reaction
• Infusion related reaction
• Administration site rash
• Administration site dermatitis
• Administration site eczema
• Administration site hypersensitivity
• Administration site urticaria
• Administration site photosensitivity reaction
• Administration site recall reaction
• Administration site vasculitis
• Administration related reaction
• Injection site dermatitis
• Injection site hypersensitivity
• Injection site rash
• Injection site urticaria
• Injection site photosensitivity reaction
• Injection site eczema
• injection site recall reaction
• Injection site vasculitis
• Injection related reaction
• Immediate post-injection reaction
• allergic reaction to excipient
• reaction to excipient
• cytokine storm
• cytokine release syndrome
• immune-mediated adverse reaction

12 References

Folstein MF, Folstein SE, McHugh PR: "Mini-mental state: A practical method for grading the cognitive state of patients for the clinician." J Psychiatr Res 1975;12:189-198.

Paul Maruff, PhD, Adrian Schembri. Utility of the International Shopping List Test for detection of memory impairment associated with prodromal and early Alzheimer's disease in clinical trials

Susan Homack , Donghyung Lee & Cynthia A. Riccio: Test Review: Delis-Kaplan Executive Function System

Judith Jaeger, PhD, MPA: Digit Symbol Substitution Test the Case for Sensitivity Over Specificity in Neuropsychological Testing

Caterina Rosano: Digit Symbol Substitution test and future clinical and subclinical disorders of cognition, mobility and mood in older adult

Jordi Llinàs-Reglà: The Trail Making Test: Association With Other Neuropsychological Measures and Normative Values for Adults Aged 55 Years and Older From a Spanish-Speaking Population-Based Sample

Sarah Tomaszewski Farias: The Measurement of Everyday Cognition (ECog): Development and validation of a short form

Ian McDowell, Measuring Health: A Guide to Rating Scales and Questionnaires, Third Edition. Oxford University Press

Jeannine Skinner: The Alzheimer's Disease Assessment Scale-Cognitive-Plus (ADAS-Cog-Plus): an expansion of the ADAS-Cog to improve responsiveness in MCI

Richard C. MohsDeborah MarinCynthia R. GreenKenneth L. Davis: The Alzheimer's Disease Assessment Scale: Modifications That Can Enhance its Use in Future Clinical Trials. Springer Link

Boon Kheng Seng: Validity and Reliability of the Zarit Burden Interview in Assessing Caregiving Burden

Carpenter JR, Roger JH, Kenward MG. Analysis of longitudinal trials with protocol deviation: a framework for relevant, accessible assumptions, and inference via multiple imputation. J Biopharm Stat. 2013;23(6):1352-71.

Galasko D, Bennett D, Sano M, Ernesto C, R Thomas R, Grundman M, S Ferris S. An inventory to assess activities of daily living for clinical trials in Alzheimer's disease, The Alzheimer's Disease Cooperative Study. Alzheimer Dis Assoc Disord 1997; 11(Suppl 2): S33–S39;

DocuSign Envelope ID: 7A1F456A-919D-4916-B66C-75B177AF3423

ICH E9 (R1): Addendum to statistical principles for clinical trials on choosing appropriate estimands and defining sensitivity analyses in clinical trials. Endorsed by the ICH Steering Committee. 2014.

ICH E9 (R1): Addendum on Estimands and Sensitivity Analysis in Clinical Trials to the Guideline on Statistical Principles for Clinical Trials. European Medicines Agency. 2017.

Jinping Wang, Veronika Logovinsky, Suzanne B Hendrix. ADCOMS: a composite clinical outcome for prodromal Alzheimer's disease trials. J Neurol Neurosurg Psychiatry 2016;87:993–999

Judith Jaeger, PhD, Clint Hagen, MS, Henrik Loft, PhD. Cognitive Endpoints for Early Alzheimer's Disease Trials: Development of the Early AD/ MCI Alzheimer's Cognitive Composite (EMACC). ISCTM 2018

Li, K. H. (1988), "Imputation Using Markov Chains," *Journal of Statistical Computation and Simulation*, 30, 57–79.

Little, R.J.A. (1993). Pattern‐mixture models for multivariate incomplete data. *Journal of the American Statistical Association, 88* , 125-134.

Little, R.J.A. (1994). A class of pattern‐mixture models for normal missing data. *Biometrika, 81* , 471-483.

Morris JC. The Clinical Dementia Rating (CDR): current version and scoring rules. Neurology. 1993;43(11):2412–2414.

Robert P, Ferris S, Gauthier S, Ihl R, Winblad B, Tennigkeit F. Review of Alzheimer's disease scales: is there a need for a new multi-domain scale for therapy evaluation in medical practice? Alzheimers Res Ther 2010; 2(4):24

Rubin, D. B. (1976) "Inference and missing data." Biometrika, 63(3), 581-592.

Rubin D.B. Multiple Imputation for Nonresponse in Surveys. John Wiley & Sons. 1987.

Schafer J.L. Analysis of Incomplete Multivariate Data. Chapman and Hall, New York. 1997.

Tractenberg RE, Weiner MF, Cummings JL, Patterson MB, Thal LJ. Independence of changes in behavior from cognition and function in community-dwelling persons with Alzheimer's disease: a factor analytic approach. J Neuropsychiatry Clin Neurosci. 2005;17(1):51–60.

van Ginkel, J. R., Sijtsma, K., van der Ark, L. A., & Vermunt, J. K. (2010). Incidence of missing item scores in personality measurement, and simple item-score imputation. Methodology: European Journal of Research Methods for the Behavioral and Social Sciences, 6(1), 17-30.

CONFIDENTIAL
The information contained herein may not be used, disclosed, or published without the written consent of Biogen MA Inc.

Wessels, A.M., Andersen, S.W., Dowsett, S.A., and Siemers, E.R. (2018), "The integrated Alzheimer's disease rating scale (iADRS) findings from the expedition3 trial", Journal of Prevention of Alzhermer's Disease, 2018(5), 134-136.

Wessels, A.M., Siemers, E.R., Yu, P., Andersen, S.W., Holdridge K.C., Sims, J.R., Sundell, K., Stern Y., Rentz, D.M., Dubois, B., Jones, R.W., Cummings, J., and Aisen, P.S. (2015), "A combined measure of cognition and function for clinical trials: the integrated Alzhermer's disease rating scale (iADRS)", Journal of Prevention of Alzheimer's Disease.

CONFIDENTIAL
The information contained herein may not be used, disclosed, or published without the written consent of Biogen MA Inc.

Certificate Of Completion

Envelope Id: 7A1F456A919D4916B66C75B177AF3423
Subject: Please DocuSign: 251AD201 SAP V2.0_final.docx
Vendor Name:
Source Envelope:
Document Pages: 70
Certificate Pages: 2
AutoNav: Enabled
EnvelopeId Stamping: Enabled
Time Zone: (UTC-05:00) Eastern Time (US & Canada)

Signatures: 4
Initials: 0

Status: Completed

Envelope Originator:
[redacted]
225 Binney St
Cambridge, MA 02142
[redacted]

Record Tracking

Status: Original<br>31 March 2021 | 13:11	Holder: [redacted]	Location: DocuSign

Signer Events	Signature	Timestamp
[redacted]<br>Biogen<br>Security Level: Email, Account Authentication (None)<br><br>**Electronic Record and Signature Disclosure:**<br>Not Offered via DocuSign	DocuSigned by:<br>[redacted]<br>Signature Adoption: Pre-selected Style<br>Using IP Address: [redacted]	Sent: 31 March 2021 | 14:07<br>Viewed: 31 March 2021 | 14:07<br>Signed: 31 March 2021 | 14:08
[redacted]<br>Biogen<br>Security Level: Email, Account Authentication (None)<br><br>**Electronic Record and Signature Disclosure:**<br>Not Offered via DocuSign	DocuSigned by:<br>[redacted]<br>Signature Adoption: Pre-selected Style<br>Using IP Address: [redacted]	Sent: 31 March 2021 | 14:08<br>Viewed: 31 March 2021 | 16:15<br>Signed: 31 March 2021 | 16:15
[redacted]<br>Biogen<br>Security Level: Email, Account Authentication (None)<br><br>**Electronic Record and Signature Disclosure:**<br>Not Offered via DocuSign	DocuSigned by:<br>[redacted]<br>Signature Adoption: Pre-selected Style<br>Using IP Address: [redacted]	Sent: 31 March 2021 | 16:15<br>Viewed: 31 March 2021 | 16:32<br>Signed: 31 March 2021 | 16:32
[redacted]<br>Biogen<br>Security Level: Email, Account Authentication (None)<br><br>**Electronic Record and Signature Disclosure:**<br>Not Offered via DocuSign	DocuSigned by:<br>[redacted]<br>Signature Adoption: Pre-selected Style<br>Using IP Address: [redacted]	Sent: 31 March 2021 | 16:32<br>Viewed: 31 March 2021 | 17:10<br>Signed: 31 March 2021 | 17:11

In Person Signer Events	Signature	Timestamp

Editor Delivery Events	Status	Timestamp

Agent Delivery Events	Status	Timestamp

Intermediary Delivery Events	Status	Timestamp

Certified Delivery Events	Status	Timestamp

Carbon Copy Events	Status	Timestamp

Witness Events	Signature	Timestamp

Notary Events	Signature	Timestamp

Envelope Summary Events	Status	Timestamps
Envelope Sent	Hashed/Encrypted	31 March 2021 | 14:07
Certified Delivered	Security Checked	31 March 2021 | 17:10
Signing Complete	Security Checked	31 March 2021 | 17:11
Completed	Security Checked	31 March 2021 | 17:11

Payment Events	Status	Timestamps

TANGO

251AD201/NCT03352557

Statistical Analysis Plan

Long-Term Extension Period

STATISTICAL ANALYSIS PLAN
Long-Term Extension Period

Product Studied: Gosuranemab
Protocol Number: 251AD201

Randomized, Double-Blind, Placebo-Controlled, Parallel-Group Study to Assess the Safety, Tolerability, and Efficacy of BIIB092 in Subjects with Mild Cognitive Impairment due to Alzheimer's Disease or with Mild Alzheimer's Disease

Protocol Version: Version 4.0
Date of Protocol: 13 January 2020

Date of Statistical Analysis Plan: 16 Sep 2021, Version 1.0

Written By: [redacted]

Approved By:

[redacted], PhD — Date

[redacted], PhD — Date

[redacted] MD — Date

[redacted] MD — Date

CONFIDENTIAL
The information contained herein may not be used, disclosed, or published without the written consent of Biogen MA Inc.

Table of Contents

LIST OF ABBREVIATIONS 5

1 INTRODUCTION 7

2 DESCRIPTION OF LONG-TERM EXTENSION (LTE) OBJECTIVES AND ENDPOINTS 7

2.1 PRIMARY LTE OBJECTIVE AND ENDPOINT 7
2.2 EXPLORATORY LTE OBJECTIVES AND ENDPOINTS 7
2.3 STUDY DESIGN 8
2.4 STUDY SCHEMATIC 10

3 DEFINITIONS 12

3.1 ANALYSIS SETS 12
3.2 ANALYSES PERIOD 13
3.3 STUDY TREATMENT 13
3.4 DATES AND POINTS OF REFERENCE 14
3.5 KEY DERIVED VARIABLES 15
3.6 STRATIFICATION FACTORS AND SUBGROUP VARIABLES 18

4 LIST OF PLANNED STUDY ANALYSES 19

4.1 LTE INTERIM ANALYSES AT PC PERIOD DATABASE LOCK 19
4.2 FINAL ANALYSIS 19

5 STATISTICAL METHODS FOR PLANNED ANALYSES 20

5.1 GENERAL CONSIDERATIONS 20
5.2 STUDY SUBJECTS 20
5.2.1 Accounting of Subject 20
5.2.2 Demographics and Baseline Characteristics 20
5.2.2.1 Concomitant Medications and Non-drug Therapies 20
5.2.3 Protocol Deviations 21
5.2.4 Study Drug Exposure and Study Drug Compliance 21
5.2.5 COVID-19 Related Analysis 21
5.2.5.1 Accounting of subjects who discontinued due to COVID-19 21
5.2.5.2 Concomitant Non-Drug Treatment for COVID-19 22
5.2.5.3 COVID-19 Effect on Drug Compliance 22
5.2.5.4 COVID-19 Protocol Deviation 22
5.2.5.5 Protocol Alternation Due to COVID-19cx 22
5.3 EFFICACY ANALYSIS 22
5.3.1 General Considerations 22
5.3.2 Method of Analysis 23
5.3.2.1 Considerations for base MMRM model for change from baseline analyses 23
5.3.3 Primary Efficacy Endpoint 23
5.3.4 Exploratory Efficacy Endpoints 24
5.4 SAFETY ANALYSES 24
5.4.1 General Considerations 24
5.4.1.1 Analysis Population 24
5.4.1.2 LTE Safety Treatment Groups 24
5.4.1.3 Incidence, Incidence Proportion and Incidence Rate 25
5.4.2 Clinical Adverse Events 25
5.4.2.1 Summary and Incidence Analysis 26
5.4.2.2 Incidence Rate Analysis 28
5.4.2.3 Infusion Reactions 28
5.4.2.4 AE of Special Interest 28
5.4.3 Clinical Laboratory Data 29
5.4.3.1 Quantitative analyses 29
5.4.3.2 Qualitative analyses 30

5.4.4 C-SSRS Data ... *31*
5.4.5 ECG Data ... *31*
5.4.5.1 ECG analyses ... 31
5.4.6 Vital Sign Data ... *32*
5.4.6.1 Vital Sign Data Analyses ... 32
5.4.7 Physical Examination ... *33*
5.4.8 Neurological Examination ... *33*
5.4.9 Safety MRI Data ... *33*
5.4.10 COVID-19 Related Safety Analysis ... *33*
5.5 PHARMACOKINETICS ANALYSIS ... 34
5.5.1 Serum and CSF PK Concentration Profile ... *34*
5.5.1 Serum PK Parameters ... *35*
5.6 BIOMARKER ... 35
5.6.1 Pharmacodynamics Analysis ... *35*
5.6.2 Structural MRI Analysis ... *35*
5.6.3 Tau PET Analysis ... *35*
5.6.3.1 By visit summary ... 36
5.7 ANTI-DRUG ANTIBODY ANALYSIS ... 36
5.7.1 Analysis Methods for Anti-Drug Antibody Data ... *36*
5.8 ADDITIONAL EXPLORATORY ENDPOINTS ... 36
5.8.1 Health Outcomes ... *36*

6 APPENDIX I: VISIT WINDOW MAPPING ... 37

REFERENCE ... 47

List of Abbreviations

AD	Alzheimer's disease
ADA	Anti-drug Antibody
ADAS-Cog 13	Alzheimer's Disease Assessment Scale-Cognitive Subscale (13 items)
ADCOMS	AD Composite Score
ADCS-ADL	Alzheimer's Disease Cooperative Study - Activities of Daily Living Inventory
ADCS-iADL	ADCS-ADL Instrumental Total Score
ADCS-bADL	ADCS-ADL Basic Total Score
AE	adverse event
ALP	alkaline phosphatase
ALT	alanine aminotransferase
APTT	Activated Partial Thromboplastin Tim
AST	aspartate aminotransferase
BLQ	below the limit of quantification
BMI	body mass index
bpm	beats per minute
BUN	blood urea nitrogen
CDR	Clinical Dementia Rating
CDR-SB	Clinical Dementia Rating-Sum of Boxes
CI	confidence interval
C_{trough}	Observed trough serum BIIB092 concentration collected at end of dosing interval (before next infusion starts)
CSF	cerebrospinal fluid
C-SSRS	Columbia Suicide Severity Rating Scale
CV	coefficient of variation
DKEFS	Delis-Kaplan Executive Function System
DSST	Digit Symbol Substitution Test
ECG	electrocardiogram
EMACC	Early AD/ MCI Alzheimer's Cognitive Composite
Emax	Maximum response
EOS	End of study
EOT	End of treatment
ET	Early termination
FAQ	Functional Activities Questionnaire
FAS	Full Analysis Set
eCOG	Everyday Cognition
ICH	International Conference on Harmonisation
INR	Prothrombin Intl. Normalized Ratio
IRT	interactive response technology
iADRS	The Integrated Alzheimer's Disease Rating Scale
LS	Lease Square Mean
ISLR	International Shopping List Test Delayed Recall
ISLT	International Shopping List Test Immediate Recall
LLOQ	lower limit of quantification
LTE	long-term extension
LP	Lumbar puncture
MA	macrohemorrhages
MCI	mild cognitive impairment

MedDRA	Medical Dictionary for Regulatory Activities
mH	microhemorrhages
MMRM	mixed-model repeated measures
MMSE	Mini-Mental State Examination3
MRI	magnetic resonance imaging
NPI-10	Neuropsychiatric Inventory-10
PC	Placebo control
PCP	Placebo control period
PCS	potentially clinically significant
PD	Pharmacodynamics(s)
PET	positron emission tomography
PLS	Partial least squares
PK	pharmacokinetic(s)
PT	Preferred term
PT	Prothrombin Time
Qol-AD	Quality of Life for Alzheimer's Desease
QTcF	Corrected QT interval by Fredericia
ROI	region of interest
RUD-Lite	Resource Utilization in Dementia – Lite Version
SAE	serious adverse event
SAP	statistical analysis plan
SD	standard deviation
SOC	System organ class
SS	superficial siderosis
SUVR	standard uptake value ratio
TEAE	treatment-emergent adverse event
TIV	Total Intracranial Volume
Trails A	Trail Making Test, Part A
ULN	upper limit of normal
VE	vasogenic edema
WHO	World Health Organization
ZBI	Zarit Burden Interview

CONFIDENTIAL
The information contained herein may not be used, disclosed, or published without the written consent of Biogen MA Inc.

1 Introduction

This statistical analysis plan (SAP) covers the analyses of the LTE period and the analyses across both the placebo-controlled period and the LTE period of the study, 251AD201. A separate SAP has been prepared for the analyses of the placebo-controlled period.

Unless stated otherwise, all statistical tests will be 2-sided with statistical significance level of 0.05.

The statistical software, SAS® will be used for all summaries and analyses.

2 DESCRIPTION OF LONG-TERM EXTENSION (LTE) OBJECTIVES AND ENDPOINTS

2.1 Primary LTE Objective and Endpoint

The primary objective for the long-term extension (LTE) period is to evaluate the long-term safety and tolerability of BIIB092 in participants with MCI due to AD or with mild AD.

The primary safety endpoint that relates to this objective is the incidence of AEs and SAEs over the placebo-controlled period and LTE period of the study.

2.2 Exploratory LTE Objectives and Endpoints

- To further evaluate the immunogenicity of BIIB092 after multiple doses in participants with MCI due to AD or with mild AD as measured by the incidence of anti-BIIB092 antibodies in serum over time up to Week 238.
- To further assess the effect of BIIB092 on disease progression as measured by fluid and radiological biomarkers, as well as additional clinical and health outcomes as measured by the following:
    - Changes over the placebo-controlled period and LTE period on the CDR-SB, MMSE, ISLT, Category Fluency and Letter Fluency tests from the DKEFS, DSST, Trails A, eCog, ADCS-ADL, FAQ, ADAS-Cog-13 (13-item), and NPI-10
    - Changes over the placebo-controlled period and LTE period on the ZBI, QoL-AD, and RUD-Lite
    - Changes over the placebo-controlled period and LTE period in CSF and blood biomarkers (for those who consent to participate in the optional CSF sampling substudy)
    - Changes over the placebo-controlled period and LTE period on 18F-MK6240 PET and MRI brain morphometric measures (for those who consent to participate in the optional PET substudy)
- To assess BIIB092 PK in serum (trough serum BIIB092 concentrations) and in CSF (for those who consent to participate in the optional CSF sampling substudy) from the samples collected at the visits indicated in the Schedule of Activities in participants with MCI due to AD or with mild AD.

2.3 Study Design

This is a Phase 2, randomized, double-blind, placebo-controlled, parallel-group study consisting of a double-blind, placebo-controlled period and an LTE period.

The study includes 2 substudies to address exploratory study objectives: a tau PET substudy and a CSF sampling substudy. The tau PET substudy is mandatory at study sites that have access to the ^{18}F-MK6240 PET radioligand and have the capability to perform ^{18}F-MK6240 PET scans. During the placebo-controlled period, the CSF sampling substudy is mandatory at all study sites that do not have access to the 18F-MK6240 PET radioligand. Participants will provide consent during Screening to participate in at least 1 of these substudies during the placebo-controlled period. Participants enrolled in the LTE period and who are participating in the tau PET substudy are required to continue participation in the tau PET substudy. Participation in the CSF sampling substudy during the LTE will be optional for all study participants, although participation is encouraged.

This study will be conducted in participants aged 50 to 80 years, inclusive, with MCI due to AD or mild AD according to National Institute on Aging-Alzheimer's Association criteria. Participants must have amyloid beta positivity confirmed at Screening by either CSF sampling or an amyloid PET scan. Participants must also perform at least 1 standard deviation below the age-adjusted normative mean on either the ISLT or the International Shopping List Test Delayed Recall and have a CDR global score of 0.5 for MCI due to AD or 0.5 or 1 for mild AD, an MMSE score of 22 to 30 (inclusive), and a CDR Memory Box score of ⩾0.5.

During the dose-blinded LTE period, participants will receive BIIB092 by IV once every 4 weeks beginning at Week 80. Participants who were randomized to receive BIIB092 during the placebo-controlled period will continue to receive BIIB092 at the dose they were randomly assigned. Participants randomized to receive placebo during the placebo-controlled period will receive BIIB092 at the high dose (2000 mg) once every 4 weeks during the LTE period.

The total duration for participants who complete the placebo-controlled period and do not enter the LTE period will be approximately 99 weeks. For participants who complete both the placebo-controlled period and the LTE period, the total study duration will be approximately 247 weeks.

Participants who meet the LTE inclusion and exclusion criteria will be eligible to enter the dose-blinded LTE period, which includes the LTE Screening Period of approximately 4 weeks starting at Week 76, the 144-week Treatment Period starting at Week 80, the EOS Visit at Week 226, and the Follow-up Visit at Week 238.

Participants will have approximately 39 outpatient clinic visits during the LTE period:

- Participant eligibility will be determined at Week 76 or Week 78 of the placebo-controlled period and confirmed at Week 80 of the LTE period.
- At the Week 80 Visit, eligible participants will have scheduled assessments per the Schedule of Activities and receive the first infusion of BIIB092 during the LTE period. All participants receiving placebo in the placebo-controlled period will receive BIIB092 at the high dose (2000 mg) once every 4 weeks during the LTE period.
- Participants will return to the clinic at 4-week intervals during the Treatment Period for infusions of study treatment and study assessments.

- The last dose of study treatment will be administered at Week 224 (End-of-Treatment Visit). The EOS Visit will occur at Week 226 and the Follow-up Visit at Week 238.
  - Participants who discontinue study treatment prematurely will be asked to remain in the study and continue all protocol-specified visits and procedures. These participants should be encouraged to attend at least the Week 104, 128, 152, 176, 200, and 226 Visits, depending on when treatment was discontinued, and undergo all the scheduled procedures. At a minimum, these visits should include assessment of AEs/SAEs and concomitant medications and key clinical assessments, including at least the CDR, MMSE, ISLT, ADAS-Cog 13, ADCS-ADL, and FAQ. Participants who discontinue study treatment prematurely will also be asked to return to the study site for a Follow-up Safety Visit 14 weeks after receiving the last dose of study treatment.
  - Participants who withdraw from the study prematurely are to return to the study site for an Early Termination Visit and assessments and for the Follow-up Safety Visit 14 weeks after receiving the last dose of study treatment.

Certain visits during the study may be conducted as home visits as specified in the Schedule of Activities, if the Investigator is in agreement and appropriate services are available to perform the required study procedures and adequately monitor for potential safety events.

CONFIDENTIAL
The information contained herein may not be used, disclosed, or published without the written consent of Biogen MA Inc.

2.4 Study Schematic

Figure 1: Study Design

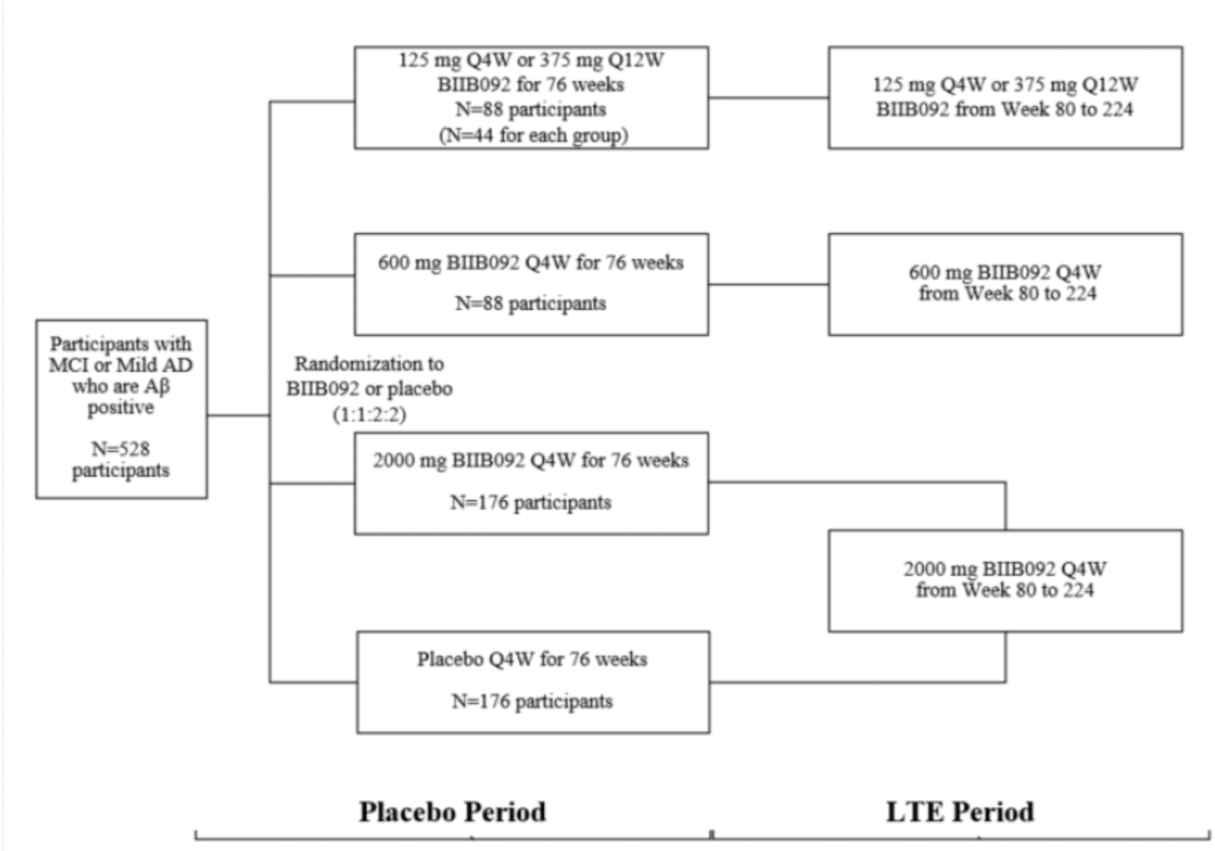

Aβ = amyloid beta; AD = Alzheimer's disease; EOS = End of Study; LTE = long-term extension; MCI = mild cognitive impairment; N = number of participants; Q4W = once every 4 weeks; Q12W = once every 12 weeks

Note: Overall, participants will have a 2:1 chance of being randomized to BIIB092 versus placebo during the placebo-controlled period, and all participants will receive BIIB092 during the dose-blinded LTE period.

Note: The study comprises a double-blind, placebo-controlled period and an LTE period. The total duration for participants who complete the placebo-controlled period and do not enter the LTE period will be approximately 99 weeks. The total study duration for participants who complete both the placebo-controlled period and the LTE period will be approximately 247 weeks. The double-blind placebo-controlled period comprises a Screening Period of approximately 9 weeks (65 days), a Treatment Period of 76 weeks, and for participants not entering the LTE period, an EOS Visit at Week 78 and a Follow-up Safety Visit at Week 90, or approximately 14 weeks after the last dose of study treatment. The LTE period comprises an LTE Screening Period of approximately 4 weeks starting at Week 76, a 144-week Treatment Period starting at Week 80, an

EOS Visit at Week 226, and a Follow-up Safety Visit at Week 238, or approximately 14 weeks after the last dose of study treatment.

3 Definitions

3.1 Analysis Sets

- Full Analysis Set (FAS):
  The FAS includes all randomized subjects who received at least one dose of study treatment (BIIB092 or placebo). In analyses performed on the FAS, subjects will be analyzed, based on the intention-to-treat principle, according to their randomized treatment assignment regardless of treatment received.

- Safety Analysis Set:

  The safety Analysis Set includes all randomized subjects who received at least one dose of study treatment (BIIB092 or placebo), essentially the same set of participants included in the FAS. In analyses performed on the Safety Analysis Set, subjects will be analyzed according to their actual treatment received.

- Safety MRI Evaluable Set:
  The Safety MRI Evaluable Set is defined as subjects in the FAS who had at least one post-baseline safety MRI scan.

- Serum PK Evaluable Set:
  The serum PK evaluable set is defined as subjects in the FAS who had at least one measurable post-baseline BIIB092 concentration in serum before the End of Study of LTE.

- CSF PK Evaluable Set:
  The CSF PK evaluable set is defined as subjects in the FAS who had at least one measurable post-baseline BIIB092 concentration in CSF.

- CSF PD Evaluable Set:
  The CSF PD evaluable set is defined as subjects in the FAS who had lumbar puncture (LP), which will be used in analyses, such as subject accounting and summary of AE related to LP. The CSF PD Modified Evaluable Set is defined as subjects in the FAS who have baseline and at least one post baseline assessment of the specific parameter being analyzed in CSF.

- Tau PET Evaluable Set
  The tau PET evaluable set is defined as subjects in the FAS who had tau PET, which will be used in analyses, such as subject accounting and summary of AE related to tau PET. The tau PET Modified Evaluable Set is defined as subjects in the FAS who had a valid baseline and a post-baseline tau PET SUVR measure using the 18F-MK6240 tracer.

- Anti-drug antibody (ADA) Evaluable Set:
  The evaluable set for anti-drug antibody is defined as subjects in the FAS who have an evaluable post-baseline ADA sample.

3.2 Analyses Period

Depending on the purpose, different analyses will be conducted on the following study periods:

1. LTE period. Only data in the LTE period will be included in these analyses. This LTE analysis period will be applied to all the AE analyses, and the analyses will include all subjects who were dosed in the LTE period.
2. Placebo-controlled and LTE period. All the data in the placebo-controlled and LTE periods will be included in these analyses. This analysis period will be applied to analyses including, but not limited to all efficacy analyses and listings. The analyses will include all subjects who were dosed in the study including those who did not enroll into the LTE period. For example, line plot of CDR sum of box mean change from baseline over time. in the placebo-controlled and LTE period.
3. Placebo-controlled and LTE active treatment period. Active treatment period is defined as the study period(s) that a subject received BIIB092. For early start subjects – subjects who received BIIB092 in both placebo-controlled and LTE period, all the data (placebo-controlled and LTE periods) will be included in the analyses. For late start subjects – subjects who received placebo in the Placebo-controlled period and BIIB092 in the LTE period, only data in the LTE period will be included. This analysis period will be applied to majority of safety tables, and the analyses will include all subjects who were dosed in the study except for subjects who received only placebo in the placebo-controlled period and did not get dosed in the LTE period. An example is the incidence rate of adverse events in the placebo-controlled and LTE active treatment period.

Subjects to be included in a certain output is determined by both the analysis set and the analysis period. For example, the incidence table of adverse events in the LTE period will include subjects in the safety population for the LTE period, i.e., all randomized subjects who received at least one dose of study treatment in the LTE period. The incidence rate table of adverse events in the placebo-controlled and LTE active treatment period will include subjects in the safety population for the active treatment period, i.e., all randomized subjects who received at least one dose of study treatment in the active treatment period. In this SAP we do not separately define the analysis set in each analysis period.

3.3 Study Treatment

For efficacy and health outcome analyses, the following LTE treatment groups of BIIB092 (per randomization) will be evaluated:

- low-dose (BIIB092 -125mg once every 4weeks or 375mg once every 12weeks)

- medium-dose (BIIB092 - 600mg once every 4 weeks)
- early start high-dose (BIIB092 - 2000mg once every 4 weeks)
- late start high-dose (BIIB092 - 2000mg once every 4 weeks)

For other analyses, such as safety, PK, PD, biomarker, and anti-drug antibody analyses, low dose group will be split out for different frequencies. The following LTE treatment groups of BIIB092 will be evaluated:

- low-dose 125mg once every 4weeks
- low-dose 375mg once every 12weeks
- medium-dose (BIIB092 - 600mg once every 4 weeks)
- early start high-dose (BIIB092 - 2000mg once every 4 weeks)
- late start high-dose (BIIB092 - 2000mg once every 4 weeks)

Since subjects in low-dose group and medium-dose group are taking the same treatment from the start of placebo-controlled period to the end of LTE period, so the subjects in these treatment groups are all considered as early start subjects. Late start subjects refer to subjects who were on placebo during placebo-controlled period then switched to high-dose (BIIB092 - 2000mg once every 4 weeks). Throughout this SAP, these study treatment schemes will be referred as LTE treatment.

3.4 Dates and Points of Reference

The study day and baseline are defined for each analyses period respectively

1. LTE period
   - Study Day 1: the date of the first dose of study treatment in the LTE period
   - Study Day
     - For a date on or after Study Day 1

       Study Day = (Date of Interest) – (Study Day 1) + 1
     - For a date before Study Day 1

       Study Day = (Date of Interest) – (Study Day 1)
   - LTE baseline: the baseline value for the LTE period is defined as the most recent non-missing measurement collected prior to the first dose in the LTE period.
   - Change from baseline will be defined as post-baseline value minus baseline value
2. Placebo-controlled and LTE period
   - Study Day 1: the date of the first dose of study treatment in the PC period

- Study Day
    - For a date on or after Study Day 1

      Study Day = (Date of Interest) – (Study Day 1) + 1
    - For a date before Study Day 1

      Study Day = (Date of Interest) – (Study Day 1)
- PC and LTE baseline: the baseline value for the Placebo-controlled and LTE period is defined as the most recent non-missing measurement collected prior to the first dose in the PC period.
- Change from baseline will be defined as post-baseline value minus baseline value

3. Placebo-controlled and LTE active treatment period

- Study Day 1: the date of the first dose of BIIB092. For low-dose, medium-dose and early start high-dose subjects, this will be the date of first dose of BIIB092 in the placebo-controlled (PC) period; for late start high-dose subjects, this will be the date of first dose of BIIB092 in the LTE period.
- Study Day
    - For a date on or after Study Day 1

      Study Day = (Date of Interest) – (Study Day 1) + 1
    - For a date before Study Day 1

      Study Day = (Date of Interest) – (Study Day 1)
- Active treatment baseline: the baseline value for the active treatment period and is defined as the most recent non-missing measurement collected prior to study day 1.
- Change from baseline will be defined as post-baseline value minus baseline value

  For data that are summarized by visit, assessment from all scheduled visits, EOS visit and unscheduled visits will be mapped to an appropriate analysis visit using a windowing scheme as describe in Appendix I

3.5 Key Derived Variables

- Handling of missing items for scales
    - If any of the individual items for the primary efficacy endpoint and exploratory efficacy endpoints is missing, the total score of the corresponding endpoint will be imputed by prorating the observed scores [van Ginkel 2010].
    - For ADAS-Cog13, if 3 or fewer of 13 items (<25%) are missing, the total score will be imputed by the following algorithm: Total score = total score from the completed items * [maximum total score (=85) / maximum total score for the

CONFIDENTIAL
The information contained herein may not be used, disclosed, or published without the written consent of Biogen MA Inc.

completed items]. The imputed number will be rounded up to the nearest integer. If more than 3 items are missing, the total score of ADAS-Cog 13 at that visit will be considered missing.

  - For ADCS-ADL, if 8 or fewer of 32 items (<25%) are missing, the total score will be imputed by a similar algorithm as that for ADAS-Cog 13. The imputed number will be rounded down to the nearest integer. If more than 8 items are missing, the total score for ADCS-ADL at that visit will be considered missing.
  - The same imputation algorithm will be applied to CDR-SB and MMSE, if only 1 box (of 6) of CDR is missing or if only 2 or fewer items (out of 11) are missing for MMSE. The imputed CDR-SB will be rounded up to the nearest half integer, and the imputed MMSE will be rounded down to the nearest integer. If the score from more than 1 box of CDR or more than 2 items of MMSE is not available, the CDR-SB or MMSE at that visit will be considered missing.
  - The total score of the tertiary endpoint NPI-10 and FAQ will be imputed using the same prorating principle and round up to the nearest integer if only 1 item (out of 10) is missing.

- iADRS derivation

The iADRS is a composite score based on ADAS-Cog and ADCS-iADL (instrumental ADCS-ADL) [Wessels et al. 2015, Wessels et al. 2018]. The iADRS is calculated as a linear combination of total scores of the two individual components, the ADAS-Cog13 (score range 0 to 85) and the ADCS-iADL (score range 0 to 59). Because higher score on the ADAS-Cog13 reflect worse performance, whereas higher scores on the ADCS-iADL reflect better performance, the ADAS-Cog score is multiplied by (-1) in the calculation of the integrated scale. To anchor the ADAS-Cog at 0, a constant (85) is added. The iADRS score is then computed as the sum of the transformed ADAS-Cog13 and the ADCS-ADL, as shown in the formula below:

iADRS score = [(-1) (ADAS-Cog13) + 85] + ADCS-iADL

The iADRS score ranges from 0 to 144 with lower scores indicating worse performance. If either ADAS-Cog13 or ADCS-iADL is missing, the iADRS score will be considered missing.

- EMACC derivation

The EMACC is a new and sensitive composite of well-known and validated neuropsychological tests that is suitable for examining the effect of disease modifying compounds on cognitive decline in the early Alzheimer Disease or MCI stage of Alzheimer's disease [Jaeger et al. 2018].

Cognitive variables (ISLT, DKEFS Category Fluency total correct score, DKEFS Letter Fluency, DSST, Trails A total time to complete) will be z-score transformed using the

baseline score's mean and SD. The EMACC will be computed by taking the average of the z-scores across the five tests. If Trails A is missing, EMACC will be computed by taking the average of the z-scores across the remaining four tests. For the rest 4 component scores, if any of them is missing then the composite score will be missing.

- ADCOMS derivation

ADCOMS is a novel instrument developed to improve the sensitivity of currently available cognitive and functional measures for subjects in the prodromal stage of AD and mild AD dementia. It consists of 4 Alzheimer's Disease Assessment Scale–cognitive subscale items, 2 Mini-Mental State Examination items, and all 6 Clinical Dementia Rating—Sum of Boxes items (Table 1). The composite score is a weighted linear combination of the individual scales items using the corresponding partial least squares (PLS) coefficients as weighting factors as listed in Table 1 [Wang et al. 2016]. If any of the individual item is missing, then the composite score will be missing. The range of ADCOMS is between 0 and 1.97.

Table 1. Items included in ADCOMS and their corresponding PLS coefficients

Scale	Item name	PLS coefficients
ADAS-cog	Delayed word recall	0.008
	Orientation	0.017
	Word recognition	0.004
	Word finding difficulty	0.016
MMSE	Orientation time	0.042
	Drawing	0.038
CDR-SB	Personal care	0.054
	Community affairs	0.109
	Home and hobbies	0.089
	Judgement and problem solving	0.069
	Memory	0.059
	Orientation	0.078

ADAS-cog, Alzheimer's Disease Assessment Scale–cognitive subscale; CDR-SB, Clinical Dementia Rating, sum of boxes; MMSE, Mini-Mental State Exam; PLS, partial least squares.

3.6 Stratification Factors and Subgroup Variables

Stratification factors are:

- Tau PET/CSF sampling substudy enrollment (If subjects enrolled in both substudies, subjects will be counted as tau PET substudy)
- Region (US, Australia, Japan, EU < EU countries include France, Germany, Italy, Spain, and Sweden >, Poland)
- Baseline disease stage (MCI or mild AD)
- Baseline AD symptomatic medication use (Yes/No)

All stratification factors are based on subjects' status at PC baseline. A comprehensive list of baseline AD symptomatic medication can be found in PC SAP.

No subgroup analyses will be conducted.

4 List of Planned Study Analyses

There are two milestone LTE analyses planned for this study: LTE analysis at PC period database lock and the final analysis. The final analysis, which is also known as End of LTE analysis, will only be performed when the LTE study is completed.

4.1 LTE interim analyses at PC period database lock

The LTE interim analyses (IA) will be performed at the PC period database lock after the last patient out in the PC period. All the efficacy analyses described in Section 5.3 will be performed with available efficacy data up to week 104 at the PC period database lock. Given the timeline of scheduled data collection, only a few subjects will have tau PET, CSF biomarker and health outcomes data collected in LTE visits. No LTE analyses of tau PET, biomarker or health outcomes will be included at LTE IA at PC period database lock.

4.2 Final analysis

The LTE analyses will be performed at the end of the LTE period database lock after the last patient out in the LTE period. All the analyses described in Section 5 will be performed with complete data of in the study.

CONFIDENTIAL
The information contained herein may not be used, disclosed, or published without the written consent of Biogen MA Inc.

5 Statistical Methods for Planned Analyses

5.1 General Considerations

Summary tables will be presented using descriptive summary statistics. For continuous variables, summary statistics will generally include: number of subjects with data, mean, standard deviation, median, 25% percentile, 75% percentile, minimum and maximum. For categorical variables, this will generally include: number of subjects randomized or dosed, number with data, and the percent of those with data in each category.

5.2 Study Subjects

The summaries in this section will be based on the FAS. Analysis period will be specified for each table or figure. Unless otherwise specified, summary tables will be presented by LTE treatment group. If not otherwise specified in later text in this SAP, listings will include all data in the placebo-controlled and LTE periods (all data in the study), with an indicator of the study period (pre-dosing, placebo-controlled, or LTE) for each record to indicate when the event occurred. Listings will be presented by LTE treatment group.

5.2.1 Accounting of Subject

Disposition in the LTE period will be summarized for subjects enrolled in LTE. The summary data will include number (%) of subjects randomized and dosed, number (%) of subjects who completed the treatment/study in LTE, and number (%) of subjects who discontinued treatment and/or withdrew from study in LTE. For subjects who discontinued treatment and/or withdrew from study, the reasons for discontinuation and/or withdrawal, days on treatment and days on study in LTE period will be summarized and listed.

Number (%) of subjects who completed the placebo-controlled period but did not enter the LTE period, number (%) of subjects enrolled in LTE (who signed the LTE informed consent) and number (%) of subjects dosed in LTE period will be summarized by LTE treatment group.

5.2.2 Demographics and Baseline Characteristics

Demographics, baseline characteristics, medical history and AD treatment history at PC period baseline will be summarized for subjects enrolled in the LTE period. Please refer to the SAP for the placebo-controlled period.

5.2.2.1 Concomitant Medications and Non-drug Therapies

The number (%) of subjects taking concomitant medication and non-drug therapies in the LTE period will be summarized. In addition, number of subjects in the FAS that have taken any concomitant medications in the placebo-controlled and LTE active treatment period will be summarized.–Concomitant medications and non-drug therapies will be listed for placebo-controlled and LTE period.

For subjects enrolled in the LTE period, the number (%) of subjects taking AD symptomatic medications concomitantly during the LTE period will be summarized. Please refer to the placebo-controlled SAP for definitions of concomitant therapies and AD symptomatic medication use at baseline.

Disallowed Therapies

Per Section 11.4.1.2 of the Protocol, prohibited and/or restricted medications taken prior to study treatment administration and during the study will be monitored. Disallowed therapies will be reported as Protocol Deviations.

5.2.3 Protocol Deviations

Protocol deviations identified during site monitoring will be captured in a Protocol Deviation log and categorized as major or minor deviations based on Protocol Deviation Classification. The major protocol deviations occurred in the LTE period will be summarized for subjects enrolled in LTE. The major protocol deviations for all FAS subjects in the combined placebo-controlled and LTE periods will also be summarized. Major and minor protocol deviations for all FAS subjects will be listed, respectively, across the placebo-controlled period and the LTE period. Subjects who had incorrect dose assigned by interactive response technology (IRT) in LTE will be summarized and listed. This data will be provided by the unblinded monitors. All summaries for protocol deviations will be presented only by LTE treatment group.

5.2.4 Study Drug Exposure and Study Drug Compliance

A summary table of study drug exposure and compliance in the placebo-controlled and LTE active treatment period will be provided. Number of infusions (BIIB092) received will be summarized as a categorical variable (categories as integers from 1 to 20, and 1-5, 6-10, 11-15, 15-20, 21-26) as well as a continuous variable. Number of weeks on study treatment (BIIB092), calculated as (date of last dose – date of first dose +1)/7, will be summarized as a categorical variable (every 8 weeks from 0 to >= 96 weeks) as well as a continuous variable. Percentage of study treatment taken up to the last dose, calculated as (the actual number of infusions / by the number of infusions a subject is expected to take until the date of last infusion) *100, will be summarized as a continuous variable. This table will be presented only by LTE treatment group. Similar outputs will also be generated for LTE period.

A listing of study drug administration records will be provided for the combined placebo-controlled and LTE period.

5.2.5 COVID-19 Related Analysis

5.2.5.1 Accounting of subjects who discontinued due to COVID-19

The disposition table due to COVID-19 will be generated for LTE period. The listing of subjects who discontinued treatment and/or withdrew from study due to COVID-19 will be generated for PC and LTE period. Please refer to the placebo-controlled SAP for more details.

5.2.5.2 Concomitant Non-Drug Treatment for COVID-19

The summary table for the number of subjects using concomitant Non-Drug Treatment for COVID-19 will be summarized for both LTE period and PC and LTE active treatment period. Please refer to the placebo-controlled SAP for more details.

5.2.5.3 COVID-19 Effect on Drug Compliance

Effect of COVID-19 on drug compliance may be assessed for LTE period. Please refer to the placebo-controlled SAP for more details.

5.2.5.4 COVID-19 Protocol Deviation

The table of protocol deviations due to COVID-19 and the summary of impact and reason for major protocol deviations due to COVID-19 will be generated for LTE period. Please refer to the placebo-controlled SAP for more details.

5.2.5.5 Protocol Alternation Due to COVID-19cx

Protocol alternation due to COVID-19 will be listed for PC and LTE period. Please refer to the placebo-controlled SAP for more details.

5.3 Efficacy Analysis

5.3.1 General Considerations

The analysis population for efficacy analysis is the same as the FAS and data from both the placebo-controlled and LTE periods will be include. All efficacy analyses will be presented by study treatment groups as defined in Section 3.3. The early start high-dose subjects are the subjects who were randomized to high-dose BIIB092 group in the PC period, and the late start high-dose subjects are the subjects who were randomized to Placebo in the PC period, regardless of enrollment of the LTE period or not.

The following comparison will be evaluated for the long-term efficacy of BIIB092:

- The early start high dose compared with the late start high dose.

The comparisons between low-dose and late start high dose, and middle-dose and late start high dose might be conducted. There will be no multiple comparison adjustments.

Visit windows for mapping efficacy endpoint

For efficacy data that are summarized by visit, assessment from all scheduled visits, EOS visit and unscheduled visits will be mapped to an appropriate analysis visit using a windowing scheme (Appendix I).

5.3.2 Method of Analysis

5.3.2.1 Considerations for base MMRM model for change from baseline analyses

A mixed model with repeated measures (MMRM) will be used to analyze changes from baseline in a parameter of interest using fixed effects of LTE treatment group, visit (categorical), LTE treatment group-by-visit interaction, baseline of the parameter of interest, baseline of the parameter of interest-by-visit interaction, region (Japan and Australia will be combined), baseline MMSE, baseline disease stage, and baseline AD symptomatic medication use. The correlation between repeated measures of the outcomes will be taken into consideration. An unstructured covariance matrix will be used to model the within-subject variance-covariance errors. If the unstructured covariance structure matrix results in a lack of convergence, the heterogeneous first-order autoregressive covariance structure followed by the heterogeneous Toeplitz covariance structure will be used. The least-squares (LS) means, the differences in LS means between each LTE treatment group versus placebo at each visit, 95% confidence intervals (CIs), and p-values will be presented. In the primary analysis of each endpoint, missing data are assumed to be missing at random [Rubin 1976].

For the LTE interim analysis at PC DBL, only records up to week 104 will be included in the MMRM model.

5.3.3 Primary Efficacy Endpoint

Estimand 1 (treatment policy strategy): The difference in change from baseline CDR-SB scores in subjects assigned to BIIB092 group in the PC period, comparing to late start BIIB092 group, regardless what actual treatment is received acknowledging a participant may miss more than four infusions, consecutively, change AD symptomatic medication and/or discontinue treatment early

- Analysis set: all subjects in the FAS
- Variable: The change from baseline CDR-SB scores at the week of interest regardless of intercurrent events (ICEs)
- Analysis set level summary: Least square (LS) mean difference from MMRM model in change from baseline between BIIB092 and placebo
- ICEs and Strategies for Addressing ICEs:

  ICEs include

  - AD symptomatic medication change (treatment policy strategy)
  - Treatment discontinuation (treatment policy strategy)
  - Missing more than or equal to four infusions consecutively (treatment policy strategy)

Since a treatment policy strategy will be used for all ICEs in this estimand, all observed data will be included regardless of miss more than or equal to four infusions consecutively, treatment discontinuation or AD symptomatic medication change. At LTE interim analysis, the primary

analysis is the mean difference of the change from baseline CDR-SB scores at Week 104 between treatment groups in the FAS who have a baseline and at least one post-baseline CDR-SB score [ICH E9 (R1) Addendum 2014, 2017]. At final analysis, the primary analysis is the mean difference of the change from baseline CDR-SB scores at the week of interest between treatment groups in the FAS who have a baseline and at least one post-baseline CDR-SB score. All observed data will be included in the primary analysis, including data collected after intercurrent events [ICH E9 (R1) Addendum 2017], i.e., treatment discontinuation or a change in concomitant use of AD symptomatic medication

The baseline and change from baseline CDR-SB scores at each post-baseline visit will be summarized by LTE treatment group. A mixed model with repeated measures (MMRM) will be used to analyze changes from Baseline in CDR-SB as described in Section 5.3.2.1.A line plot of adjusted mean change from baseline over time will be provided.

5.3.4 Exploratory Efficacy Endpoints

The clinical endpoints assessing AD progression from Baseline include the results of the MMSE, ADAS-Cog13, ADCS-ADL, ISLT, eCog, FAQ, NPI-10, EMACC (composite score of ISLT, DKEFS, DSST, and Trails A), ADCOMS (composite score of selected items from CDR, MMSE, and ADAS-Cog 13) and iADRS.

The by visit summary and MMRM analysis will be performed for MMSE, ISLT, eCog, ADCS-ADL, FAQ, ADAS-Cog-13 (13-item), NPI-10, EMACC, ADCOMS, and iADRS as described in Section 5.3.2.1. For LTE interim analysis at PC DBL, all available records up to week 104 will be analyzed in MMRM table. The baseline and change from baseline scores at each post-baseline visit will be summarized by LTE treatment group at final analysis.

5.4 Safety Analyses

5.4.1 General Considerations

5.4.1.1 Analysis Population

The safety analysis set will be used for safety analyses of AEs, SAEs, clinical laboratory data, Columbia Suicide Severity Rating Scale (C-SSRS) data, ECG data and vital sign data. The safety MRI evaluable set will be used for the analysis of safety MRI data.

5.4.1.2 LTE Safety Treatment Groups

Since all the subjects are supposed to receive active treatment in LTE, the LTE safety treatment groups which are the same as the LTE treatment groups will be used for all the safety analyses.

CONFIDENTIAL
The information contained herein may not be used, disclosed, or published without the written consent of Biogen MA Inc.

5.4.1.3 Incidence, Incidence Proportion and Incidence Rate

- Incidence and incidence proportion will be provided in incidence proportion tables. Incidence is defined as the number of subjects who experienced an event. Incidence proportion is defined as the number of subjects who experienced an event divided by total number of subjects in the analysis population, i.e., percentage. Each subject will be counted only once within each category.

- Incidence and incidence rate will be provided in incidence rate tables. Incidence rate of an event based on the entire follow-up time – defined as the number of subjects who experienced an event divided by the total of entire follow-up time among the subjects in the analysis set (e.g., incidence rate per 100 subject-years). The entire follow-up time for a subject (subject-years) is defined as the sum of all subjects' follow-up time, where a subject's follow-up time is calculated as the number of days (inclusive) from first dose of study drug until the last day on study, divided by 365.25. Each subject will be counted only once within each category.

5.4.2 Clinical Adverse Events

Treatment-emergent AEs (TEAE)

A treatment-emergent AE is defined as AE that started or worsened after the start of first infusion of study treatment in LTE period.

First dose for below definitions is the first dose in LTE period

To define treatment emergence for AEs with missing start or stop date or time the following additional criteria will be used:

- if both the start and stop dates for a particular AE are missing, then that AE is considered treatment emergent;
- if the start date for a particular AE is missing and the stop date/time falls after the first dose date/time, then that AE is considered treatment emergent;
- if the start date for a particular AE was the same as the first dose date, and the start time was missing and stop time is after the first dose or missing, then that event is considered treatment emergent.

For AEs with a partial start date, the following imputation method will be used to determine if the event is treatment emergent:

- When only the day is missing, and the year/month is equal to the year/month of first dose date, and the AE stop date is missing or on/after the first dose date, then use the first dose date as AE start date. Otherwise impute the AE start date as the first day (1) of the AE start month
- When the AE start month is missing, and the year equals to the year of first dose date, and the AE stop date is missing or on/after the first dose date, then use the first dose date as AE start date. Otherwise impute the AE start date as the first day (01January) of the AE start year

For AEs with a partial end date, the following imputation method will be used:

- When only the day is missing, and the year/month equal to the year/month of the last date on study, then use the last date on study as AE stop date. Otherwise impute the AE stop date as the last day of the AE stop month
- When the AE stop month is missing, and the year equals to the year of last date on study, then use the last date on study as AE stop date. Otherwise impute the AE stop date as the last day (31December) of the AE stop year

Only TEAEs will be included in the tables, unless otherwise specified. All SAEs (including pre-dosing SAEs) will be included in the listing of SAEs, with an indicator of pre-dosing or treatment-emergent. Only TEAEs will be included in other AE listings, if not otherwise specified. A listing of AE will be provided for the combined placebo-controlled and LTE period.

Pre-treatment AE: AE starting before LTE first dose are considered pre-treatment AE for LTE period if it does not qualify for above TEAE definition i.e., no worsening.

Analysis period and analysis displays

Analysis period will be specified for each output. However, for either analysis period in this SAP (LTE period or placebo-controlled and LTE active treatment period), AE data will be summarized by LTE treatment group (5 LTE treatment groups as low-dose BIIB092 125mg/4weeks, low-dose BIIB092 375mg/12weeks, medium-dose, early start high-dose, late start high-dose, and BIIB092 total). Listings will include all data in placebo-controlled and LTE period (all data in the study), with an indicator of the study period (pre-dosing, placebo-controlled, or LTE) for each record when the event occurred.

5.4.2.1 Summary and Incidence Analysis

Overall summary of AE table will be done for the LTE period and placebo-controlled and LTE active treatment period, presented by LTE treatment group. The following information will be summarized: the number of subjects with any AE, with any AE by maximum severity, the number of subjects with any related AE (related to study drug as assessed by investigator), the number of subjects with SAE, the number of subjects with related SAE, the number of subjects with AE leading to drug withdrawal, the number of subjects with AE leading to study withdrawal, and the number of subjects with a fatal event.

The sorting order of AE incidence tables, unless otherwise specified, will be by decreasing frequency order of “BIIB092 total” column within each category in the tables presented by LTE treatment group. A subject is counted only once within each category in each table. For example, for the table of AEs by system organ class and preferred terms sorted by decreasing frequency presented by LTE treatment group, system organ class will be presented in decreasing frequency order of BIIB092 total column, and within each system organ class, preferred terms will be presented in decreasing frequency order of BIIB092 total column. A subject is counted only once within each system organ class and preferred term.

CONFIDENTIAL
The information contained herein may not be used, disclosed, or published without the written consent of Biogen MA Inc.

The following AE incidence tables will be provided for the LTE period. Selected tables (marked with *) will be presented for incidence rate of AEs for the placebo-controlled and LTE active treatment period:

1. AEs by system organ class and preferred term sorted by decreasing frequency *
2. AEs by system organ class and preferred term sorted by alphabetical order
3. AEs by system organ class *
4. AEs at least 2% higher in incidence by system organ class and preferred term for early start BIIB092 2000 mg/4wk compared to late start BIIB092 2000 mg/4wk presented only for LTE period
5. AEs by preferred term *
6. AEs by preferred term with an incidence of 5% or more
7. AEs by maximum severity by system organ class and preferred term by decreasing frequency (System organ class will be presented alphabetically. Preferred terms will be presented in decreasing frequency order. Maximum severity will be presented within each preferred term in the order of mild, moderate, severe, unknown and total. A subject will be counted only once at the maximum severity within each system organ class and preferred term.)
8. AEs by maximum severity by preferred term
9. Severe AEs by system organ class and preferred term by decreasing frequency *
10. Severed AEs by preferred term *
11. Related AEs by system organ class and preferred term by decreasing frequency *
12. AEs related to tau PET ligand by system organ class and preferred term by decreasing frequency (using tau PET population)
13. SAEs by system organ class and preferred term by decreasing frequency *
14. SAEs by preferred term *
15. Related SAEs by system organ class and preferred term by decreasing frequency *
16. SAEs with fatal outcome by system organ class and preferred term by decreasing frequency
17. AEs that led to drug interrupted by system organ class and preferred term by decreasing frequency
18. AEs that led to discontinuation of study treatment by system organ class and preferred term by decreasing frequency
19. AEs that led to withdrawal from study by system organ class and preferred term by decreasing frequency
20. AEs related to lumbar puncture (LP) by system organ class and preferred term (using CSF population)

The following listings will be provided.

1. Listing of AEs
2. Listing of SAEs (including pre-dosing SAEs)
3. Listing of AEs that led to infusion interruption
4. Listing of AEs that led to discontinuation of study drug
5. Listing of AEs that led to withdrawal from study
6. Listing of AEs related to tau PET ligands
7. Listing of SAEs with fatal outcome
8. Listing of AEs related to lumbar puncture (LP)
9. Listing of death
10. Listing of pre-treatment AEs

5.4.2.2 Incidence Rate Analysis

Incidence rate of AEs based on the entire follow-up time for subjects with at least 1 AE in LTE period may be summarized by system organ class and preferred terms. The incidence rate tables will be presented by LTE treatment group. The entire follow-up time for LTE period is from the first dose in LTE period until the last day on study.

Follow-up adjusted incidence rate of AEs may also be summarized for placebo-controlled and LTE active treatment period, ue to the different length of placebo-controlled and LTE active treatment period in early start versus late start high-dose BIIB002 subjects. The entire follow-up time is from the first dose in active treatment period (placebo-controlled period first dose of BIIB092 for early start subjects and LTE period first dose of BIIB092 for late start subjects) until the last day in the study.

5.4.2.3 Infusion Reactions

Please refer to the placebo-controlled period SAP for infusion reaction definitions. The same infusion reaction tables as described in placebo-controlled period SAP will be summarized for LTE period by LTE treatment groups.

5.4.2.4 AE of Special Interest

Please refer to the placebo-controlled period SAP for AEs of special interest. The same tables as described in placebo-controlled period SAP will be summarized for LTE by LTE treatment groups.

CONFIDENTIAL
The information contained herein may not be used, disclosed, or published without the written consent of Biogen MA Inc.

5.4.3 Clinical Laboratory Data

The following clinical laboratory parameters are assessed in the protocol:

- Hematology: red blood cell count, platelet count, hemoglobin level, hematocrit, mean corpuscular volume, mean corpuscular hemoglobin, mean corpuscular hemoglobin concentration, and total white blood cell count with absolute counts and percentages of neutrophils, monocytes, lymphocytes, eosinophils, and basophils
- Blood chemistry: total protein, albumin, creatinine, BUN, uric acid, bilirubin (total and direct), alkaline phosphatase, ALT, AST, lactate dehydrogenase, gamma-glutamyl transferase, glucose, calcium, phosphorus, bicarbonate, chloride, sodium, and potassium
- Urinalysis: color, specific gravity, pH, protein, glucose, leukocyte esterase, blood, and ketones (and microscopic examination, if abnormal)
- Coagulation: Activated Partial Thromboplastin Time (APTT), Prothrombin Intl. Normalized Ratio (INR), Prothrombin Time (PT)

Analysis period will be specified for each table or figure. Unless otherwise specified, all the laboratory tables will be summarized by LTE treatment group. Listings will include all data in the study, with an indicator of the study period (pre-dosing, placebo-controlled, or LTE) for each record when the assessment occurred. Listings will be presented by PC treatment group.

Study day and analysis visit window for analyses using LTE baseline will be derived based on the first day of study drug in LTE. Details are specified in Appendix I.

5.4.3.1 Quantitative analyses

For numeric laboratory parameters, actual values will be summarized by visit for all the visits in the placebo-controlled and LTE active treatment period. Number of evaluable subjects, mean, standard deviation, 25% and 75% quartiles, min and max values will be presented at each visit. Plots of mean actual values (with standard error) at each visit for all the visits in the placebo-controlled and LTE active treatment period will be provided.

Summary of change from baseline and percent change from baseline for numeric laboratory parameters will be done on the placebo-controlled and LTE active treatment period. LTE baseline will be used for late start high-dose group subjects, while placebo-controlled baseline will be used for the rest of the subjects (low-dose BIIB092 125mg/4weeks, low-dose BIIB092 375mg/12weeks, medium-dose, early start high-dose). Number of evaluable subjects, mean, standard deviation, 25% and 75% quartiles, min and max values will be presented at each visit.

Listings of individual laboratory measurements by patients for all the parameters will be provided.

Visit windows for by visit summaries

For Laboratory data that are summarized by visit, assessment from all scheduled visits, EOT visit, EOS visit and unscheduled visits will be mapped to an appropriate analysis visit using a windowing scheme (Appendix I).

5.4.3.2 Qualitative analyses

For qualitative analyses, all values will be included (not just the "analyzed record" within each visit window in the quantitative analyses).

Shift analyses

Laboratory data will be summarized using shift tables where appropriate for the placebo-controlled and LTE active treatment period. Each subject's hematology, blood chemistry and urinalysis values will be flagged as "low", "normal", or "high" relative to the normal ranges of the central laboratory or as "unknown" if no result is available.

For each parameter, the analysis will be based on subjects with at least one post-baseline value. Shifts from baseline to high/low status will be presented for hematology, blood chemistry coagulation, serology, and urinalysis. Shift to low includes normal to low, high to low, and unknown to low; Shift to high includes normal to high, low to high, and unknown to high. Subjects need to have at least one post-baseline evaluation and a baseline value not low or high (including missing) in order to be included in the analysis for corresponding categories in the analyses.

For early start subjects, the placebo-controlled baseline will be used and shifts that occurred in either placebo-controlled or LTE period will be included. For late start subjects, the LTE baseline will be used and shifts that occurred in the LTE period based on LTE baseline will be included.

Potentially Clinically Significant laboratory abnormalities analyses

Please refer to the placebo-controlled SAP for the parameters and criteria for potentially clinically significant laboratory abnormalities analyses.

The number of subjects with potentially clinically significant laboratory abnormalities post-baseline will be summarized for the placebo-controlled and LTE active treatment period.

Subjects need to have at least one post-baseline evaluation in the active treatment period and a baseline value not potentially clinically significant (including missing) in order to be included in the analysis.

Same as the shift analysis, for early start subjects, the placebo-controlled baseline will be used and abnormalities that occurred in either placebo-controlled or LTE period will be included. For late start subjects, the LTE baseline will be used and PCS abnormalities that occurred in the LTE period based on LTE baseline will be included.

CONFIDENTIAL
The information contained herein may not be used, disclosed, or published without the written consent of Biogen MA Inc.

Potential serious hepatotoxicity

In this SAP, we define potential serious hepatotoxicity as ALT or AST > 3x ULN and total bilirubin > 2x ULN at any time post-baseline in the placebo-controlled and LTE active treatment period (not necessarily concurrent). A scatterplot of the maximum post-baseline ALT or AST value relative to ULN and maximum post-baseline total bilirubin value relative to ULN (not necessarily concurrent) for each subject will be provided. A line plot of ALT, AST, ALP and total bilirubin values over time in the active treatment period for subjects with potential serious hepatotoxicity will be provided. In addition, subjects with ALT > 1x ULN, >3x ULN, >5x ULN, >10x ULN or >20x ULN, subjects with AST > 1x ULN, >3x ULN, >5x ULN, >10x ULN or >20x ULN, subjects with total bilirubin >1x ULN or >2x ULN, subjects with ALP >1x ULN or >1.5x ULN, and subjects with AST or ALT > 3x ULN post-baseline accompanied by concurrently elevated total bilirubin >1.5x ULN or > 2x ULN in the active treatment period will be presented. A listing of subjects with potential serious hepatotoxicity will be provided with the concurrent records labeled. Concurrent is defined as on the same day.

5.4.4 C-SSRS Data

Suicidal ideation events include 11 categories: (1) Wish To Be Dead, (2) Non-Specific Active Thoughts, (3) Active Thoughts Without Intent To Act, (4) Active Thoughts With Some Intent—No Plan, (5) Active Thoughts with Plan and Intent. Suicidal behavior events include: (6) Preparatory acts or behavior, (7) Aborted Attempt, (8) Interrupted Attempt, (9) Actual Attempt, (10) Suicidal behavior, (11) Completed Suicide. Another “Yes/No” question of whether the subject has engaged in non-suicidal self-injurious behavior is also collected.

The analysis will be based on the safety population. Number of subjects in each ideation category at any post-baseline visit, number of subjects in each suicidal behavior category at any post-baseline visit, number of subjects with at least one suicidal ideation or behavior event at any post-baseline visit, and subjects who have engaged in non-suicidal self-injurious behavior at any post-baseline visit will be summarized in the placebo-controlled and LTE active treatment period. A listing of C-SSRS data by subjects will be provided.

5.4.5 ECG Data

5.4.5.1 ECG analyses

Actual values of numeric ECG parameters will be summarized by visit. number of evaluable subjects, mean, standard deviation, 25% and 75% quartiles, min and max values will be presented at each visit for placebo-controlled and LTE period active treatment period. Similarly change from baseline values will be summarized in the same fashion for placebo-controlled and LTE active treatment period.

Summary of ECGs (number of normal, <abnormal, not adverse event>, or <abnormal, adverse event>) at scheduled visits will be presented by the LTE treatment group through the placebo-controlled and LTE active treatment period.

CONFIDENTIAL
The information contained herein may not be used, disclosed, or published without the written consent of Biogen MA Inc.

Shift table from normal or unknown ECG at baseline to ever abnormal ((<abnormal, not adverse event>, or <abnormal, adverse event>) post-baseline ECG will be summarized for the placebo-controlled and LTE active treatment group. The worst post-baseline record of each subject is selected.

For the above mentioned ECG analyses, for early start subjects, the placebo-controlled baseline will be used while for late start subjects, the LTE baseline will be used.

In addition, PR and QTcF values will be summarized. Please see the detail in the SAP for placebo-controlled period.

Visit windows for by visit summaries

For ECG data that are summarized by visit, assessment from all scheduled visits, EOT visit, EOS visit and unscheduled visits will be mapped to an appropriate analysis visit using a windowing scheme (Appendix I).

5.4.6 Vital Sign Data

5.4.6.1 Vital Sign Data Analyses

Vital sign parameters include temperature, diastolic blood pressure, systolic blood pressure, heart rate, and respiration rate. The descriptive statistics for actual values will be summarized by all the visits in the combined placebo-controlled and LTE active treatment period. The lines of mean vital sign over time by LTE treatment group will be graphed in the combined placebo-controlled and LTE active treatment period.

Summary of change from baseline including number of subjects, mean, standard deviation, median, minimum, and maximum values will be summarized in the placebo-controlled and LTE active treatment period. Placebo-controlled baseline will be used for early start subjects and LTE baseline will be used for late start analysis.

A by-patient listing for all vital sign parameters in the combined placebo-controlled and LTE period will also be presented.

The analysis of vital signs will also focus on the incidence of clinically relevant outliers.

For details of criteria to assess potential clinically relevant outliers in vital sign, refer to section 5.4.6 in the SAP for the placebo-controlled period. The number of clinically relevant outliers determined by each criterion will be summarized across combined placebo-controlled and LTE active treatment period by treatment group.

Visit windows for by visit summaries

For vital sign data that are summarized by visit, assessment from all scheduled visits, EOT visit, EOS visit and unscheduled visits will be mapped to an appropriate analysis visit using a windowing scheme (Appendix I).

CONFIDENTIAL
The information contained herein may not be used, disclosed, or published without the written consent of Biogen MA Inc.

5.4.7 Physical Examination

Clinically significant findings during physical examinations will be recorded as AEs. Abnormal findings from physical examination will be included in AE listing..

5.4.8 Neurological Examination

Clinically significant findings during neurological examinations will be recorded as AEs. Abnormal findings from neurological examination will be included in AE listing..

5.4.9 Safety MRI Data

The following MRI data will be summarized by LTE treatment group using safety MRI population for placebo-controlled and LTE active treatment period:

- Number of subjects with new or worsening of vasogenic edema (VE) at post-baseline
  - New VE or questionable VE is considered as subjects who has a VE which is worsening as post-baseline or for the following:
    - The subject has answered “Not-Applicable” at any post-baseline visit for question: VE on this MRI as compared to previous MRI
    - The subject has answered “VE present and increased in size” at any post-baseline visit for question: VE on this MRI as compared to previous MRI
  - Worsening of VE from baseline is defined as any severity increase compared to baseline. The following sequence indicates the increase of the severity:

    No VE present < Questionable presence of VE (Mild Severity) < Questionable presence of VE (Moderate Severity) < Questionable presence of VE (Severe Severity) < VE present (Mild Severity) < VE present (Moderate Severity) < VE present (Severe Severity)

- Number of subjects with new microhemorrhages (mH) findings at post-baseline
  - New mH is define as subjects who has mH increased in number and/or size at any post-baseline comparing to the baseline. If a subject has no mHs at baseline, then new mH is defined as any initial identification at any post-baseline visit.
- Number of subjects with macrohemorrhages (MA) (>1cm) at post-baseline
- Number of subjects with new superficial siderosis (SS) (>1cm^3) at post-baseline
- Number of subjects with new SS (≤1cm^3) at post-baseline

5.4.10 COVID-19 Related Safety Analysis

All AE tables due to COVID-19 will be summarized for LTE period and PC and LTE active treatment period. Please refer to the placebo-controlled SAP for more details.

5.5 Pharmacokinetics Analysis

The Serum PK evaluable set as defined in Section 3.1, will be used for the description of the serum concentration data, and for the estimation of serum PK parameters. The CSF PK evaluable set as defined in Section 3.1, will be used for the description of CSF concentration data. PK analysis will be conducted with serum and CSF concentrations of BIIB092 by visit and dose group. Subjects who receive BIIB092 125mg once every 4 weeks will be analyzed separately from those who receive 375mg once every 12 weeks. The summaries and listing will be done using the combined placebo-controlled and LTE active treatment period.

Atypical drug concentrations (e.g., very low or very high) will be excluded from the analysis, if no apparent explanation exists. Concentration observations will also be removed from the data set if

i) corresponding dosing or sampling times are missing or cannot be reconstructed

ii) large deviations between actual administered dose and nominal dose exists

iii) large deviations between scheduled and actual sampling days or times exists

iv) large deviations between actual and nominal dose administration time exists.

All deletions of data points will be appropriately documented.

5.5.1 Serum and CSF PK Concentration Profile

Individual serum and CSF concentrations will be listed for BIIB092. Concentrations below the lower limit of quantification (LLOQ) will be indicated by "BLQ". Differences between scheduled and actual sampling times will be listed for these subjects, along with the percentage differences between nominal and actual dose amount. Additional listings may be generated as deemed necessary.

Descriptive statistics (N, arithmetic mean, standard deviation, geometric mean, CV, median, minimum, and maximum), will also be used to summarize serum PK and CSF concentrations of BIIB092 by visit/scheduled time points and dose group. For the purpose of calculating typical descriptive statistics (n, mean, SD, %CV, geometric mean, geometric %CV, median, minimum, and maximum) for serum PK, BLQ value on pre-day 1 of study treatment will be set to 0, and all post first dose of study treatment BLQ values will be set to half the LLOQ value. In linear and semi- plots, all BLQ values will be treated as LLOQ/2. Mean CSF concentrations that are BLQ will be presented as BLQ, and the SD and %CV will be reported as not applicable. When summarizing concentrations or PK parameters in serum and CSF, a minimum of 2 values are required to show the arithmetic mean and geometric mean, and at least 3 values are required to show the standard deviation and coefficient of variation (CV).

Serum and CSF concentrations of BIIB092 will be plotted versus time by dose group on both a linear and a logarithmic scale. Additional plots may be included as deemed necessary.

CSF to serum concentration ratio will be computed using pre-infusion concentrations at week 12, 48, 76, 128, 176, and 224. Individual ratios along with descriptive statistics (N, arithmetic mean, standard deviation, geometric mean, CV, median, minimum, and maximum) at 12, 48, 76, 128,

176, and 224 weeks, by dose will be listed. Any BLQ value will be excluded from CSF to serum ratio calculation, and exclusions will be appropriately documented. Individual CSF vs. serum concentrations above LLOQ (for both serum and CSF) will be plotted (scatter plot) by week, color coded by dose.

5.5.1 Serum PK Parameters

The following PK parameters will be listed per visit, as data permits, from serum concentration data:

Parameter	Definition/Calculation	Units
C_{trough}	Observed trough serum BIIB092 concentration collected at end of dosing interval (before next infusion starts)	ug/mL

Individual PK parameter data will be listed. Descriptive statistics (N, mean, standard deviation, CV, median, minimum, and maximum) will be used to summarize the PK parameters by visit and dose. Geometric means (by visit and dose) will also be presented. Box plots and plots of individual C_{trough} over time will be provided.

5.6 Biomarker

The CSF PD evaluable set or CSF PD modified evaluable set as defined in Section 3.1, will be used for the statistical modeling of CSF PD data.

5.6.1 Pharmacodynamics Analysis

The actual, change from baseline, and percentage change from baseline of CSF N-terminal tau will be summarized to evaluate pharmacodynamics effect after multiple IV infusions of BIIB092. For descriptive statistics, BLQ is imputed as LLOQ/2. For individual subject listings, BLQs are listed as BLQ. Summary statistics will be generated showing N, mean, median, standard deviation, Q1, Q3, minimum and maximum results over time by treatment group for CSF N-terminal tau for placebo-control and LTE period.

Mean (± standard error) plots of change from baseline of CSF N-terminal over time by treatment group and mean (± standard error) % change of CSF N-terminal tau over time by treatment group may be presented.

5.6.2 Structural MRI Analysis

No subjects reached week 226, the primary analysis timepoint for structural MRI. Therefore, structural MRI is not reported or statistically analyzed for the LTE period.

5.6.3 Tau PET Analysis

All tau PET analysis will be using tau PET evaluable set defined in Section 3.1

5.6.3.1 By visit summary

The baseline, change and percent change from baseline tau PET SUVR scores will be summarized by LTE treatment groups by visit for placebo-controlled and LTE period for each primary (Braak 1&2, Braak 3&4, and Braak 5&6) and secondary (Posterior composite, Temporal composite, Amyloid composite, Frontal cortex, Parietal cortex, Occipital cortex, Anterior cingulate cortex, Posterior cingulate cortex, Lateral temporal cortex, Inferior temporal cortex, Medial temporal lobe, Lateral temporal lobe) target region and primary reference region (Cerebellum (superior section eroded)). Visit window is defined in Appendix I.

5.7 Anti-Drug Antibody Analysis

5.7.1 Analysis Methods for Anti-Drug Antibody Data

Anti-Drug Antibody (ADA) population will be used to analyze ADA data.

The baseline value is defined as the last available value prior to first dose of placebo or BIIB092 in placebo-controlled period. For subjects with missing baseline assessment, the most conservative approach will be taken and they will be considered negative for ADA at baseline.

For the definitions of treatment emergent positive, persistently positive, and transiently positive, please refer to the placebo-controlled SAP.

Summary table listing the number and percentage of all anti-BIIB092-positive and -negative antibody events by LTE treatment group and visits throughout the placebo-controlled and LTE active treatment period will be displayed.

In addition, a summary table of patients with treatment-emergent, persistent, and transient responses will be presented by LTE treatment group during the placebo-controlled and LTE active treatment period. A listing of anti-BIIB092 antibody results will also be provided.

Visit windows for by visit summaries

For ADA data that are summarized by visit, assessment from all scheduled visits, EOT visit, and unscheduled visits will be mapped to an appropriate analysis visit using a windowing scheme (Appendix I).

5.8 Additional Exploratory Endpoints

5.8.1 Health Outcomes

The FAS will be used for the analysis of health outcomes data for LTE period. The result will be listed by LTE treatment group for placebo-controlled and LTE period.

CONFIDENTIAL
The information contained herein may not be used, disclosed, or published without the written consent of Biogen MA Inc.

6 Appendix I: Visit Window Mapping

For data that are summarized by visit and longitudinal analysis, assessment from all scheduled visits including EOT visit and EOS visit, and all unscheduled visits will be mapped to an appropriate analysis visit using a windowing scheme. Analysis visit windows are defined in [Table 1 - Table 12] for different endpoints.

If there are 2 or more assessments from visits other than EOT or EOS visits mapped to the same analysis visit for a subject, the assessment that is closest to the target visit day will be used for analysis. If there are 2 or more assessments from visits other than EOT or EOS visits mapped to the same analysis visit with the same distance from the target visit day, then select the later one(s) for the analysis. If there are 2 or more assessments from visits other than EOT or EOS visits mapped to the same analysis visit and on the same day, use the average value for quantitative parameters and the worst value for qualitative parameters for analysis.

Table 1. Visit Windows for Efficacy Endpoints

Efficacy Endpoint	Analysis visit	Target visit day	Analysis visit window
CDR, NPI-10, ADAS-Cog13, FAQ, and ADCS-ADL	Baseline	1	Most recent non-missing pre-dose value
	Week 24	169	[2, 267]
	Week 52	365	[268, 456]
	Week 78	547	[457, the end day of the placebo-controlled period [1]]
	Week 104	729	[one day after the start day of the LTE period[2] , 813]
	Week 128	897	[814, 981]
	Week 152	1065	[982, 1149]
	Week 176	1233	[1150, 1317]
	Week 200	1401	[1318, 1492]
	Week 226	1582	≥1493
ISLT, DKEFS, DSST, Trails A,	Baseline800-	1	Most recent non-missing pre-dose value
	Week 12	85	[2, 141]
	Week 28	197	[142, 239]
	Week 40	281	[240, 337]
	Week 56	393	[338, 435]
	Week 68	477	[436, 512]
	Week 78	547	[513, the end day of the placebo-controlled period [1]]
	Week 108	757	[one day after the start day of the LTE period[2], 841]

Efficacy Endpoint	Analysis visit	Target visit day	Analysis visit window
	Week 132	925	[842, 1009]
	Week 156	1093	[1010, 1261]
	Week 204	1429	[1262, 1506]
	Week 226	1582	≥1507
eCog	Baseline	1	Most recent non-missing pre-dose value
	Week 28	197	[2, 295]
	Week 56	393	[296, 470]
	Week 78	547	[471, the end day of the placebo-controlled period [1]]
	Week 108	757	[one day after the start day of the LTE period[2], 841]
	Week 132	925	[842, 1009]
	Week 156	1093	[1010, 1261]
	Week 204	1429	[1262, 1506]
	Week 226	1582	≥1507
MMSE	Baseline	1	Most recent non-missing pre-dose value
	Week 12	85	[2, 127]
	Week 24	169	[128, 225]
	Week 40	281	[226, 323]
	Week 52	365	[324, 421]
	Week 68	477	[422, 512]
	Week 78	547	[513, the end day of the placebo-controlled period[1]]
	Week 104	729	[one day after the start day of the LTE period[2], 813]
	Week 128	897	[814, 981]
	Week 152	1065	[982, 1149]
	Week 176	1233	[1150, 1317]
	Week 200	1401	[1318, 1492]
	Week 226	1582	≥1493
	1 The end day of the placebo-controlled period is the last day on or before the first infusion in LTE for subjects who enter LTE and is the last day in study for subjects who do not enter LTE.<br>2 The start day of the LTE period is the day of the first infusion in the LTE period.		

CONFIDENTIAL
The information contained herein may not be used, disclosed, or published without the written consent of Biogen MA Inc.

Table 2. Visit Windows for Laboratory by Visit Summaries using Placebo-Controlled Baseline

Analysis visit	Target visit day	Analysis visit window
Baseline	1	Most recent non-missing pre-dose value
Week 12	85	[2, 127]
Week 24	169	[128, 253]
Week 48	337	[254, 421]
Week 72	505	[422, 526]
Week 78	547	[527, 630* or the end day of the placebo-controlled period[1]]
Week 90*	631	[631, the last day in the study]
Week 100	701	[the start day of the LTE period[2], 785]
Week 124	869	[786, 953]
Week 148	1037	[954, 1121]
Week 172	1205	[1122, 1289]
Week 196	1373	[1290, 1457]
Week 220	1541	[1458, 1562]
Week 226	1582	[1563, 1625]
Week 238	1667	≥1626
* Applicable only for the subjects who do not go to LTE. They are expected to have week 90 follow-up assessment.<br>1 The end day of the placebo-controlled period is the last day before the first infusion in LTE for subjects who enter LTE and is the last day in study for subjects who do not enter LTE.<br>2 The start day of the LTE period is the day of the first infusion in the LTE period.		

Table 3. Visit Windows for Laboratory by Visit Summaries using LTE Baseline

Analysis visit	Target visit day	Analysis visit window	Protocol visit
Baseline	1	Most recent non-missing value prior to the first infusion in LTE	
Week 24	169	[2, 253]	Week 100
Week 48	337	[254, 421]	Week 124
Week 72	505	[422, 526]	Week 148
Week 100	701	[527, 785]	Week 172
Week 124	869	[786, 953]	Week 196
Week 148	1037	[954, 1121]	Week 220 and Week 226
Week 172	1205	≥1122	Week 238
Note: Study day and analysis visit window are derived based on the first day of study drug in LTE.			

Table 4. Visit Window for Coagulation Panel by Visit Summaries using Placebo-Controlled Baseline

Analysis visit	Target visit day	Analysis visit window
Baseline	1	Most recent non-missing pre-dose value
Week 8	57	[2,183]
Week 44	309	[184, 407]
Week 72	505	[408, the end day of the placebo-controlled period[1]]
Week 124	869	[the start day of the LTE period[2], 1037]
Week 172	1205	[1038, 1373]
Week 220	1541	> 1373

1 The end day of the placebo-controlled period is the last day before the first infusion in LTE for subjects who enter LTE and is the last day in study for subjects who do not enter LTE.
2 The start day of the LTE period is the day of the first infusion in the LTE period.

Table 5. Visit Window for Coagulation Panel by Visit Summaries using LTE Baseline

Analysis visit	Target visit day	Analysis visit window	Protocol visit
Baseline	Most recent non-missing value	Most recent non-missing value prior to the first infusion in LTE	Week 72
Week 44	309	[2, 477]	Week 124
Week 72	645	[478, 813]	Week 172
Week 124	981	> 813	Week 220

Table 6. Visit Windows for ECG by Visit Summaries for Placebo-Controlled and LTE Period using Placebo-Controlled Baseline

Analysis visit	Target visit day	Analysis visit window
Baseline	1	Most recent non-missing pre-dose value
D1 Post dose	1	Day1: 15 min post dose or 1 hour post dose
Week 12	85	[2, 127]
Week 24	169	[128, 253]
Week 48	337	[254, 421]
Week 72	505	[422, 526]
Week 78	547	[527, 630* or the end day of the placebo-controlled period[1]]
Week 90*	631	[631, the last day in the study]
Week 104	729	[the start day of the LTE period[2], 813]
Week 128	897	[814, 981]
Week 152	1065	[982, 1149]
Week 176	1233	[1150, 1317]
Week 200	1401	[1318, 1492]
Week 226	1582	[1493, 1625]
Week 238	1667	[1626, the end day of the LTE period]

* Applicable only for the subjects who do not go to LTE. They are expected to have week 90 follow-up assessment.
1 The end day of the placebo-controlled period is the last day before the first infusion in LTE for subjects who enter LTE and is the last day in study for subjects who do not enter LTE.
2 The start day of the LTE period is the day of the first infusion in the LTE period.

Table 7. Visit Windows for ECG by Visit Summaries using LTE Baseline

Analysis visit	Target visit day	Analysis visit window	Protocol visit
Baseline	Most recent non-missing value prior to the first infusion in LTE	Most recent non-missing value prior to the first infusion in LTE	Week 78

Analysis visit	Target visit day	Analysis visit window	Protocol visit
Week 24	169	[the start day of the LTE period, 253]	Week 104
Week 48	337	[254, 421]	Week 128
Week 72	505	[422, 589]	Week 152
Week 104	673	[590, 757]	Week 176
Week 128	841	[758, 932]	Week 200
Week 152	1023	[933, 1065]	Week 226
Week 176	1107	>1065	Week 238
Day 1 is the first infusion day in LTE period			

Table 8. Visit Windows for Vital Sign by Visit Summaries using Placebo-Controlled Baseline

Analysis visit	Target visit day	Analysis visit window
Baseline	1	Most recent non-missing pre-dose value
D1 Post dose	1	Day 1 post dose infusion (if more than one Day 1 post dose results available, the average of the available results will be used)
Week 4– Pre dose	29	[2, 43] (Pre-dose assessment)
Week 4- Post dose	29	[2, 43] (Post dose assessment)
Week 8– Pre dose	57	[44, 71] (Pre-dose assessment)
Week 8- Post dose	57	[44, 71] (Post dose assessment)
Week 12– Pre dose	85	[72, 99] (Pre-dose assessment)
Week 12- Post dose	85	[72, 99] (Post dose assessment)
Week 16	113	[100, 127]
Week 20	141	[128, 155]
Week 24	169	[156, 183]
Week 28	197	[184, 211]
Week 32	225	[212, 239]
Week 36	253	[240, 267]
Week 40	281	[268, 295]
Week 44	309	[296, 323]
Week 48	337	[324, 351]
Week 52	365	[352, 379]
Week 56	393	[380, 407]
Week 60	421	[408, 435]
Week 64	449	[436, 463]
Week 68	477	[464, 491]
Week 72	505	[492, 519]
Week 76	533	[520, 540]
Week 78	547	[541, 589* or the end day of the placebo-controlled period[1]]
Week 90*	631	[590, the last day in the study]
Week 80	561	[the start day of the LTE period[2], 575]
Week 84	589	[576, 603]
Week 88	617	[604, 631]
Week 92	645	[632, 659]
Week 96	673	[660, 687]

Analysis visit	Target visit day	Analysis visit window
Week 100	701	[688, 715]
Week 104	729	[716, 743]
Week 108	757	[744, 771]
Week 112	785	[772, 799]
Week 116	813	[800, 827]
Week 120	841	[828, 855]
Week 124	869	[856, 883]
Week 128	897	[884, 911]
Week 132	925	[912, 939]
Week 136	953	[940, 967]
Week 140	981	[968, 995]
Week 144	1009	[996, 1023]
Week 148	1037	[1024, 1051]
Week 152	1065	[1052, 1079]
Week 156	1093	[1080, 1107]
Week 160	1121	[1108, 1135]
Week 164	1149	[1136, 1163]
Week 168	1177	[1164, 1191]
Week 172	1205	[1192, 1219]
Week 176	1233	[1220, 1247]
Week 180	1261	[1248, 1275]
Week 184	1289	[1276, 1303]
Week 188	1317	[1304, 1331]
Week 192	1345	[1332, 1359]
Week 196	1373	[1360, 1387]
Week 200	1401	[1388, 1415]
Week 204	1429	[1416, 1443]
Week 208	1457	[1444, 1471]
Week 212	1485	[1472, 1499]
Week 216	1513	[1500, 1527]
Week 220	1541	[1528, 1555]
Week 224	1569	[1556, 1576]
Week 226	1582	[1577, 1625]
Week 238	1667	[1626, the end of the LTE period]
* Applicable only for the subjects who do not go to LTE. They are expected to have week 90 follow-up assessment.<br>1 The end day of the placebo-controlled period is the last day before the first infusion in LTE for subjects who enter LTE, and is the last day in study for subjects who do not enter LTE.<br>2 The start day of the LTE period is the day of the first infusion in the LTE period.		

Table 9. Visit Windows for Vital Sign by Visit Summaries using LTE baseline

Analysis visit	Target visit day	Analysis visit window	Protocol visit
LTE Baseline	1	Most recent non-missing value prior to the first infusion in LTE	Week 80 pre dose
D1 Post dose	1	Day 1 post dose infusion (if more than one Day 1 post dose results available, the average of the available results will be used)	Week 80 post dose
Week 4– Pre dose	29	[2, 43] (Pre-dose assessment)	Week 84 (Pre-dose assessment)
Week 4-- Post dose	29	[2, 43] (Post dose assessment)	Week 84 (Post dose assessment)
Week 8– Pre dose	57	[44, 71] (Pre-dose assessment)	Week 88 (Pre-dose assessment)
Week 8- Post dose	57	[44, 71] (Post dose assessment)	Week 88 (Post dose assessment)
Week 12– Pre dose	85	[72, 99] (Pre-dose assessment)	Week 92 (Pre-dose assessment)
Week 12- Post dose	85	[72, 99] (Post dose assessment)	Week 92 (Post dose assessment)
Week 16	113	[100, 127]	Week 96
Week 20	141	[128, 155]	Week 100
Week 24	169	[156, 183]	Week 104
Week 28	197	[184, 211]	Week 108
Week 32	225	[212, 239]	Week 112
Week 36	253	[240, 267]	Week 116
Week 40	281	[268, 295]	Week 120
Week 44	309	[296, 323]	Week 124
Week 48	337	[324, 351]	Week 128
Week 52	365	[352, 379]	Week 132
Week 56	393	[380, 407]	Week 136
Week 60	421	[408, 435]	Week 140
Week 64	449	[436, 463]	Week 144
Week 68	477	[464, 491]	Week 148
Week 72	505	[492, 519]	Week 152
Week 76	533	[520, 547]	Week 156
Week 80	561	[548, 575]	Week 160
Week 84	589	[576, 603]	Week 164
Week 88	617	[604, 631]	Week 168
Week 92	645	[632, 659]	Week 172
Week 96	673	[660, 687]	Week 176
Week 100	701	[688, 715]	Week 180
Week 104	729	[716, 743]	Week 184
Week 108	757	[744, 771]	Week 188
Week 112	785	[772, 799]	Week 192
Week 116	813	[800, 827]	Week 196
Week 120	841	[828, 855]	Week 200
Week 124	869	[856, 883]	Week 204
Week 128	897	[884, 911]	Week 208
Week 132	925	[912, 939]	Week 212
Week 136	953	[940, 967]	Week 216
Week 140	981	[968, 995]	Week 220
Week 144	1009	[996, 1023]	Week 224
Week 148	1037	[1024, 1051]	Week 226
Week 152	1065	[1052, the end day of the LTE period]	Week 238

CONFIDENTIAL
The information contained herein may not be used, disclosed, or published without the written consent of Biogen MA Inc.

Table 10. Visit Windows for ADA data using Placebo controlled baseline

Analysis visit	Target visit day	Analysis visit window
Baseline	1	See baseline definition in Section 3.7.1
Week 4	29	[2, 99]
Week 24	169	[100, 253]
Week 48	337	[254, 435]
Week 76	533	[436, the end day of the placebo-controlled period[1]]
Week 80	561	[the start day of the LTE period[2], 645]
Week 104	729	[646, 771]
Week 116	813	[772, 953]
Week 156	1093	[954, 1163]
Week 176	1233	[1164, 1303]
Week 196	1373	[1304, 1471]
Week 224	1569	[1472, 1618]
Week 238	1667	≥1619
1 The end day of the placebo-controlled period is the last day before the first infusion in LTE for subjects who enter LTE, and is the last day in study for subjects who do not enter LTE.<br>2 The start day of the LTE period is the day of the first infusion in the LTE period.		

Table 11. Visit Windows for ADA data using LTE Baseline

Analysis visit	Target visit day	Analysis visit window	Protocol visit
Baseline	Most recent non-missing value prior to the first infusion in LTE	Most recent non-missing value prior to the first infusion in LTE	Week 80
Week 24	169	[2, 253]	Week 104
Week 48	337	[254, 435]	Week 116
Week 76	533	[436, 631]	Week 156
Week 104	729	[632, 771]	Week 176
Week 116	813	[772, 953]	Week 196
Week 156	1093	[954, 1163]	Week 224
Week 176	1233	>1163	Week 238
Day 1 is the first infusion day in LTE period			

Table 12. Visit Windows for tau PET data

Analysis visit *	Target visit day	Analysis visit window
Baseline	1	Most recent non-missing pre-dose value
Week 52	364	[120, 456]
Week 78	547	[457, 727*]
Week 152	1065	[728, 1324]
Week 226	1583	>1325
* 180 days after Week 78 target date.		

Table 13. Visit Windows for C-SSRS data using Placebo controlled baseline

Analysis visit	Target visit day	Analysis visit window
Baseline	1	Most recent non-missing pre-dose value
Week 24	169	[2, 267]
Week 52	365	[268, 456]
Week 78	547	[457, the last day of the PC period[1]]
Week 104	729	[the start day of the LTE period[2], 813]
Week 128	897	[814, 981]
Week 152	1065	[982, 1149]
Week 176	1233	[1150, 1317]
Week 200	1401	[1318, 1492]
Week 226	1583	>1492

1. The end day of the placebo-controlled period is the last day before the first infusion in LTE, and is the last day in study for subjects who do not enter LTE.
2. The start day of the LTE period is the day of first infusion in LTE period.

Table 14. Visit Windows for C-SSRS by Visit Summaries using LTE Baseline

Analysis visit	Target visit day	Analysis visit window	Protocol visit
Baseline	Most recent non-missing value prior to the first infusion in LTE	Most recent non-missing value prior to the first infusion in LTE	Week 78
Week 24	169	[the start day of the LTE period, 253]	Week 104
Week 52	337	[254, 421]	Week 128
Week 78	505	[422, 589]	Week 152
Week 104	673	[590, 757]	Week 176
Week 128	841	[758, 932]	Week 200
Week 152	1023	> 932	Week 226

Day 1 is the first infusion day in LTE period

Table 15. Visit Windows for safety MRI data with placebo-controlled baseline

Analysis visit	Target visit day	Analysis visit window
Baseline	1	Most recent non-missing pre-dose value
Week 28	197	[90, 281]
Week 52	365	[282, 456]
Week 78	547	[457, the last day of the PC period[1]]
Week 104	729	[the start day of LTE period[2], 911]
Week 156	1093	[912, 1338]
Week 226	1583	≥ 1339

1. The end day of the placebo-controlled period is the last day before the first infusion in LTE,and is the last day in study for subjects who do not enter LTE.
2. The start day of the LTE period is the day of first infusion in LTE period.

Table 16. Visit Windows for safety MRI data with LTE baseline

CONFIDENTIAL
The information contained herein may not be used, disclosed, or published without the written consent of Biogen MA Inc.

**Analysis visit**	**Target visit day**	**Analysis visit window**	**Protocol specified visit**
Baseline	1	Most recent non-missing pre-dose value	Week 78
Week 28	169	[2, 351]	Week 104
Week 52	533	[352, 778]	Week 156
Week 78	1023	>778	Week 226

Reference

van Ginkel, J. R., Sijtsma, K., van der Ark, L. A., & Vermunt, J. K. (2010). Incidence of missing item scores in personality measurement, and simple item-score imputation. Methodology: European Journal of Research Methods for the Behavioral and Social Sciences, 6(1), 17-30.

Wessels, A.M., Andersen, S.W., Dowsett, S.A., and Siemers, E.R. (2018), "The integrated Alzheimer's disease rating scale (iADRS) findings from the expedition3 trial", Journal of Prevention of Alzhermer's Disease, 2018(5), 134-136.

Wessels, A.M., Siemers, E.R., Yu, P., Andersen, S.W., Holdridge K.C., Sims, J.R., Sundell, K., Stern Y., Rentz, D.M., Dubois, B., Jones, R.W., Cummings, J., and Aisen, P.S. (2015), "A combined measure of cognition and function for clinical trials: the integrated Alzhermer's disease rating scale (iADRS)", Journal of Prevention of Alzheimer's Disease.

Judith Jaeger, PhD, Clint Hagen, MS, Henrik Loft, PhD. Cognitive Endpoints for Early Alzheimer's Disease Trials: Development of the Early AD/ MCI Alzheimer's Cognitive Composite (EMACC). ISCTM 2018

Jinping Wang, Veronika Logovinsky, Suzanne B Hendrix. ADCOMS: a composite clinical outcome for prodromal Alzheimer's disease trials. J Neurol Neurosurg Psychiatry 2016;87:993–999

Rubin, D. B. (1976) "Inference and missing data." Biometrika, 63(3), 581-592.
