# Supplementary material for: TANGO: a placebo-controlled randomized phase 2 study of efficacy and safety of the anti-tau monoclonal antibody gosuranemab in early Alzheimer’s disease
Source: Nat Aging. 2023 Nov 27;3(12):1591–601. doi: 10.1038/s43587-023-00523-w (PMC10724064; doi:10.1038/s43587-023-00523-w)
Supplement: Supplementary file 2 — Reporting Summary [file 43587_2023_523_MOESM2_ESM.pdf]

nature portfolio

Corresponding author(s): Melanie Shulman

Last updated by author(s): September 15, 2023

Reporting Summary

Nature Portfolio wishes to improve the reproducibility of the work that we publish. This form provides structure for consistency and transparency in reporting. For further information on Nature Portfolio policies, see our Editorial Policies and the Editorial Policy Checklist.

Please do not complete any field with "not applicable" or n/a. Refer to the help text for what text to use if an item is not relevant to your study.
For final submission: please carefully check your responses for accuracy; you will not be able to make changes later.

Statistics

For all statistical analyses, confirm that the following items are present in the figure legend, table legend, main text, or Methods section.

n/a	Confirmed	
☐	☒	The exact sample size (*n*) for each experimental group/condition, given as a discrete number and unit of measurement
☐	☒	A statement on whether measurements were taken from distinct samples or whether the same sample was measured repeatedly
☐	☒	The statistical test(s) used AND whether they are one- or two-sided<br>*Only common tests should be described solely by name; describe more complex techniques in the Methods section.*
☐	☒	A description of all covariates tested
☐	☒	A description of any assumptions or corrections, such as tests of normality and adjustment for multiple comparisons
☐	☒	A full description of the statistical parameters including central tendency (e.g. means) or other basic estimates (e.g. regression coefficient) AND variation (e.g. standard deviation) or associated estimates of uncertainty (e.g. confidence intervals)
☐	☒	For null hypothesis testing, the test statistic (e.g. *F*, *t*, *r*) with confidence intervals, effect sizes, degrees of freedom and *P* value noted<br>*Give P values as exact values whenever suitable.*
☒	☐	For Bayesian analysis, information on the choice of priors and Markov chain Monte Carlo settings
☒	☐	For hierarchical and complex designs, identification of the appropriate level for tests and full reporting of outcomes
☒	☐	Estimates of effect sizes (e.g. Cohen's *d*, Pearson's *r*), indicating how they were calculated

Our web collection on statistics for biologists contains articles on many of the points above.

Software and code

Policy information about availability of computer code

Data collection	Rave EDC
Data analysis	SAS, version 9.4

For manuscripts utilizing custom algorithms or software that are central to the research but not yet described in published literature, software must be made available to editors and reviewers. We strongly encourage code deposition in a community repository (e.g. GitHub). See the Nature Portfolio guidelines for submitting code & software for further information.

Data

Policy information about availability of data

All manuscripts must include a data availability statement. This statement should provide the following information, where applicable:

- Accession codes, unique identifiers, or web links for publicly available datasets
- A description of any restrictions on data availability
- For clinical datasets or third party data, please ensure that the statement adheres to our policy

Trial results are publicly available at ClinicalTrials.gov (Study Record | ClinicalTrials.gov) and the EudraCT website (EudraCT Number 2017-002901-37 - Clinical trial results - EU Clinical Trials Register). Individual participant data collected during the trial may be shared after anonymization and on approval of the research proposals in accordance with internal policies and procedures. Biogen commits to sharing patient-level data, study-level data, CSRs, and protocols with qualified scientific researchers who provide a methodologically sound proposal. Biogen reviews all data requests internally based on the review criteria and in accordance with our Clinical Trial Transparency and Data Sharing Policy, which is available at https://www.biogentrialtransparency.com. De-identified data and documents will be shared under agreements that further protect against participant re-identification. To request access to data, please visit https://vivli.org/.

Research involving human participants, their data, or biological material

Policy information about studies with human participants or human data. See also policy information about sex, gender (identity/presentation), and sexual orientation and race, ethnicity and racism.

Reporting on sex and gender	Sex is reported as part of baseline demographics.
Reporting on race, ethnicity, or other socially relevant groupings	Race and ethnicity were collected at part of baseline demographic information in order to monitor the diversity of the participants and better categorize treatment effect in different subgroups. Race and ethnicity were based on participants' self-reported information.
Population characteristics	TANGO included participants aged 50 to 80 years who had exhibited progressive decline in memory function for more than 6 months before screening and were diagnosed with either MCI due to AD or mild AD disease dementia according to National Institute on Aging–Alzheimer's Association criteria. Participants must have demonstrated cognitive impairment at the time of screening, defined by an International Shopping List Test Immediate Recall or International Shopping List Test Delayed Recall score 1 SD below the age-adjusted normative mean, a Clinical Dementia Rating (CDR) global score of 0.5 (for MCI due to AD) or 0.5 or 1.0 (for mild AD dementia), a CDR Memory Box score of at least 0.5, and a Mini-Mental State Exam (MMSE) score between 22 to 30 (inclusive). Participants must also have demonstrated evidence of amyloid pathology, confirmed by amyloid PET scan (visual read) or CSF testing. Participants were randomized to one of four groups: placebo, low-dose gosuranemab (125 mg q4w subgroup or 375 mg q12w subgroup), intermediate-dose gosuranemab (600 mg q4w), or high-dose gosuranemab (2000 mg q4w). Across groups: mean age ranged from 69.4 to 70.4; the percentage of females ranged from 44.8 to 52.3%; the percentage of ApoE4 carriers ranged from 60.3 to 74.8%; the percentage of participants at the clinical stage of MCI due to Alzheimer's disease ranged from 43.1 to 53.4%.
Recruitment	Participants were recruited through direct outreach by TANGO sites in 9 countries across US, EU, and Asia Pacific region. The selected TANGO sites were a mix of academic and clinical establishments experienced in clinical studies and had proven records in AD trials. Patient funnel included patient database within selected sites, referrals, memory awareness event/ community outreach. Recruitment vendors to support sites were carefully selected to service a wide-ranging population. Patient selection bias could not be entirely ruled out; however, impacts to results were not expected.
Ethics oversight	This study was conducted in accordance with the Declaration of Helsinki and all applicable International Council for Harmonisation and Good Clinical Practice guidelines. Investigators were required to obtain ethics committee approval prior to beginning the study. For study sites in the US, the study protocol was approved by Advarra's central institutional review board or by one of the following local ethics committees: BioMed IRB, San Diego, CA; Biomedical Research Alliance of New York, Lake Success, NY; Western Institutional Review Board, Puyallup, WA; UCLA OHRRP, Los Angeles, CA; Tufts Health Sciences Institutional Review Board, Boston, MA; Stanford University Research Compliance Office, Palo Alto, CA; Houston Methodist Institutional Review Board, Houston, TX; Human Investigation Committee, Institutional Review Board – Yale University, New Haven, CT. For sites in other countries, the study protocol was approved within their respective country by the following local institutional review boards or ethics committees: Melbourne Health Human Research Ethics Committee (Australia); Alfred Hospital Ethics Committee (Australia); Eastern Health Research and Ethics Committee (Australia); Austin Health Human Research Ethics Committee (Australia); Comité de Protection des Personnes Ouest I (France); Ethikkommission des Fachbereichs Medizin der Ludwig-Maximilians-Universität München (Germany); Comitato Etico dell'Azienda Ospedaliero Universitaria Policlinico Paolo Giaccone, Palermo (Italy); Comitato Etico Istituzioni Ospedaliere Cattoliche – CEIOC (Italy); Azienda Ospedaliera Universitaria Policlinico Umberto I - Università di Roma La Sapienza (Italy); Comitato Etico IRCCS Ospedale S. Raffaele di Milano (Italy); Comitato Etico per le Sperimentazioni Cliniche della Provincia di Vicenza (Italy); Adachi Kyosai Hospital IRB (Japan); Teikyo University Hospital, Mizonokuchi IRB (Japan); Tokyo Medical University Hospital IRB (Japan); Takeda Hospital Group IRB (Japan); Koseikai Sone Clinic IRB (Japan); National Center for Geriatrics and Gerontology IRB (Japan); Osaka University Hospital IRB (Japan); Bioetyczna przy Okregowej Izbie Lekarskiej w Gdansku (Poland); Hospital Universitari i Politecnic La Fe (Spain); Etikprövningsmyndigheten (Sweden). All participants provided written informed consent prior to participating in any study-related activities. An independent data monitoring committee reviewed safety data on an ongoing basis.

Note that full information on the approval of the study protocol must also be provided in the manuscript.

Field-specific reporting

Please select the one below that is the best fit for your research. If you are not sure, read the appropriate sections before making your selection.

☒ Life sciences ☐ Behavioural & social sciences ☐ Ecological, evolutionary & environmental sciences

For a reference copy of the document with all sections, see nature.com/documents/nr-reporting-summary-flat.pdf

Life sciences study design

All studies must disclose on these points even when the disclosure is negative.

Sample size	Sample size calculation was based on MCP-MOD. A sample size of 528 participants was planned to provide approximately 80% power to detect a dose-response relationship in the change from baseline in CDR-SB (primary efficacy endpoint) at 18 months (week 78), a maximal 40% reduction with the highest gosuranemab dose group compared with the placebo group, and an estimated 20% dropout rate at 18 months (week 78) in this study.
Data exclusions	Data were excluded from longitudinal data analyses following a pre-specified algorithm in the statistical analysis plan. Data collected on all scheduled visits and all unscheduled visits were mapped to an appropriate analysis visit using the windowing scheme. If there were 2 or more assessments available in the same analysis window for a participant, the assessment that was closest to the target visit day was used for analysis.
Replication	Reproducibility of the experimental findings was done via analytical replication. Analyses based on the statistical analysis plan and analysis specifications were produced by a primary statistical programmer. These analyses were reproduced by an independent statistical programmer and reviewed by two statisticians. All attempts at analytical replication were successful.
Randomization	Enrolled participants were randomized 1:1:2:2 to one of four treatment arms: (1) low-dose gosuranemab (participants in this group were subsequently randomized 1:1 to receive either 125 mg once every 4 weeks [q4w] or 375 mg once every 12 weeks [q12]), (2) intermediate-dose gosuranemab (600 mg q4w), (3) high-dose gosuranemab (2000 mg q4w), or (4) placebo (0.9% NaCl q4w).
Blinding	The study was double-blind for the placebo-controlled period and dose-blind for the long-term extension.

Reporting for specific materials, systems and methods

We require information from authors about some types of materials, experimental systems and methods used in many studies. Here, indicate whether each material, system or method listed is relevant to your study. If you are not sure if a list item applies to your research, read the appropriate section before selecting a response.

Materials & experimental systems

n/a	Involved in the study
☒	☐ Antibodies
☒	☐ Eukaryotic cell lines
☒	☐ Palaeontology and archaeology
☒	☐ Animals and other organisms
☐	☒ Clinical data
☒	☐ Dual use research of concern
☒	☐ Plants

Methods

n/a	Involved in the study
☒	☐ ChIP-seq
☒	☐ Flow cytometry
☐	☒ MRI-based neuroimaging

Clinical data

Policy information about clinical studies

All manuscripts should comply with the ICMJE guidelines for publication of clinical research and a completed CONSORT checklist must be included with all submissions.

Clinical trial registration

ClinicalTrials.gov no. NCT03352557
We are including the protocol as part of the submission; protocol and SAP are also available at ClinicalTrials.gov

Study protocol

Data collection

TANGO was conducted at 104 sites in 9 countries. Study visits occurred between May 2018 and August 2021. Data were collected up to the last visit.

Outcomes

The primary outcome measure was safety, assessed by the incidence of AEs and SAEs, during the placebo-controlled period. Safety measures, including incident rates of deaths, TEAEs, SAEs, clinical laboratory data, and physical and neurological examination results, were chosen because they are standard measures for evaluating the safety and tolerability of drugs in development. The secondary outcome measure was CDR-SB, which is a validated tests and widely used tools to assess the cognitive, behavioral, and functional status of patients with AD. Safety and tolerability were pre-defined as primary outcome measures as TANGO was a Phase 2 trial and was the first study in an AD patient population evaluating participants who were administered gosuranemab. CDR-SB is a well-established clinical endpoint with proven regulatory acceptance utilized in early AD trials. The incidence of anti-gosuranemab antibodies were chosen to evaluate the immunogenicity of gosuranemab. The safety measures were assessed at baseline and during the placebo-controlled period. The secondary outcome measure was assessed at baseline and at Week 78. Clinical efficacy assessments were administered by a trained clinician or rater, preferably by a neuropsychologist, a psychometrician or another qualified person who was experienced in the assessment of participants with cognitive deficits. For safety assessments, at each study visit, the Investigator assessed the participant for AEs including SAEs. Determination of whether an abnormal laboratory value, vital sign result, and/or ECG result meets the definition of an AE was made by the Investigator. The primary and secondary measures were prespecified and analyzed according to the Statistical Analysis Plan (SAP), which was finalized before database lock.
