# Supplementary material for: TANGO: a placebo-controlled randomized phase 2 study of efficacy and safety of the anti-tau monoclonal antibody gosuranemab in early Alzheimer’s disease
Source: Nat Aging. 2023 Nov 27;3(12):1591–601. doi: 10.1038/s43587-023-00523-w (PMC10724064; doi:10.1038/s43587-023-00523-w)
Supplement: Supplementary file 3 — ADAS-Cog13 results in the placebo-controlled and LTE periods. Analysis was performed using a mixed model for repeated measures with data from the placebo-controlled and LTE periods. Analyses were two-sided at a 5% significance level. No adjustments were made for multiple comparisons. [file 43587_2023_523_MOESM3_ESM.pdf]

**Supplementary Table 1. ADAS-Cog13 results in the placebo-controlled and LTE periods.**

| | | Placebo (High Dose Late Start after week 78) (N=214) | Low Dose (N=116) | Intermediate Dose (N=106) | High Dose (N=214) |
|---|---|---|---|---|---|
| Week 24 | n | 204 | 109 | 102 | 207 |
| | Adjusted mean change from baseline | 0.77 | 1.60 | 0.83 | 1.16 |
| | 95% CI | (−0.037, 1.577) | (0.597, 2.594) | (−0.193, 1.848) | (0.361, 1.951) |
| | p-value (compared with placebo/ high dose late-start) | N/A | 0.1463 | 0.9212 | 0.4146 |
| Week 52 | n | 174 | 98 | 95 | 179 |
| | Adjusted mean change from baseline | 2.23 | 3.28 | 3.84 | 3.21 |
| | 95% CI | (1.221, 3.236) | (2.006, 4.555) | (2.545, 5.142) | (2.217, 4.207) |
| | p-value (compared with placebo/ high dose late-start) | N/A | 0.1673 | 0.0371 | 0.1252 |
| Week 78 | n | 170 | 96 | 90 | 173 |
| | Adjusted mean change from baseline | 5.83 | 7.54 | 7.58 | 7.50 |
| | 95% CI | (4.602, 7.054) | (5.963, 9.113) | (5.956, 9.195) | (6.292, 8.717) |
| | p-value (compared with placebo/ high dose late-start) | N/A | 0.0765 | 0.0760 | 0.0397 |
| Week 104 (LTE) | n | 126 | 75 | 72 | 132 |
| | Adjusted mean change from baseline | 7.98 | 9.55 | 8.78 | 9.78 |
| | 95% CI | (6.426, 9.533) | (7.542, 11.553) | (6.723, 10.837) | (8.247, 11.307) |
| | p-value (compared with placebo/ high dose late-start) | N/A | 0.2105 | 0.5296 | 0.0907 |

Analysis was performed using mixed model repeated measures with data from the placebo-controlled period and LTE period. Analyses were two-sided at 5% significance level. No adjustments were made for multiple comparisons.
