# Supplementary material for: TANGO: a placebo-controlled randomized phase 2 study of efficacy and safety of the anti-tau monoclonal antibody gosuranemab in early Alzheimer’s disease
Source: Nat Aging. 2023 Nov 27;3(12):1591–601. doi: 10.1038/s43587-023-00523-w (PMC10724064; doi:10.1038/s43587-023-00523-w)
Supplement: Supplementary file 4 — Statistical source data. [file 43587_2023_523_MOESM4_ESM.zip › Figure 1_Source data (1).rtf]

Accounting of subjects - full analysis set: placebo-controlled period	
	
	Placebo
(N=214)
 n (%)	BIIB092
125mg/4wk
(N=58)
 n (%)	BIIB092
375mg/12wk
(N=58)
 n (%)	BIIB092
Low Dose
(N=116)
 n (%)	BIIB092
600mg/4wk
(N=106)
 n (%)	BIIB092
2000mg/4wk
(N=214)
 n (%)	Total
(N=650)
 n (%)	
 	
Number of subjects randomized	214	 58	 58	116	106	218	654	
 	
Number of subjects dosed	214 ( 100)	 58 ( 100)	 58 ( 100)	116 ( 100)	106 ( 100)	214 ( 100)	650 ( 100)	
 	
Number of subjects who completed study treatment	160 (74.8)	 44 (75.9)	 45 (77.6)	 89 (76.7)	 90 (84.9)	168 (78.5)	507 (78.0)	
 	
Number of subjects who completed the study	172 (80.4)	 48 (82.8)	 50 (86.2)	 98 (84.5)	 91 (85.8)	175 (81.8)	536 (82.5)	
 	
Number of subjects who discontinued treatment yet completed the placebo-controlled period	 12 ( 5.6)	  5 ( 8.6)	  5 ( 8.6)	 10 ( 8.6)	  2 ( 1.9)	 11 ( 5.1)	 35 ( 5.4)	
 	

NOTE: 4 subjects were randomised but not dosed are not included in 'full analysis set'.	
	


Accounting of subjects - full analysis set: placebo-controlled period	
	
	Placebo
(N=214)
 n (%)	BIIB092
125mg/4wk
(N=58)
 n (%)	BIIB092
375mg/12wk
(N=58)
 n (%)	BIIB092
Low Dose
(N=116)
 n (%)	BIIB092
600mg/4wk
(N=106)
 n (%)	BIIB092
2000mg/4wk
(N=214)
 n (%)	Total
(N=650)
 n (%)	
 	
Number of subjects who discontinued study drug	 54 (25.2)	 14 (24.1)	 13 (22.4)	 27 (23.3)	 16 (15.1)	 46 (21.5)	143 (22.0)	
  Death	  1 ( 0.5)	  1 ( 1.7)	  0	  1 ( 0.9)	  0	  0	  2 ( 0.3)	
  Adverse event	 11 ( 5.1)	  3 ( 5.2)	  2 ( 3.4)	  5 ( 4.3)	  0	  8 ( 3.7)	 24 ( 3.7)	
  Non-compliance with study drug	  1 ( 0.5)	  0	  0	  0	  0	  0	  1 ( 0.2)	
  Protocol deviation	  1 ( 0.5)	  0	  0	  0	  1 ( 0.9)	  2 ( 0.9)	  4 ( 0.6)	
  Randomized by mistake	  3 ( 1.4)	  0	  0	  0	  1 ( 0.9)	  0	  4 ( 0.6)	
  Site terminated by sponsor	  2 ( 0.9)	  1 ( 1.7)	  1 ( 1.7)	  2 ( 1.7)	  1 ( 0.9)	  2 ( 0.9)	  7 ( 1.1)	
  Withdrawal by subject-study visit burden/scheduling conflicts	  7 ( 3.3)	  0	  1 ( 1.7)	  1 ( 0.9)	  1 ( 0.9)	  5 ( 2.3)	 14 ( 2.2)	
  Withdrawal by subject-concern about study procedures/perceived 
   risks	  1 ( 0.5)	  0	  0	  0	  1 ( 0.9)	  3 ( 1.4)	  5 ( 0.8)	
  Withdrawal by subject-relocation (moving or has moved)	  1 ( 0.5)	  1 ( 1.7)	  0	  1 ( 0.9)	  1 ( 0.9)	  1 ( 0.5)	  4 ( 0.6)	
  Withdrawal by subject-desire for change in treatment (unrelated 
   to safety)	  0	  0	  1 ( 1.7)	  1 ( 0.9)	  0	  1 ( 0.5)	  2 ( 0.3)	
  Withdrawal by subject-other	  6 ( 2.8)	  2 ( 3.4)	  1 ( 1.7)	  3 ( 2.6)	  4 ( 3.8)	  4 ( 1.9)	 17 ( 2.6)	
  Withdrawal by parent/guardian-study visit burden/scheduling 
   conflicts	  1 ( 0.5)	  0	  0	  0	  0	  2 ( 0.9)	  3 ( 0.5)	
  Withdrawal by parent/guardian-concern about study 
   procedures/perceived risks	  1 ( 0.5)	  0	  1 ( 1.7)	  1 ( 0.9)	  0	  1 ( 0.5)	  3 ( 0.5)	
  Withdrawal by parent/guardian-desire for change in treatment 
   (unrelated to safety)	  1 ( 0.5)	  0	  0	  0	  0	  1 ( 0.5)	  2 ( 0.3)	
  Withdrawal by parent/guardian-unable to continue to enable 
   participation due to illness/hospitalization/death	  0	  0	  0	  0	  0	  1 ( 0.5)	  1 ( 0.2)	
  Withdrawal by parent/guardian-other	  0	  1 ( 1.7)	  0	  1 ( 0.9)	  0	  3 ( 1.4)	  4 ( 0.6)	
  Physician decision-unrelated to safety	  2 ( 0.9)	  0	  0	  0	  0	  1 ( 0.5)	  3 ( 0.5)	
  Lost to follow-up	  1 ( 0.5)	  0	  1 ( 1.7)	  1 ( 0.9)	  0	  2 ( 0.9)	  4 ( 0.6)	
  Other	 14 ( 6.5)	  5 ( 8.6)	  5 ( 8.6)	 10 ( 8.6)	  6 ( 5.7)	  9 ( 4.2)	 39 ( 6.0)	
 	

NOTE: 4 subjects were randomised but not dosed are not included in 'full analysis set'.	
	


Accounting of subjects - full analysis set: placebo-controlled period	
Page: 3 of 3	
	Placebo
(N=214)
 n (%)	BIIB092
125mg/4wk
(N=58)
 n (%)	BIIB092
375mg/12wk
(N=58)
 n (%)	BIIB092
Low Dose
(N=116)
 n (%)	BIIB092
600mg/4wk
(N=106)
 n (%)	BIIB092
2000mg/4wk
(N=214)
 n (%)	Total
(N=650)
 n (%)	
 	
Number of subjects who withdrew from study	 42 (19.6)	 10 (17.2)	  8 (13.8)	 18 (15.5)	 15 (14.2)	 39 (18.2)	114 (17.5)	
  Death	  1 ( 0.5)	  1 ( 1.7)	  0	  1 ( 0.9)	  0	  1 ( 0.5)	  3 ( 0.5)	
  Adverse event	 10 ( 4.7)	  1 ( 1.7)	  1 ( 1.7)	  2 ( 1.7)	  0	  5 ( 2.3)	 17 ( 2.6)	
  Progressive disease	  0	  0	  0	  0	  0	  1 ( 0.5)	  1 ( 0.2)	
  Non-compliance with study drug	  1 ( 0.5)	  0	  0	  0	  0	  0	  1 ( 0.2)	
  Protocol deviation	  1 ( 0.5)	  0	  0	  0	  1 ( 0.9)	  2 ( 0.9)	  4 ( 0.6)	
  Randomized by mistake	  2 ( 0.9)	  0	  0	  0	  1 ( 0.9)	  0	  3 ( 0.5)	
  Site terminated by sponsor	  2 ( 0.9)	  1 ( 1.7)	  1 ( 1.7)	  2 ( 1.7)	  1 ( 0.9)	  2 ( 0.9)	  7 ( 1.1)	
  Withdrawal by subject-study visit burden/scheduling conflicts	  7 ( 3.3)	  1 ( 1.7)	  1 ( 1.7)	  2 ( 1.7)	  0	  5 ( 2.3)	 14 ( 2.2)	
  Withdrawal by subject-concern about study procedures/perceived 
   risks	  1 ( 0.5)	  0	  0	  0	  1 ( 0.9)	  2 ( 0.9)	  4 ( 0.6)	
  Withdrawal by subject-relocation (moving or has moved)	  1 ( 0.5)	  1 ( 1.7)	  0	  1 ( 0.9)	  1 ( 0.9)	  1 ( 0.5)	  4 ( 0.6)	
  Withdrawal by subject-desire for change in treatment (unrelated 
   to safety)	  0	  0	  1 ( 1.7)	  1 ( 0.9)	  0	  1 ( 0.5)	  2 ( 0.3)	
  Withdrawal by subject-other	  6 ( 2.8)	  2 ( 3.4)	  2 ( 3.4)	  4 ( 3.4)	  5 ( 4.7)	  7 ( 3.3)	 22 ( 3.4)	
  Withdrawal by parent/guardian-study visit burden/scheduling 
   conflicts	  0	  0	  0	  0	  1 ( 0.9)	  3 ( 1.4)	  4 ( 0.6)	
  Withdrawal by parent/guardian-concern about study 
   procedures/perceived risks	  1 ( 0.5)	  0	  1 ( 1.7)	  1 ( 0.9)	  1 ( 0.9)	  1 ( 0.5)	  4 ( 0.6)	
  Withdrawal by parent/guardian-desire for change in treatment 
   (unrelated to safety)	  1 ( 0.5)	  0	  0	  0	  0	  1 ( 0.5)	  2 ( 0.3)	
  Withdrawal by parent/guardian-unable to continue to enable 
   participation due to illness/hospitalization/death	  0	  0	  0	  0	  0	  1 ( 0.5)	  1 ( 0.2)	
  Withdrawal by parent/guardian-other	  0	  2 ( 3.4)	  0	  2 ( 1.7)	  0	  3 ( 1.4)	  5 ( 0.8)	
  Physician decision-unrelated to safety	  2 ( 0.9)	  0	  0	  0	  0	  1 ( 0.5)	  3 ( 0.5)	
  Lost to follow-up	  1 ( 0.5)	  0	  1 ( 1.7)	  1 ( 0.9)	  0	  2 ( 0.9)	  4 ( 0.6)	
  Other	  5 ( 2.3)	  1 ( 1.7)	  0	  1 ( 0.9)	  3 ( 2.8)	  0	  9 ( 1.4)	
 	

NOTE: 4 subjects were randomised but not dosed are not included in 'full analysis set'.	
