# Supplementary material for: TANGO: a placebo-controlled randomized phase 2 study of efficacy and safety of the anti-tau monoclonal antibody gosuranemab in early Alzheimer’s disease
Source: Nat Aging. 2023 Nov 27;3(12):1591–601. doi: 10.1038/s43587-023-00523-w (PMC10724064; doi:10.1038/s43587-023-00523-w)
Supplement: Supplementary file 5 — Statistical source data. [file 43587_2023_523_MOESM5_ESM.zip › Figure 2_Source data (1).rtf]

Analysis of change from baseline in CDR sum of boxes by MMRM - full analysis set: placebo-controlled period	
	
	Placebo
(N=214)	BIIB092
Low Dose
(N=116)	BIIB092
600mg/4wk
(N=106)	BIIB092
2000mg/4wk
(N=214)	
 	
Baseline					
  n	    214	    116	    106	    214	
  Mean	      3.07	      2.92	      3.24	      3.04	
 	
Change from baseline at Week 24					
  n	    203	    107	    102	    206	
  Adjusted mean	      0.53	      0.61	      0.86	      0.58	
  Standard error	      0.120	      0.150	      0.152	      0.118	
					
					
					
  p-value (compared with Placebo)		      0.6377	      0.0577	      0.7602	
 	
	
	
	
	


Analysis of change from baseline in CDR sum of boxes by MMRM - full analysis set: placebo-controlled period	
	
	Placebo
(N=214)	BIIB092
Low Dose
(N=116)	BIIB092
600mg/4wk
(N=106)	BIIB092
2000mg/4wk
(N=214)	
 	
Change from baseline at Week 52					
  n	    172	     96	     93	    173	
  Adjusted mean	      1.21	      1.40	      1.62	      1.22	
  Standard error	      0.148	      0.188	      0.191	      0.147	
					
					
					
  p-value (compared with Placebo)		      0.3812	      0.0686	      0.9433	
 	
Change from baseline at Week 78					
  n	    170	     98	     91	    174	
  Adjusted mean	      1.85	      2.20	      2.24	      1.85	
  Standard error	      0.188	      0.241	      0.247	      0.185	
					
					
					
					
  p-value (compared with Placebo)		      0.2362	      0.1965	      0.9778	
 	
	
	
	
	
