# Supplementary material for: TANGO: a placebo-controlled randomized phase 2 study of efficacy and safety of the anti-tau monoclonal antibody gosuranemab in early Alzheimer’s disease
Source: Nat Aging. 2023 Nov 27;3(12):1591–601. doi: 10.1038/s43587-023-00523-w (PMC10724064; doi:10.1038/s43587-023-00523-w)
Supplement: Supplementary file 6 — Statistical source data. [file 43587_2023_523_MOESM6_ESM.zip › Figure 3_Source data (1).rtf]

Analysis of change from baseline in CSF N-terminal tau (pg/mL) by MMRM - CSF PD modified evaluable set: placebo-controlled period	
	
	Placebo
(N=76)	BIIB092
125mg/4wk
(N=25)	BIIB092
375mg/12wk
(N=21)	BIIB092
600mg/4wk
(N=43)	BIIB092
2000mg/4wk
(N=74)	
 	
Baseline						
  n	       75	      25	      21	      42	      74	
  Mean	      219.62	     233.24	     233.50	     232.00	     229.74	
 	
Change from baseline at Week 48						
  n	       68	       24	       19	       39	       66	
  Adjusted mean	      -11.12	     -188.10	     -173.66	     -199.16	     -210.99	
  Standard error	        7.013	       11.801	       12.969	        9.195	        6.974	
						
						
						
  p-value (compared with Placebo)		     <0.0001	     <0.0001	     <0.0001	     <0.0001	
 	
	
	
	
	


Analysis of change from baseline in CSF N-terminal tau (pg/mL) by MMRM - CSF PD modified evaluable set: placebo-controlled period	
	
	Placebo
(N=76)	BIIB092
125mg/4wk
(N=25)	BIIB092
375mg/12wk
(N=21)	BIIB092
600mg/4wk
(N=43)	BIIB092
2000mg/4wk
(N=74)	
 	
Change from baseline at Week 76						
  n	       43	       13	       13	       29	       50	
  Adjusted mean	      -19.39	     -191.38	     -187.15	     -199.45	     -212.83	
  Standard error	        6.910	       11.942	       12.449	        8.650	        6.560	
						
						
						
						
  p-value (compared with Placebo)		     <0.0001	     <0.0001	     <0.0001	     <0.0001	
 	
	
	
	
	


Analysis of change from baseline in CSF p-tau (pg/mL) by MMRM- CSF PD modified evaluable set: placebo-controlled period	
	Placebo
(N=76)	BIIB092
125mg/4wk
(N=25)	BIIB092
375mg/12wk
(N=21)	BIIB092
600mg/4wk
(N=43)	BIIB092
2000mg/4wk
(N=74)	
 	
Baseline						
  n	       76	       25	       21	       42	       73	
  Mean	       96.80	      107.32	      110.32	      109.51	      111.45	
 	
Change from baseline at Week 48						
  n	       69	       24	       19	       39	       66	
  Adjusted mean	       -2.76	      -12.26	       -7.94	      -12.24	      -17.22	
  Standard error	        2.515	        4.235	        4.684	        3.324	        2.552	
						
						
						
  p-value (compared with Placebo)		        0.0535	        0.3296	        0.0222	       <0.0001	
 	
	
	
	
	
	


Analysis of change from baseline in CSF p-tau (pg/mL) by MMRM- CSF PD modified evaluable set: placebo-controlled period	
	
	Placebo
(N=76)	BIIB092
125mg/4wk
(N=25)	BIIB092
375mg/12wk
(N=21)	BIIB092
600mg/4wk
(N=43)	BIIB092
2000mg/4wk
(N=74)	
 	
Change from baseline at Week 76						
  n	       48	       12	       13	       29	       48	
  Adjusted mean	       -1.05	      -18.84	      -11.72	      -17.44	      -26.52	
  Standard error	        5.783	       11.242	       11.049	        7.474	        5.790	
						
						
						
						
  p-value (compared with Placebo)		        0.1612	        0.3931	        0.0838	        0.0022	
 	
	
	
	
	
	


Analysis of change from baseline in CSF amyloid beta 1-42 (pg/mL) by MMRM - CSF PD modified evaluable set: placebo-controlled period	
	
	Placebo
(N=76)	BIIB092
125mg/4wk
(N=25)	BIIB092
375mg/12wk
(N=21)	BIIB092
600mg/4wk
(N=43)	BIIB092
2000mg/4wk
(N=74)	
 	
Baseline						
  n	       76	       25	       21	       42	       73	
  Mean	      455.66	      484.44	      507.00	      513.26	      474.29	
 	
Change from baseline at Week 48						
  n	       69	       24	       19	       39	       66	
  Adjusted mean	      -14.18	      -20.48	       18.10	       -2.23	       -0.54	
  Standard error	       10.664	       17.921	       19.879	       14.119	       10.741	
						
						
						
  p-value (compared with Placebo)		        0.7614	        0.1533	        0.4969	        0.3607	
 	
	


Analysis of change from baseline in CSF amyloid beta 1-42 (pg/mL) by MMRM - CSF PD modified evaluable set: placebo-controlled period	
	
	Placebo
(N=76)	BIIB092
125mg/4wk
(N=25)	BIIB092
375mg/12wk
(N=21)	BIIB092
600mg/4wk
(N=43)	BIIB092
2000mg/4wk
(N=74)	
 	
Change from baseline at Week 76						
  n	       48	       12	       13	       29	       47	
  Adjusted mean	      -17.38	      -16.24	       12.66	      -21.07	       -8.99	
  Standard error	       11.829	       22.341	       22.103	       15.204	       11.815	
						
						
						
						
  p-value (compared with Placebo)		        0.9642	        0.2291	        0.8468	        0.6096	
 	


Analysis of change from baseline in CSF total tau (pg/mL) by MMRM - CSF PD modified evaluable set: placebo-controlled period	
	
	Placebo
(N=76)	BIIB092
125mg/4wk
(N=25)	BIIB092
375mg/12wk
(N=21)	BIIB092
600mg/4wk
(N=43)	BIIB092
2000mg/4wk
(N=74)	
 	
Baseline						
  n	       74	       25	       21	       41	       69	
  Mean	      604.69	      702.40	      736.48	      711.63	      629.39	
 	
Change from baseline at Week 48						
  n	       67	       23	       19	       38	       62	
  Adjusted mean	      -13.50	      -69.45	       -2.53	      -83.97	      -96.74	
  Standard error	       15.300	       25.919	       28.113	       20.135	       15.632	
						
						
						
  p-value (compared with Placebo)		         0.0633	         0.7318	         0.0053	         0.0001	
 	
	
	
	
	
	


Analysis of change from baseline in CSF total tau (pg/mL) by MMRM - CSF PD modified evaluable set: placebo-controlled period	
	
	Placebo
(N=76)	BIIB092
125mg/4wk
(N=25)	BIIB092
375mg/12wk
(N=21)	BIIB092
600mg/4wk
(N=43)	BIIB092
2000mg/4wk
(N=74)	
 	
Change from baseline at Week 76						
  n	       47	       12	       13	       28	       45	
  Adjusted mean	       37.21	      -56.14	       -9.41	     -110.57	      -97.46	
  Standard error	       17.764	       33.150	       32.953	       22.871	       17.957	
						
						
						
						
  p-value (compared with Placebo)		         0.0138	         0.2131	        <0.0001	        <0.0001	
 	
	
	
	
	
	
