# Supplementary material for: TANGO: a placebo-controlled randomized phase 2 study of efficacy and safety of the anti-tau monoclonal antibody gosuranemab in early Alzheimer’s disease
Source: Nat Aging. 2023 Nov 27;3(12):1591–601. doi: 10.1038/s43587-023-00523-w (PMC10724064; doi:10.1038/s43587-023-00523-w)
Supplement: Supplementary file 7 — Statistical source data. [file 43587_2023_523_MOESM7_ESM.zip › Figure 4_Source data (1).rtf]

Analysis of change from baseline in tau PET SUVR scores for each target region and the primary reference region by MMRM - tau PET modified evaluable set: placebo-controlled period	
	
Tau PET Braak 1 and 2 Staging SUVR measure (Reference Region = Cerebellum Superior Cropped)	
	Placebo
(N=98)	BIIB092
Low Dose
(N=53)	BIIB092
600mg/4wk
(N=51)	BIIB092
2000mg/4wk
(N=115)	
 	
Baseline					
  n	      98	      52	      51	     112	
  Mean	       1.916	       1.852	       1.966	       1.965	
 	
Change from baseline at Week 52					
  n	      90	      46	      46	     100	
  Adjusted mean	       0.066	       0.016	       0.036	       0.009	
  Standard error	       0.0206	       0.0286	       0.0285	       0.0195	
					
					
					
  p-value (compared with Placebo)		       0.1523	       0.3969	       0.0452	
 	
	
	
	
	


Analysis of change from baseline in tau PET SUVR scores for each target region and the primary reference region by MMRM - tau PET modified evaluable set: placebo-controlled period	
	
Tau PET Braak 1 and 2 Staging SUVR measure (Reference Region = Cerebellum Superior Cropped)	
	Placebo
(N=98)	BIIB092
Low Dose
(N=53)	BIIB092
600mg/4wk
(N=51)	BIIB092
2000mg/4wk
(N=115)	
 	
Change from baseline at Week 78					
  n	      92	      49	      48	      98	
  Adjusted mean	       0.047	       0.033	       0.054	      -0.012	
  Standard error	       0.0226	       0.0309	       0.0312	       0.0218	
					
					
					
					
  p-value (compared with Placebo)		       0.7276	       0.8535	       0.0606	
 	
	
	
	
	


Analysis of change from baseline in tau PET SUVR scores for each target region and the primary reference region by MMRM - tau PET modified evaluable set: placebo-controlled period	
	
Tau PET Braak 3 and 4 Staging SUVR measure (Reference Region = Cerebellum Superior Cropped)	
	Placebo
(N=98)	BIIB092
Low Dose
(N=53)	BIIB092
600mg/4wk
(N=51)	BIIB092
2000mg/4wk
(N=115)	
 	
Baseline					
  n	      98	      52	      51	     112	
  Mean	       1.858	       1.929	       1.928	       1.909	
 	
Change from baseline at Week 52					
  n	      90	      46	      46	     100	
  Adjusted mean	       0.155	       0.081	       0.129	       0.104	
  Standard error	       0.0213	       0.0293	       0.0294	       0.0201	
  p-value (compared with Placebo)		       0.0386	       0.4692	       0.0762	
 	
	
	
	
	


Analysis of change from baseline in tau PET SUVR scores for each target region and the primary reference region by MMRM - tau PET modified evaluable set: placebo-controlled period	
	
Tau PET Braak 3 and 4 Staging SUVR measure (Reference Region = Cerebellum Superior Cropped)	
	Placebo
(N=98)	BIIB092
Low Dose
(N=53)	BIIB092
600mg/4wk
(N=51)	BIIB092
2000mg/4wk
(N=115)	
 	
Change from baseline at Week 78					
  n	      92	      49	      48	      98	
  Adjusted mean	       0.177	       0.142	       0.178	       0.129	
  Standard error	       0.0250	       0.0340	       0.0345	       0.0239	
					
					
					
					
  p-value (compared with Placebo)		       0.4018	       0.9877	       0.1598	
 	
	
	
	
	


Analysis of change from baseline in tau PET SUVR scores for each target region and the primary reference region by MMRM - tau PET modified evaluable set: placebo-controlled period	
	
Tau PET Braak 5 and 6 Staging SUVR measure (Reference Region = Cerebellum Superior Cropped)	
	Placebo
(N=98)	BIIB092
Low Dose
(N=53)	BIIB092
600mg/4wk
(N=51)	BIIB092
2000mg/4wk
(N=115)	
 	
Baseline					
  n	      98	      52	      51	     112	
  Mean	       1.707	       1.758	       1.766	       1.773	
 	
Change from baseline at Week 52					
  n	      90	      46	      46	     100	
  Adjusted mean	       0.159	       0.091	       0.131	       0.111	
  Standard error	       0.0193	       0.0266	       0.0266	       0.0182	
					
					
					
  p-value (compared with Placebo)		       0.0383	       0.3953	       0.0685	
 	
	
	
	
	


Analysis of change from baseline in tau PET SUVR scores for each target region and the primary reference region by MMRM - tau PET modified evaluable set: placebo-controlled period	
	
Tau PET Braak 5 and 6 Staging SUVR measure (Reference Region = Cerebellum Superior Cropped)	
	Placebo
(N=98)	BIIB092
Low Dose
(N=53)	BIIB092
600mg/4wk
(N=51)	BIIB092
2000mg/4wk
(N=115)	
 	
Change from baseline at Week 78					
  n	      92	      49	      48	      98	
  Adjusted mean	       0.180	       0.168	       0.175	       0.135	
  Standard error	       0.0254	       0.0347	       0.0351	       0.0245	
					
					
					
					
  p-value (compared with Placebo)		       0.7893	       0.9133	       0.2022	
 	
	
	
	
	
