# Supplementary material for: TANGO: a placebo-controlled randomized phase 2 study of efficacy and safety of the anti-tau monoclonal antibody gosuranemab in early Alzheimer’s disease
Source: Nat Aging. 2023 Nov 27;3(12):1591–601. doi: 10.1038/s43587-023-00523-w (PMC10724064; doi:10.1038/s43587-023-00523-w)
Supplement: Supplementary file 8 — Statistical source data. [file 43587_2023_523_MOESM8_ESM.zip › Extended data figure 1_Source data (1).rtf]

Analysis of change from baseline in ADAS-Cog 13 by MMRM - full analysis set: placebo-controlled period	
	
	Placebo
(N=214)	BIIB092
Low Dose
(N=116)	BIIB092
600mg/4wk
(N=106)	BIIB092
2000mg/4wk
(N=214)	
 	
Baseline					
  n	      214	      116	      106	      214	
  Mean	       26.40	       25.88	       27.14	       25.27	
 	
Change from baseline at Week 24					
  n	      204	      109	      102	      207	
  Adjusted mean	        0.74	        1.57	        0.80	        1.13	
  Standard error	        0.412	        0.509	        0.520	        0.406	
					
					
					
  p-value (compared with Placebo)		        0.1480	        0.9246	        0.4183	
 	
	
	
	
	


Analysis of change from baseline in ADAS-Cog 13 by MMRM - full analysis set: placebo-controlled period	
	
	Placebo
(N=214)	BIIB092
Low Dose
(N=116)	BIIB092
600mg/4wk
(N=106)	BIIB092
2000mg/4wk
(N=214)	
 	
Change from baseline at Week 52					
  n	      174	       98	       95	      179	
  Adjusted mean	        2.19	        3.26	        3.86	        3.19	
  Standard error	        0.515	        0.651	        0.663	        0.509	
					
					
					
  p-value (compared with Placebo)		        0.1618	        0.0320	        0.1211	
 	
Change from baseline at Week 78					
  n	      170	       96	       90	      173	
  Adjusted mean	        5.78	        7.51	        7.57	        7.46	
  Standard error	        0.623	        0.801	        0.822	        0.617	
					
					
					
					
  p-value (compared with Placebo)		        0.0719	        0.0681	        0.0378	
 	
	
	
	
	


Analysis of change from baseline in MMSE by MMRM - full analysis set: placebo-controlled period	
	
	Placebo
(N=214)	BIIB092
Low Dose
(N=116)	BIIB092
600mg/4wk
(N=106)	BIIB092
2000mg/4wk
(N=214)	
 	
Baseline					
  n	   214	   116	   106	   214	
  Mean	    25.38	    25.36	    25.10	    25.43	
 	
Change from baseline at Week 12					
  n	   209	   111	   104	   206	
  Adjusted mean	     -1.48	     -1.48	     -1.50	     -1.39	
  Standard error	      0.234	      0.293	      0.301	      0.233	
					
					
					
  p-value (compared with Placebo)		      0.9801	      0.9744	      0.7327	
 	
	
	
	
	


Analysis of change from baseline in MMSE by MMRM - full analysis set: placebo-controlled period	
	
	Placebo
(N=214)	BIIB092
Low Dose
(N=116)	BIIB092
600mg/4wk
(N=106)	BIIB092
2000mg/4wk
(N=214)	
 	
Change from baseline at Week 24					
  n	   202	   109	   104	   203	
  Adjusted mean	     -1.70	     -1.98	     -2.01	     -1.72	
  Standard error	      0.244	      0.306	      0.313	      0.242	
					
					
					
  p-value (compared with Placebo)		      0.4339	      0.3912	      0.9574	
 	
Change from baseline at Week 40					
  n	   174	   103	    93	   181	
  Adjusted mean	     -2.03	     -2.69	     -2.50	     -2.23	
  Standard error	      0.271	      0.339	      0.351	      0.267	
					
					
					
  p-value (compared with Placebo)		      0.0972	      0.2465	      0.5377	
 	
	
	
	
	


Analysis of change from baseline in MMSE by MMRM - full analysis set: placebo-controlled period	
	
	Placebo
(N=214)	BIIB092
Low Dose
(N=116)	BIIB092
600mg/4wk
(N=106)	BIIB092
2000mg/4wk
(N=214)	
 	
Change from baseline at Week 52					
  n	   170	    95	    89	   173	
  Adjusted mean	     -2.25	     -2.93	     -2.88	     -2.53	
  Standard error	      0.290	      0.369	      0.380	      0.287	
					
					
					
  p-value (compared with Placebo)		      0.1203	      0.1611	      0.4582	
 	
Change from baseline at Week 68					
  n	   157	    90	    89	   167	
  Adjusted mean	     -3.07	     -3.56	     -3.58	     -3.44	
  Standard error	      0.318	      0.408	      0.417	      0.314	
					
					
					
  p-value (compared with Placebo)		      0.3203	      0.3089	      0.3710	
 	
	
	
	
	


Analysis of change from baseline in MMSE by MMRM - full analysis set: placebo-controlled period	
	
	Placebo
(N=214)	BIIB092
Low Dose
(N=116)	BIIB092
600mg/4wk
(N=106)	BIIB092
2000mg/4wk
(N=214)	
 	
Change from baseline at Week 78					
  n	   173	    99	    91	   174	
  Adjusted mean	     -3.32	     -4.11	     -4.13	     -3.66	
  Standard error	      0.345	      0.446	      0.460	      0.343	
					
					
					
					
  p-value (compared with Placebo)		      0.1415	      0.1415	      0.4459	
 	
	
	
	
	


Analysis of change from baseline in ADCS-ADL by MMRM - full analysis set: placebo-controlled period	
	
	Placebo
(N=214)	BIIB092
Low Dose
(N=116)	BIIB092
600mg/4wk
(N=106)	BIIB092
2000mg/4wk
(N=214)	
 	
Baseline					
  n	    212	    113	    103	    214	
  Mean	     69.35	     69.06	     67.63	     69.50	
 	
Change from baseline at Week 24					
  n	    200	    105	    100	    206	
  Adjusted mean	     -0.87	     -2.21	     -0.49	     -1.27	
  Standard error	      0.447	      0.557	      0.569	      0.437	
					
					
					
  p-value (compared with Placebo)		      0.0326	      0.5445	      0.4403	
 	
	
	
	
	


Analysis of change from baseline in ADCS-ADL by MMRM - full analysis set: placebo-controlled period	
	
	Placebo
(N=214)	BIIB092
Low Dose
(N=116)	BIIB092
600mg/4wk
(N=106)	BIIB092
2000mg/4wk
(N=214)	
 	
Change from baseline at Week 52					
  n	    171	     92	     89	    177	
  Adjusted mean	     -3.18	     -3.40	     -3.74	     -3.79	
  Standard error	      0.564	      0.723	      0.738	      0.555	
					
					
					
  p-value (compared with Placebo)		      0.7943	      0.5209	      0.3869	
 	
Change from baseline at Week 78					
  n	    170	     96	     88	    174	
  Adjusted mean	     -5.08	     -6.54	     -6.13	     -5.14	
  Standard error	      0.685	      0.885	      0.916	      0.676	
					
					
					
					
  p-value (compared with Placebo)		      0.1685	      0.3336	      0.9429	
 	
	
	
	
	
