# Supplementary material for: TANGO: a placebo-controlled randomized phase 2 study of efficacy and safety of the anti-tau monoclonal antibody gosuranemab in early Alzheimer’s disease
Source: Nat Aging. 2023 Nov 27;3(12):1591–601. doi: 10.1038/s43587-023-00523-w (PMC10724064; doi:10.1038/s43587-023-00523-w)
Supplement: Supplementary file 9 — Statistical source data. [file 43587_2023_523_MOESM9_ESM.zip › Extended data figure 2_Source data (1).rtf]

Analysis of change from baseline in FAQ by MMRM - full analysis set: placebo-controlled period	
	
	Placebo
(N=214)	BIIB092
Low Dose
(N=116)	BIIB092
600mg/4wk
(N=106)	BIIB092
2000mg/4wk
(N=214)	
 	
Baseline					
  n	    212	    114	    103	    214	
  Mean	      8.13	      8.39	      9.93	      8.07	
 	
Change from baseline at Week 24					
  n	    200	    106	     99	    207	
  Adjusted mean	      0.78	      1.06	      0.26	      0.58	
  Standard error	      0.389	      0.484	      0.496	      0.381	
					
					
					
  p-value (compared with Placebo)		      0.6088	      0.3466	      0.6586	
 	
	
	
	
	


Analysis of change from baseline in FAQ by MMRM - full analysis set: placebo-controlled period	
	
	Placebo
(N=214)	BIIB092
Low Dose
(N=116)	BIIB092
600mg/4wk
(N=106)	BIIB092
2000mg/4wk
(N=214)	
 	
Change from baseline at Week 52					
  n	    172	     94	     89	    176	
  Adjusted mean	      2.97	      2.55	      2.49	      2.89	
  Standard error	      0.466	      0.591	      0.607	      0.459	
					
					
					
  p-value (compared with Placebo)		      0.5422	      0.4953	      0.8886	
 	
Change from baseline at Week 78					
  n	    168	     95	     86	    172	
  Adjusted mean	      4.56	      4.64	      4.04	      4.16	
  Standard error	      0.508	      0.645	      0.670	      0.500	
					
					
					
					
  p-value (compared with Placebo)		      0.9139	      0.5100	      0.5320	
 	
	
	
	
	
