# Supplementary material for: TANGO: a placebo-controlled randomized phase 2 study of efficacy and safety of the anti-tau monoclonal antibody gosuranemab in early Alzheimer’s disease
Source: Nat Aging. 2023 Nov 27;3(12):1591–601. doi: 10.1038/s43587-023-00523-w (PMC10724064; doi:10.1038/s43587-023-00523-w)
Supplement: Supplementary file 10 — Statistical source data. [file 43587_2023_523_MOESM10_ESM.zip › Extended data figure 3_Source data (1).rtf]

Analysis of change from baseline in tau PET SUVR scores for secondary target region (RR=cerebellum superior cropped) by MMRM - tau PET modified evaluable set: placebo-controlled period	
	
Tau PET Frontal lobe SUVR measure (Reference Region=Cerebellum Superior Cropped)	
	Placebo
(N=98)	BIIB092
Low Dose
(N=53)	BIIB092
600mg/4wk
(N=51)	BIIB092
2000mg/4wk
(N=115)	
 	
Baseline					
  n	      98	      52	      51	     112	
  Mean	       1.589	       1.631	       1.572	       1.603	
 	
Change from baseline at Week 52					
  n	      90	      46	      46	     100	
  Adjusted mean	       0.168	       0.096	       0.136	       0.117	
  Standard error	       0.0192	       0.0265	       0.0265	       0.0181	
					
					
					
  p-value (compared with Placebo)		       0.0263	       0.3219	       0.0460	
 	
	
	
	
	


Analysis of change from baseline in tau PET SUVR scores for secondary target region (RR=cerebellum superior cropped) by MMRM - tau PET modified evaluable set: placebo-controlled period	
	
Tau PET Frontal lobe SUVR measure (Reference Region=Cerebellum Superior Cropped)	
	Placebo
(N=98)	BIIB092
Low Dose
(N=53)	BIIB092
600mg/4wk
(N=51)	BIIB092
2000mg/4wk
(N=115)	
 	
Change from baseline at Week 78					
  n	      92	      49	      48	      98	
  Adjusted mean	       0.197	       0.170	       0.180	       0.147	
  Standard error	       0.0250	       0.0342	       0.0345	       0.0241	
					
					
					
					
  p-value (compared with Placebo)		       0.5160	       0.6830	       0.1478	
 	
	
	
	
	


Analysis of change from baseline in tau PET SUVR scores for secondary target region (RR=cerebellum superior cropped) by MMRM - tau PET modified evaluable set: placebo-controlled period	
	
Tau PET Lateral temporal cortex SUVR measure (Reference Region=Cerebellum Superior Cropped)	
	Placebo
(N=98)	BIIB092
Low Dose
(N=53)	BIIB092
600mg/4wk
(N=51)	BIIB092
2000mg/4wk
(N=115)	
 	
Baseline					
  n	      98	      52	      51	     112	
  Mean	       2.238	       2.353	       2.318	       2.306	
 	
Change from baseline at Week 52					
  n	      90	      46	      46	     100	
  Adjusted mean	       0.198	       0.094	       0.162	       0.138	
  Standard error	       0.0290	       0.0399	       0.0401	       0.0274	
					
					
					
  p-value (compared with Placebo)		       0.0329	       0.4578	       0.1229	
 	
	
	
	
	


Analysis of change from baseline in tau PET SUVR scores for secondary target region (RR=cerebellum superior cropped) by MMRM - tau PET modified evaluable set: placebo-controlled period	
	
Tau PET Lateral temporal cortex SUVR measure (Reference Region=Cerebellum Superior Cropped)	
	Placebo
(N=98)	BIIB092
Low Dose
(N=53)	BIIB092
600mg/4wk
(N=51)	BIIB092
2000mg/4wk
(N=115)	
 	
Change from baseline at Week 78					
  n	      92	      49	      48	      98	
  Adjusted mean	       0.215	       0.170	       0.216	       0.166	
  Standard error	       0.0337	       0.0459	       0.0465	       0.0323	
					
					
					
					
  p-value (compared with Placebo)		       0.4204	       0.9938	       0.2799	
 	
	
	
	
	


Analysis of change from baseline in tau PET SUVR scores for secondary target region (RR=cerebellum superior cropped) by MMRM - tau PET modified evaluable set: placebo-controlled period	
	
Tau PET Medial temporal lobe roi SUVR measure (Reference Region=Cerebellum Superior Cropped)	
	Placebo
(N=98)	BIIB092
Low Dose
(N=53)	BIIB092
600mg/4wk
(N=51)	BIIB092
2000mg/4wk
(N=115)	
 	
Baseline					
  n	      98	      52	      51	     112	
  Mean	       2.118	       2.209	       2.224	       2.214	
 	
Change from baseline at Week 52					
  n	      90	      46	      46	     100	
  Adjusted mean	       0.161	       0.084	       0.129	       0.099	
  Standard error	       0.0261	       0.0358	       0.0360	       0.0246	
					
					
					
  p-value (compared with Placebo)		       0.0822	       0.4721	       0.0824	
 	
	
	
	
	


Analysis of change from baseline in tau PET SUVR scores for secondary target region (RR=cerebellum superior cropped) by MMRM - tau PET modified evaluable set: placebo-controlled period	
	
Tau PET Medial temporal lobe roi SUVR measure (Reference Region=Cerebellum Superior Cropped)	
	Placebo
(N=98)	BIIB092
Low Dose
(N=53)	BIIB092
600mg/4wk
(N=51)	BIIB092
2000mg/4wk
(N=115)	
 	
Change from baseline at Week 78					
  n	      92	      49	      48	      98	
  Adjusted mean	       0.180	       0.143	       0.189	       0.122	
  Standard error	       0.0300	       0.0408	       0.0413	       0.0287	
					
					
					
					
  p-value (compared with Placebo)		       0.4640	       0.8601	       0.1577	
 	
	
	
	
	
