# Supplementary material for: TANGO: a placebo-controlled randomized phase 2 study of efficacy and safety of the anti-tau monoclonal antibody gosuranemab in early Alzheimer’s disease
Source: Nat Aging. 2023 Nov 27;3(12):1591–601. doi: 10.1038/s43587-023-00523-w (PMC10724064; doi:10.1038/s43587-023-00523-w)
Supplement: Supplementary file 11 — Statistical source data. [file 43587_2023_523_MOESM11_ESM.zip › Extended data figure 4_Source data (1).rtf]

Analysis of change from baseline in structural MRI for primary ROI measurements by MMRM - structural MRI evaluable set: placebo-controlled period	
	
Hippocampus Volume	
	Placebo
(N=190)	BIIB092
Low Dose
(N=109)	BIIB092
600mg/4wk
(N=94)	BIIB092
2000mg/4wk
(N=195)	
 	
Baseline					
  n	    178	    104	     92	    189	
  Mean	      4.08	      4.09	      4.06	      4.09	
 	
Change from baseline at Week 28					
  n	     170	     101	      89	     188	
  Adjusted mean	      -0.10	      -0.10	      -0.11	      -0.11	
  Standard error	       0.009	       0.011	       0.011	       0.009	
					
					
					
  p-value (compared with Placebo)		       0.9767	       0.4922	       0.4934	
 	


Analysis of change from baseline in structural MRI for primary ROI measurements by MMRM - structural MRI evaluable set: placebo-controlled period	
	
Hippocampus Volume	
	Placebo
(N=190)	BIIB092
Low Dose
(N=109)	BIIB092
600mg/4wk
(N=94)	BIIB092
2000mg/4wk
(N=195)	
 	
Change from baseline at Week 52					
  n	     149	      82	      81	     162	
  Adjusted mean	      -0.17	      -0.19	      -0.18	      -0.18	
  Standard error	       0.010	       0.012	       0.013	       0.010	
					
					
					
  p-value (compared with Placebo)		       0.2558	       0.2995	       0.4569	
 	
Change from baseline at Week 78					
  n	     140	      79	      78	     151	
  Adjusted mean	      -0.25	      -0.26	      -0.25	      -0.25	
  Standard error	       0.012	       0.015	       0.015	       0.011	
					
					
					
					
  p-value (compared with Placebo)		       0.6192	       0.8757	       0.9920	
 	


Analysis of change from baseline in structural MRI for primary ROI measurements by MMRM - structural MRI evaluable set: placebo-controlled period	
	
Whole Brain Volume	
	Placebo
(N=190)	BIIB092
Low Dose
(N=109)	BIIB092
600mg/4wk
(N=94)	BIIB092
2000mg/4wk
(N=195)	
 	
Baseline					
  n	      189	      109	       94	      194	
  Mean	     1086.56	     1084.32	     1063.18	     1071.34	
 	
Change from baseline at Week 28					
  n	       188	       108	        93	       194	
  Adjusted mean	     -11.64	     -12.19	     -12.38	     -11.51	
  Standard error	       1.026	       1.245	       1.313	       1.009	
					
					
					
  p-value (compared with Placebo)		       0.6978	       0.6199	       0.9139	
 	
	
	
	
	


Analysis of change from baseline in structural MRI for primary ROI measurements by MMRM - structural MRI evaluable set: placebo-controlled period	
	
Whole Brain Volume	
	Placebo
(N=190)	BIIB092
Low Dose
(N=109)	BIIB092
600mg/4wk
(N=94)	BIIB092
2000mg/4wk
(N=195)	
 	
Change from baseline at Week 52					
  n	       162	        91	        83	       172	
  Adjusted mean	     -18.21	     -19.35	     -19.52	     -19.22	
  Standard error	       1.222	       1.526	       1.600	       1.199	
					
					
					
  p-value (compared with Placebo)		       0.5256	       0.4831	       0.5054	
 	
Change from baseline at Week 78					
  n	       156	        90	        81	       160	
  Adjusted mean	     -27.52	     -27.89	     -28.44	     -28.00	
  Standard error	       1.389	       1.751	       1.843	       1.367	
					
					
					
					
  p-value (compared with Placebo)		       0.8606	       0.6729	       0.7873	
 	
	
	
	
	


Analysis of change from baseline in structural MRI for primary ROI measurements by MMRM - structural MRI evaluable set: placebo-controlled period	
	
Lateral Ventricle Volume	
	Placebo
(N=190)	BIIB092
Low Dose
(N=109)	BIIB092
600mg/4wk
(N=94)	BIIB092
2000mg/4wk
(N=195)	
 	
Baseline					
  n	     181	     106	      93	     189	
  Mean	      43.32	      45.04	      46.43	      41.01	
 	
Change from baseline at Week 28					
  n	     178	     104	      91	     187	
  Adjusted mean	       2.61	       2.60	       2.88	       2.82	
  Standard error	       0.176	       0.213	       0.225	       0.172	
					
					
					
  p-value (compared with Placebo)		       0.9662	       0.2999	       0.3213	
 	
	
	
	
	


Analysis of change from baseline in structural MRI for primary ROI measurements by MMRM - structural MRI evaluable set: placebo-controlled period	
Lateral Ventricle Volume	
	Placebo
(N=190)	BIIB092
Low Dose
(N=109)	BIIB092
600mg/4wk
(N=94)	BIIB092
2000mg/4wk
(N=195)	
 	
Change from baseline at Week 52					
  n	     150	      87	      82	     168	
  Adjusted mean	       4.46	       4.91	       5.08	       5.32	
  Standard error	       0.257	       0.325	       0.343	       0.249	
					
					
					
  p-value (compared with Placebo)		       0.2600	       0.1343	       0.0112	
 	
Change from baseline at Week 78					
  n	     143	      85	      77	     154	
  Adjusted mean	       7.03	       7.35	       7.36	       7.98	
  Standard error	       0.355	       0.455	       0.481	       0.344	
					
					
					
					
  p-value (compared with Placebo)		       0.5692	       0.5711	       0.0481	
 	
	
	
	
	
